# Supplementary material for: Extracellular vesicles adhere to cells primarily by interactions of integrins and GM1 with laminin
Source: J Cell Biol. 2025 Apr 30;224(6):e202404064. doi: 10.1083/jcb.202404064 (PMC12042775; doi:10.1083/jcb.202404064)
Supplement: Table S1 — shows the numbers of sEVs labeled with tetraspanin–Halo7-TMR attached to glass coated with the ECM. [file jcb_202404064_tables1.docx]

**Supplemental Table 1.** The numbers of sEVs labeled with tetraspanin-Halo7-TMR attached to glass coated with the ECM.

| sEV | Integrin | Fibronectin | | | Collagen type I | | | Laminin | | |
| --- | --- | --- | --- | --- | --- | --- | --- | --- | --- | --- |
|  |  | Mean±SE | KO/WT^*^ | p value^†^ | Mean±SE | KO/WT | p value | Mean±SE | KO/WT | p value |
| CD63 | WT | 19±14.8 |  |  | 42±2.1 | 0.16±0.024 | 8.7×10^-16^ | 424±10.5 | 0.22±0.008 | 1.2×10^-21^ |
|  | β1KO | 15±3.5 |  |  | 7±1.0 |  |  | 93±2.7 |  |  |
|  | WT | 9±1.6 |  |  | 126±9.4 | 0.38±0.056 | 8.4×10^-7^ | 437±21.1 |  |  |
|  | α2KO | 20±4.7 |  |  | 48±6.0 |  |  | 380±8.6 |  |  |
|  | WT | 10±1.0 |  |  | 66±1.3 |  |  | 220±6.0 | 0.24±0.011 | 9.9×10^-16^ |
|  | α6KO | 19±1.0 |  |  | 76±1.6 |  |  | 52±2.0 |  |  |
|  | WT | 22±1.6 |  |  | 65±1.3 |  |  | 232±6.7 | 0.40±0.014 | 9.6×10^-16^ |
|  | β4KO | 9±0.8 |  |  | 73±1.5 |  |  | 93±1.8 |  |  |
| CD81 | WT | 8±2.9 |  |  | 108±7.0 | -0.02±0.076 | 1.8×10^-10^ | 376±9.1 | 0.21±0.009 | 4.4×10^-19^ |
|  | β1KO | 2±1.6 |  |  | -2±8.2 |  |  | 77±2.8 |  |  |
|  | WT | 39±2.7 |  |  | 88±16.2 | 0.20±0.057 | 6.9×10^-4^ | 223±6.9 |  |  |
|  | α2KO | 29±3.7 |  |  | 18±3.8 |  |  | 279±10.3 |  |  |
|  | WT | 2±1.6 |  |  | 62±2.1 |  |  | 403±16.4 | 0.46±0.027 | 1.0×10^-10^ |
|  | α6KO | 9±0.8 |  |  | 59±3.7 |  |  | 186±7.9 |  |  |
|  | WT | 0±0.1 |  |  | 51±2.5 |  |  | 393±11.4 | 0.31±0.011 | 3.2×10^-19^ |
|  | β4KO | 0±0.1 |  |  | 36±2.5 |  |  | 122±2.3 |  |  |
| CD9 | WT | 3±0.9 |  |  | 163±18.2 | 0.41±0.057 | 1.3×10^-4^ | 390±12.4 | 0.22±0.014 | 4.5×10^-15^ |
|  | β1KO | 3±0.9 |  |  | 68±5.3 |  |  | 87±4.6 |  |  |
|  | WT | -8±0.8 |  |  | 54±3.3 | 0.35±0.039 | 1.0×10^-10^ | 271±13.5 |  |  |
|  | α2KO | -2±0.7 |  |  | 19±1.7 |  |  | 220±7.3 |  |  |
|  | WT | -1±0.2 |  |  | 23±1.2 |  |  | 289±11.2 | 0.26±0.017 | 4.3×10^-14^ |
|  | α6KO | 5±1.6 |  |  | 39±2.4 |  |  | 74±4.0 |  |  |
|  | WT | 0±0.3 |  |  | 30±1.9 |  |  | 444±11.5 | 0.24±0.012 | 4.8×10^-24^ |
|  | β4KO | 0±0.4 |  |  | 37±2.0 |  |  | 106±4.4 |  |  |

*The ratio of the number of integrin KO PC3-derived sEVs bound to ECM components to the number of wild-type (WT) PC3-derived sEVs bound to ECM components is shown only when the ratio was less than 0.5 and the difference was greater than 20.

^†^p values of Welch’s t-test (two-sided) are shown.
