# Supplementary material for: Extracellular vesicles adhere to cells primarily by interactions of integrins and GM1 with laminin
Source: J Cell Biol. 2025 Apr 30;224(6):e202404064. doi: 10.1083/jcb.202404064 (PMC12042775; doi:10.1083/jcb.202404064)

Fig. 1A

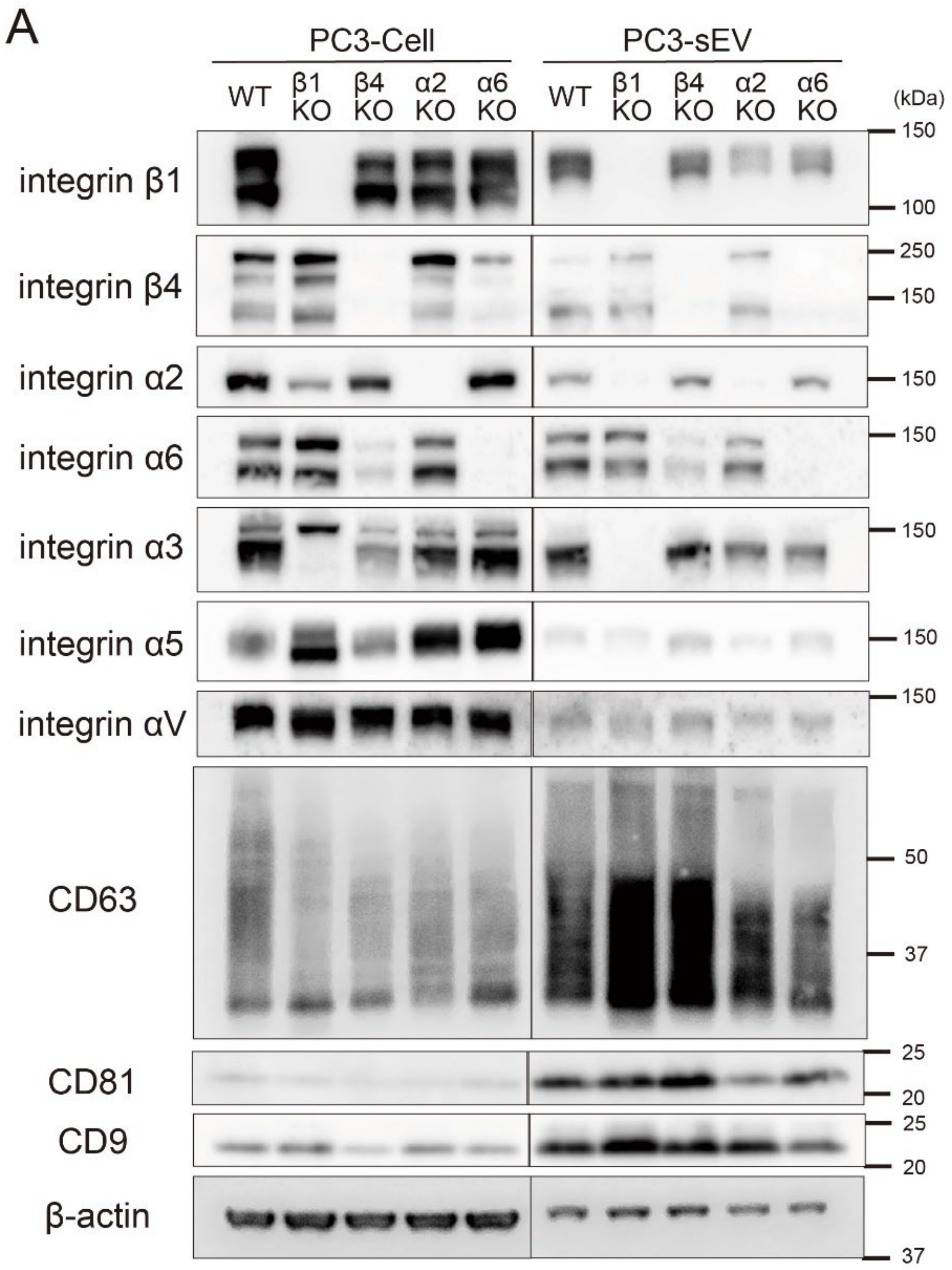
A
PC3-Cell
PC3-sEV
WT
β1 KO
β4 KO
α2 KO
α6 KO
(kDa)
integrin β1
integrin β4
integrin α2
integrin α6
integrin α3
integrin α5
integrin αV
CD63
CD81
CD9
β-actin
150
100
250
50
37
25
20

SourceDataF1A-Integrin β1

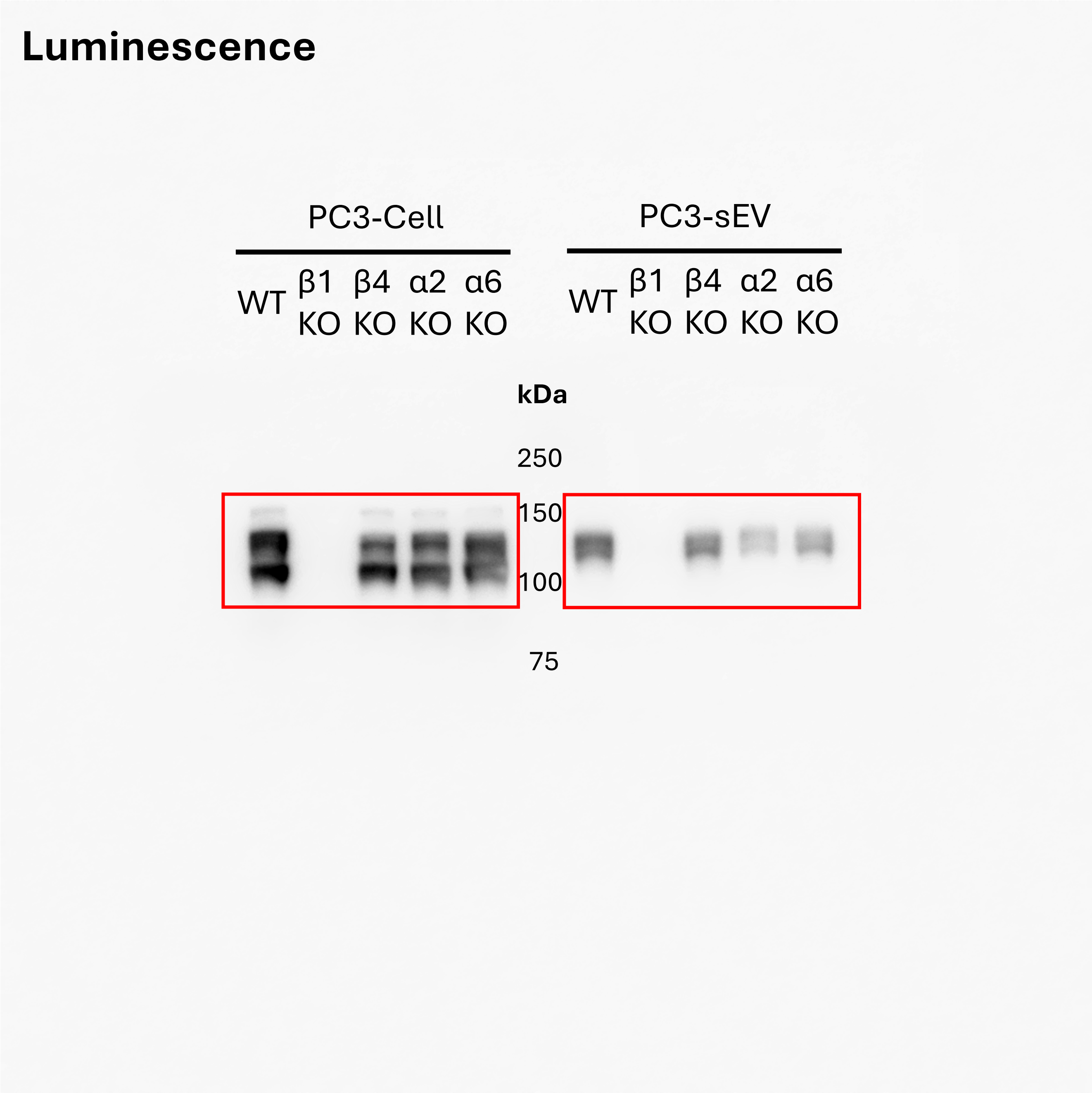

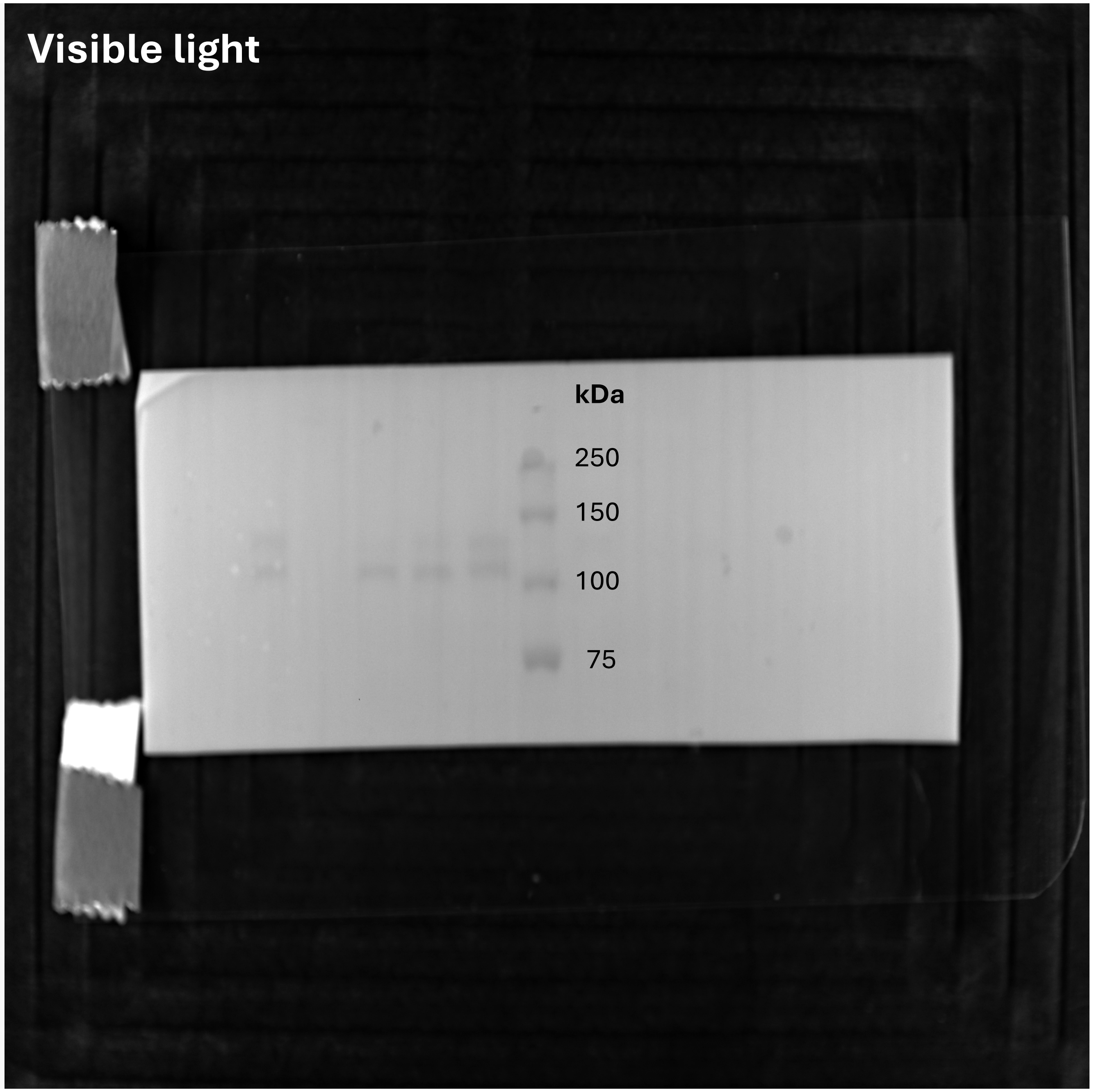

SourceDataF1A-Integrin β4

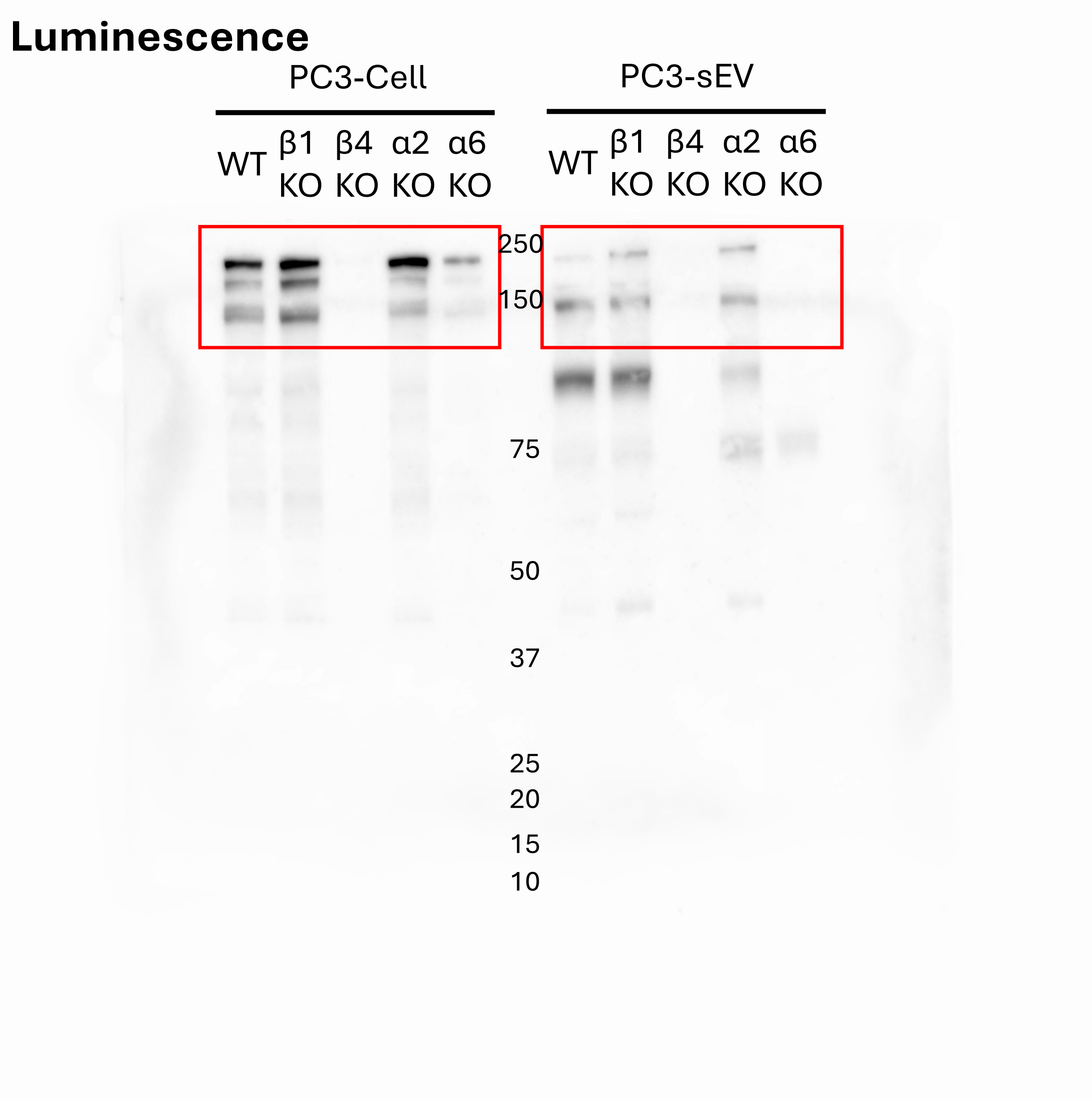

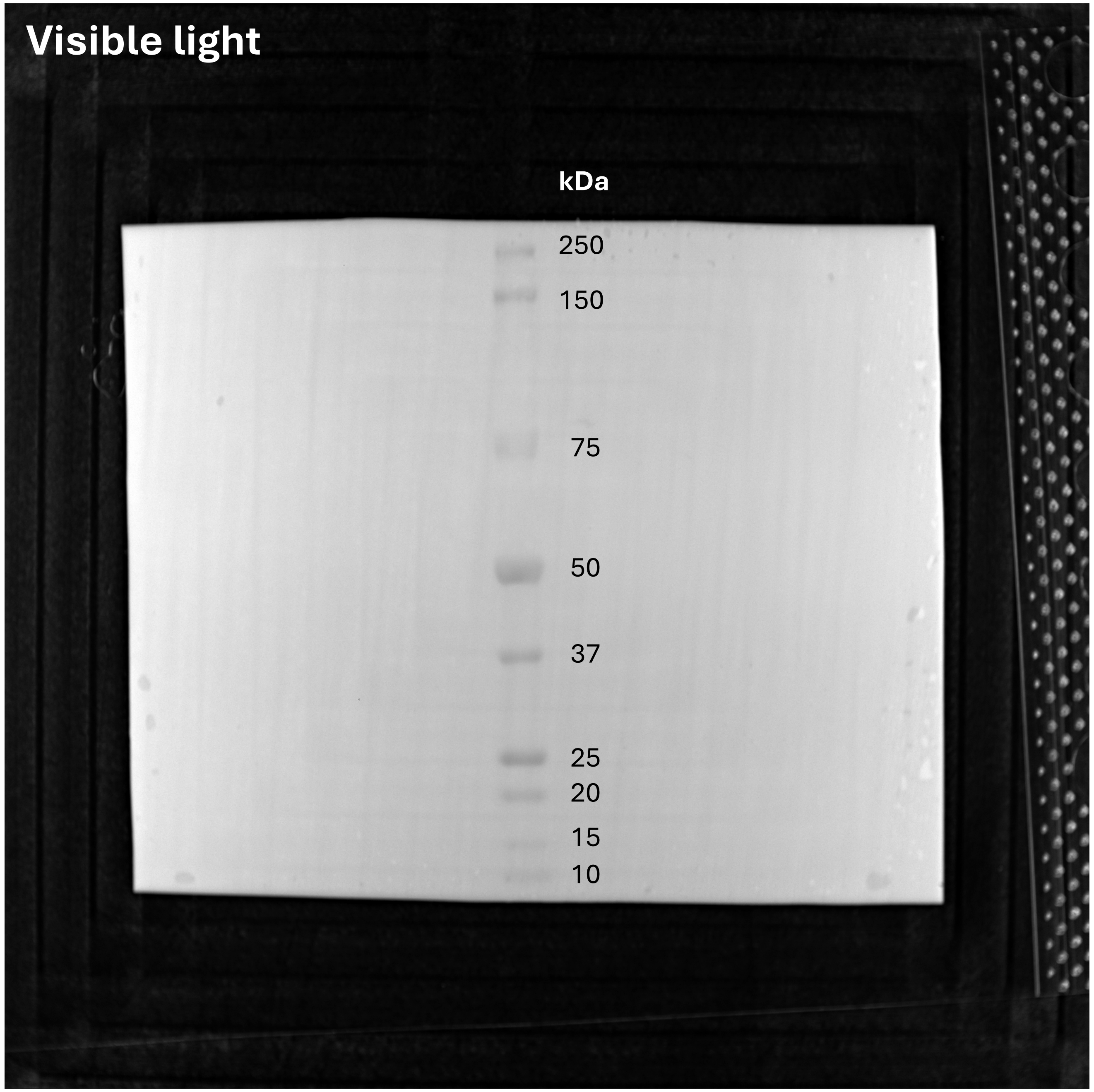

SourceDataF1A-Integrin α2

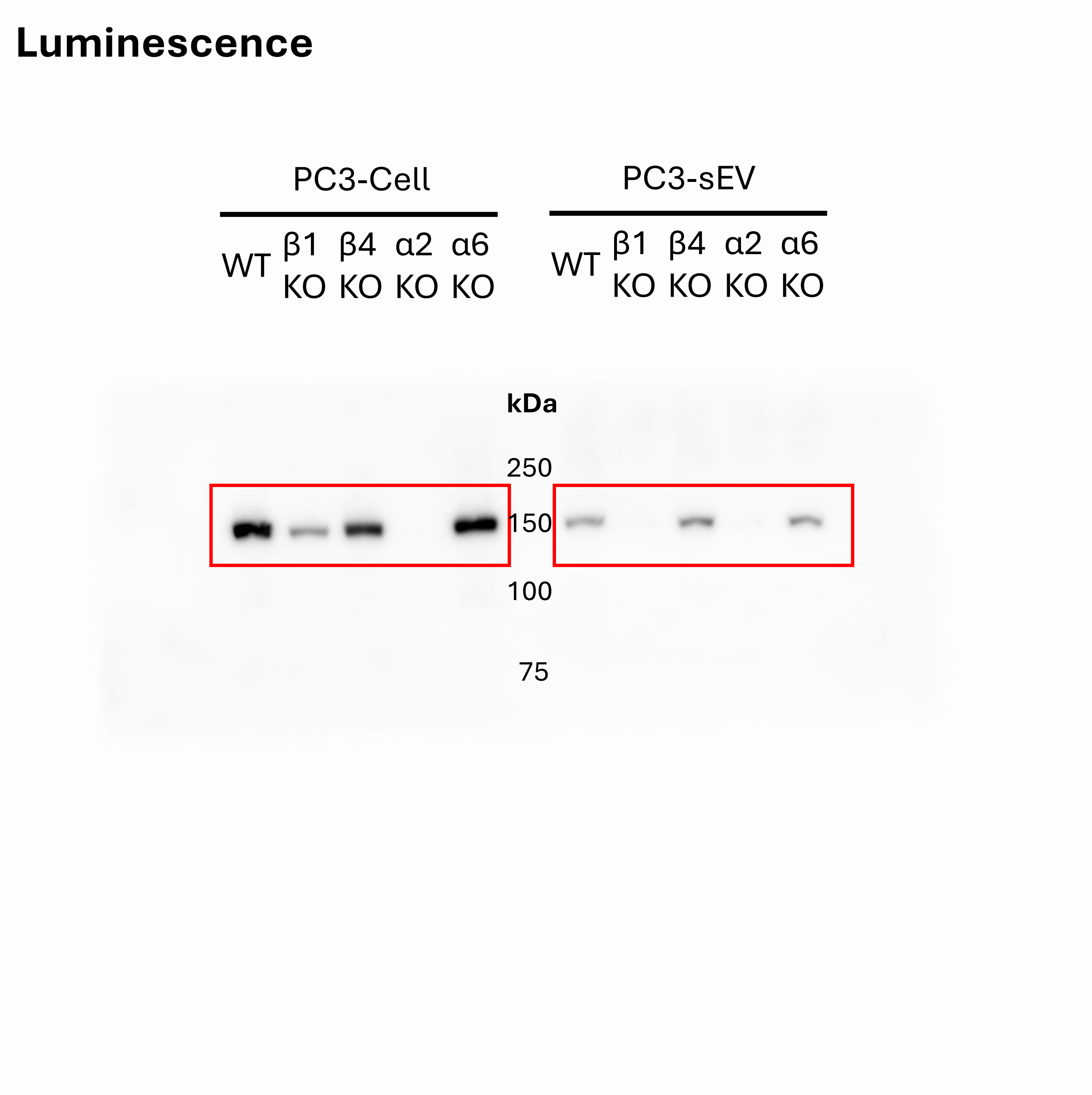

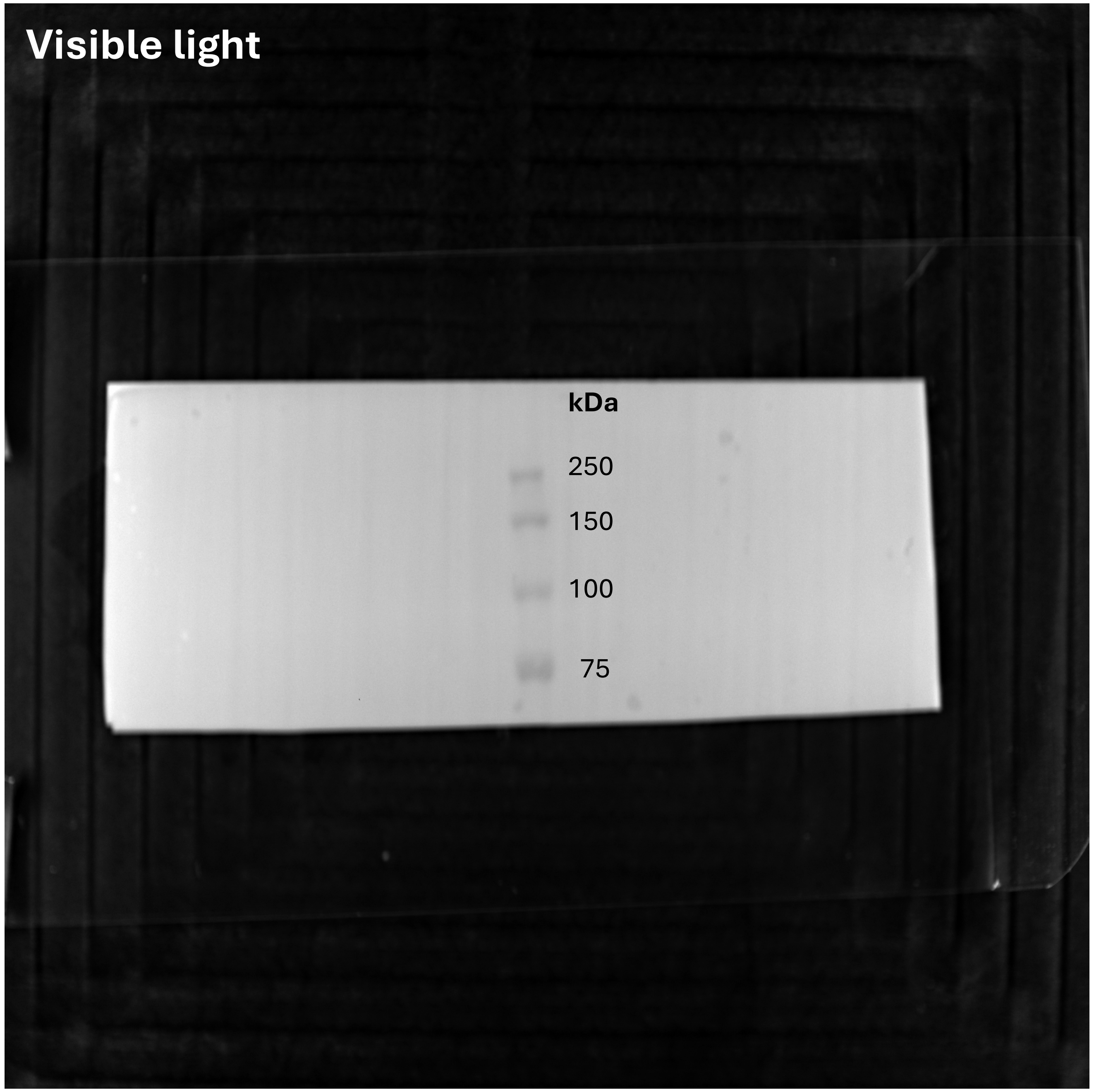

SourceDataF1A-Integrin α6

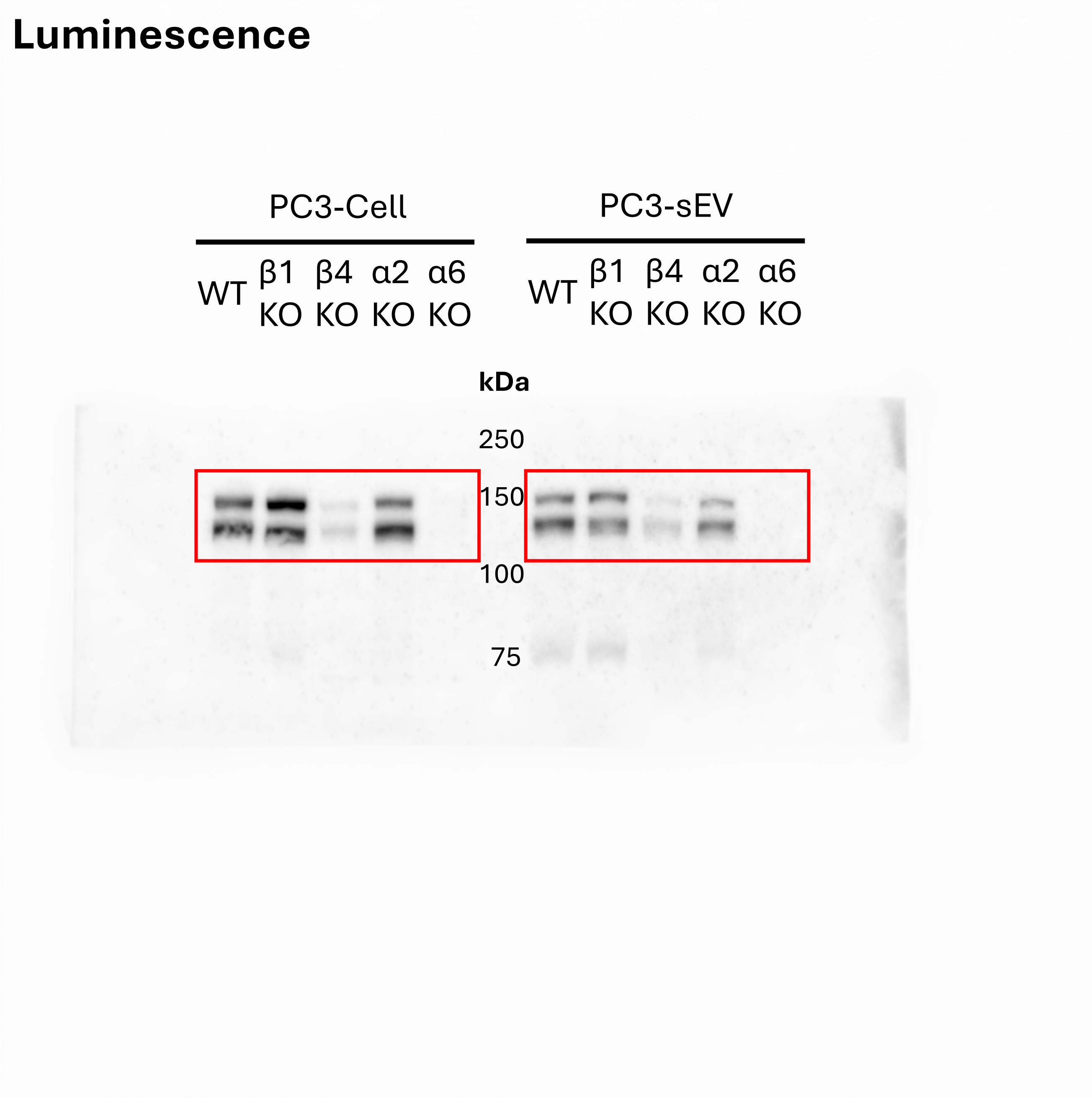

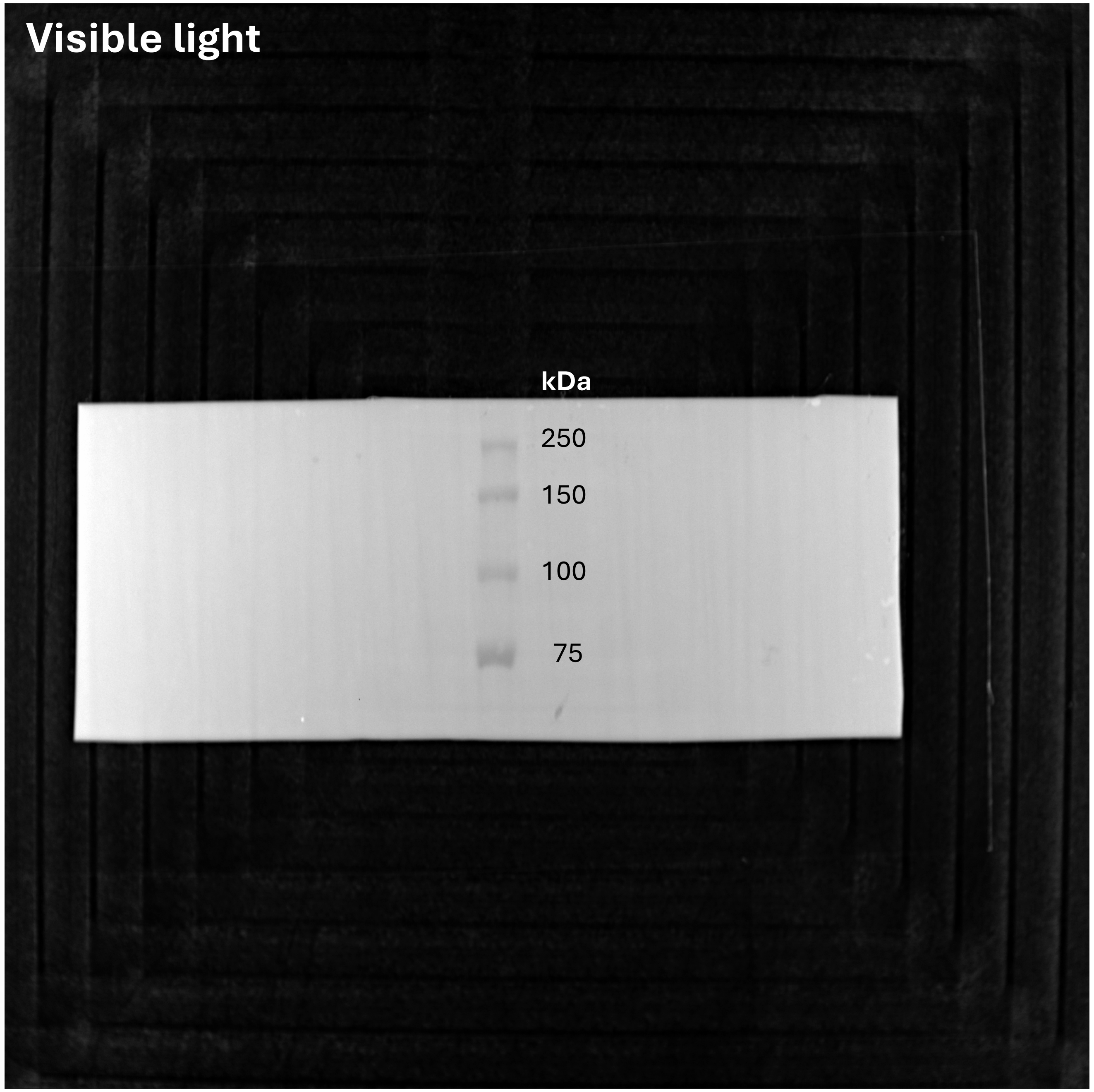

SourceDataF1A-Integrin α3

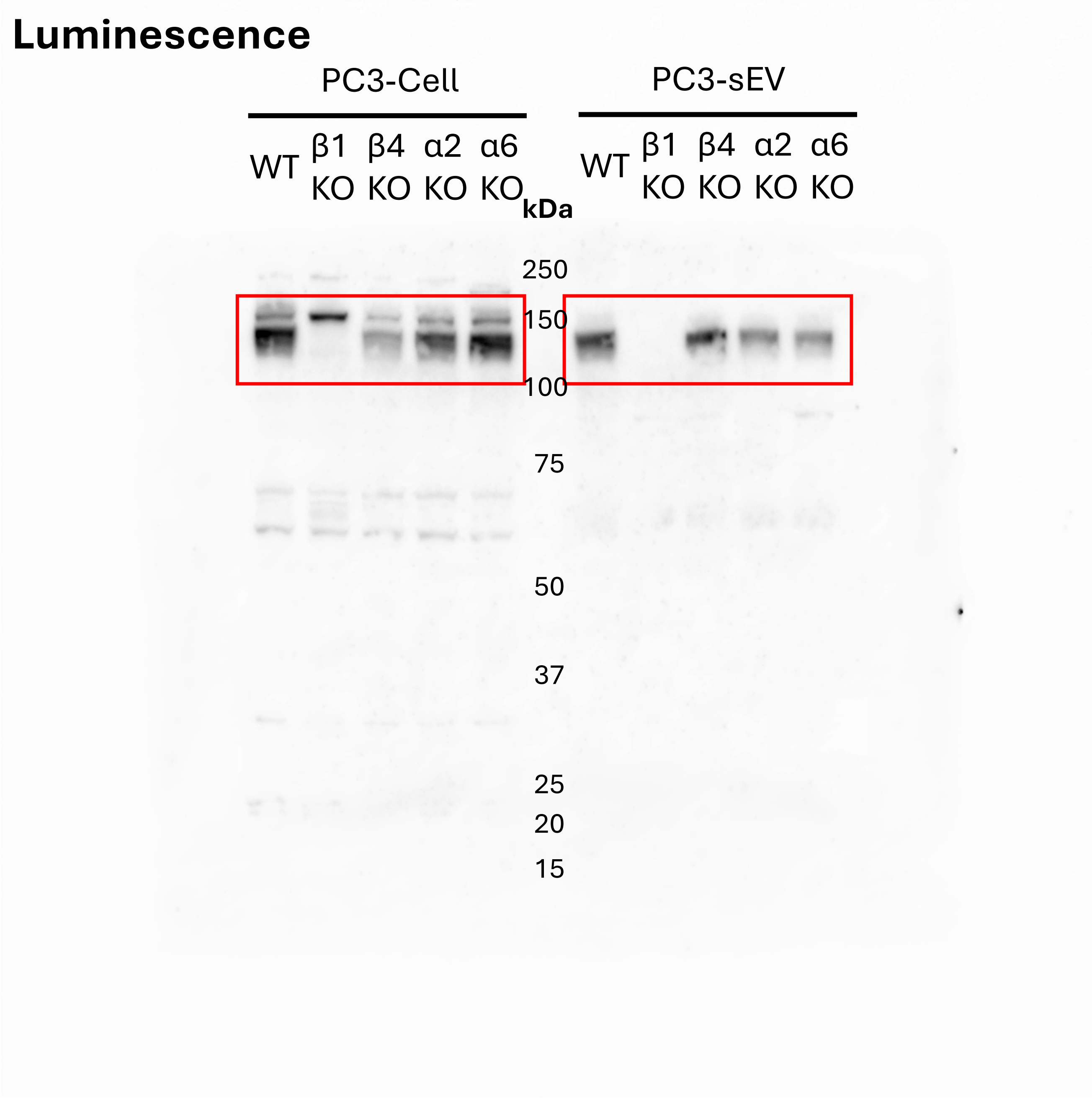

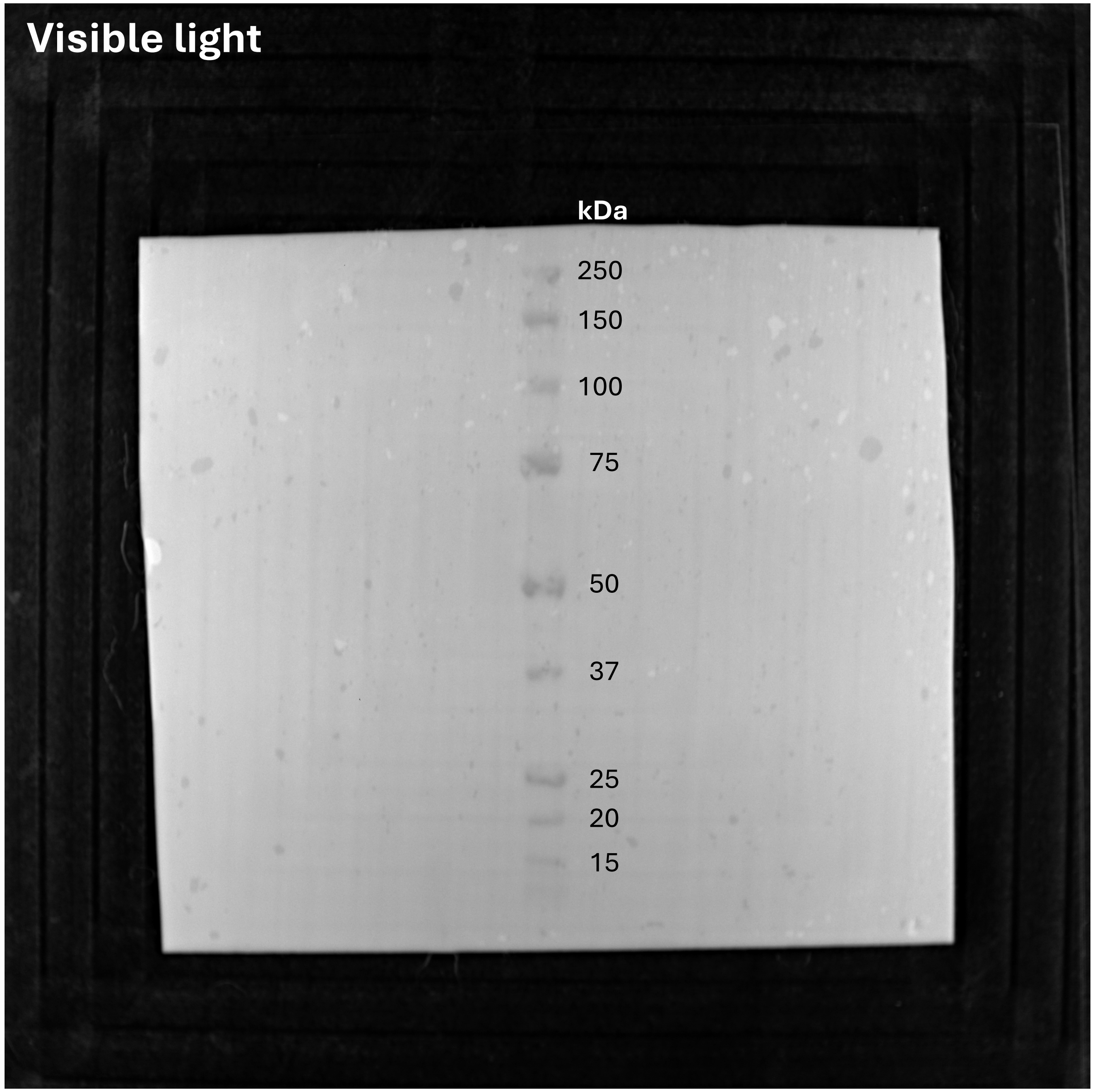

SourceDataF1A-Integrin α5

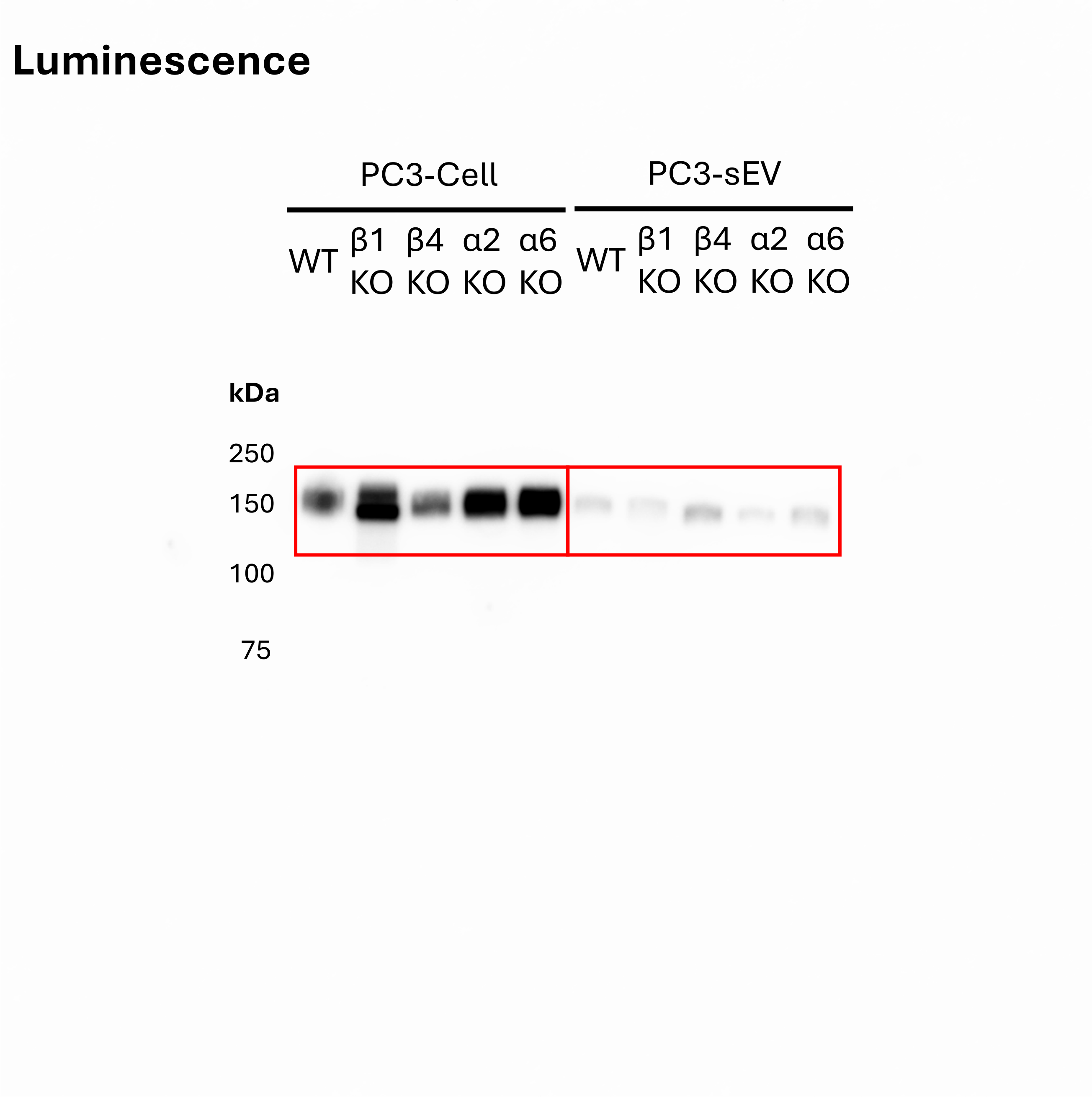

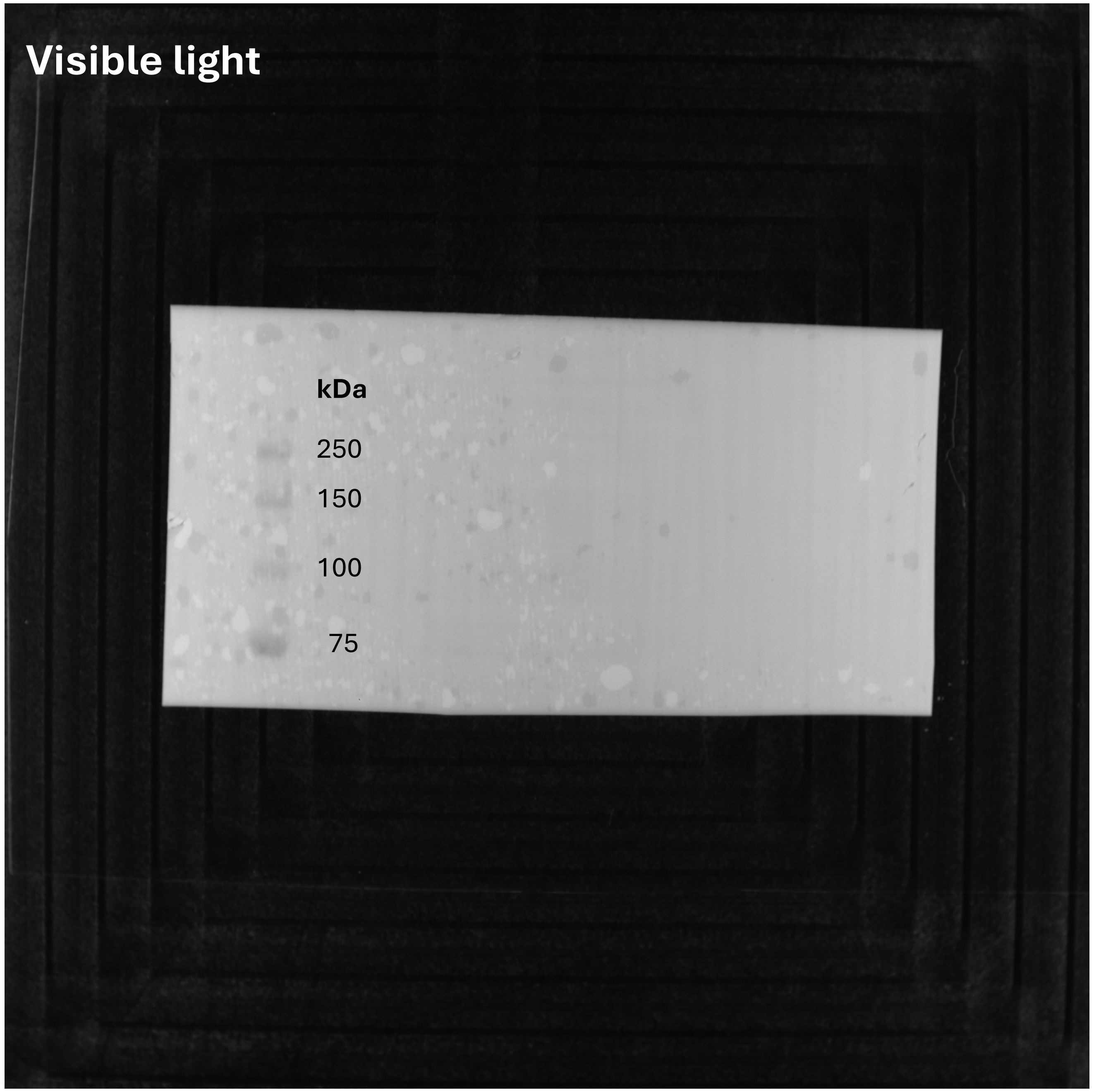

SourceDataF1A-Integrin αV

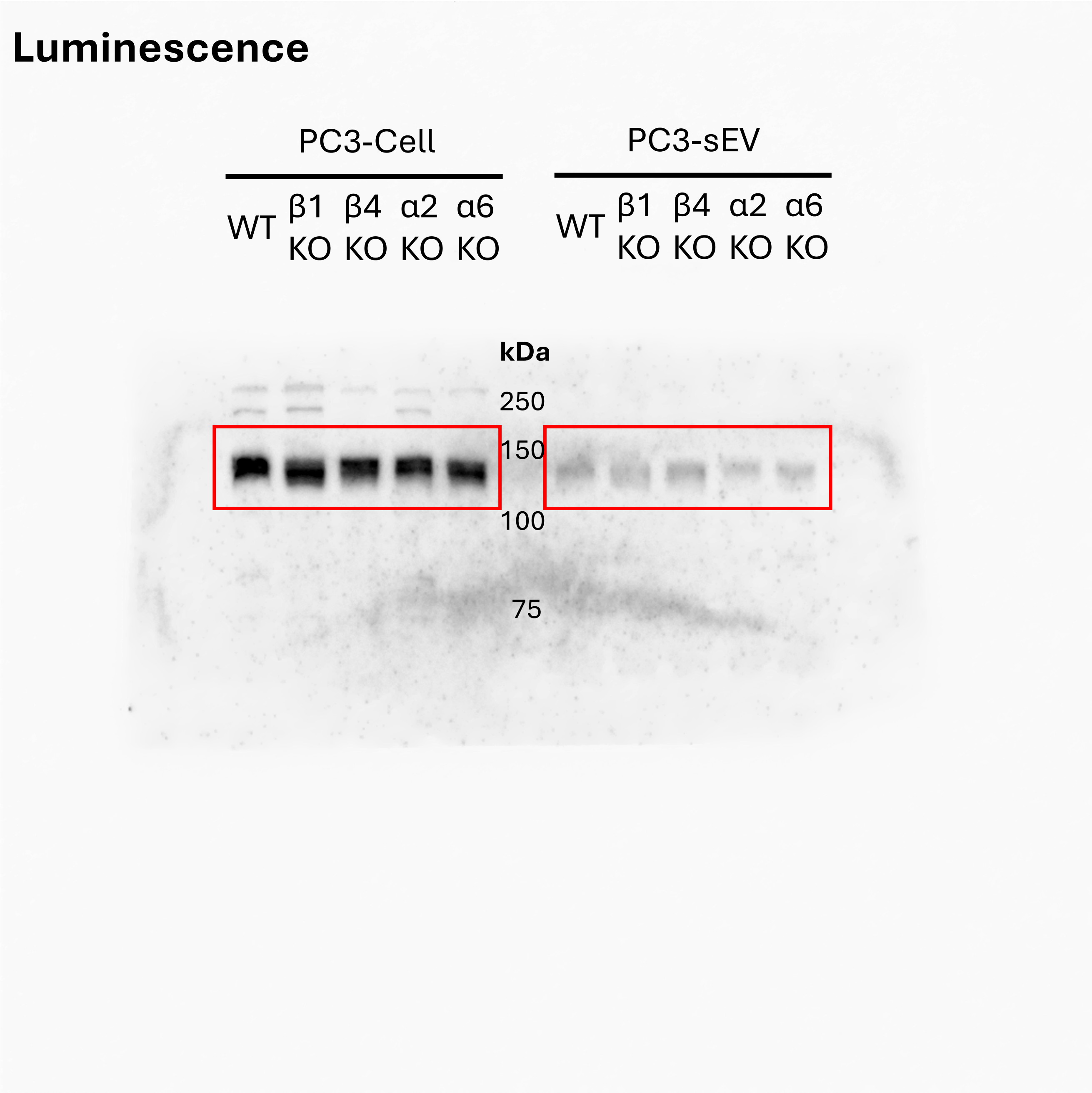

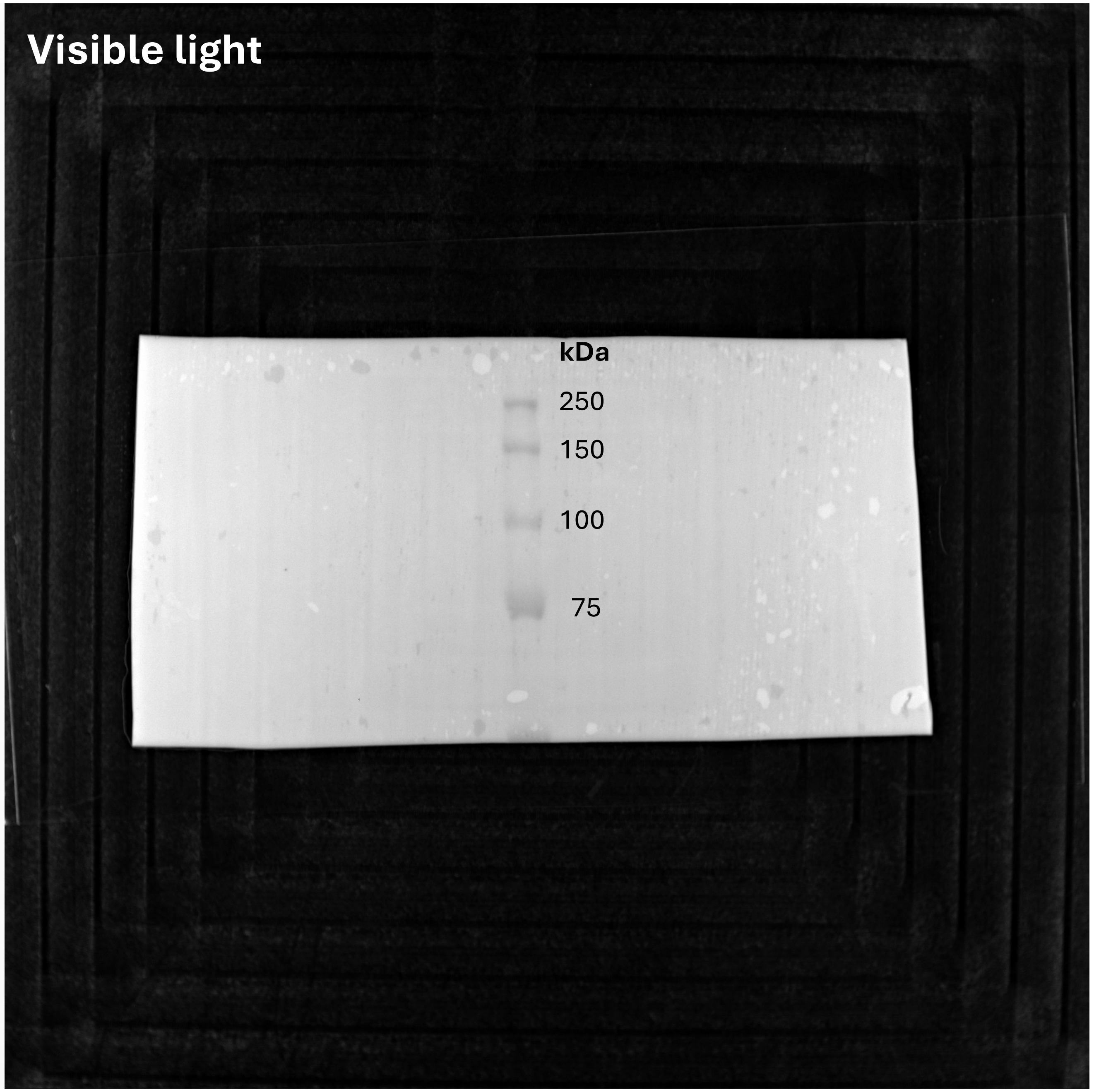

SourceDataF1A-CD63

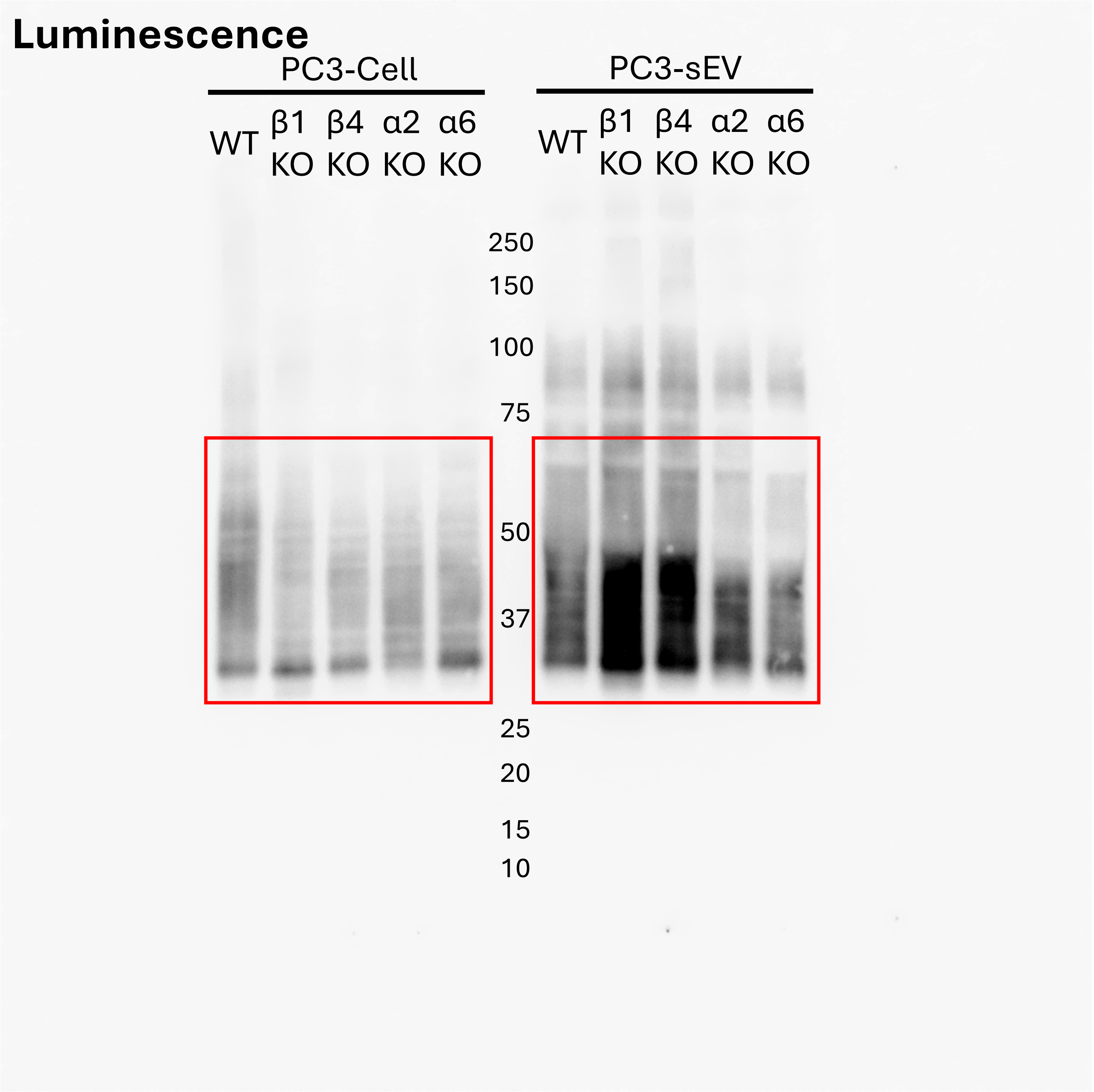

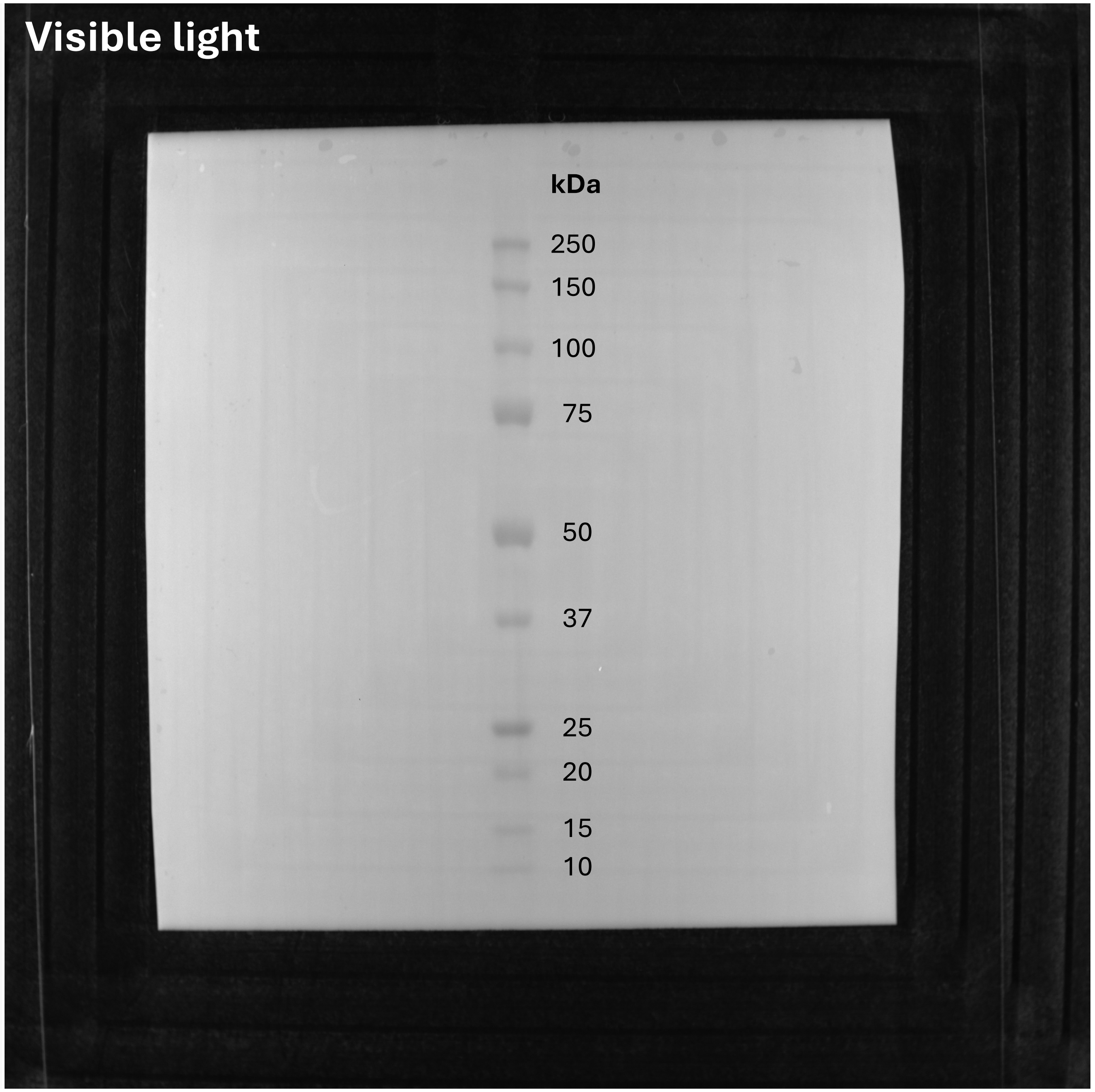

SourceDataF1A-CD81

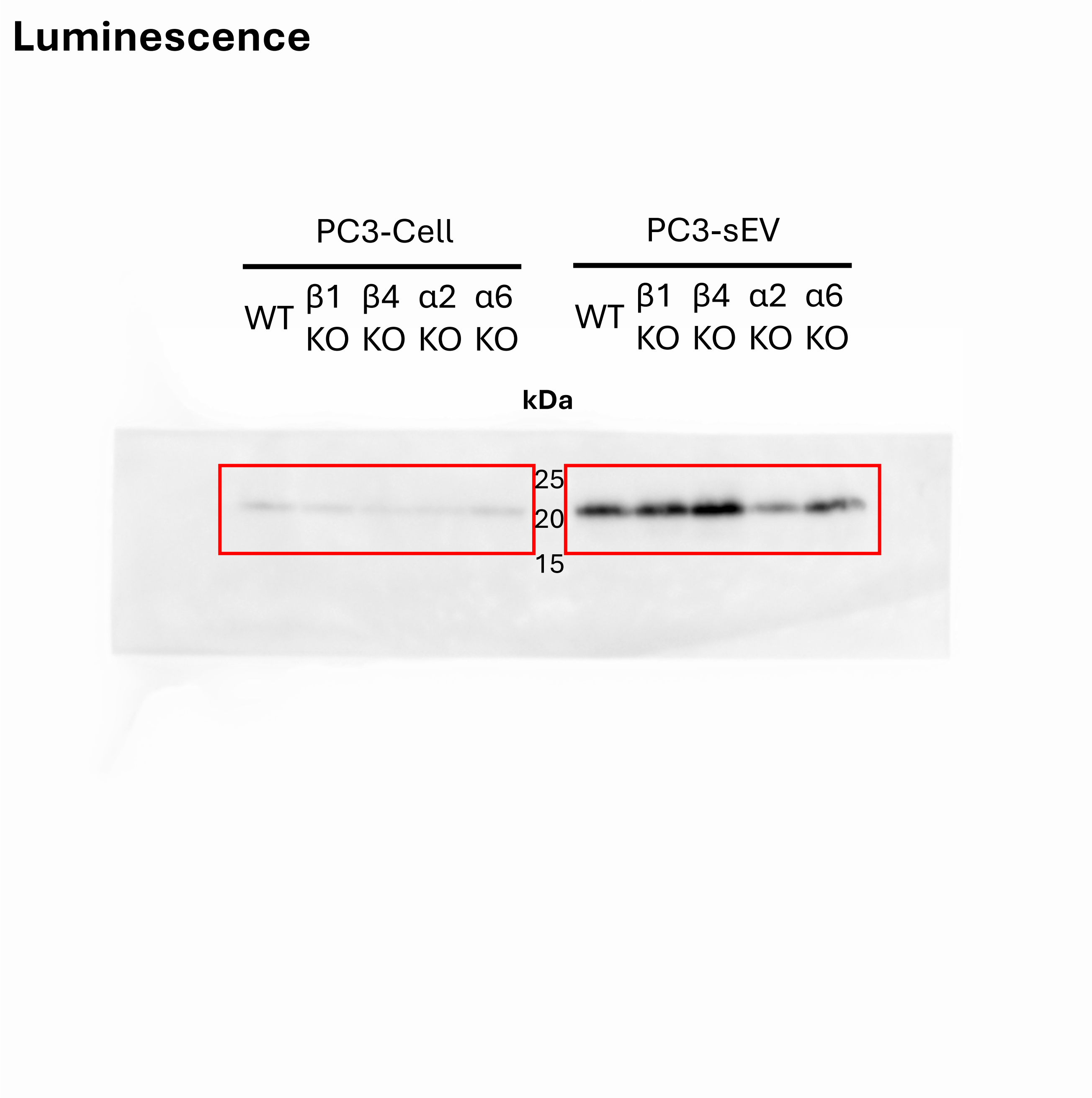

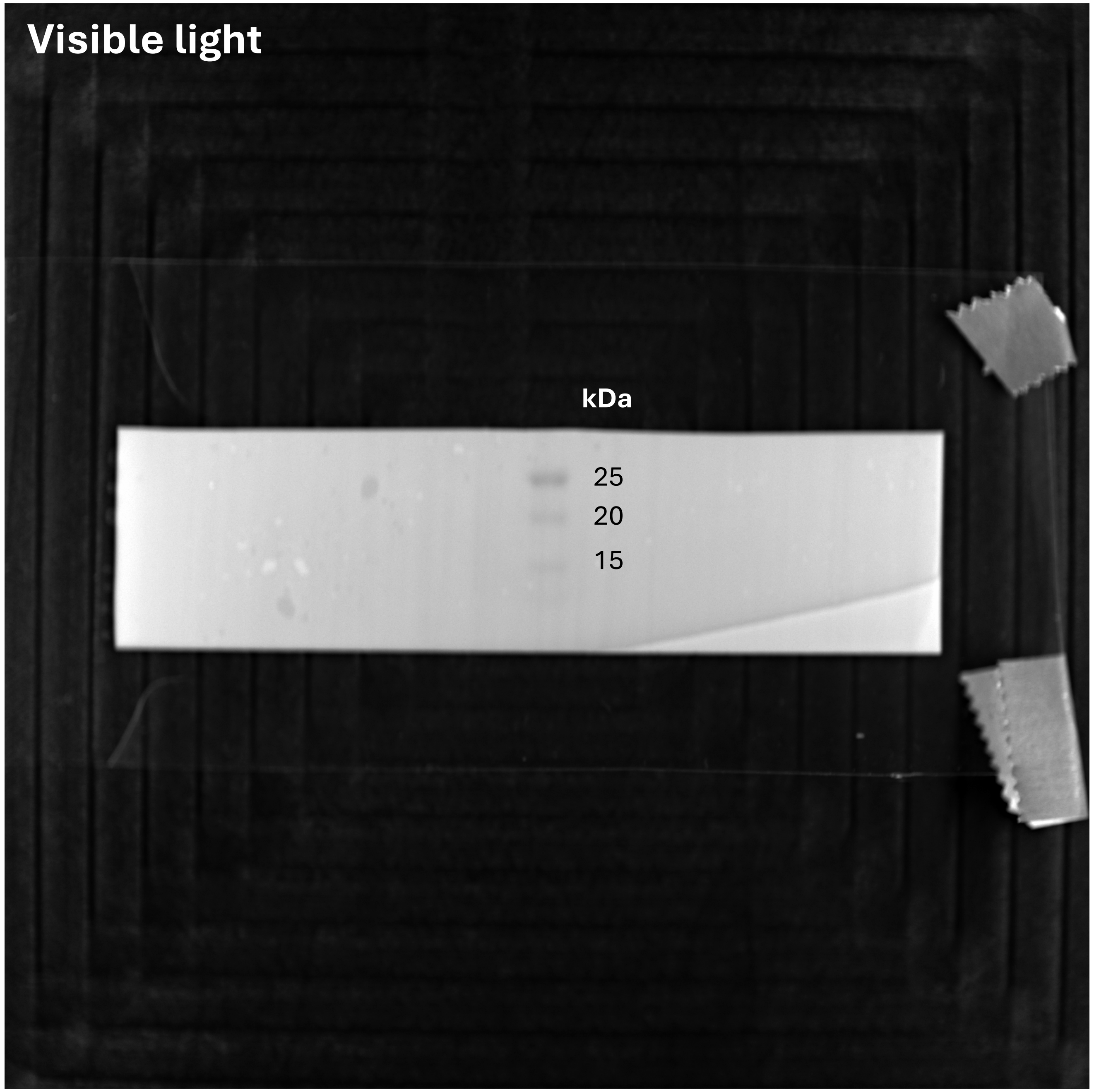

SourceDataF1A-CD9

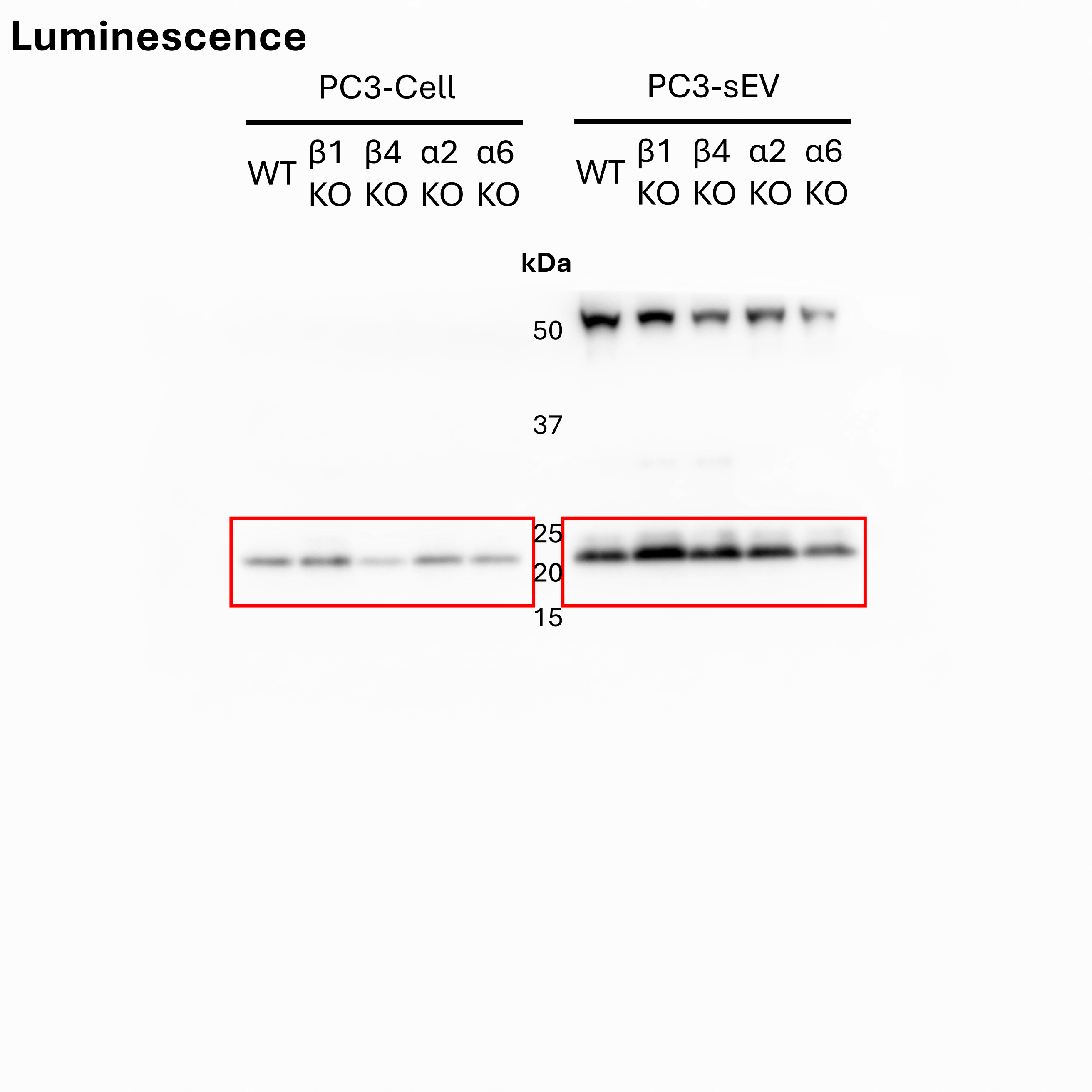

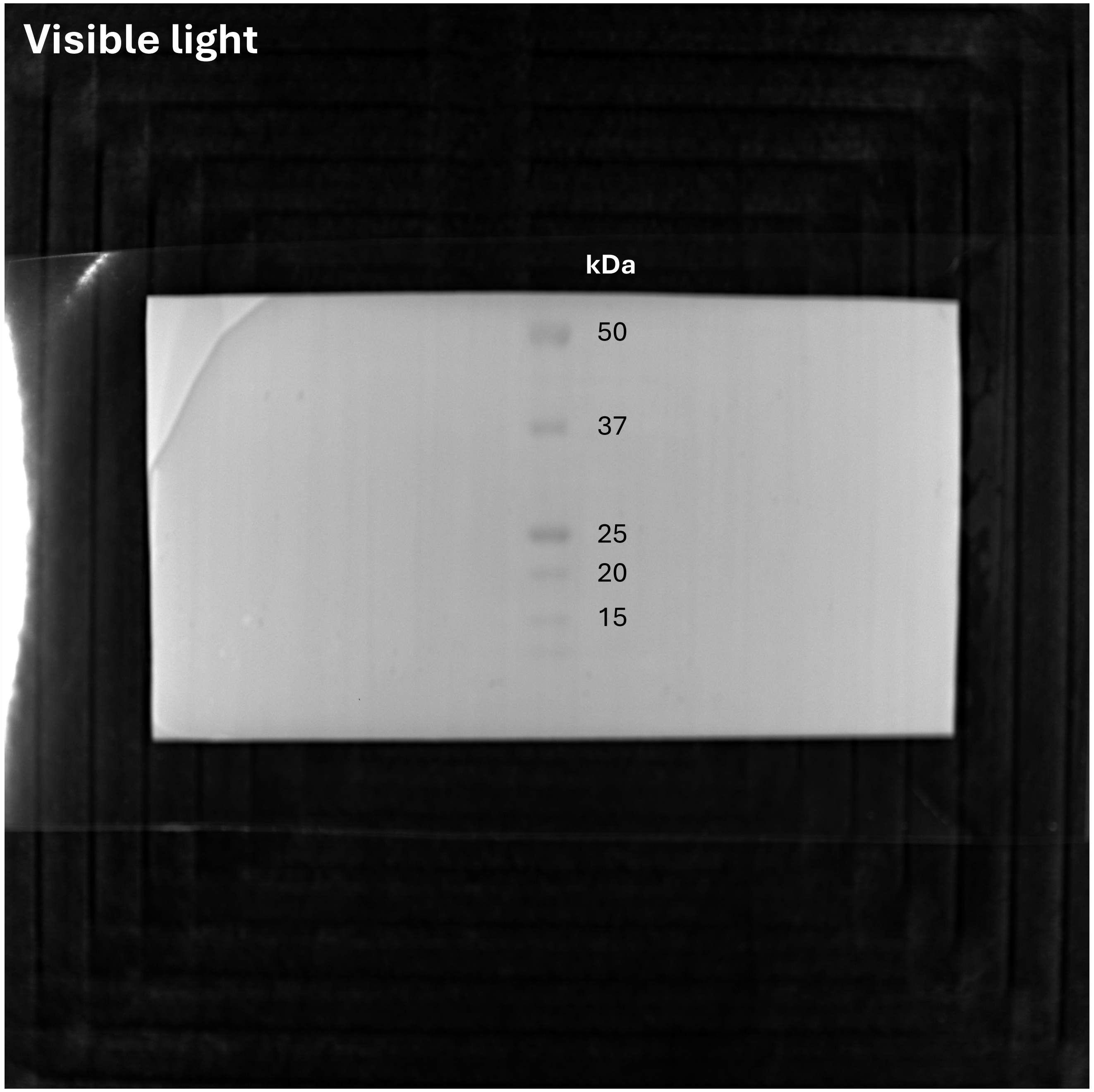

SourceDataF1A-β-actin

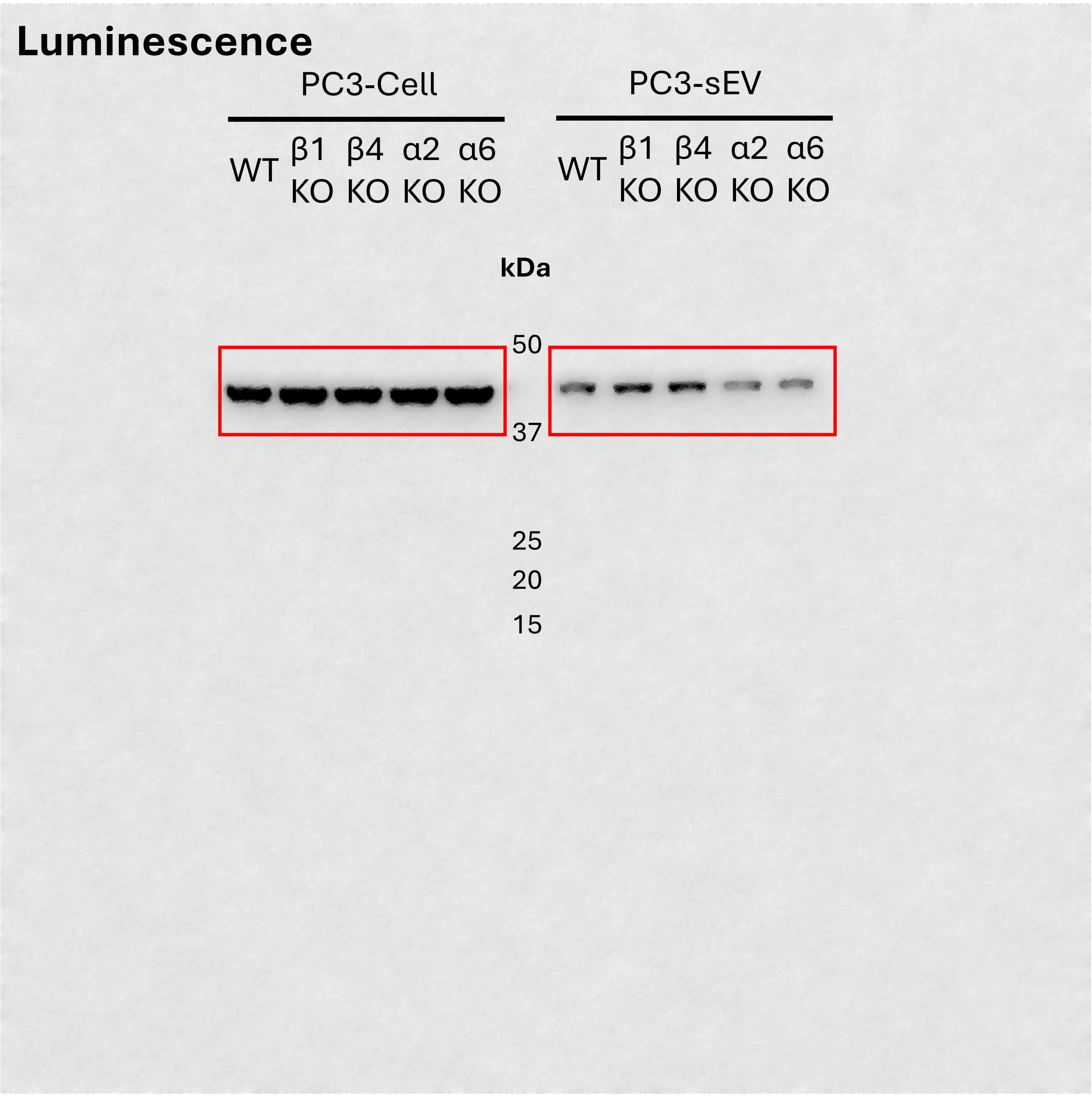

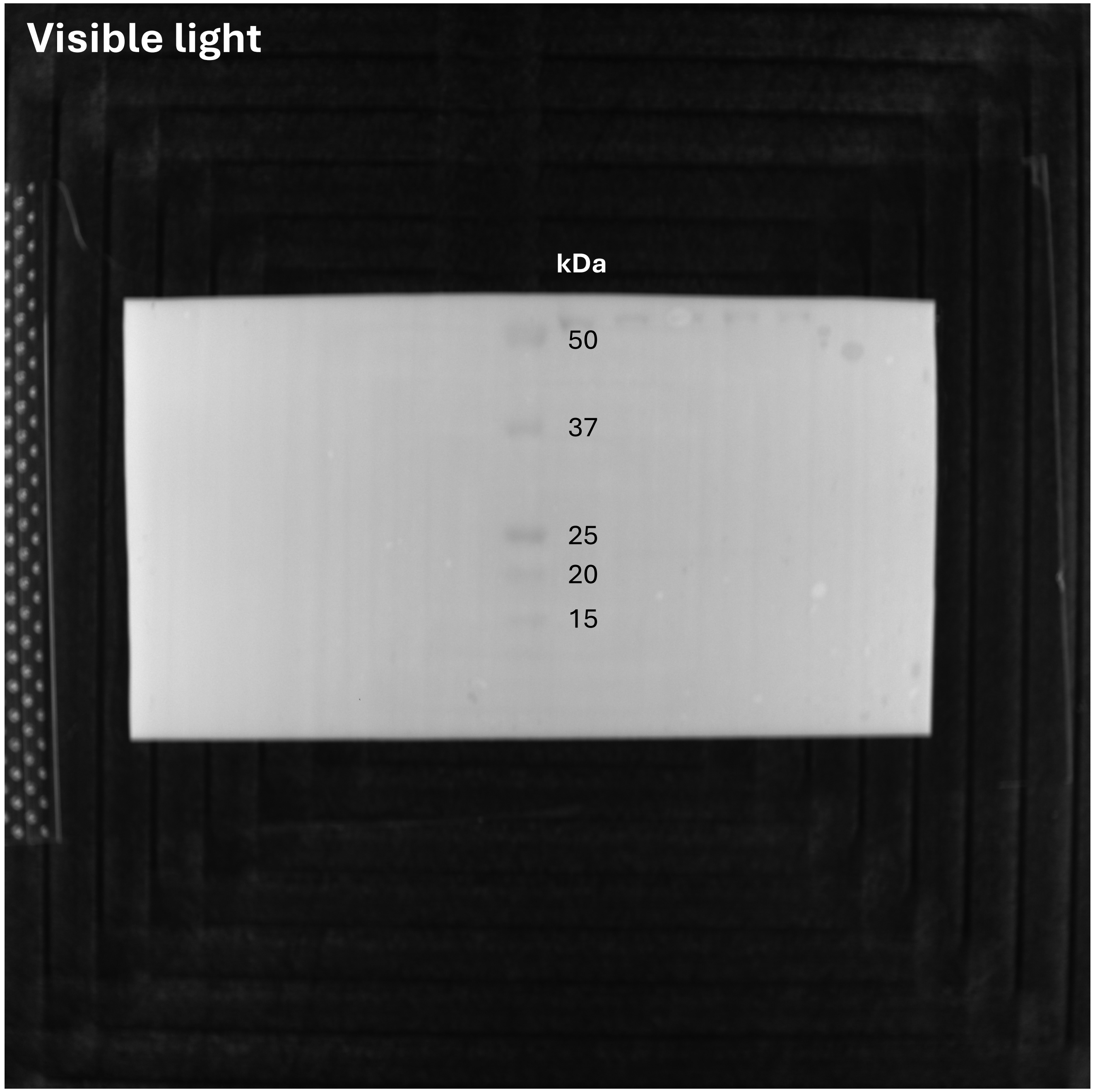

Supplement: SourceData F1 — is the source file for Fig. 1. [file jcb_202404064_sourcedataf1.pdf]
