# Supplementary material for: Extracellular vesicles adhere to cells primarily by interactions of integrins and GM1 with laminin
Source: J Cell Biol. 2025 Apr 30;224(6):e202404064. doi: 10.1083/jcb.202404064 (PMC12042775; doi:10.1083/jcb.202404064)

Fig. 3F

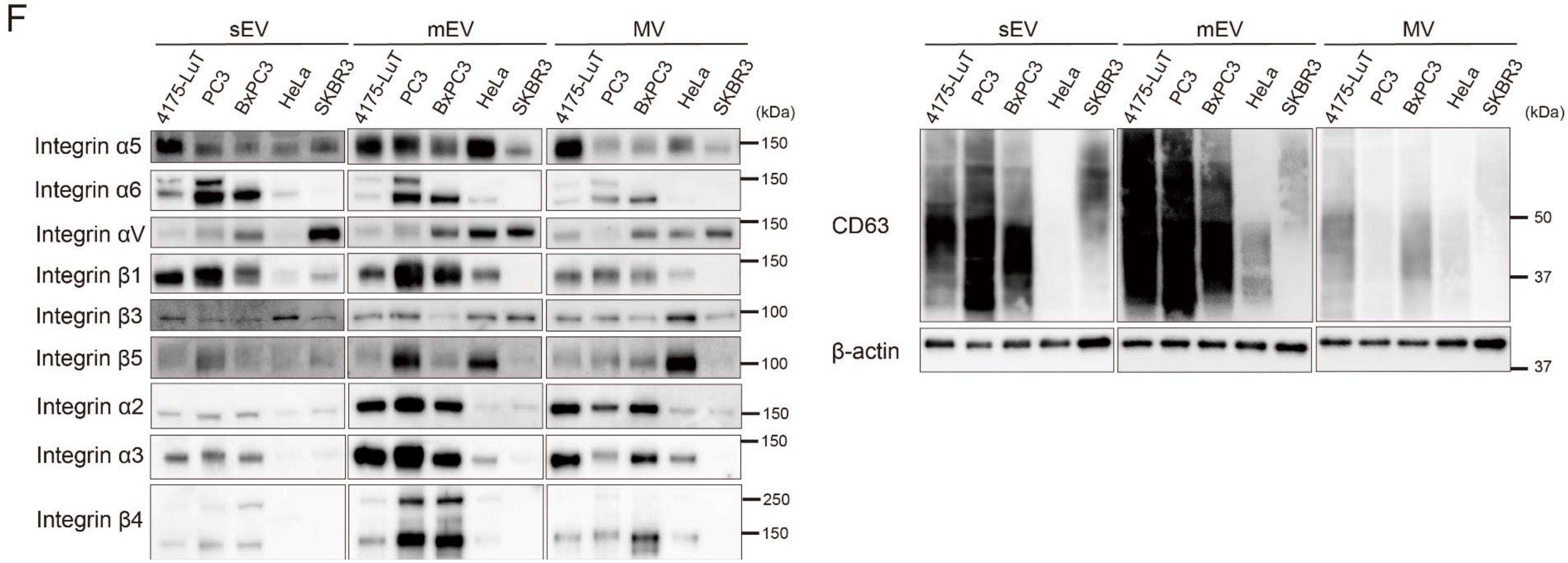

F
sEV
mEV
MV
4175-LuT
PC3
BxPC3
HeLa
SKBR3
(kDa)
Integrin α5
Integrin α6
Integrin αV
Integrin β1
Integrin β3
Integrin β5
Integrin α2
Integrin α3
Integrin β4
150
100
250
CD63
β-actin
50
37

SourceDataF3F_sEV-Integrin α5

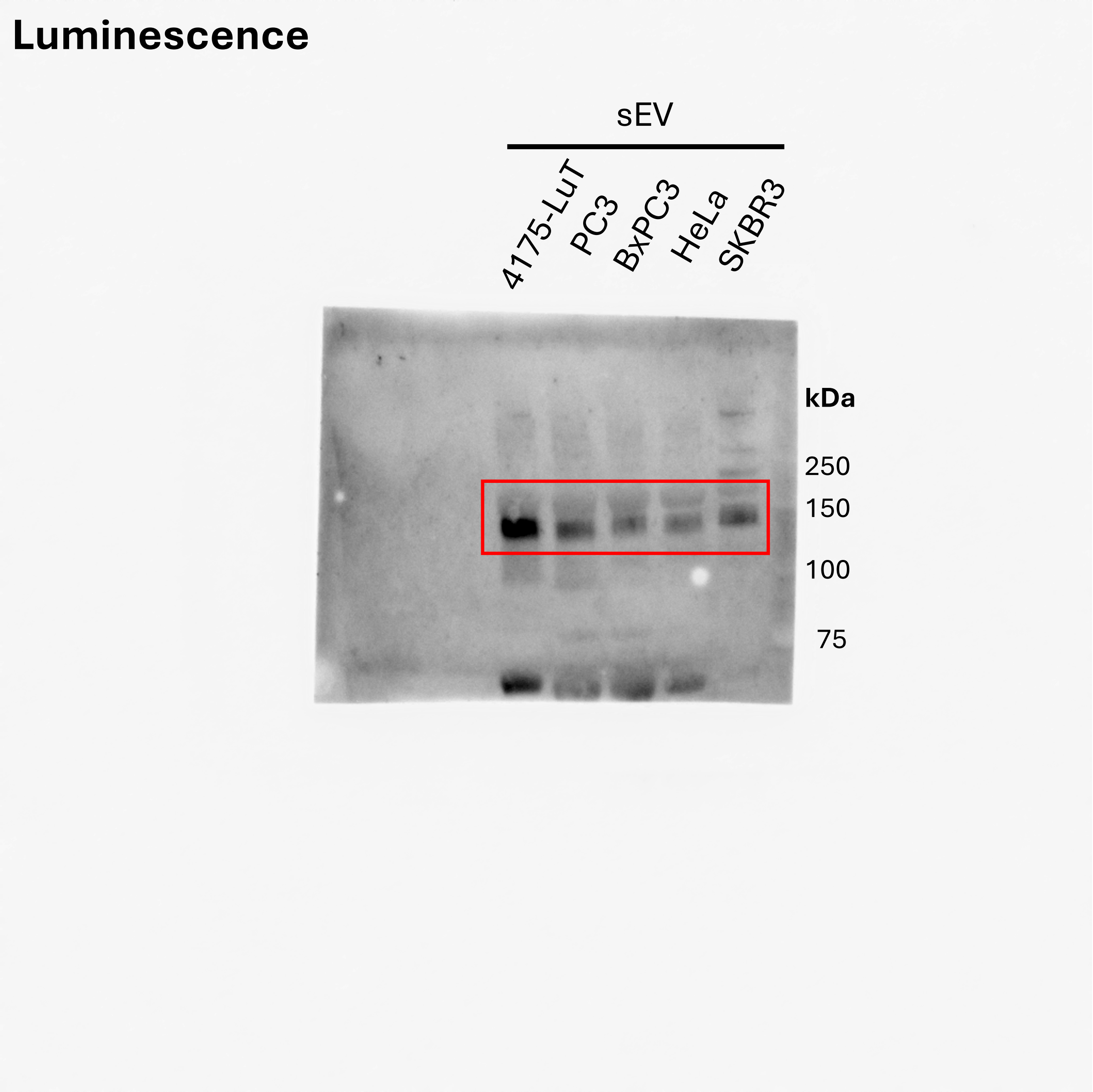

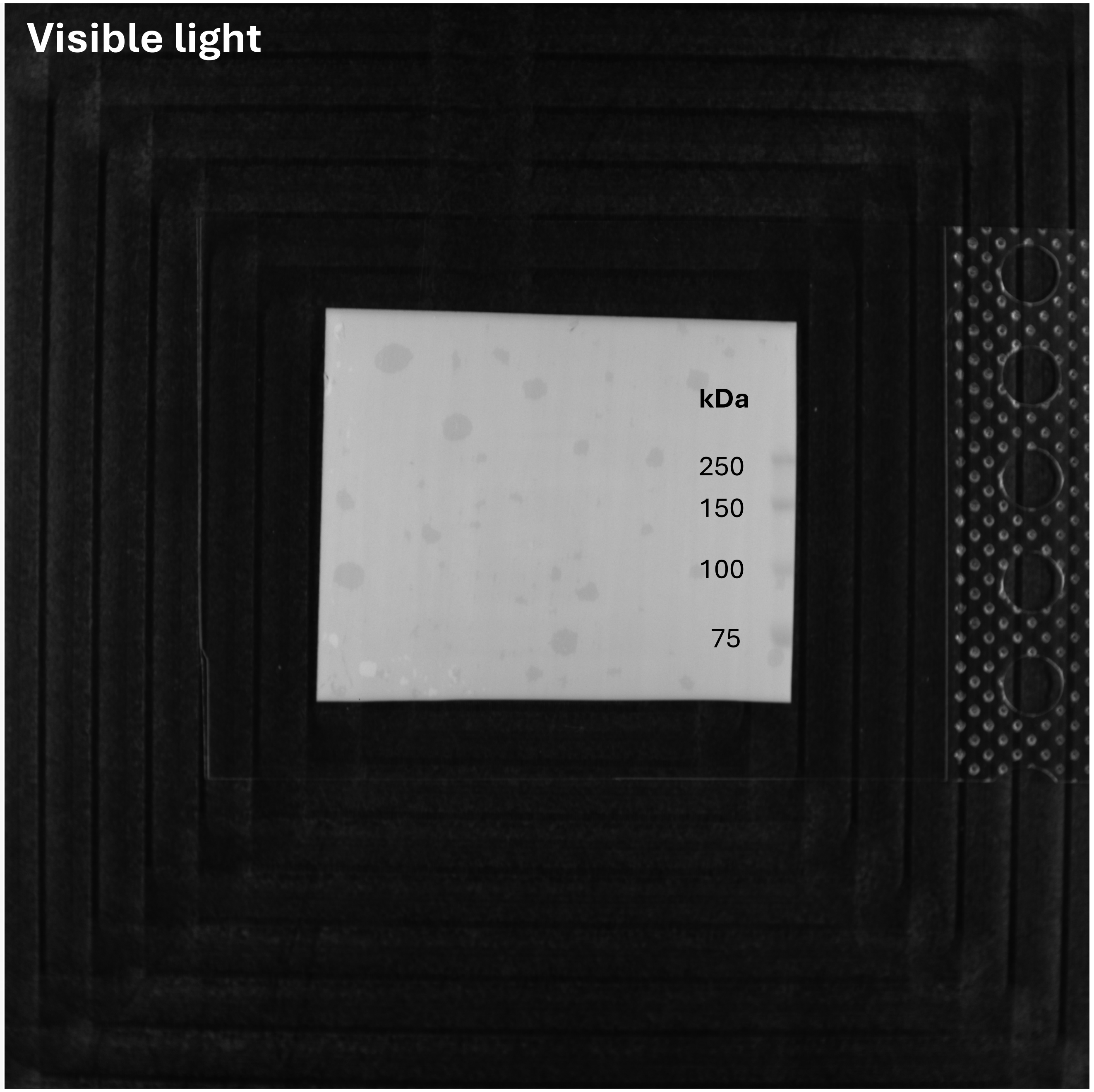

SourceDataF3F_mEV/MV-Integrin α5

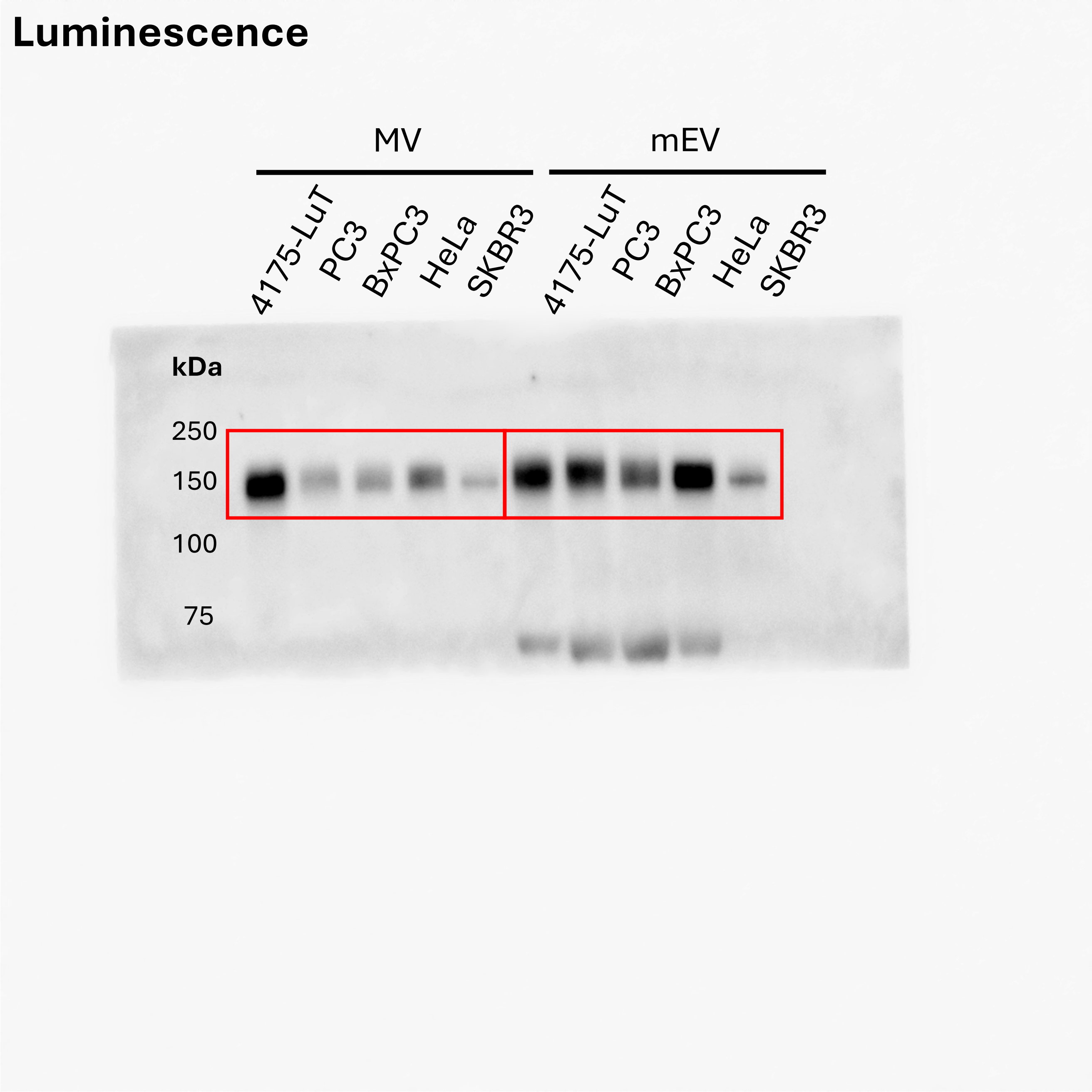

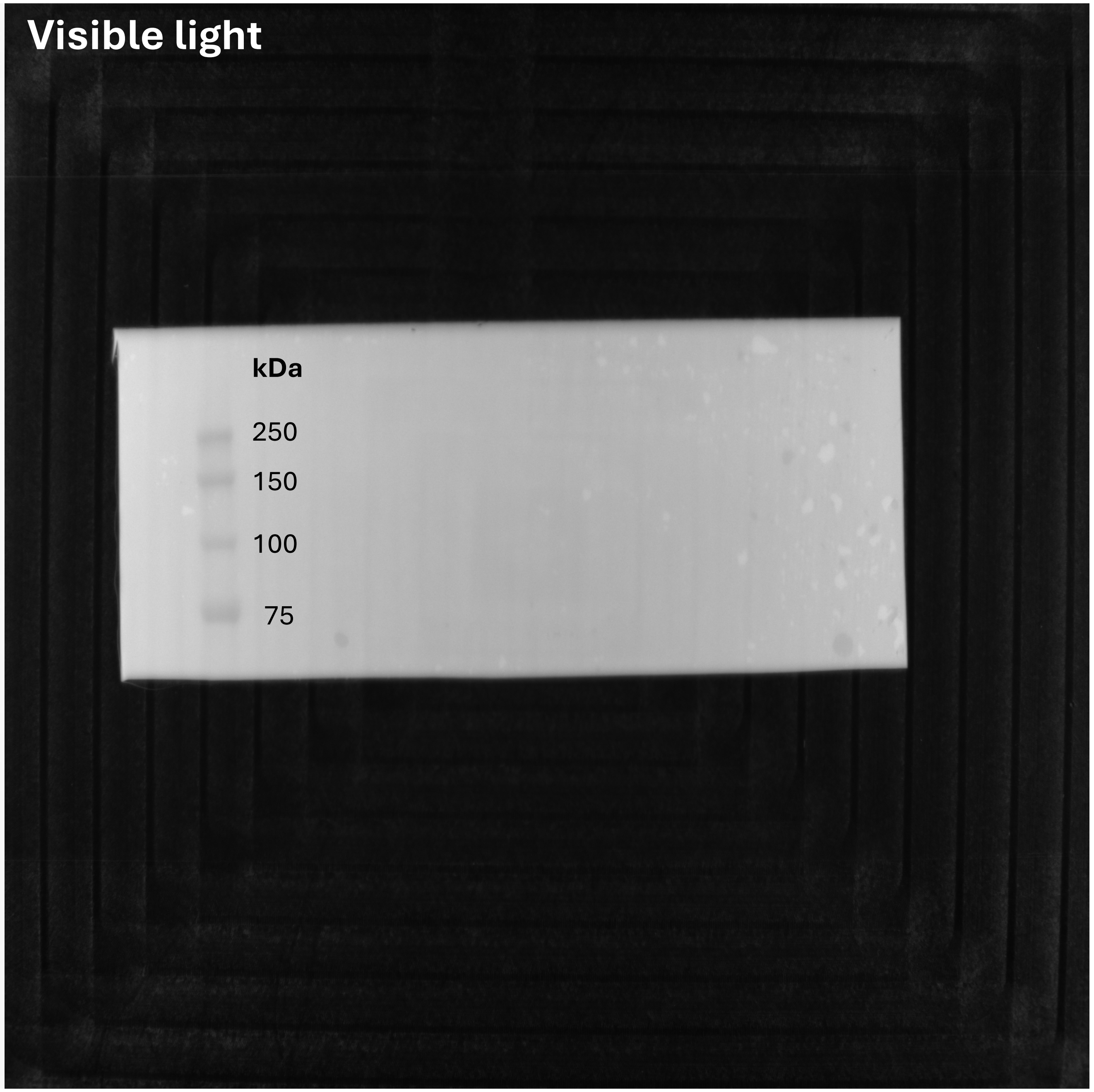

SourceDataF3F_sEV-Integrin α6

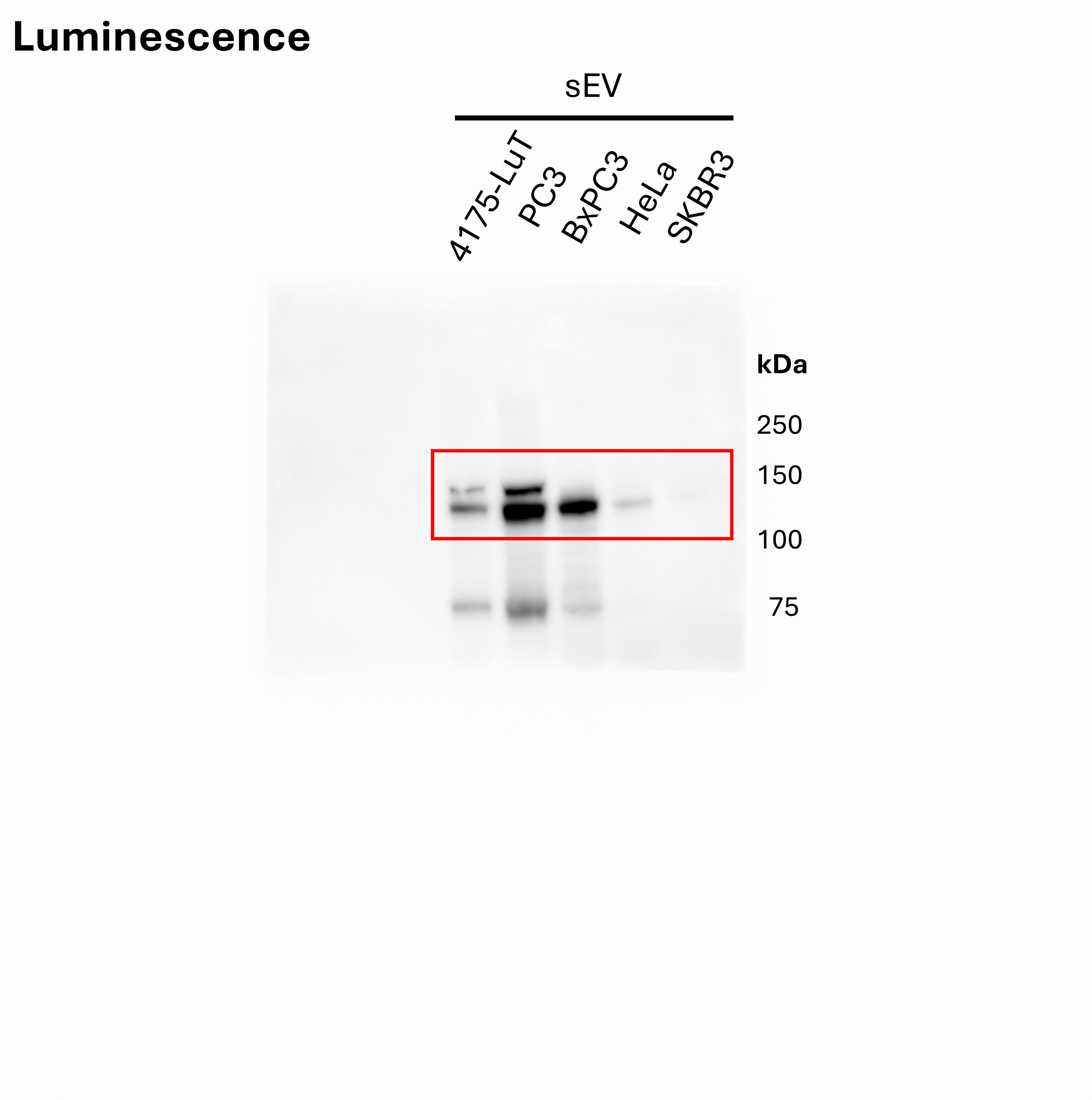

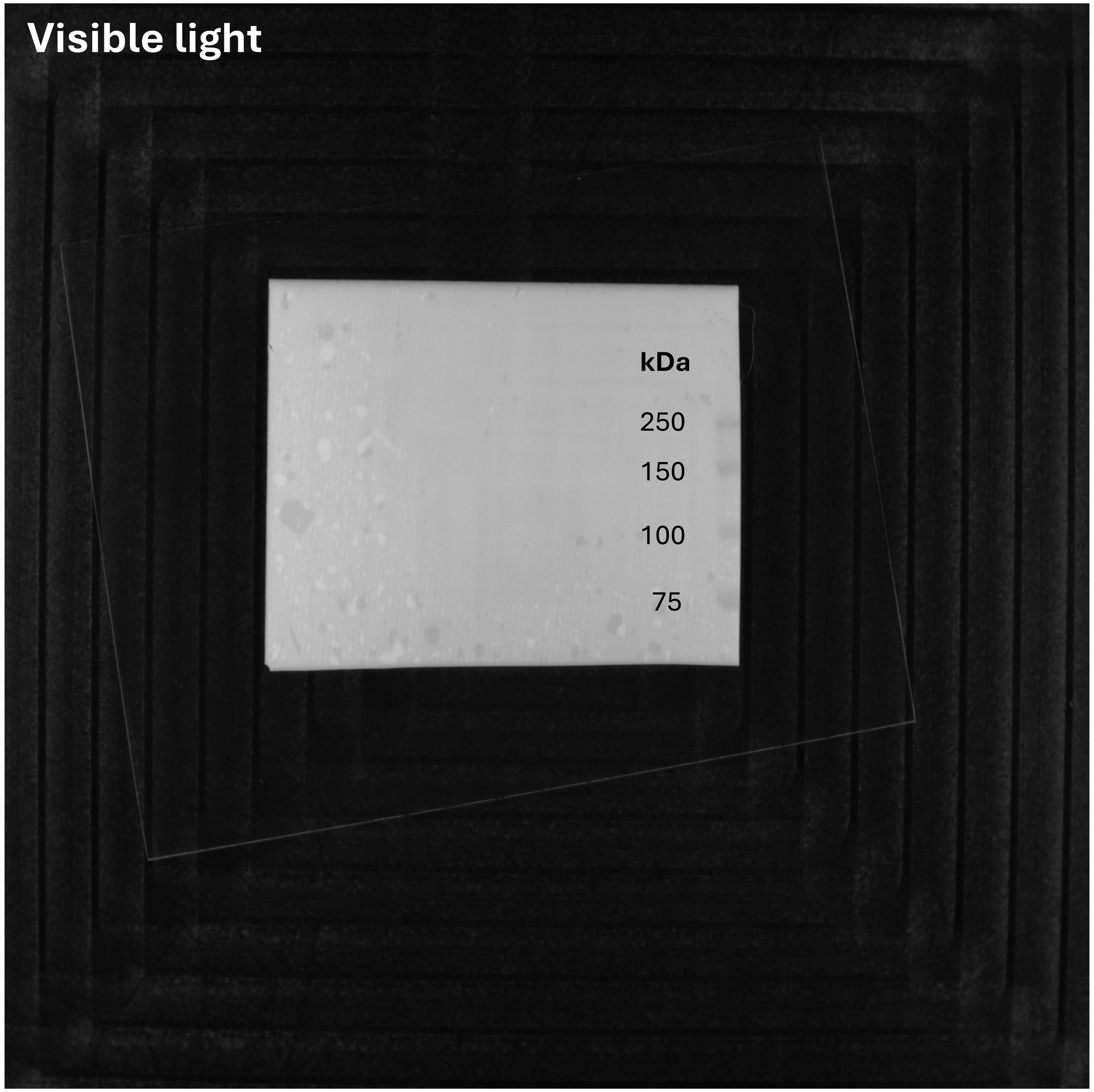

SourceDataF3F_mEV/MV-Integrin α6

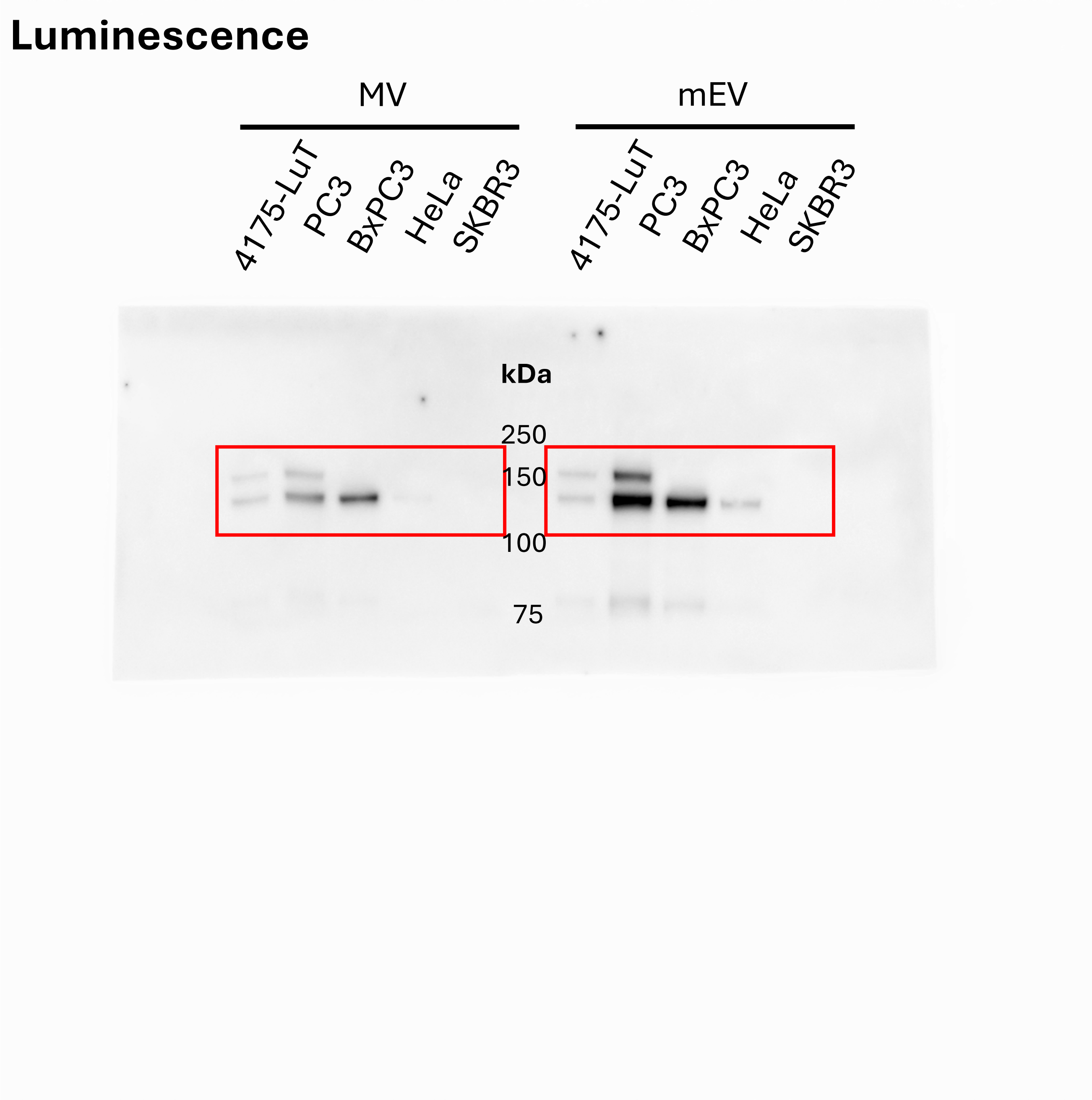

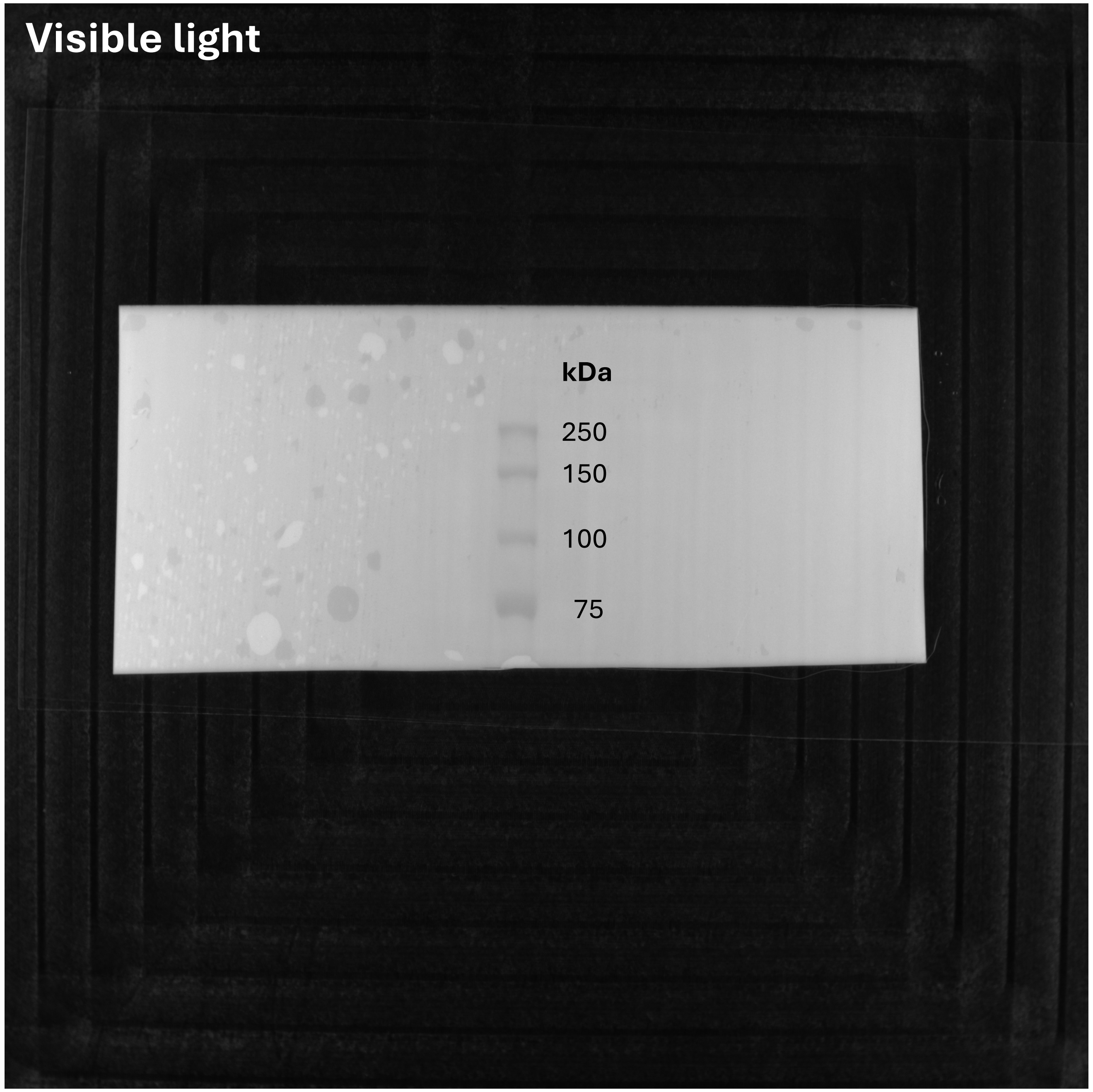

SourceDataF3F_sEV-Integrin αV

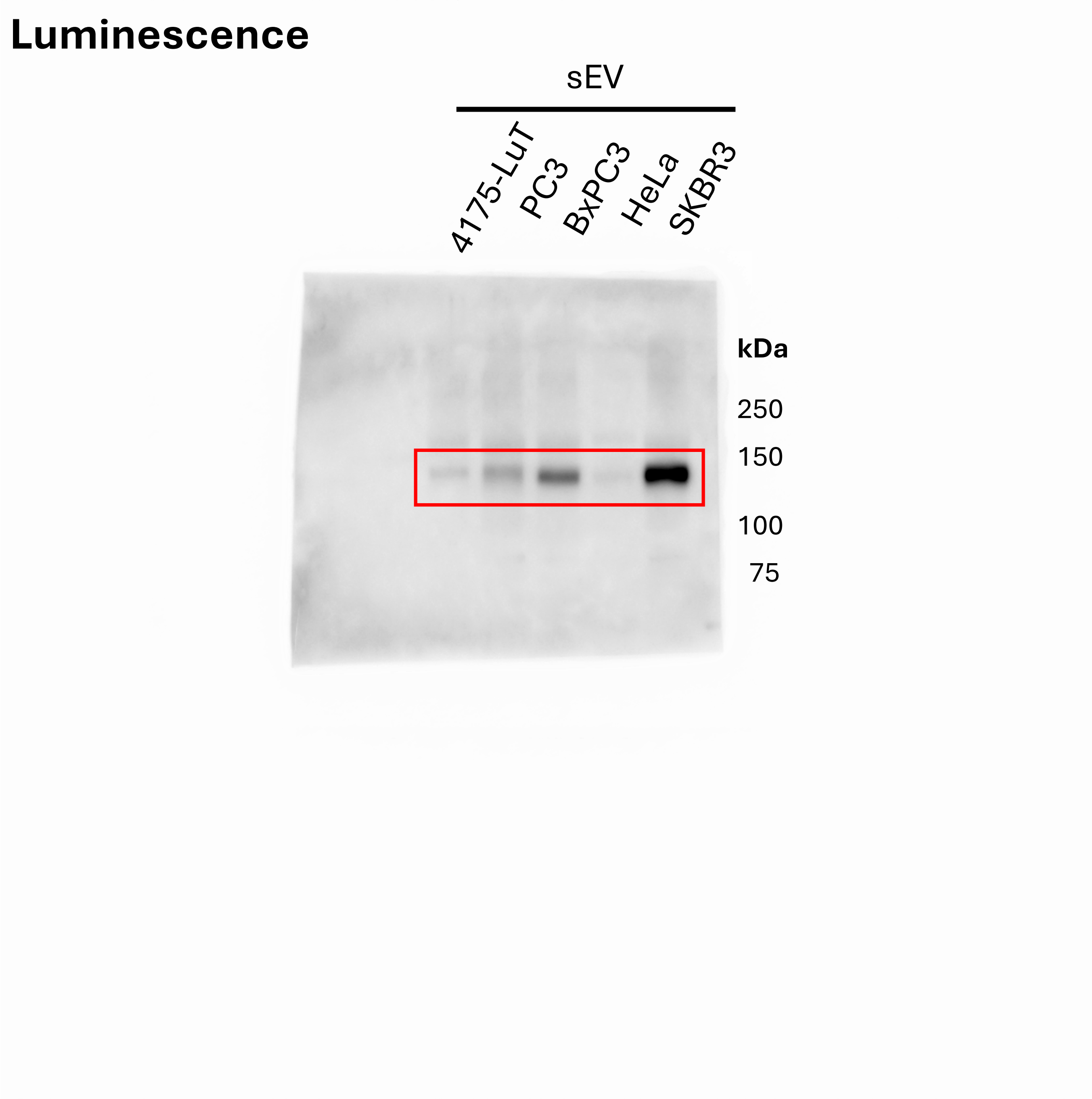

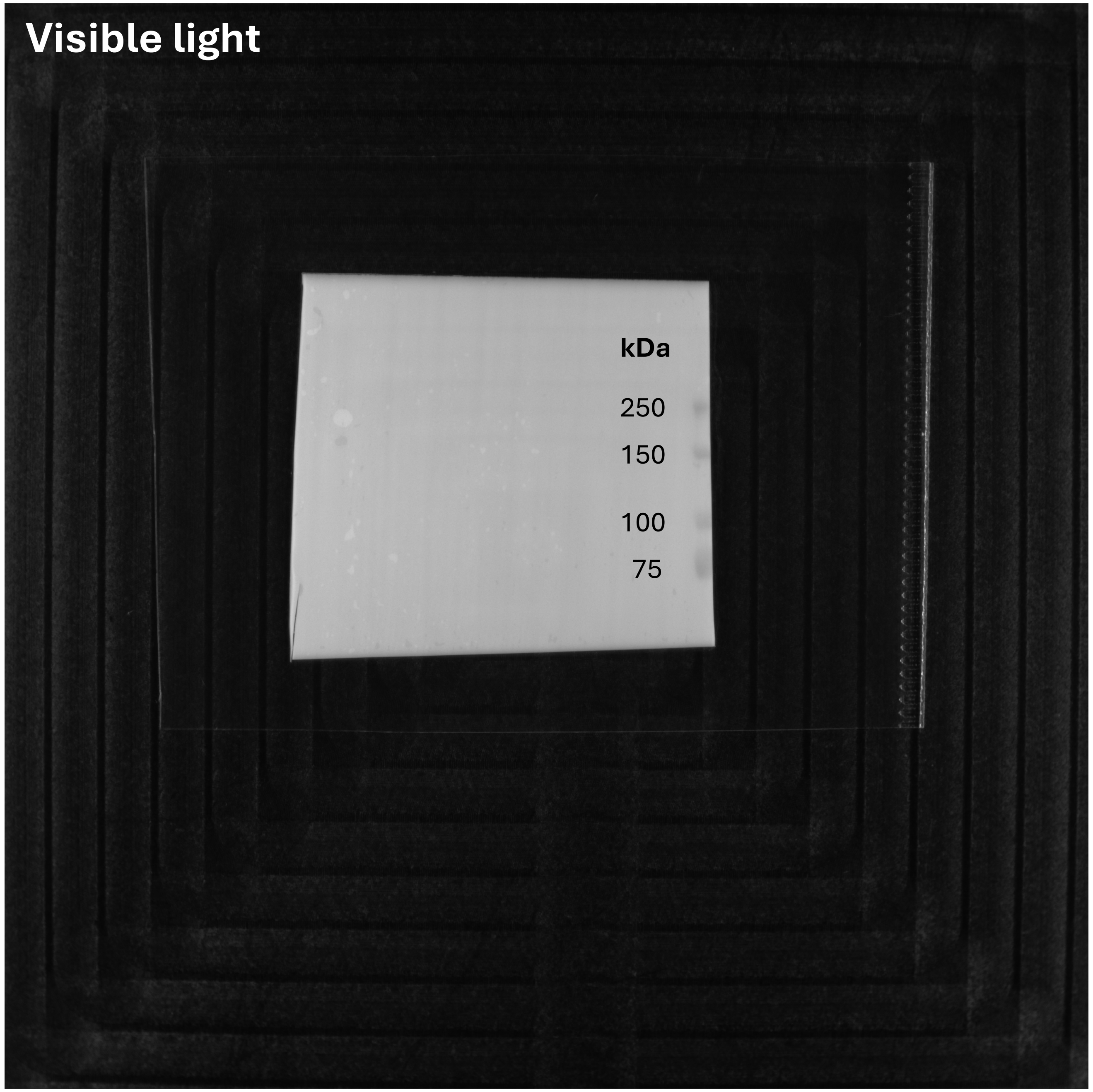

SourceDataF3F_mEV/MV-Integrin αV

Luminescence

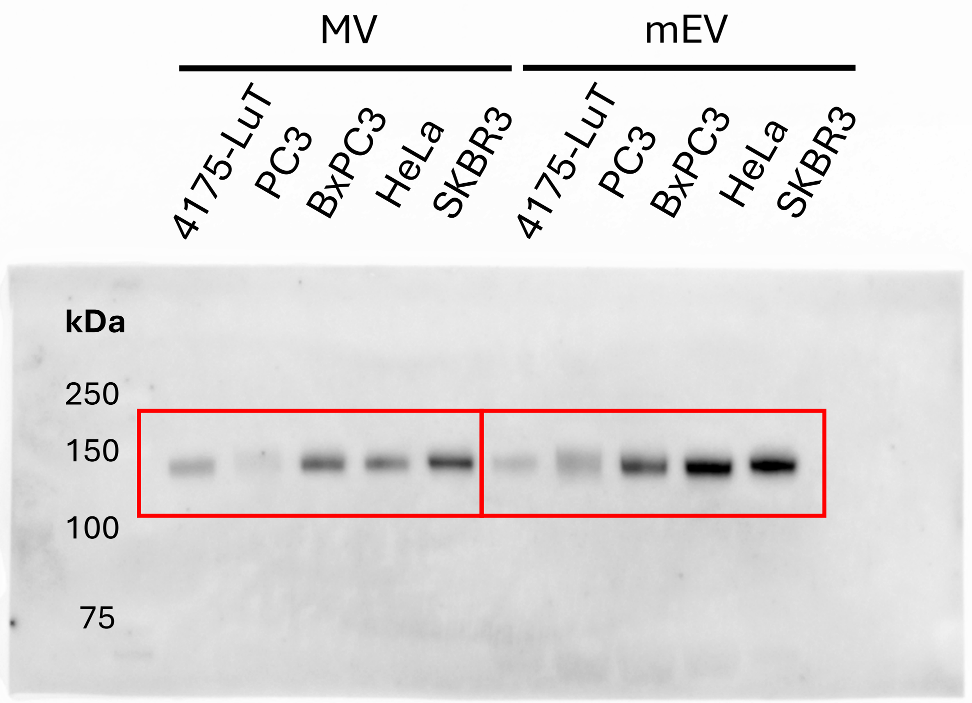

Visible light

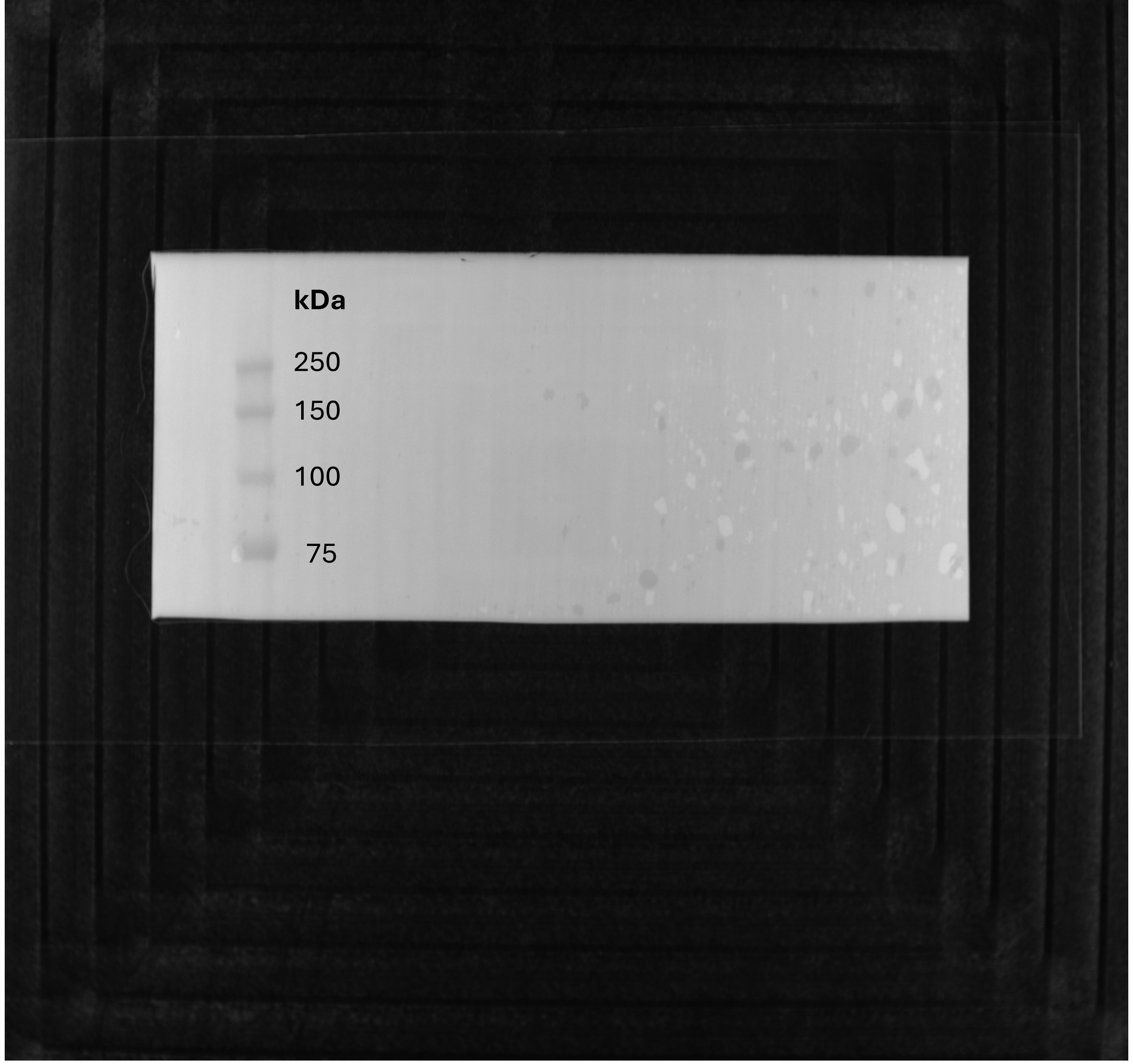

SourceDataF3F_sEV-Integrin β1

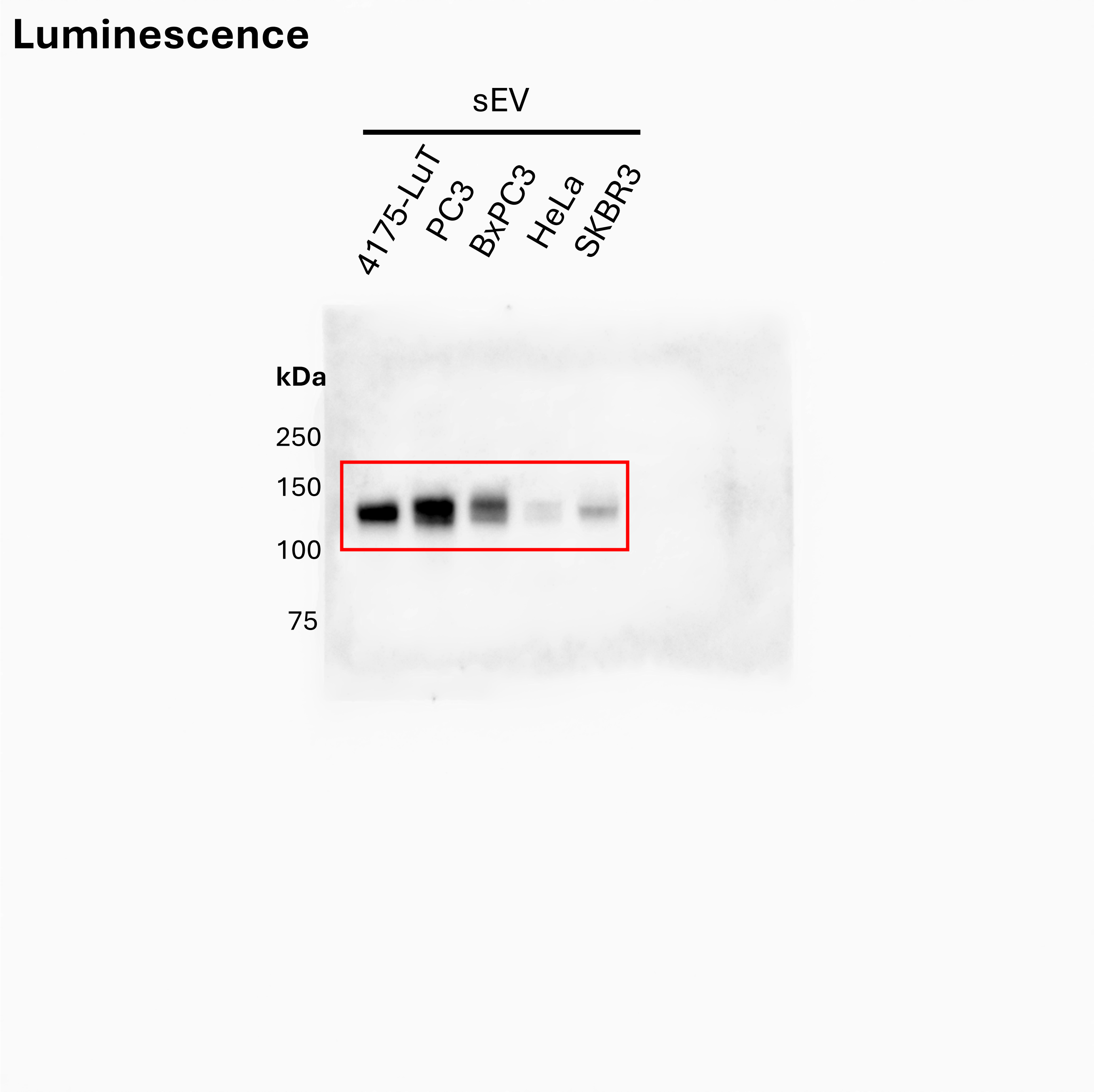

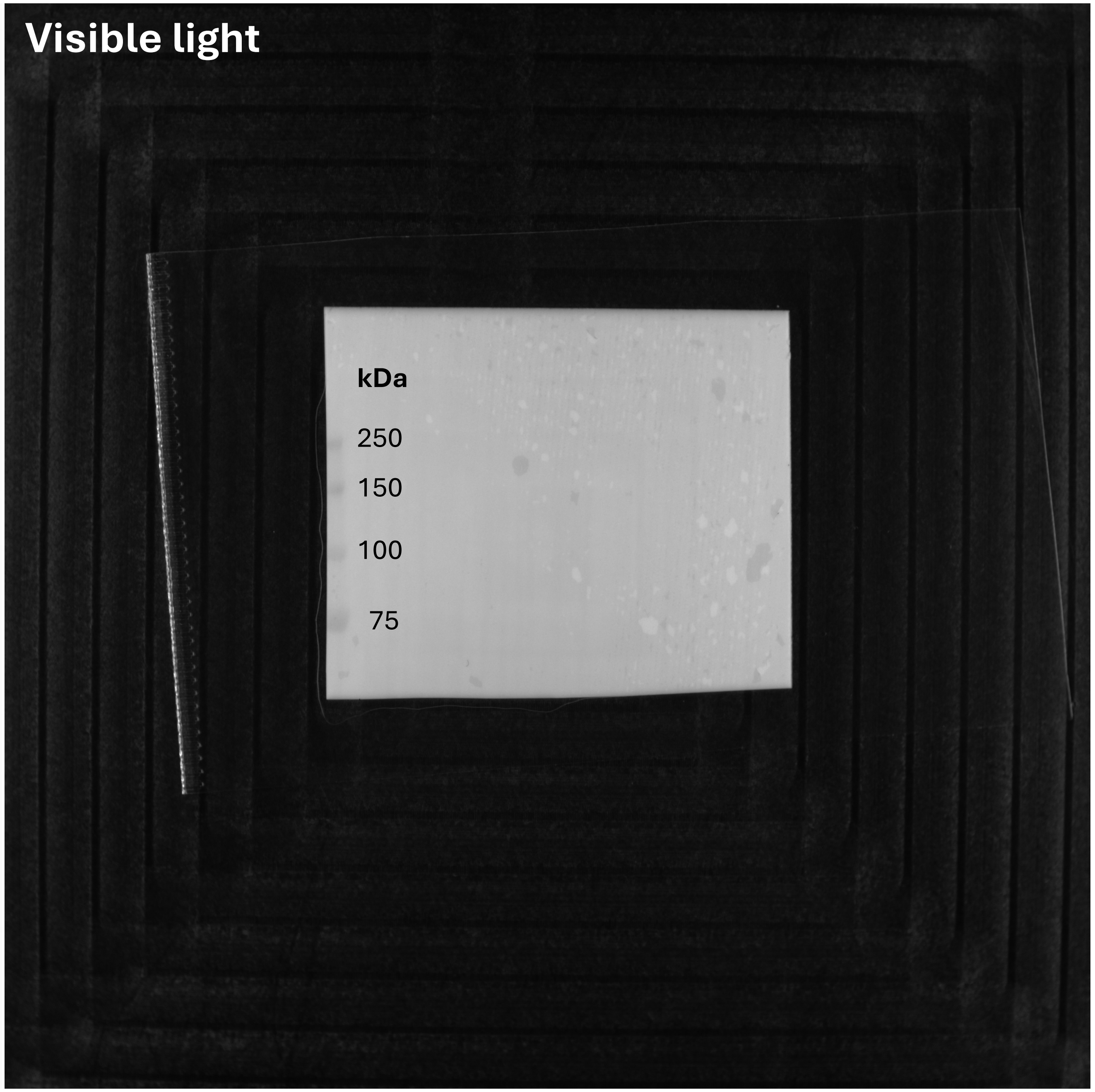

SourceDataF3F_mEV/MV-Integrin β1

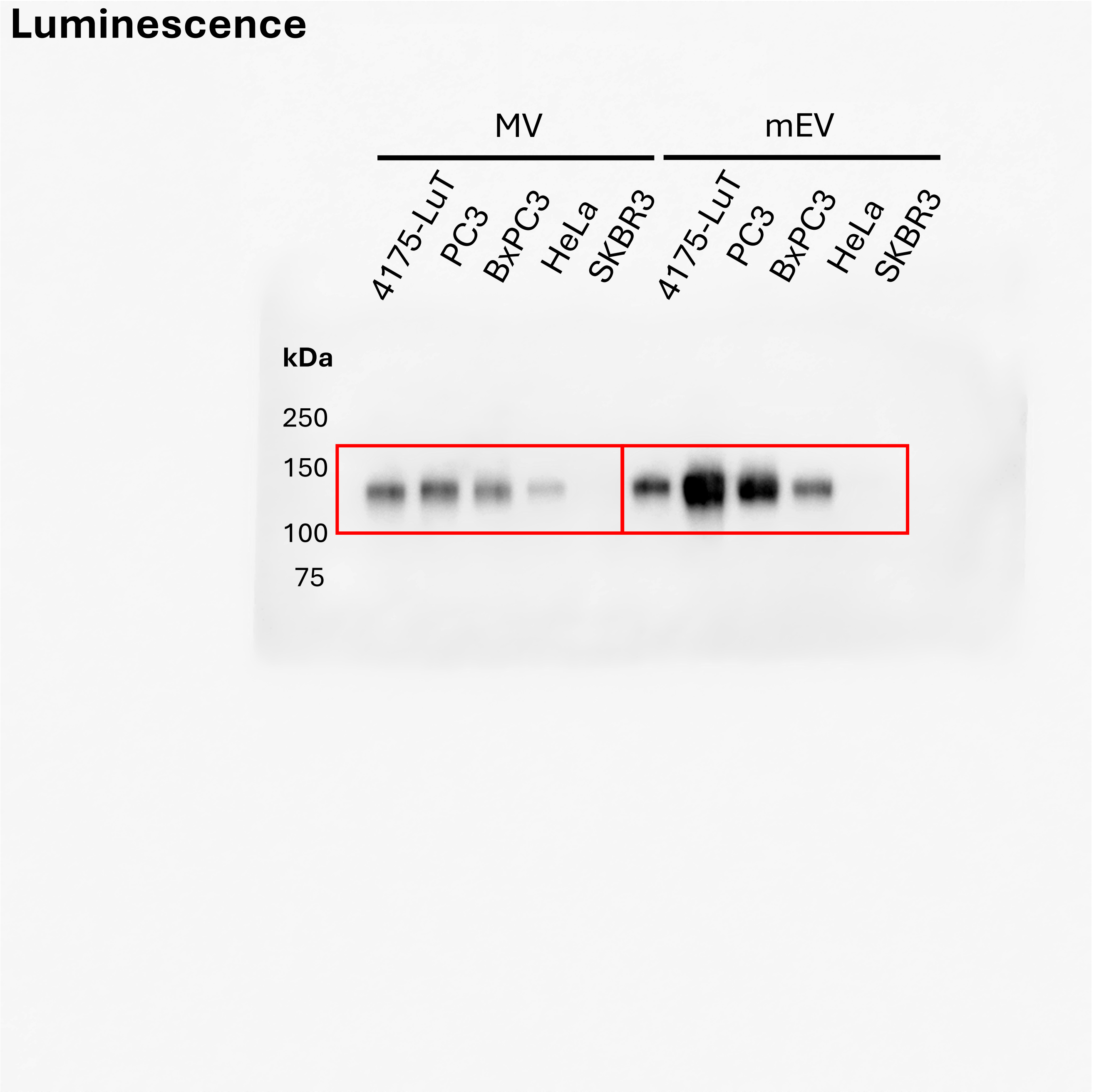

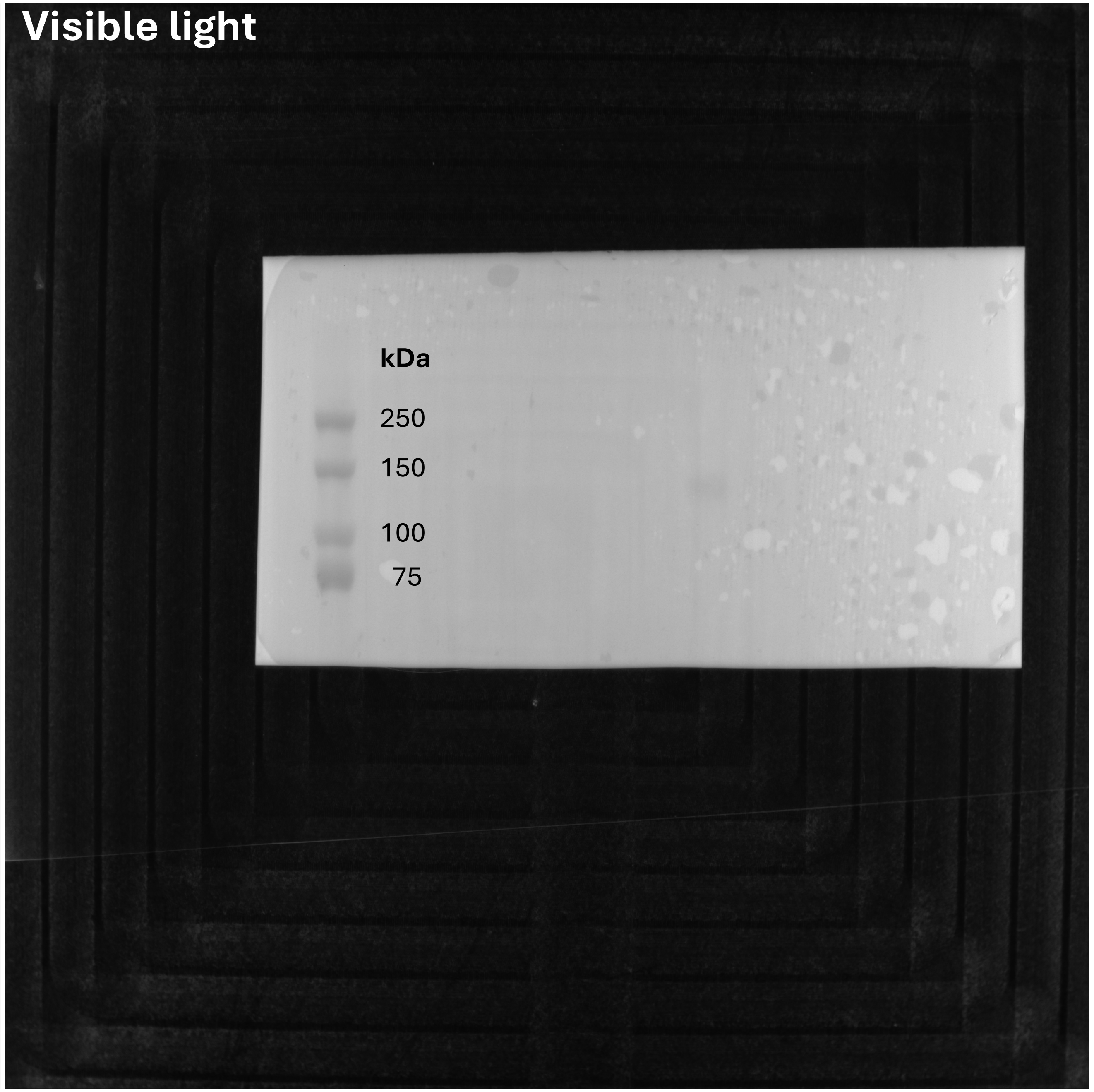

SourceDataF3F_sEV-Integrin β3

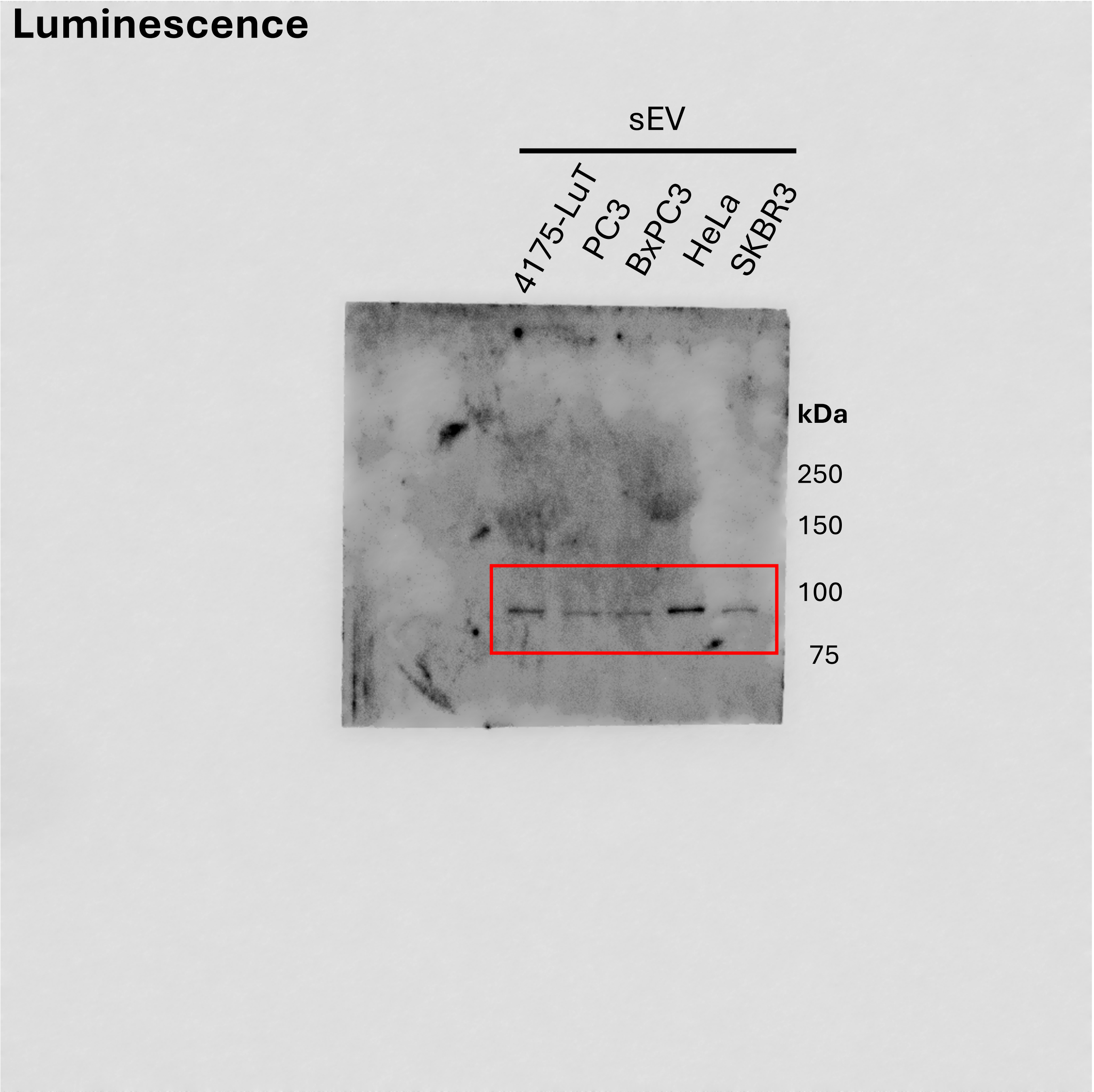

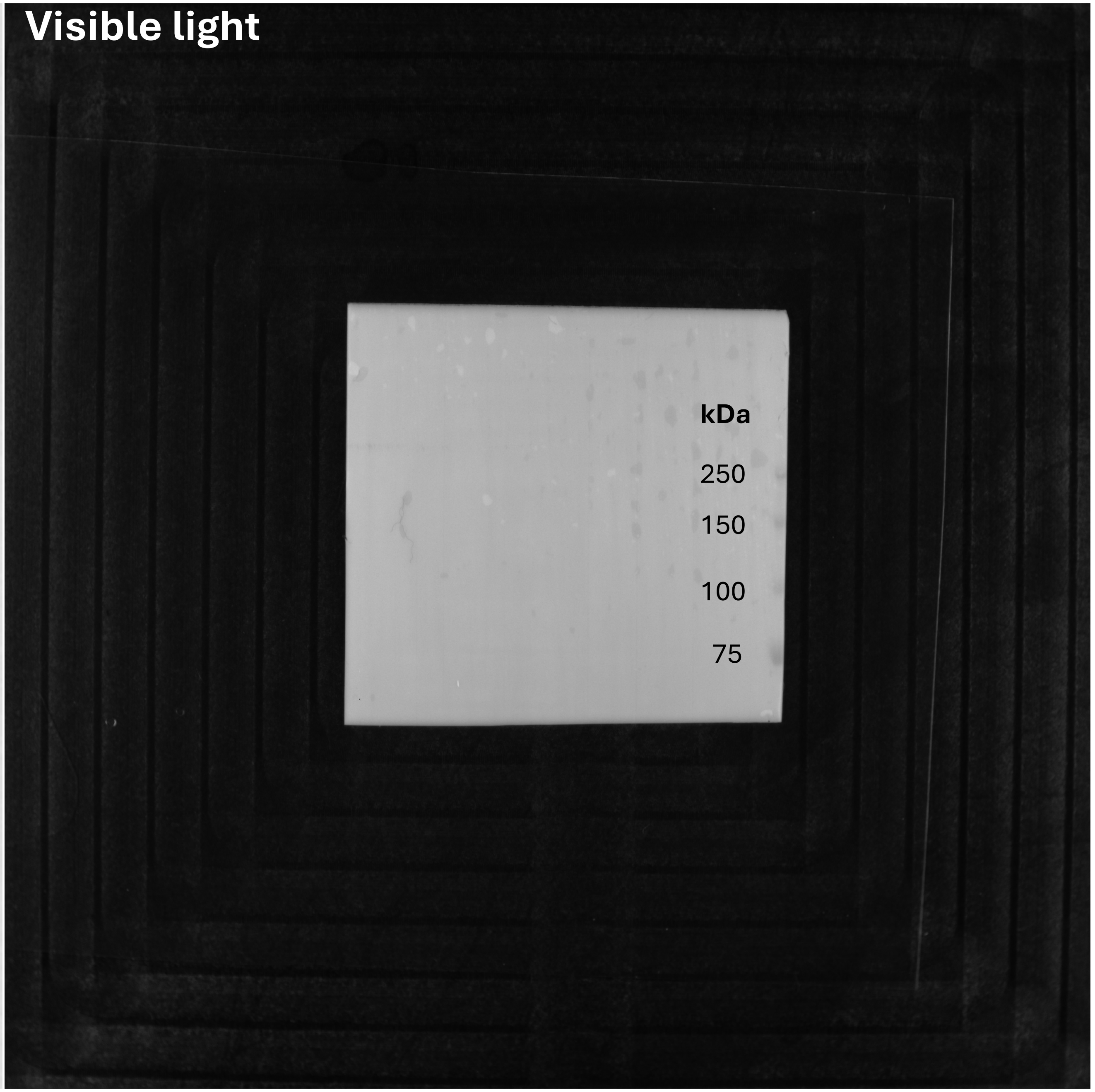

SourceDataF3F_mEV/MV-Integrin β3

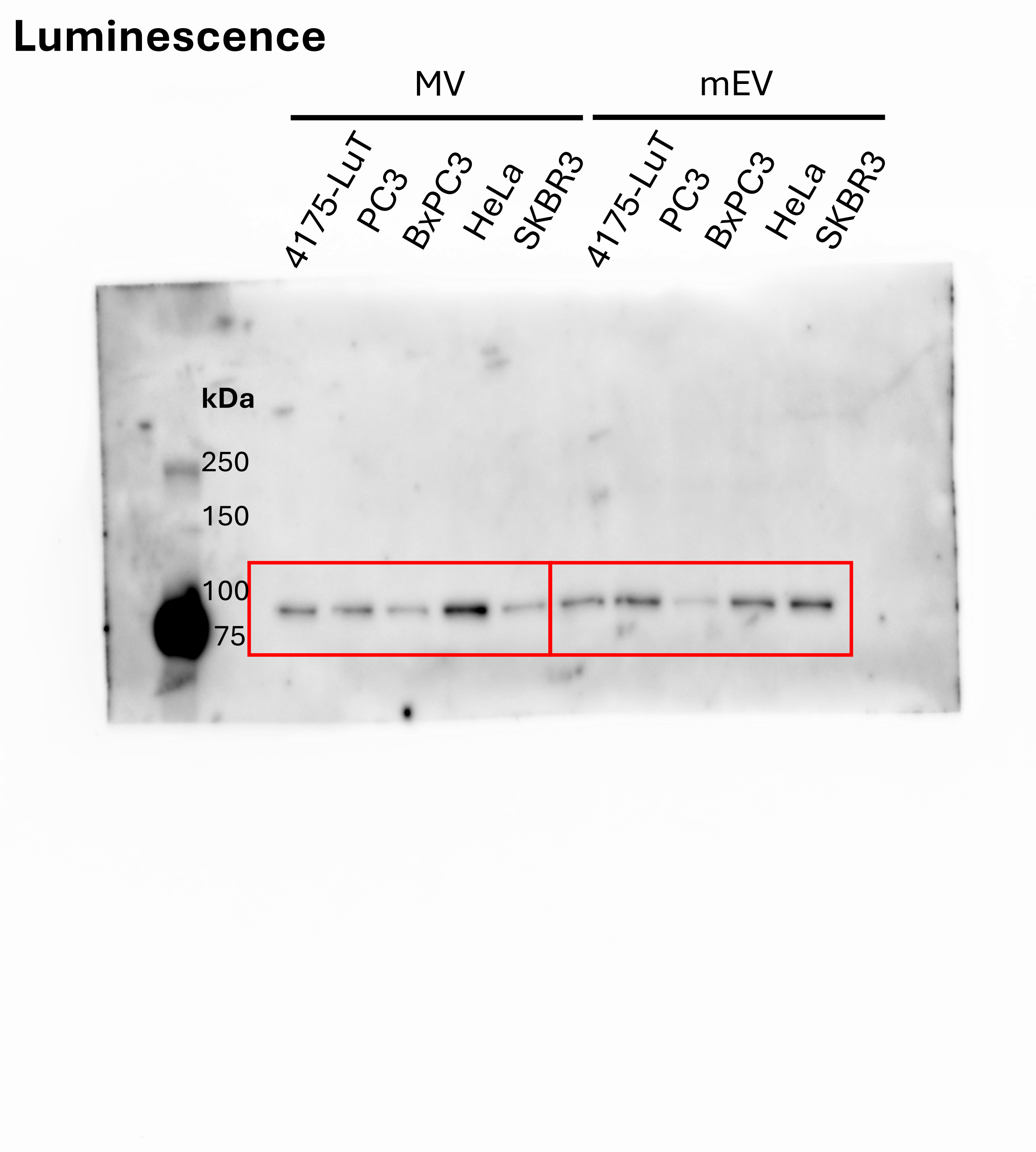

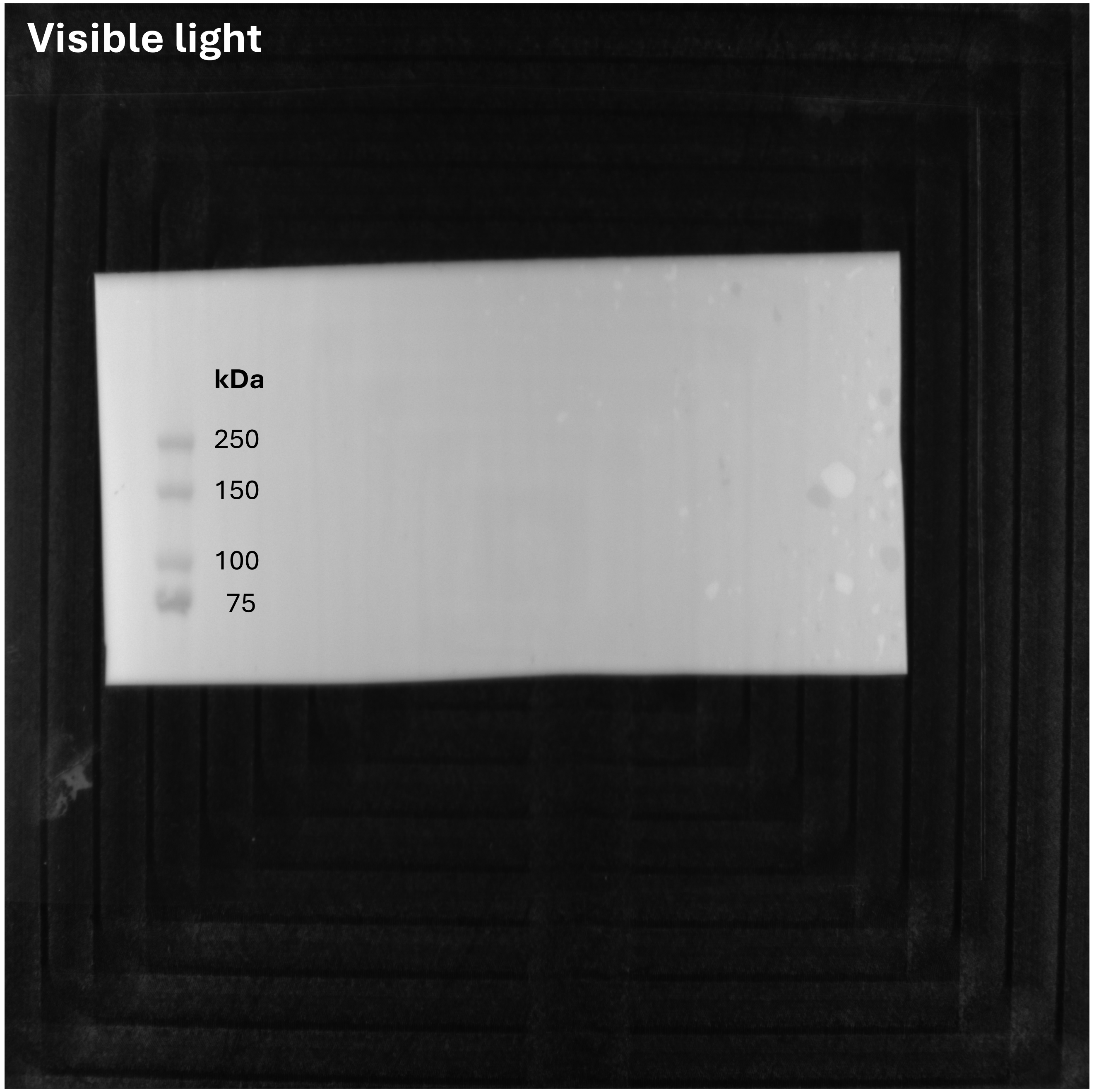

SourceDataF3F_sEV-Integrin β5

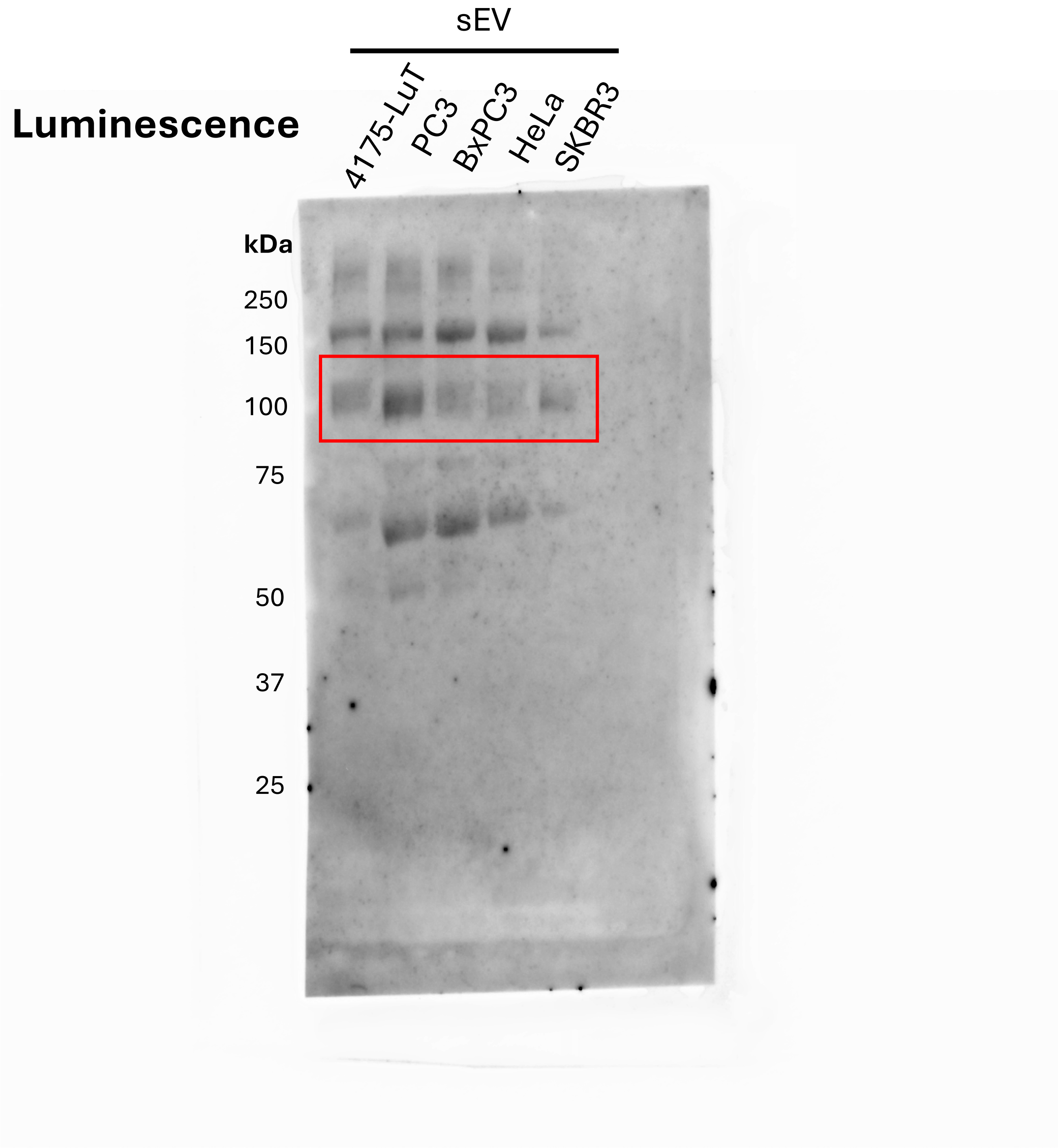

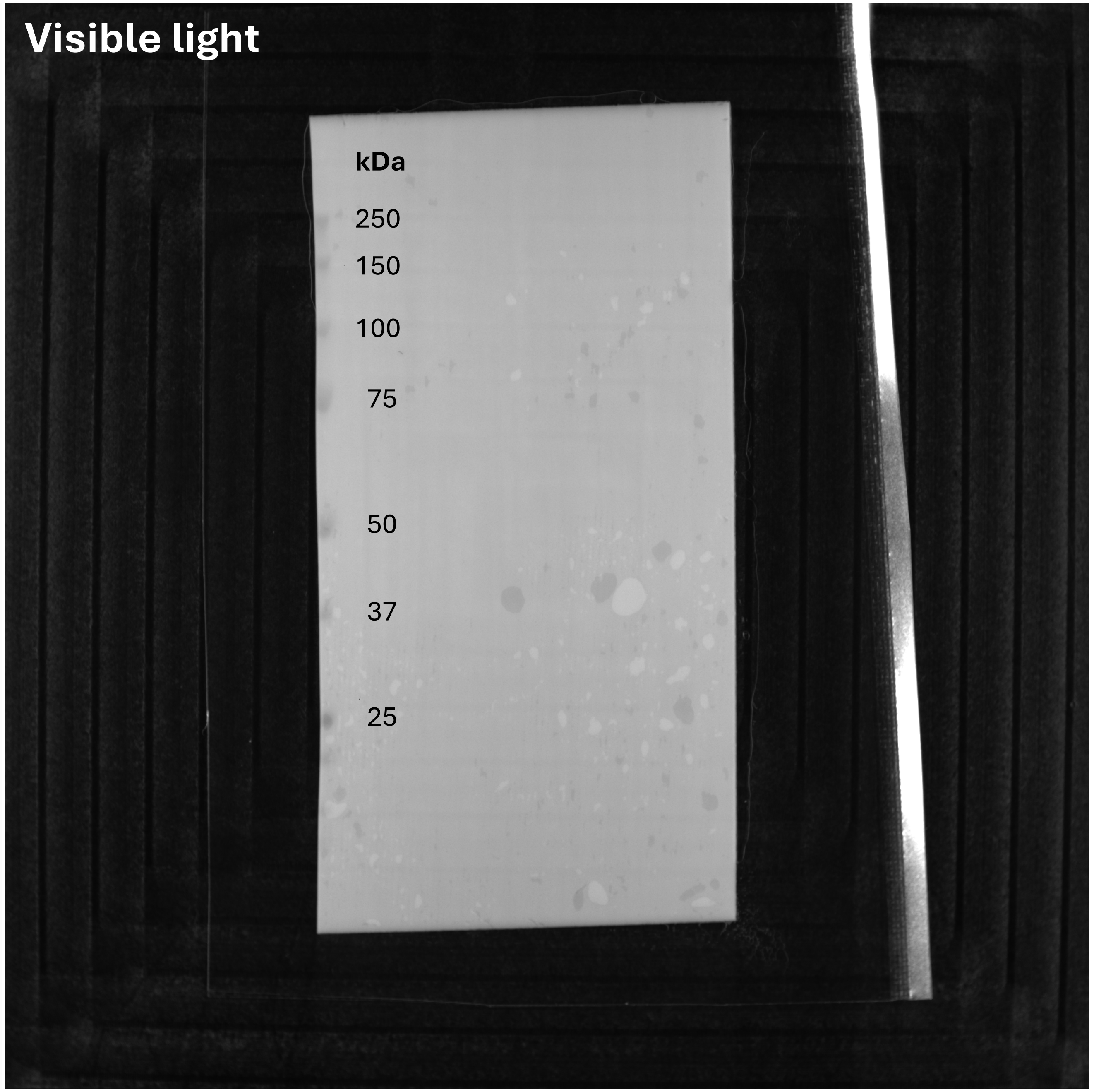

SourceDataF3F_mEV-Integrin β5

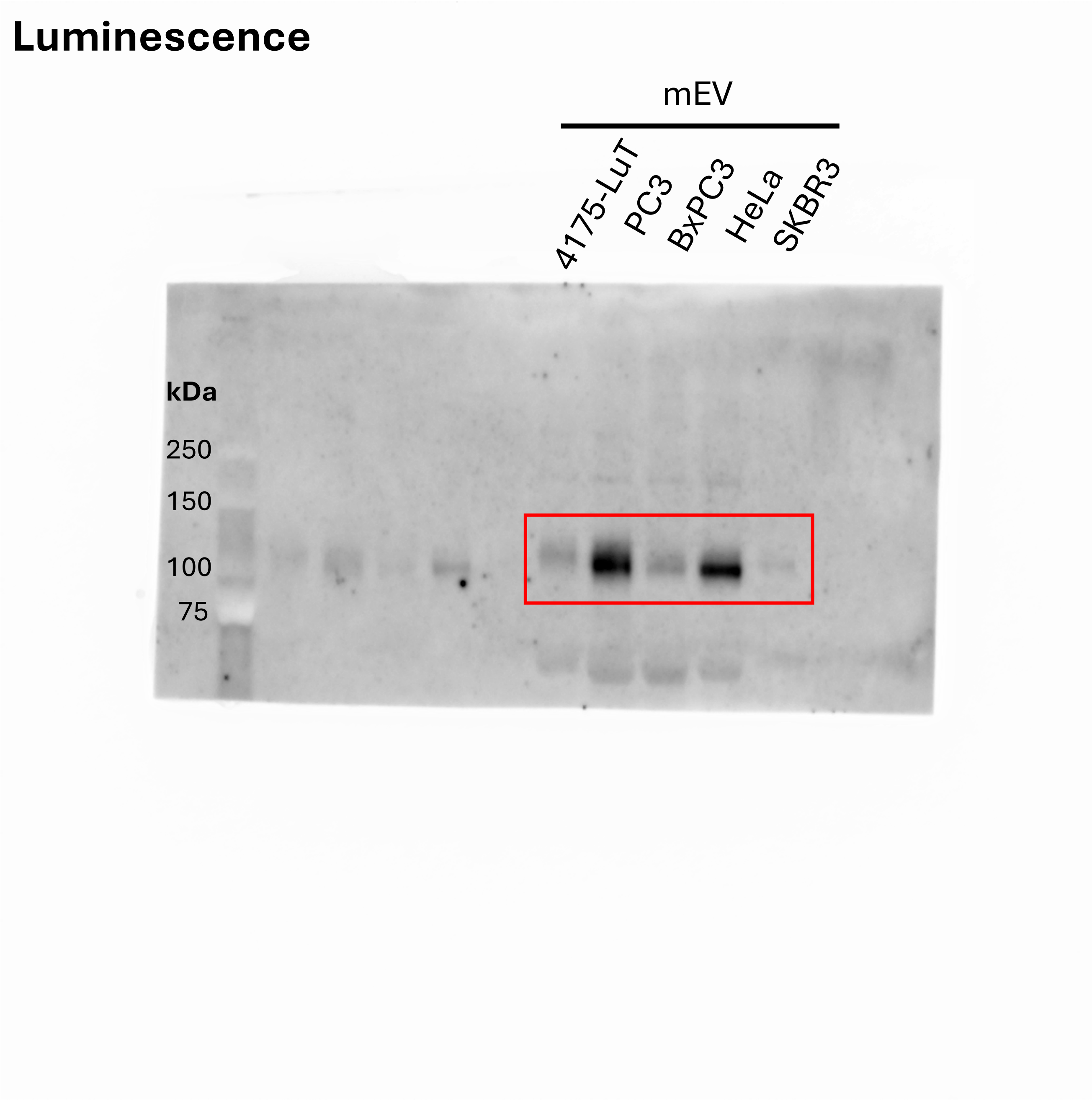

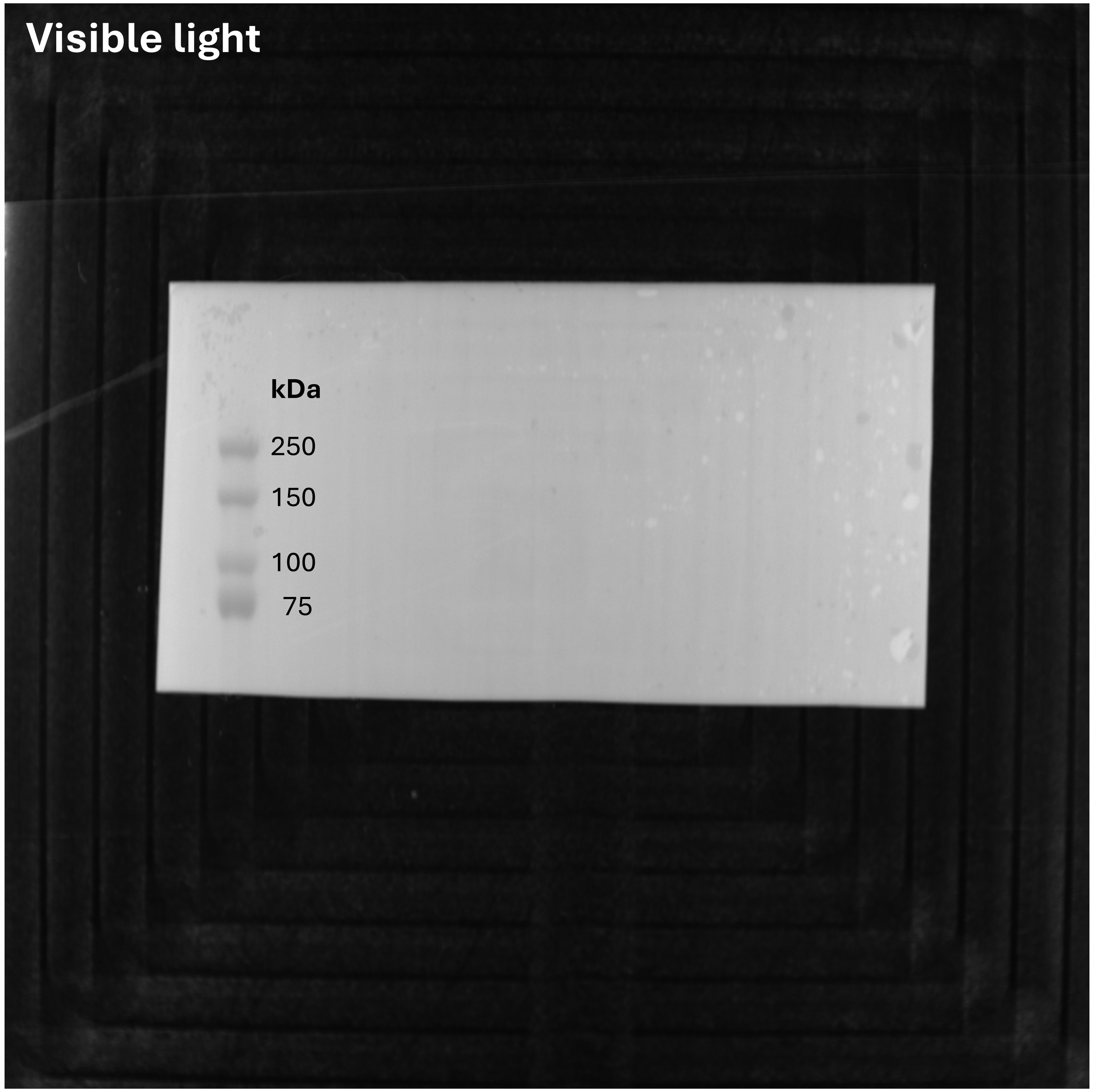

SourceDataF3F_MV-Integrin β5

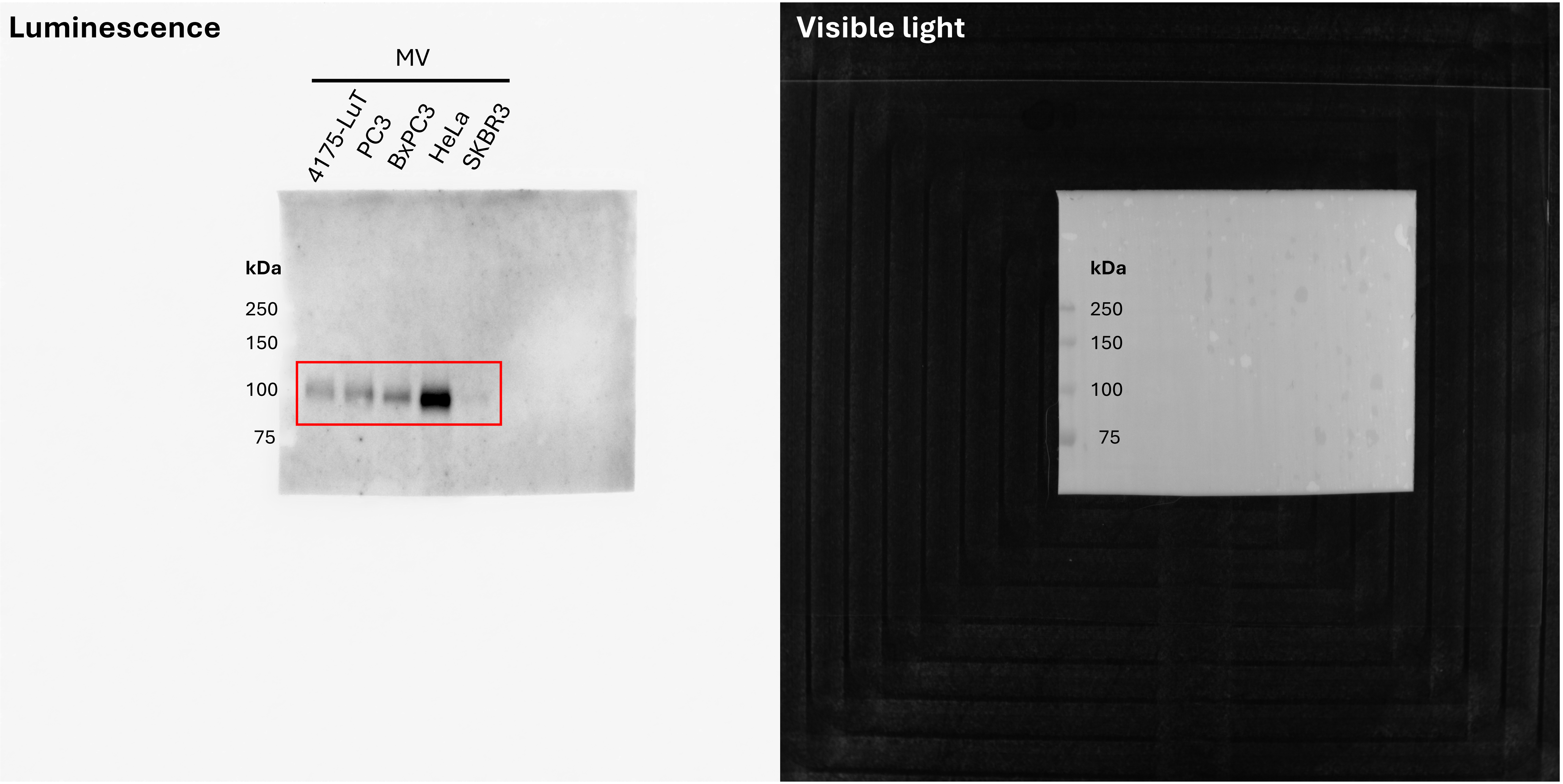

SourceDataF3F_sEV/mEV/MV-Integrin α2

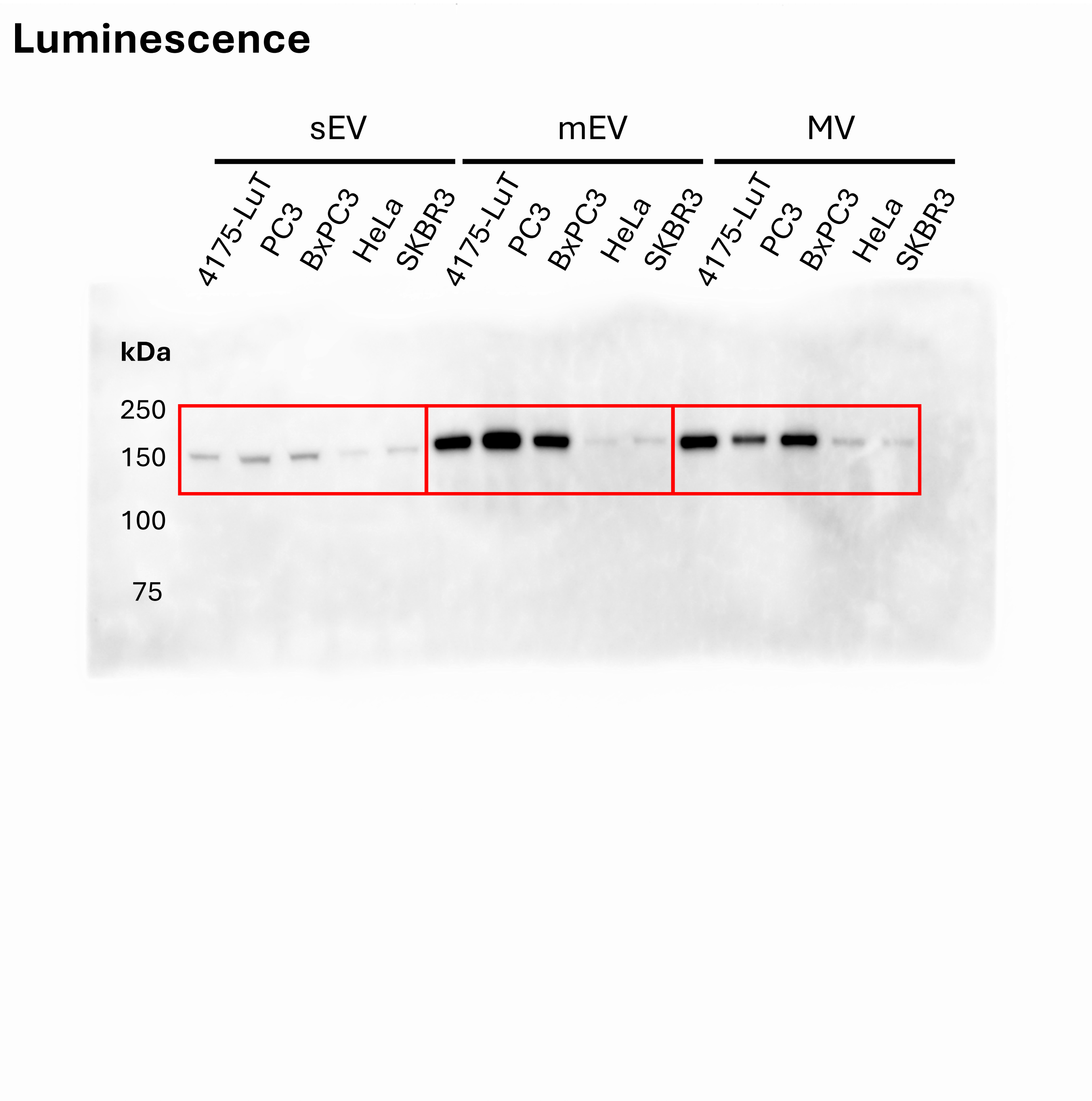

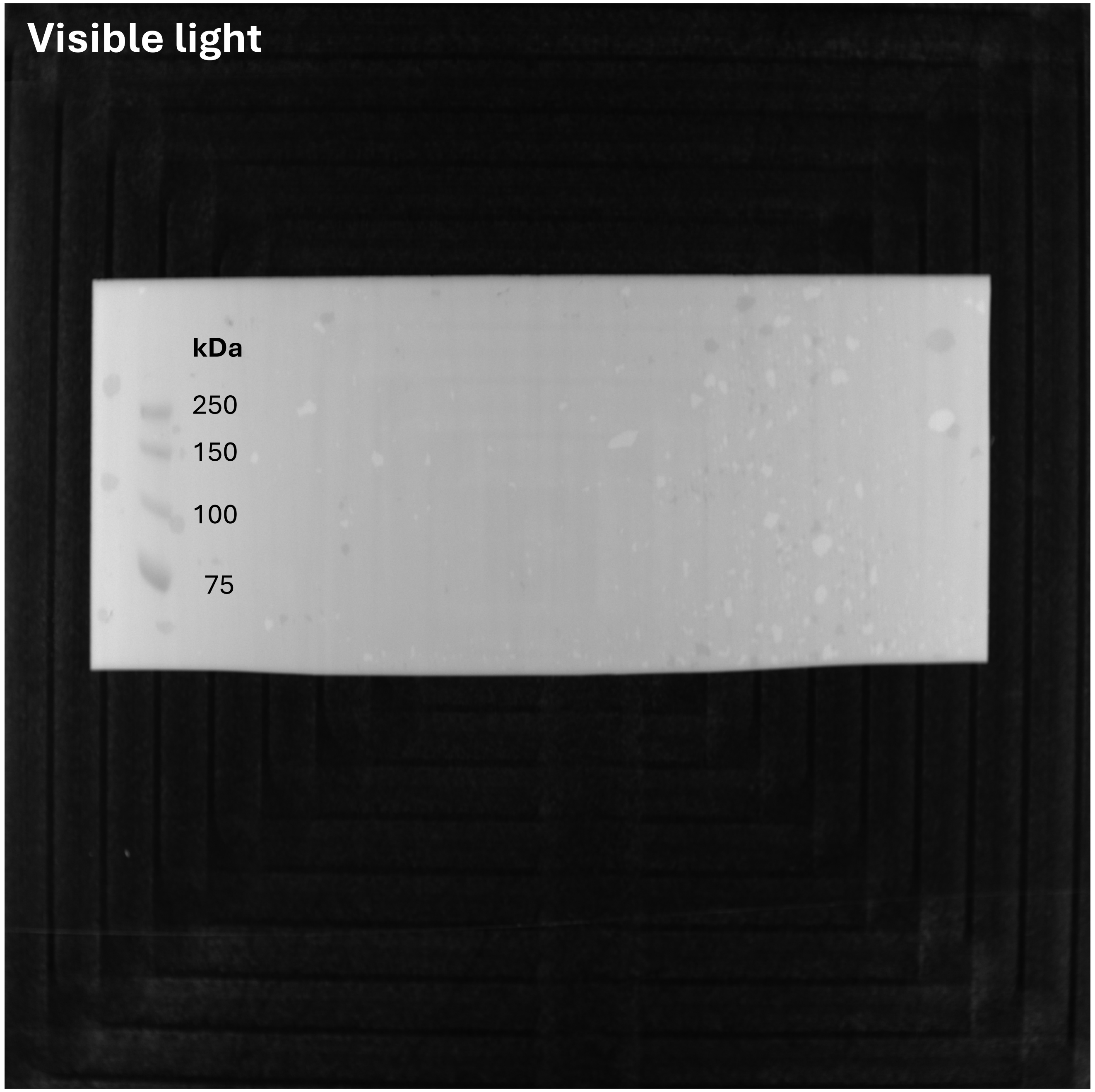

SourceDataF3F_sEV/mEV/MV-Integrin α3

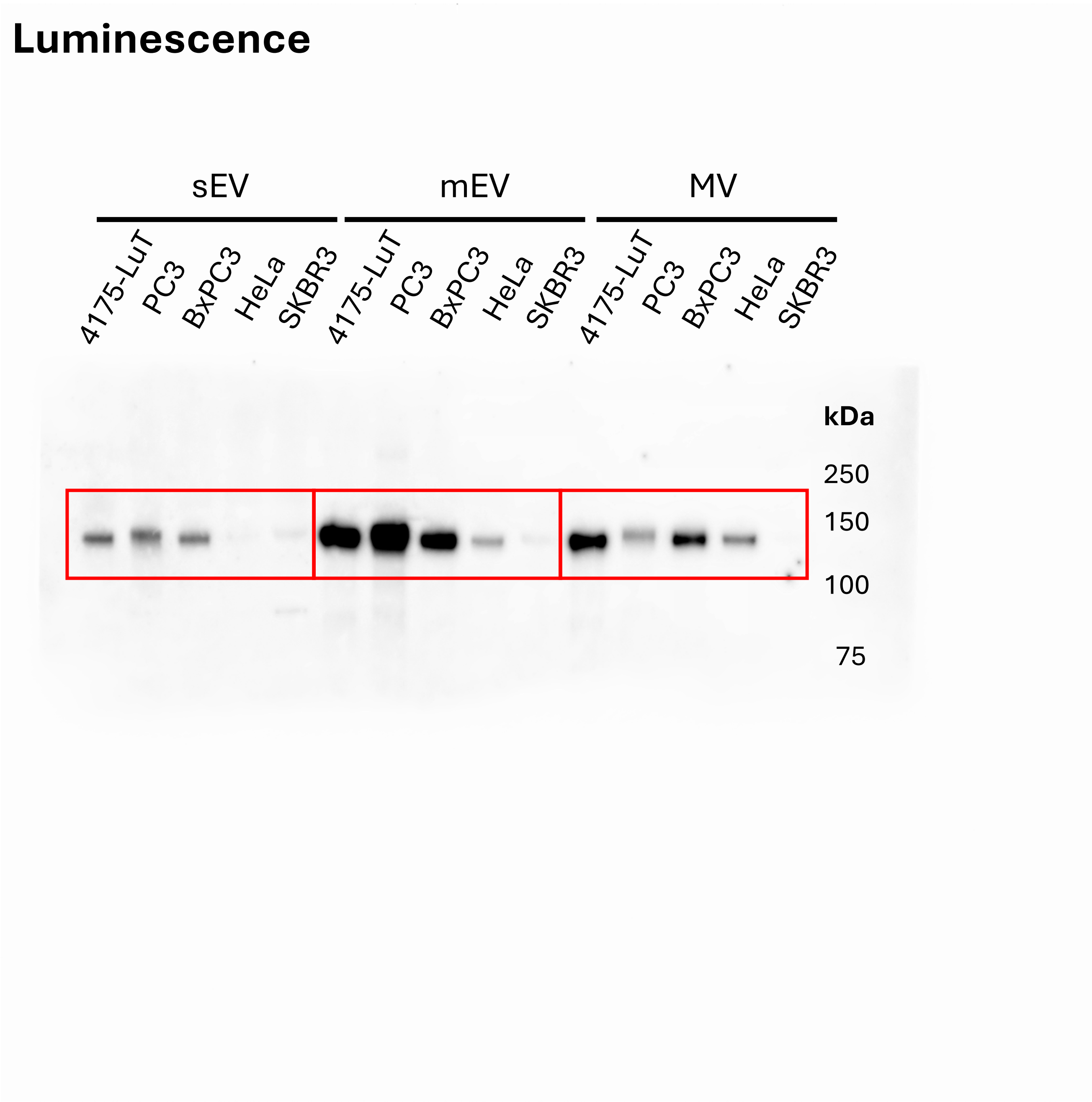

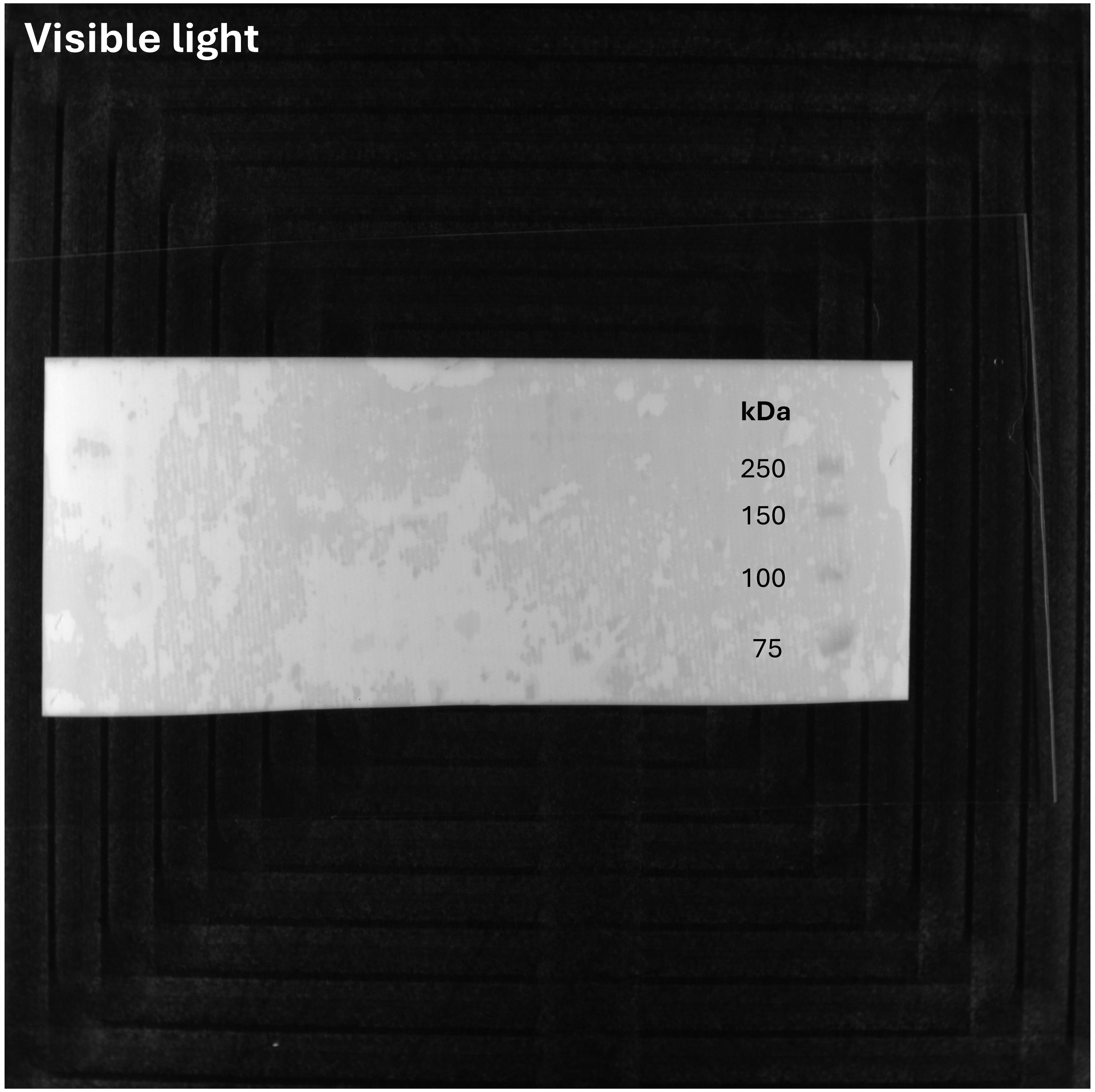

SourceDataF3F_sEV/mEV/MV-Integrin β4

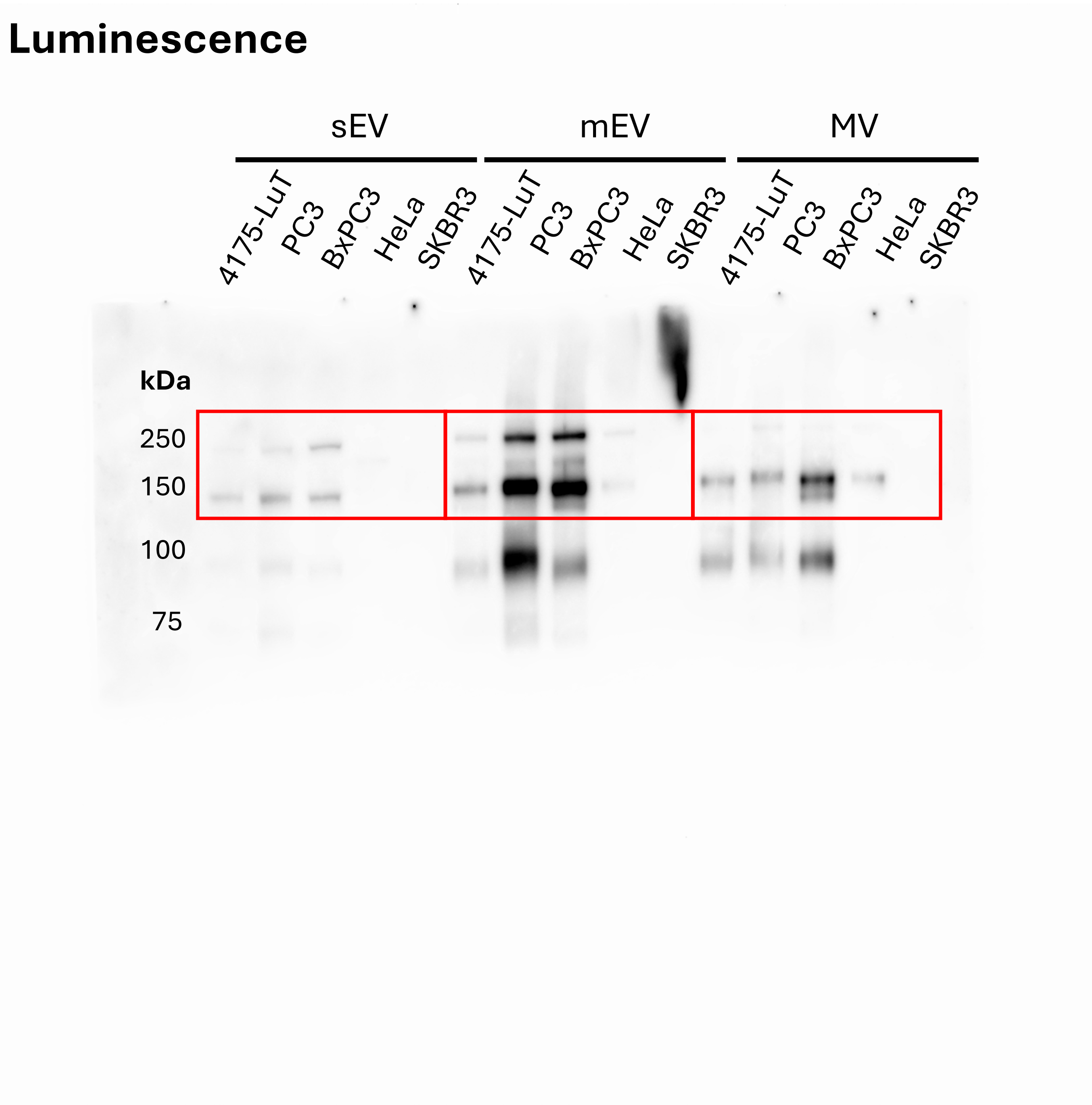

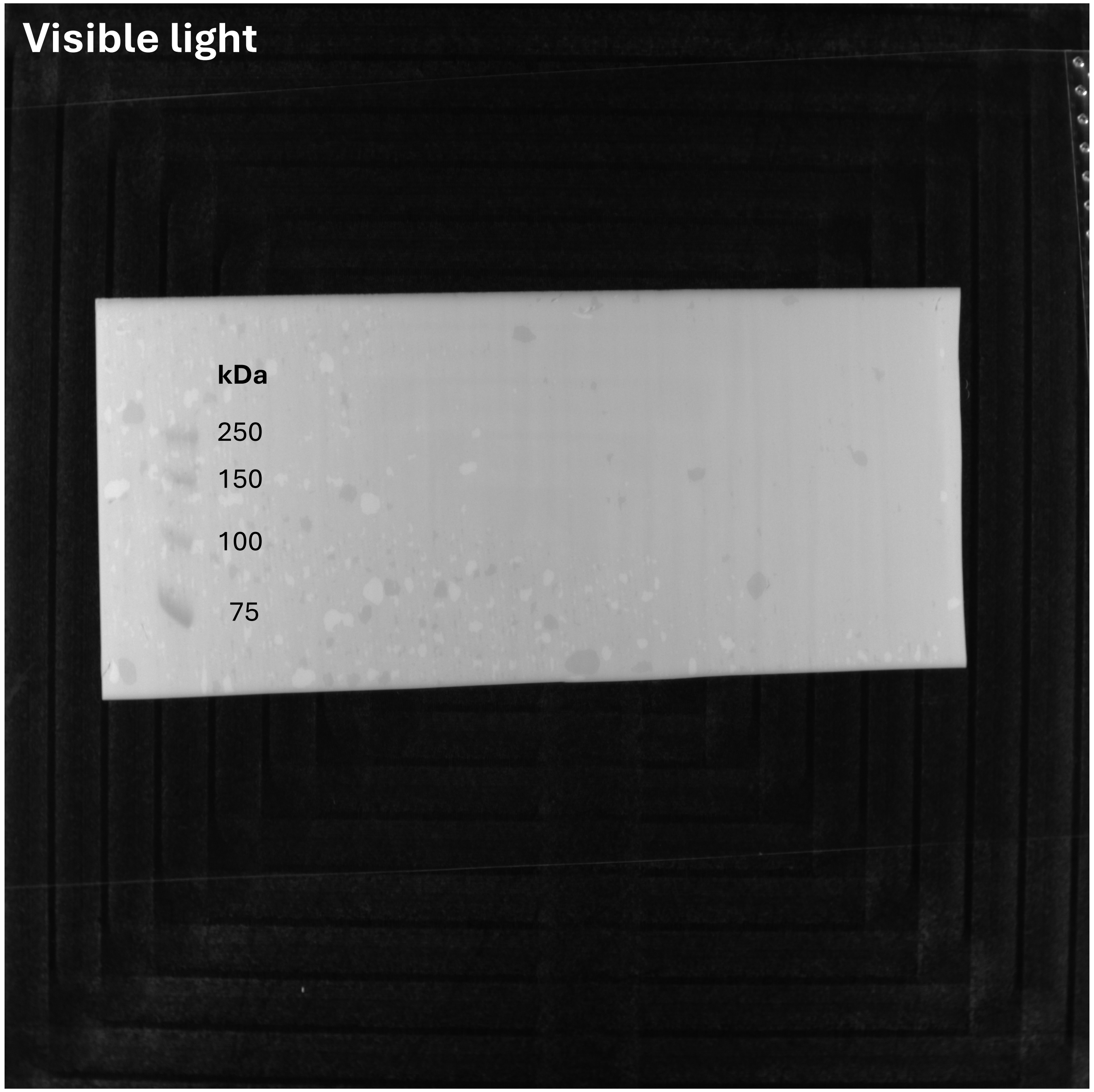

SourceDataF3F_sEV-CD63

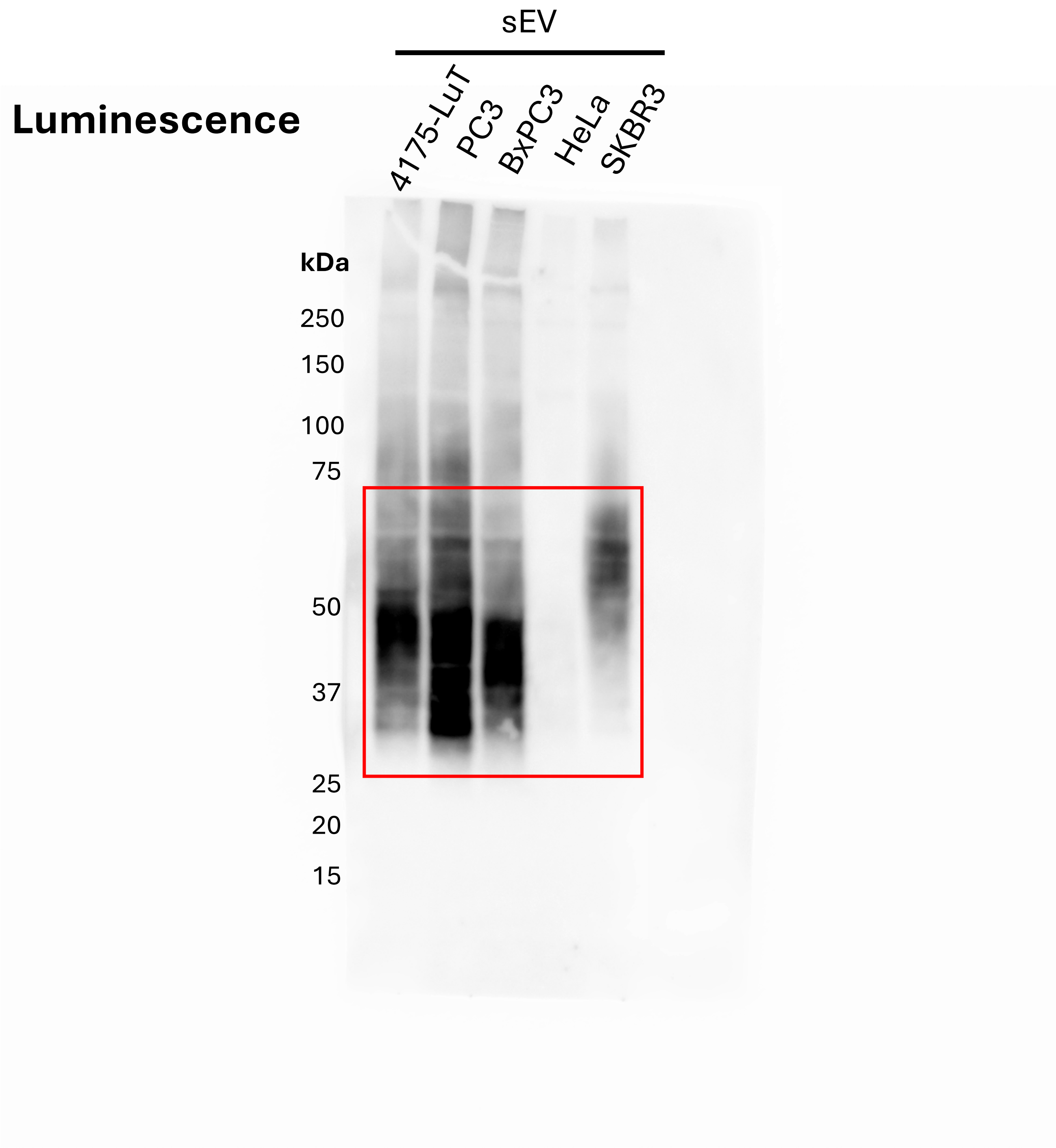

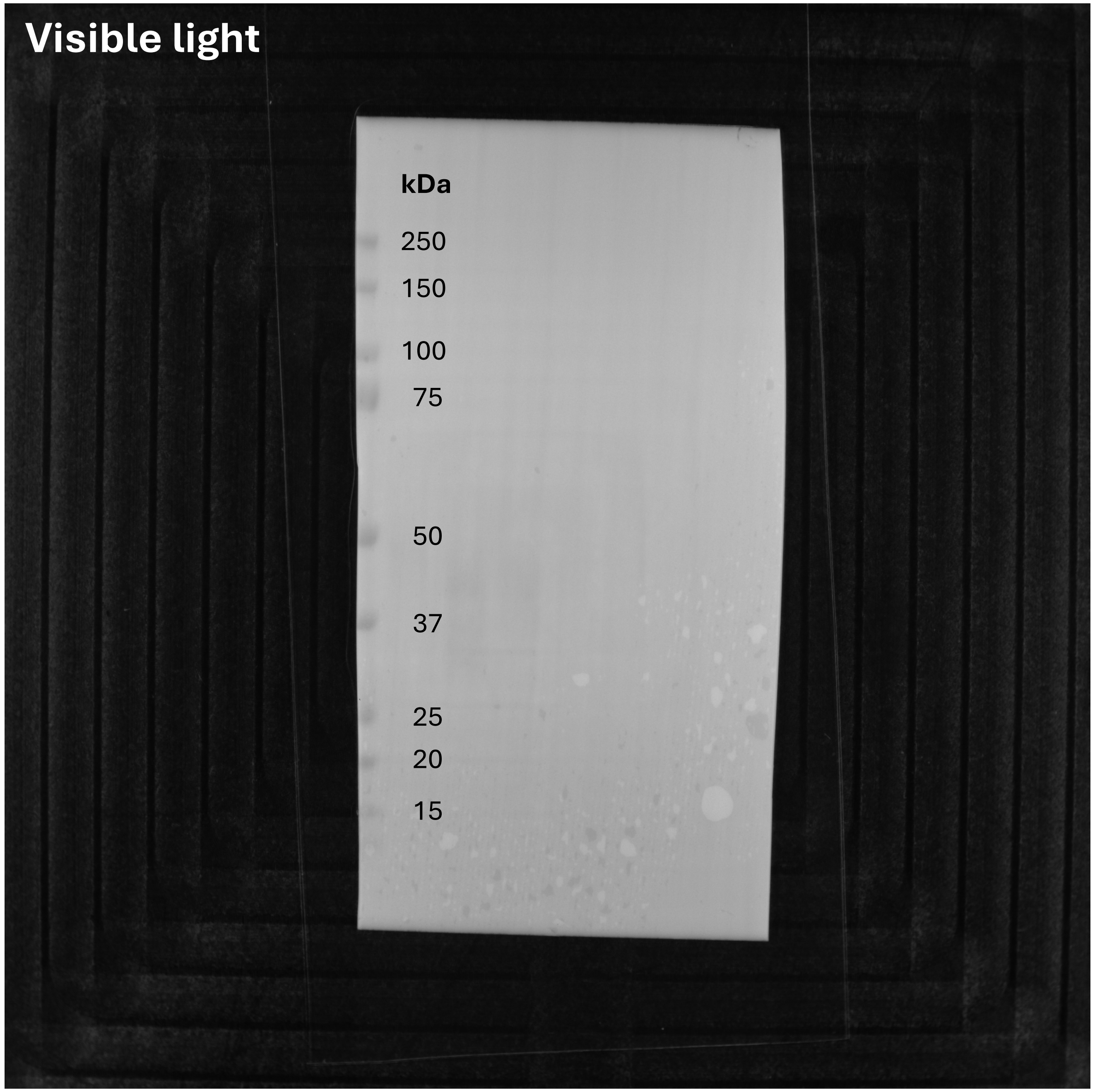

SourceDataF3F_mEV-CD63

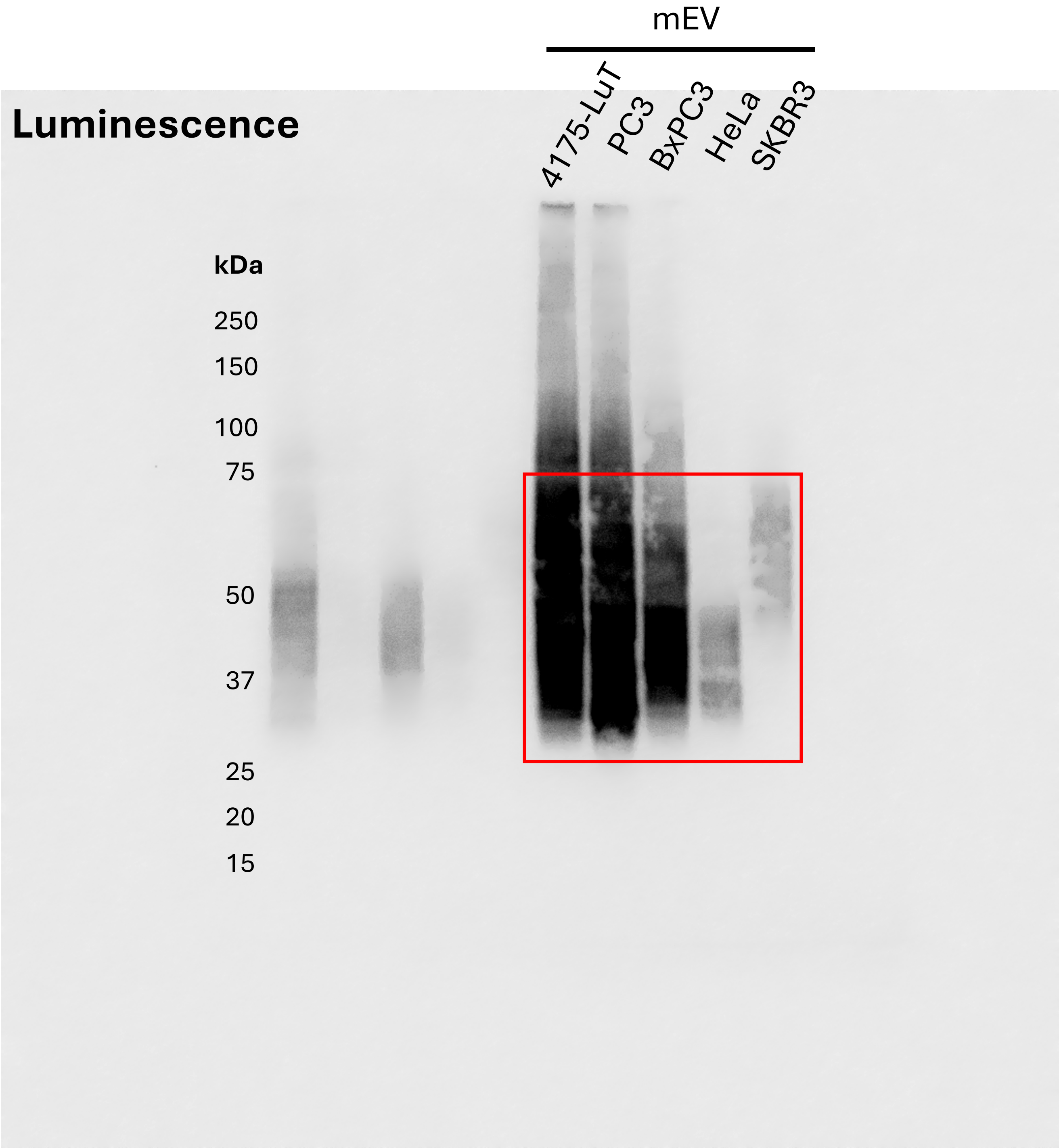

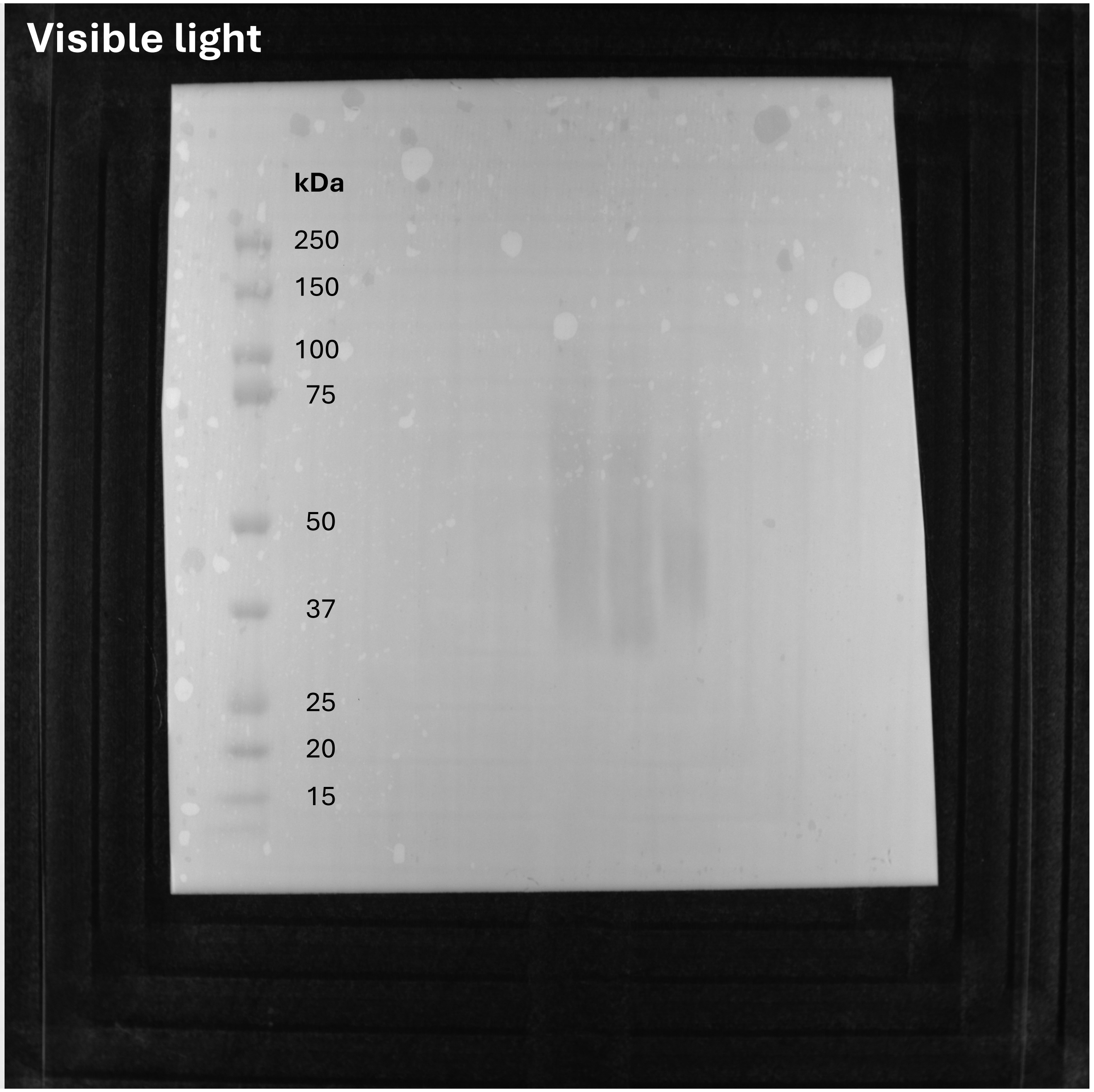

SourceDataF3F_MV-CD63

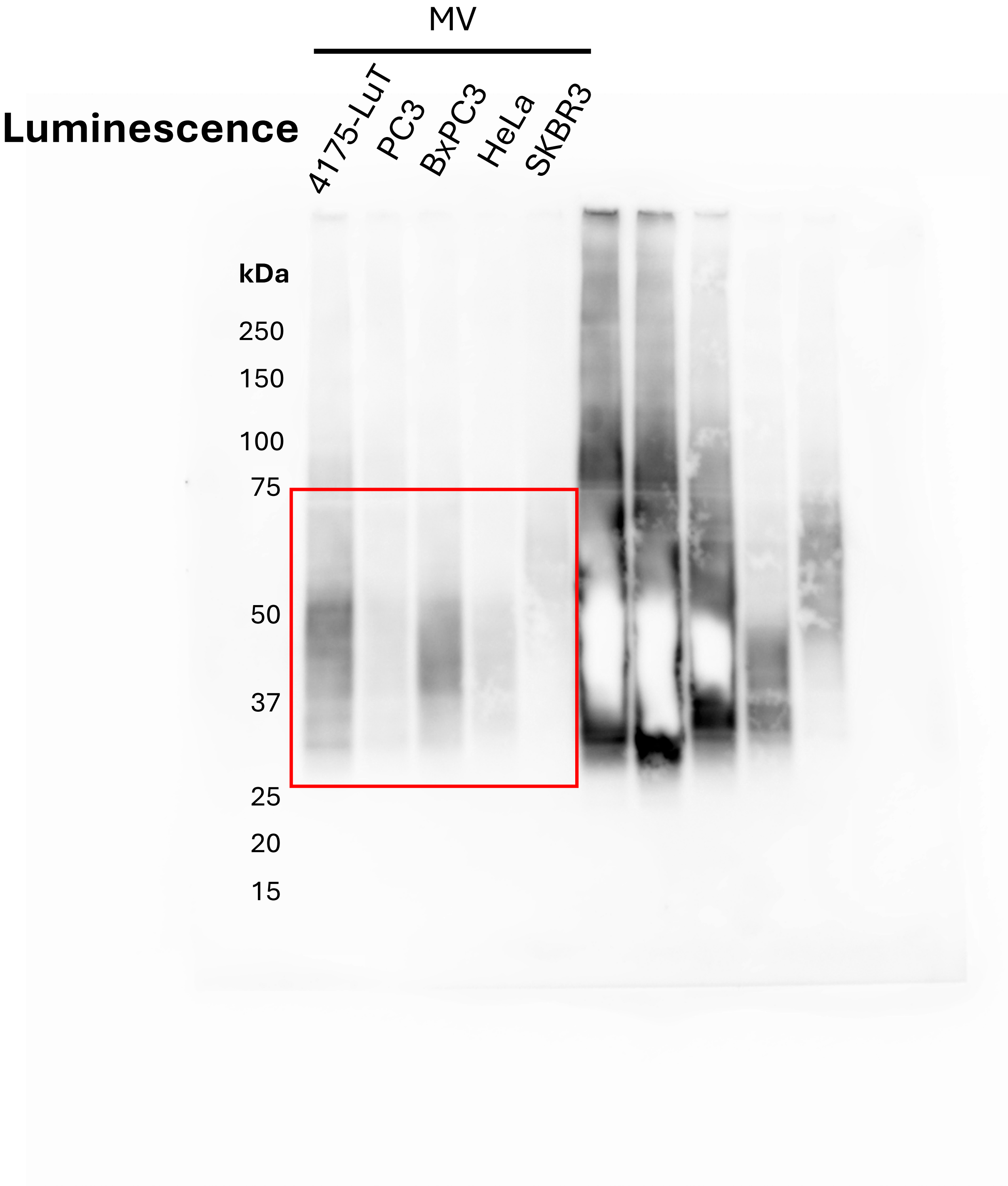

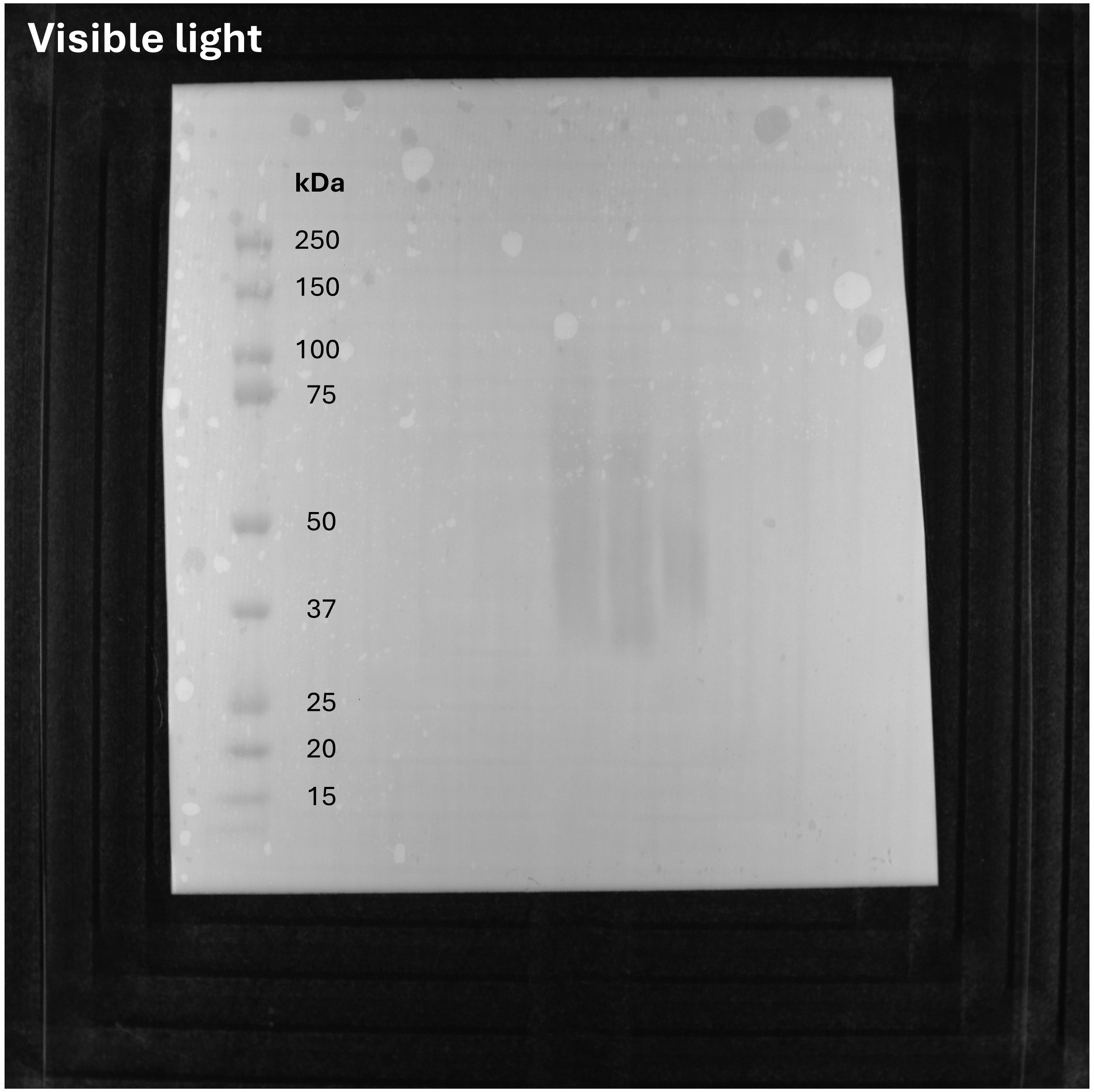

SourceDataF3F_sEV-β-actin

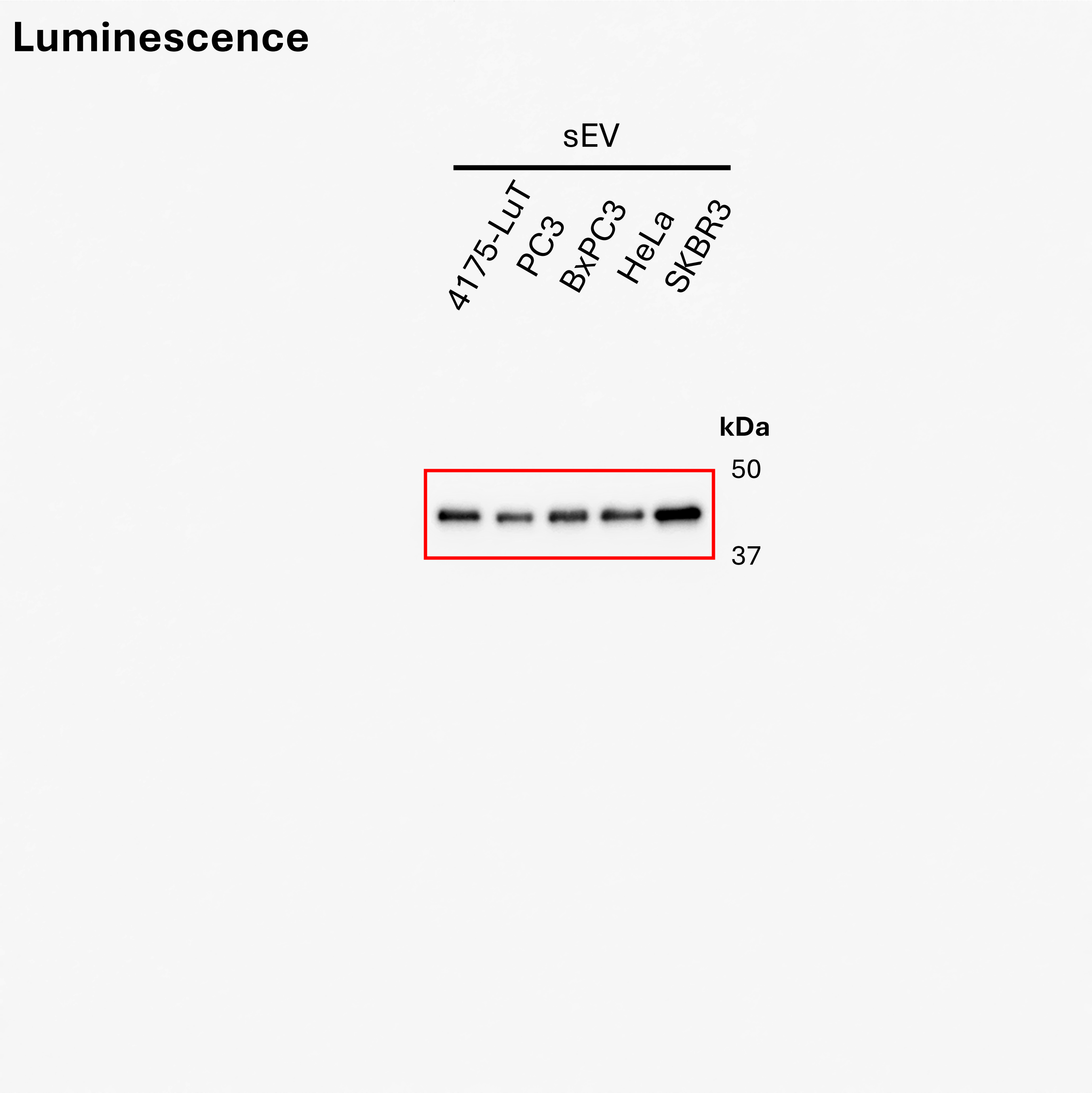

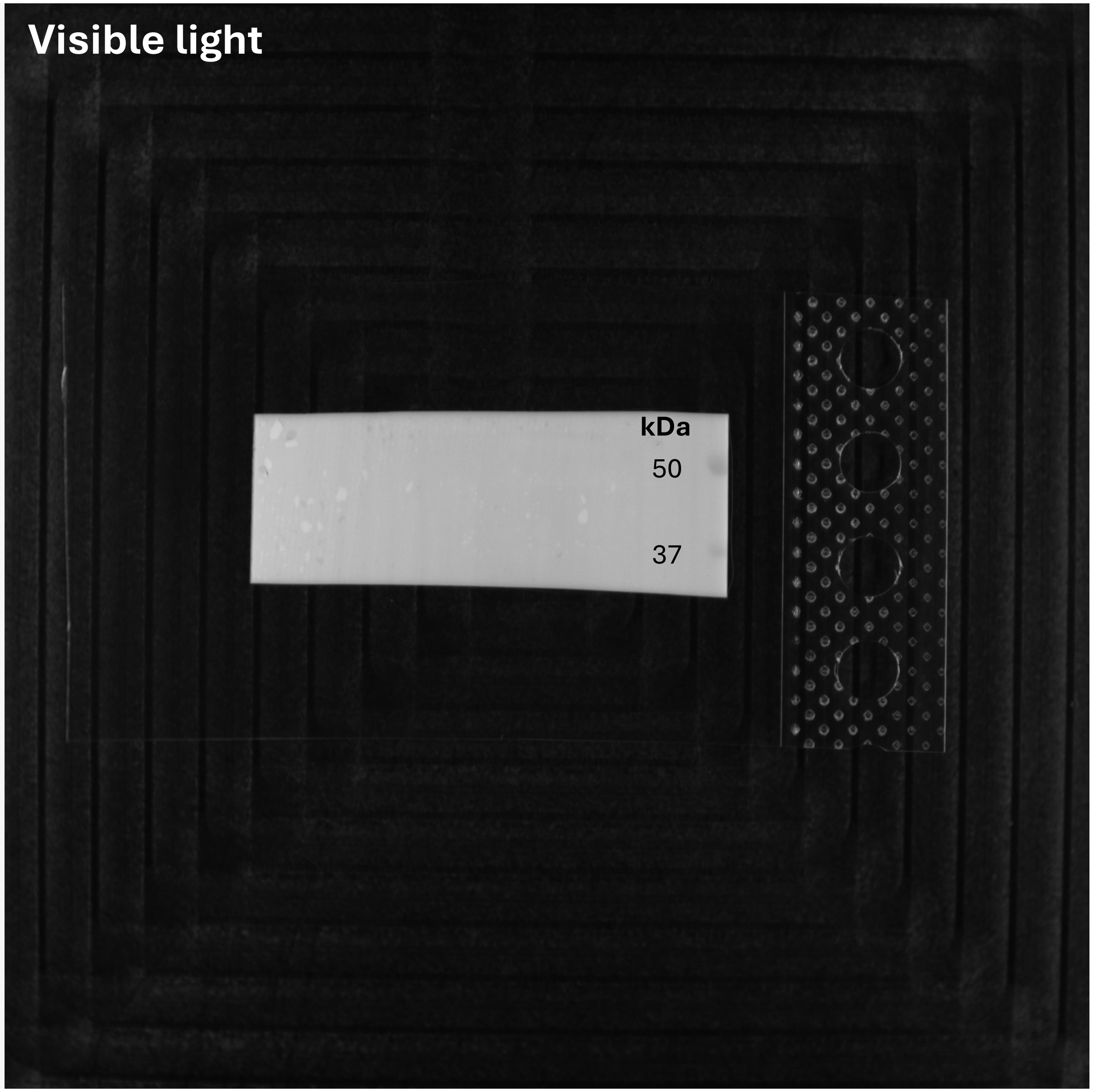

SourceDataF3F_mEV/MV-β-actin

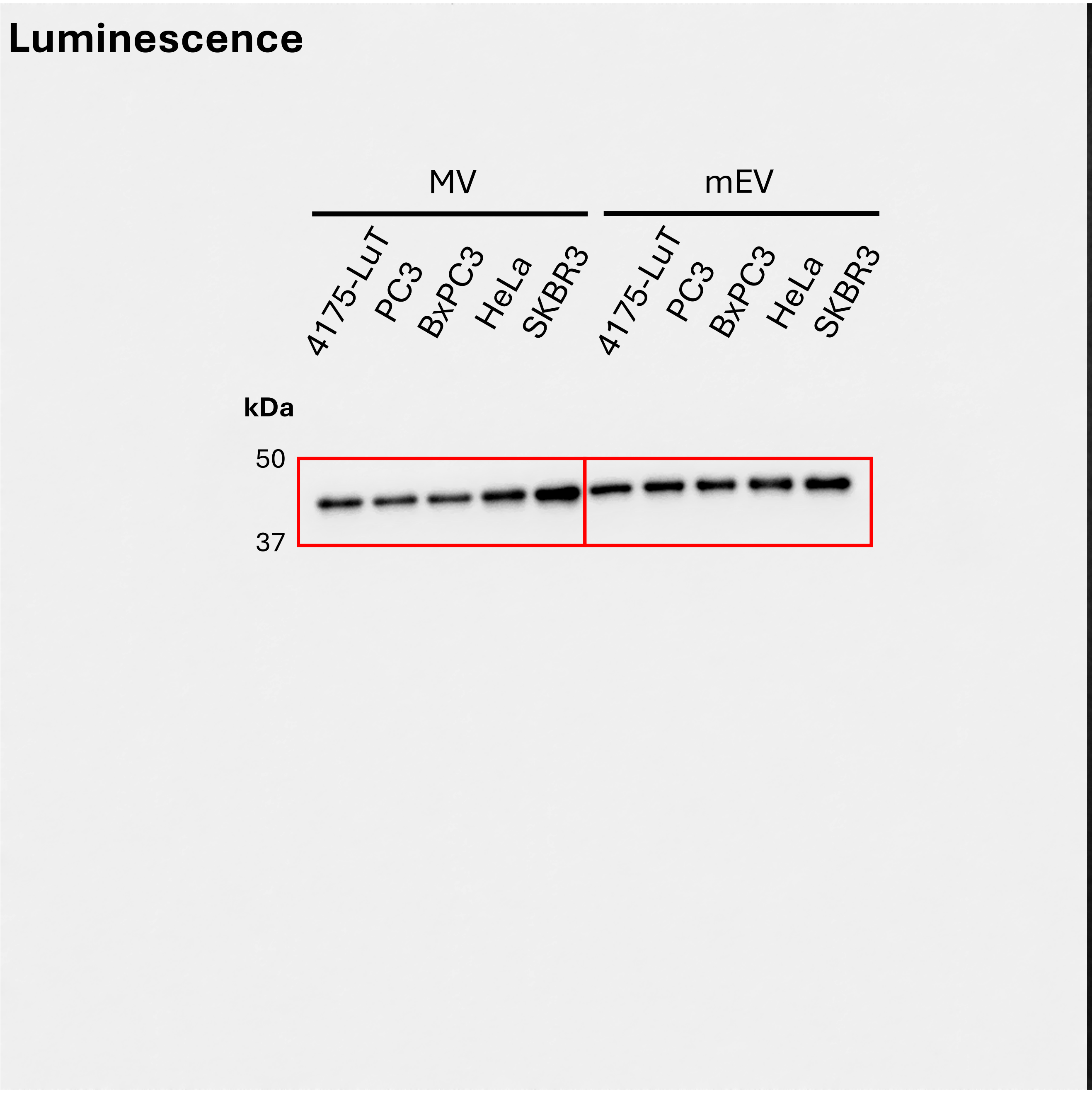

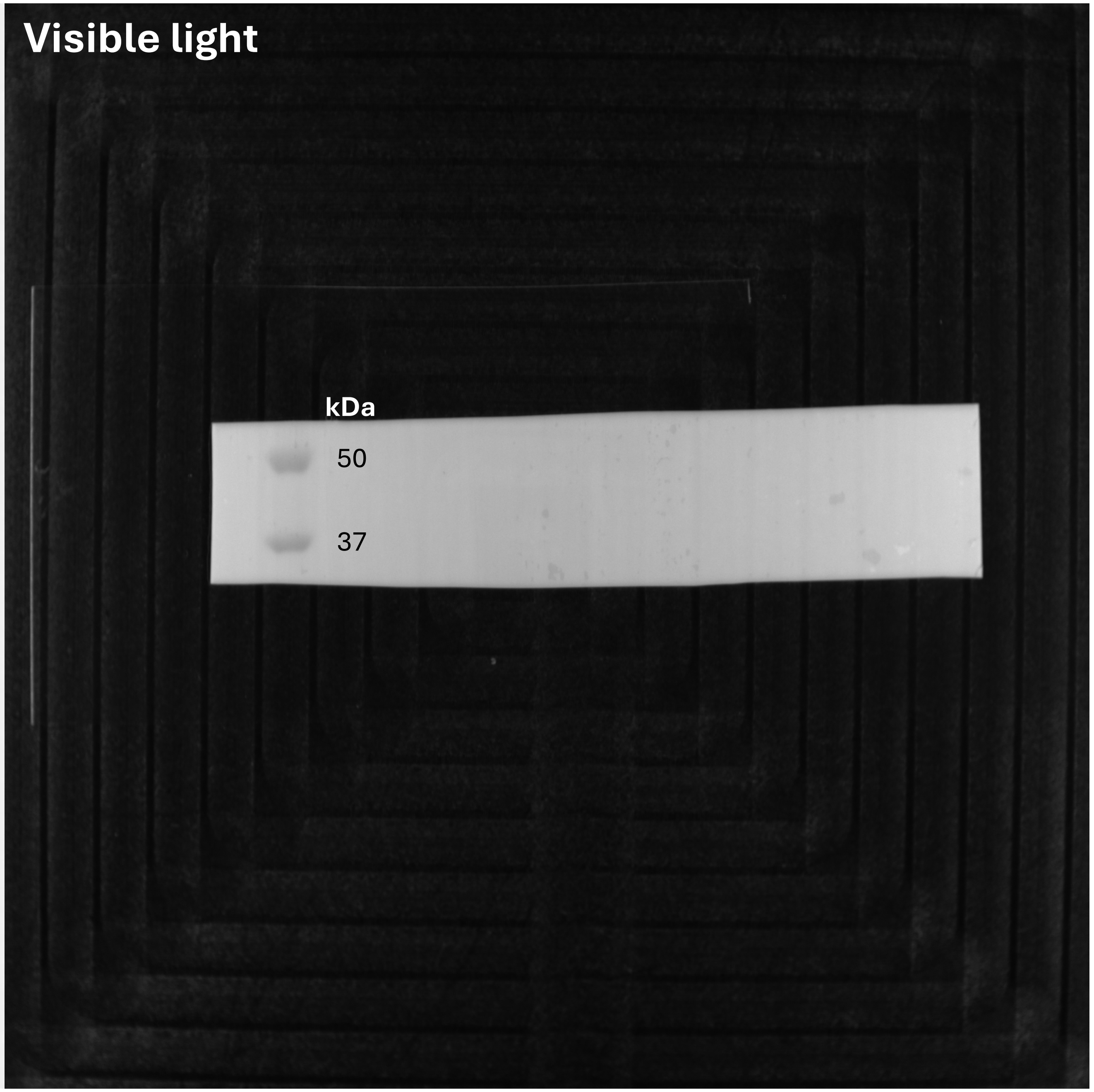

Supplement: SourceData F3 — is the source file for Fig. 3. [file jcb_202404064_sourcedataf3.pdf]
