# Supplementary figures and images for: Extracellular vesicles adhere to cells primarily by interactions of integrins and GM1 with laminin
Source: J Cell Biol. 2025 Apr 30;224(6):e202404064. doi: 10.1083/jcb.202404064 (PMC12042775; doi:10.1083/jcb.202404064)

Fig. 5D

D

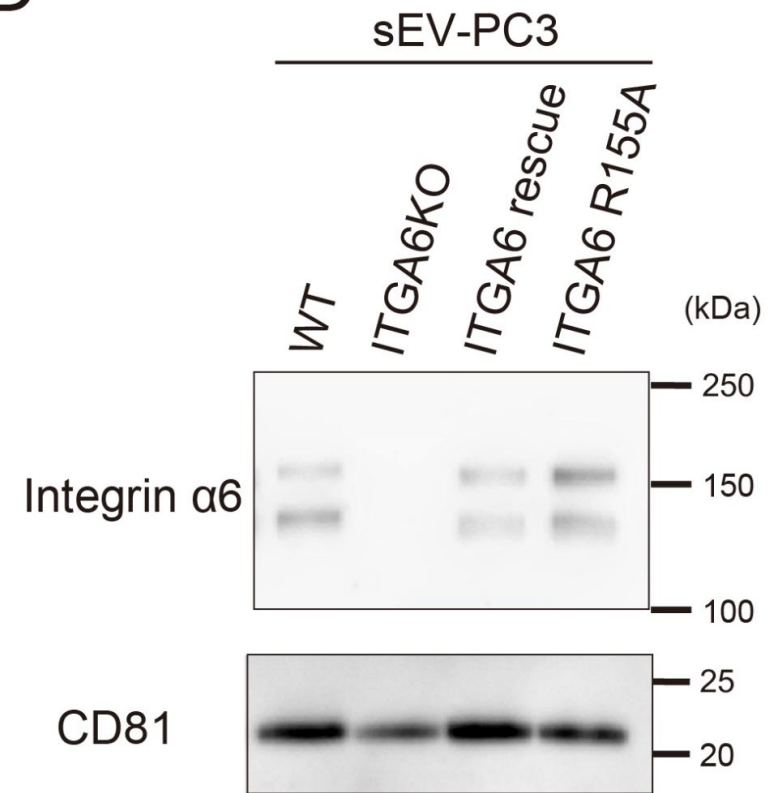

# SourceDataF5D-Integrin α6

Luminescence

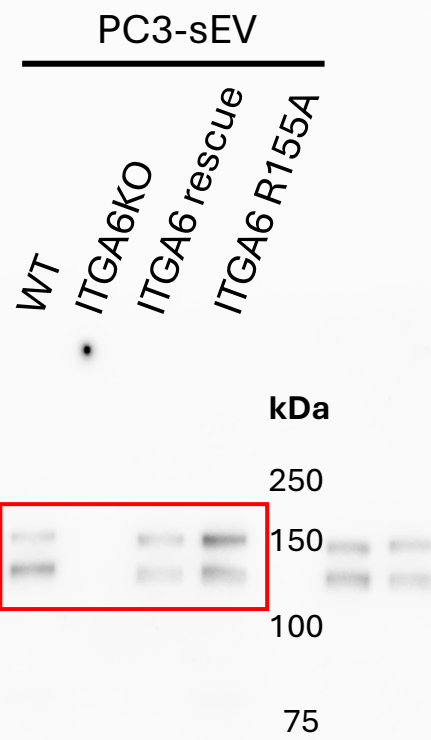

Visible light

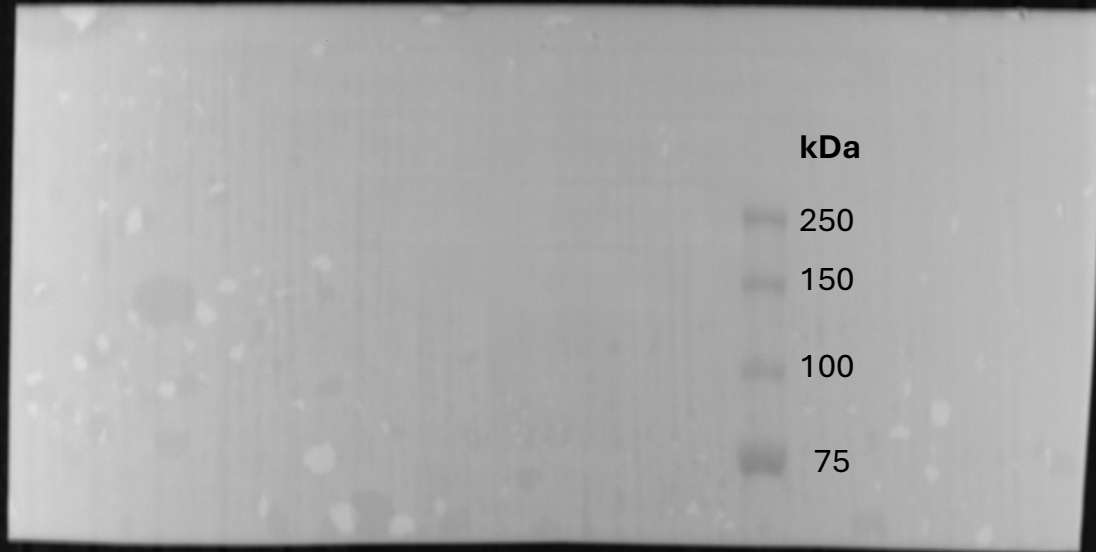

# SourceDataF5D-CD81

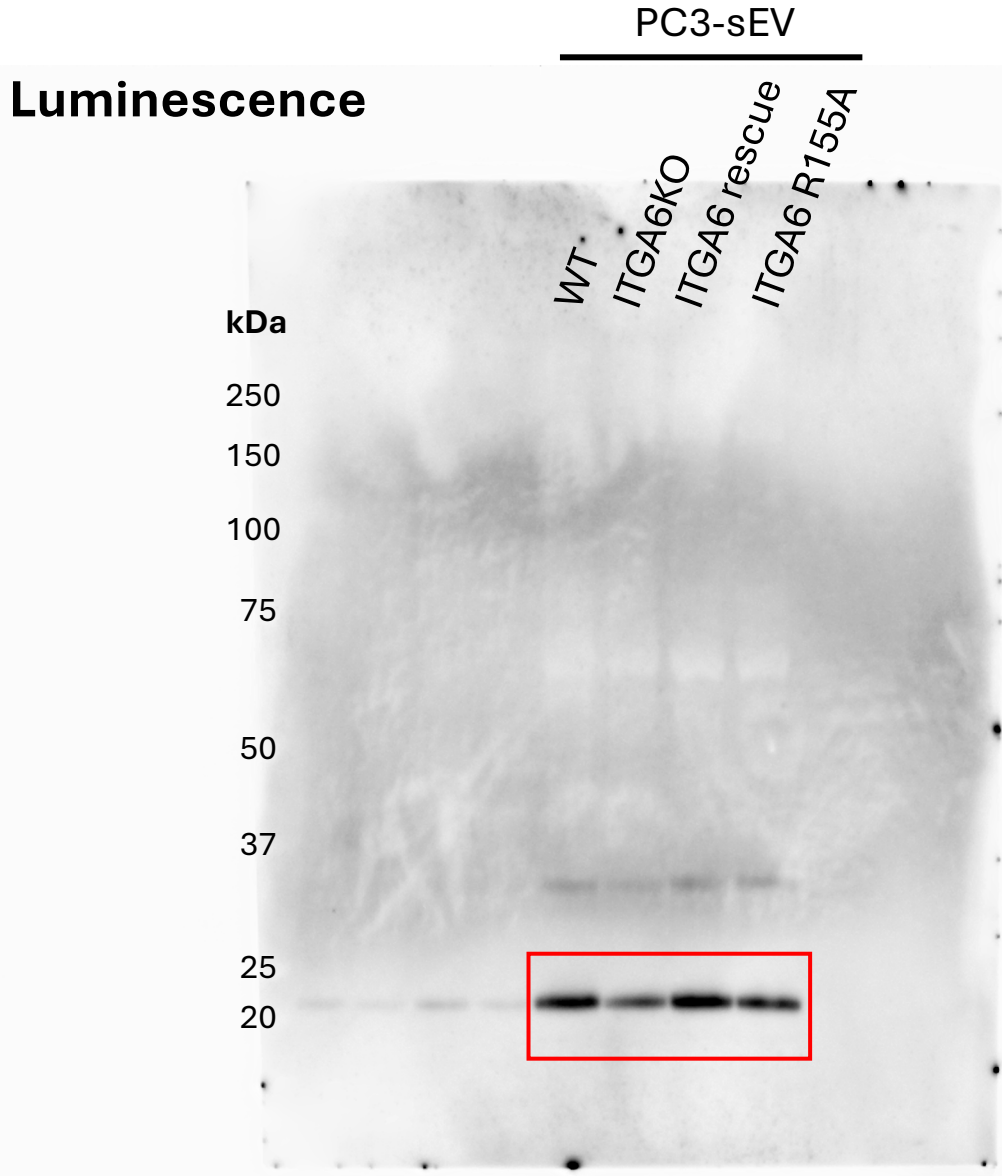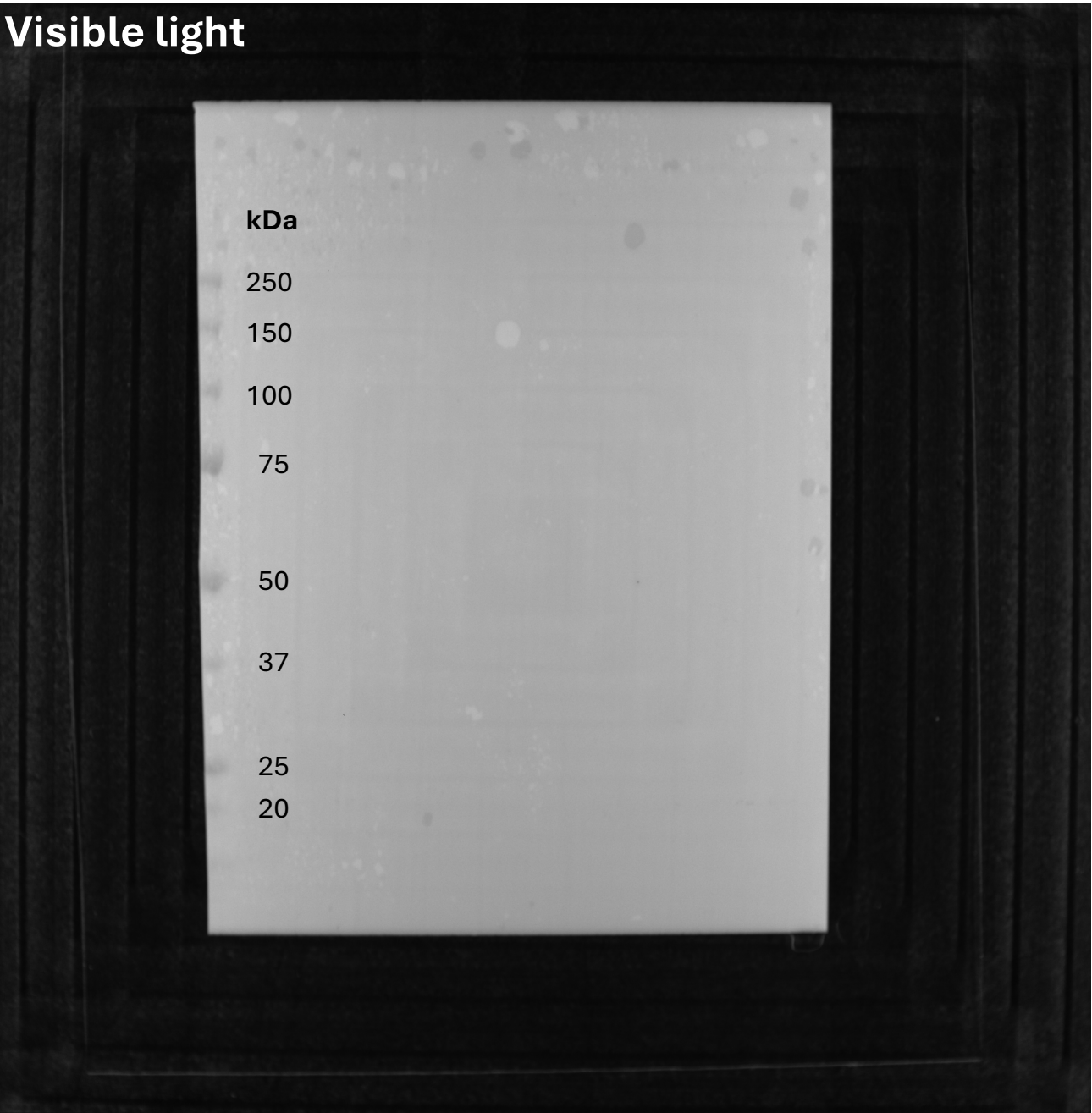

Supplement: SourceData F5 — is the source file for Fig. 5. [file jcb_202404064_sourcedataf5.pdf]
