# Supplementary material for: Extracellular vesicles adhere to cells primarily by interactions of integrins and GM1 with laminin
Source: J Cell Biol. 2025 Apr 30;224(6):e202404064. doi: 10.1083/jcb.202404064 (PMC12042775; doi:10.1083/jcb.202404064)

Fig. 7A, 7D, 7M, 7Q, 7R

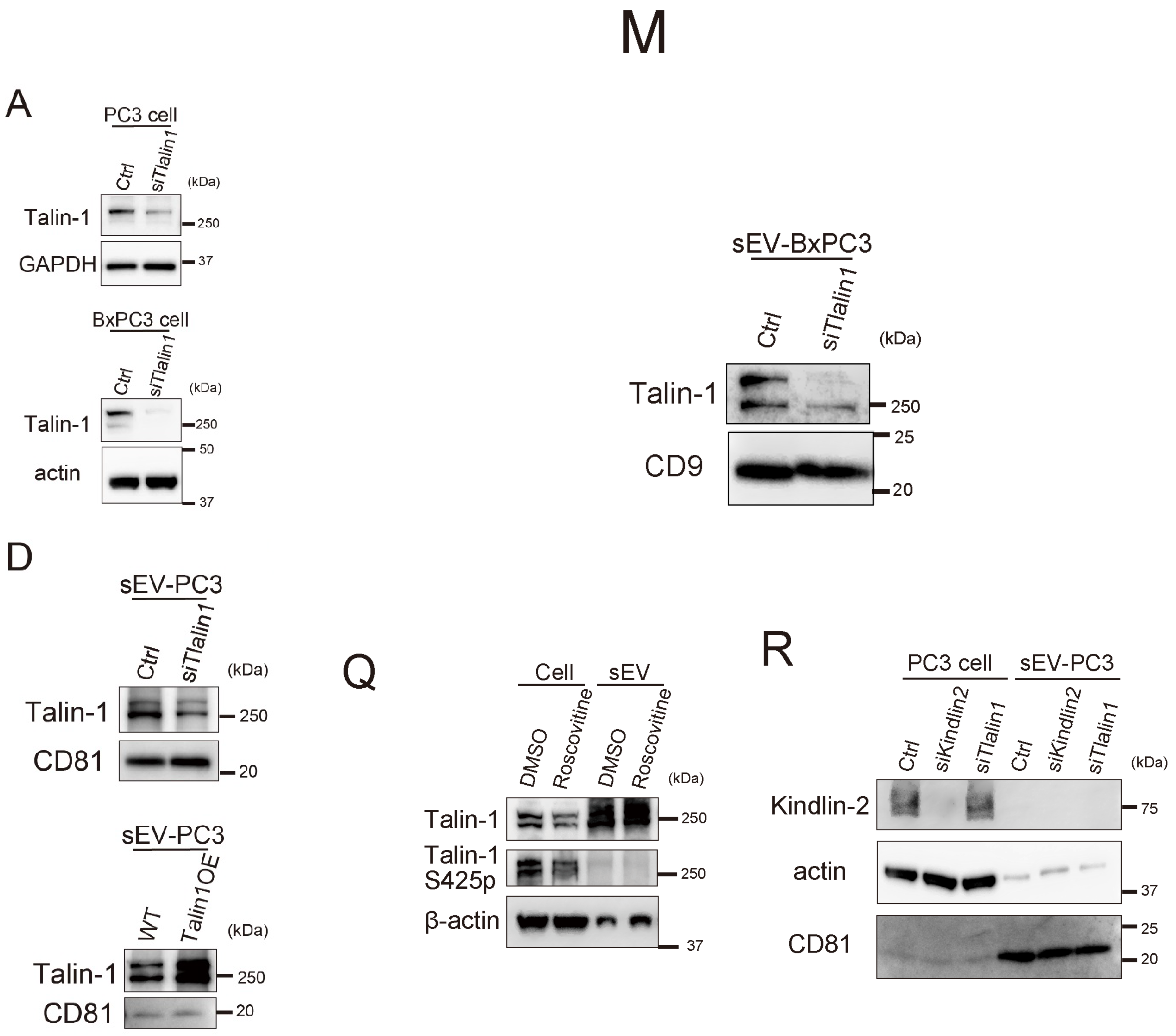

SourceDataF7A, 7D_PC3 cell/PC3-sEV_Talin1

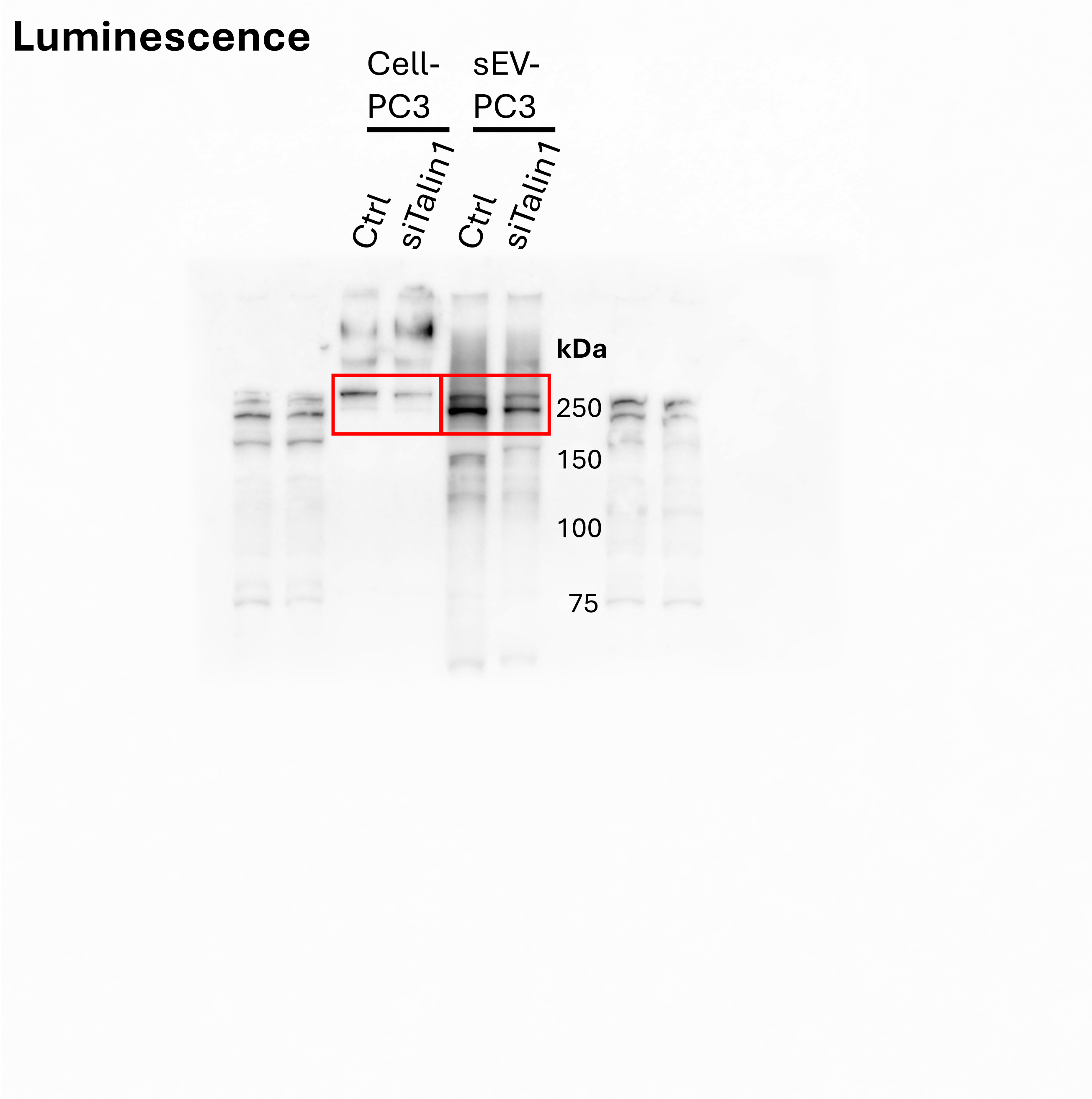

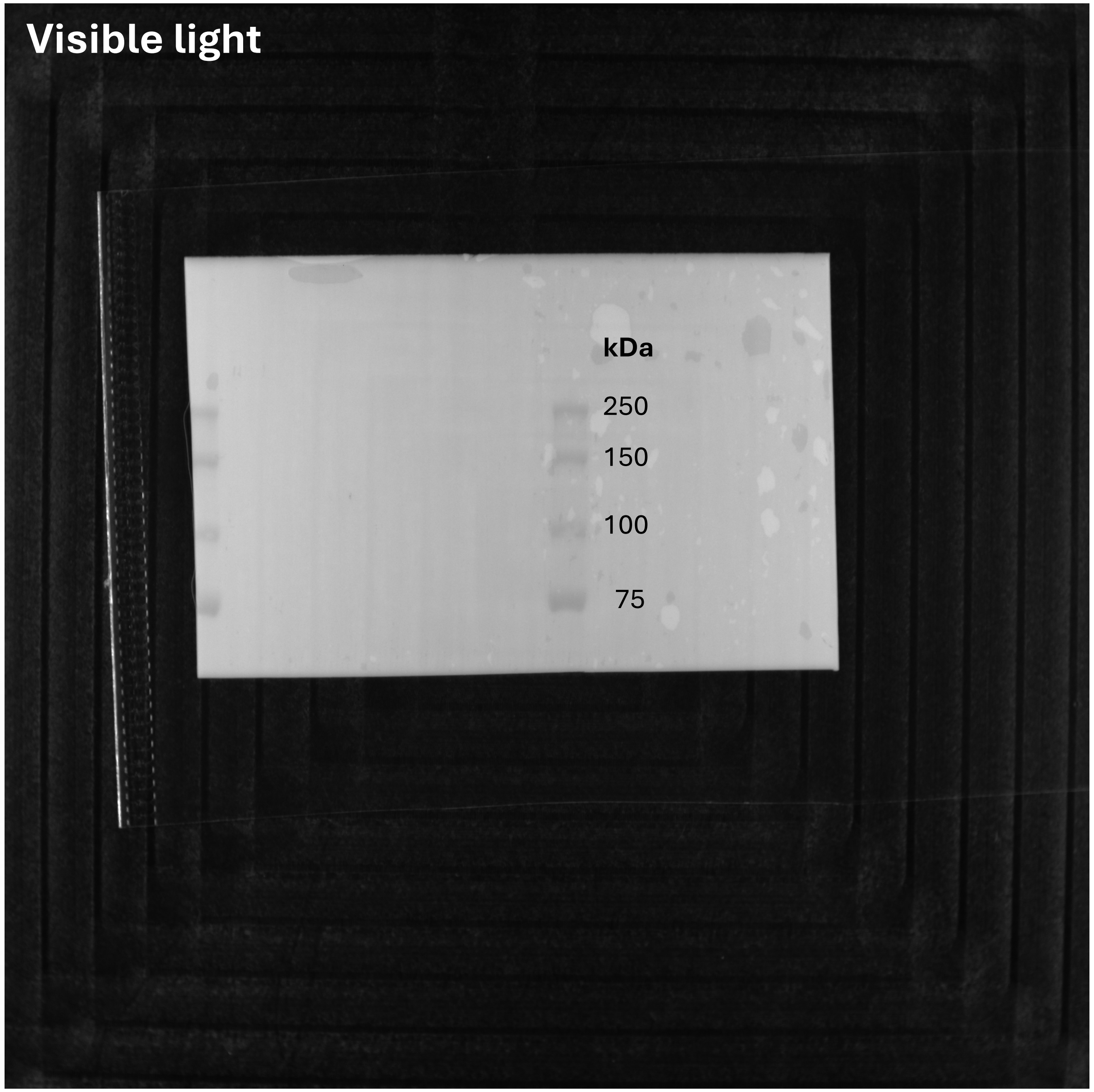

SourceDataF7A_PC3 cell_GAPDH

Luminescence

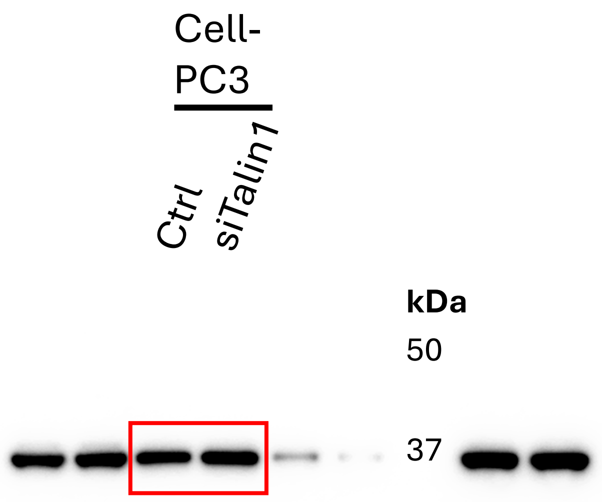

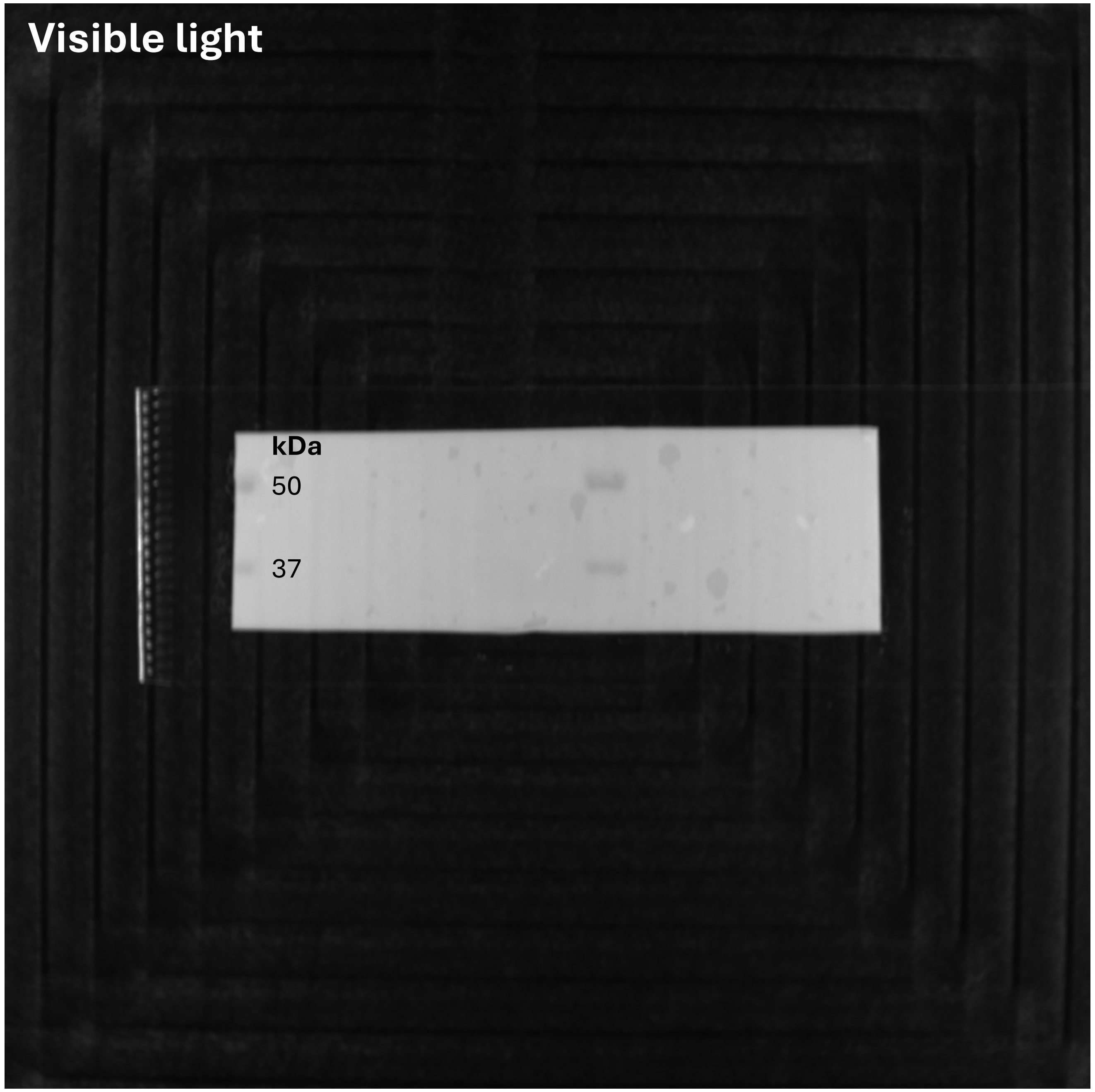

SourceDataF7A_BxPC3 cell_Talin1

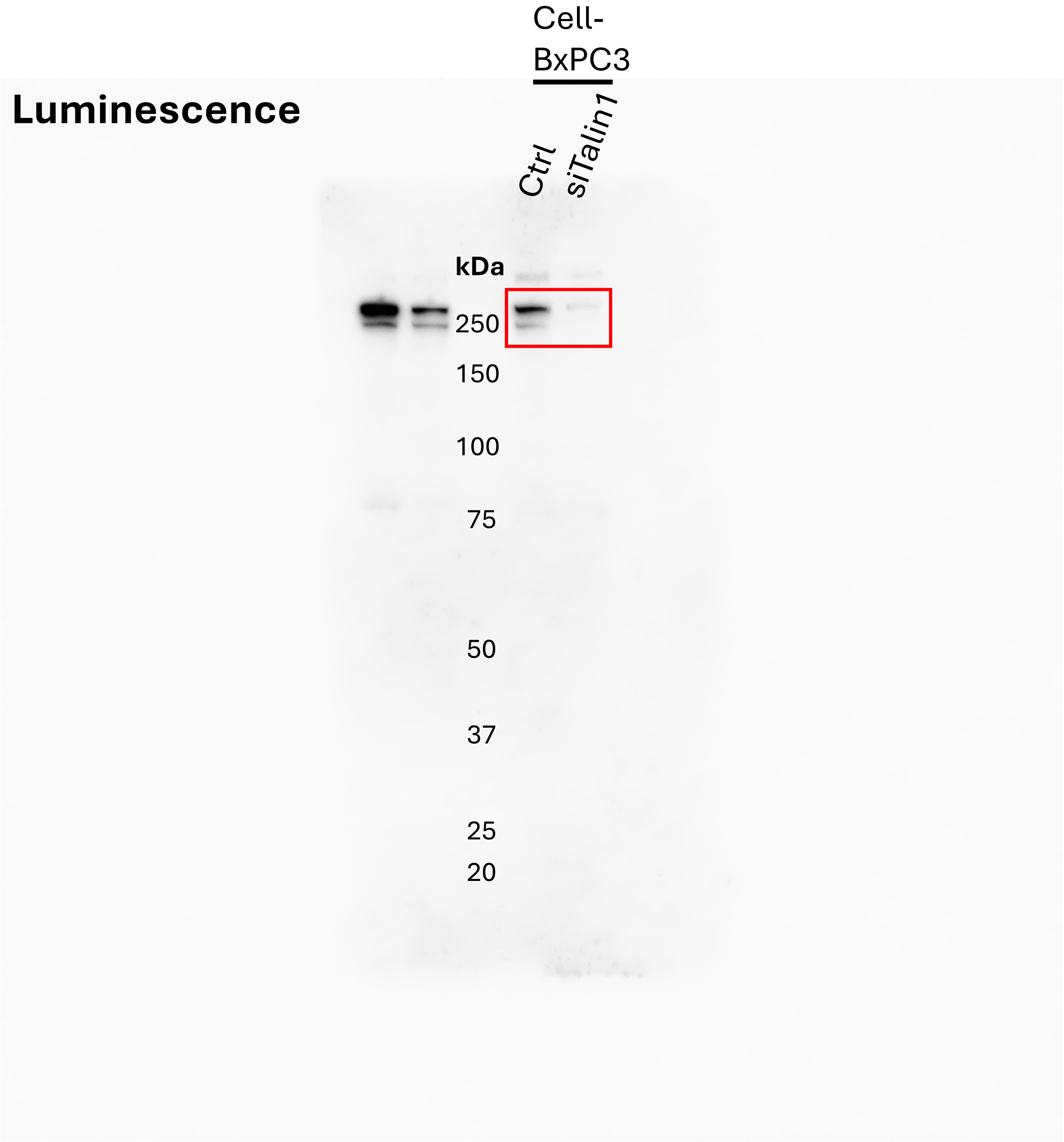

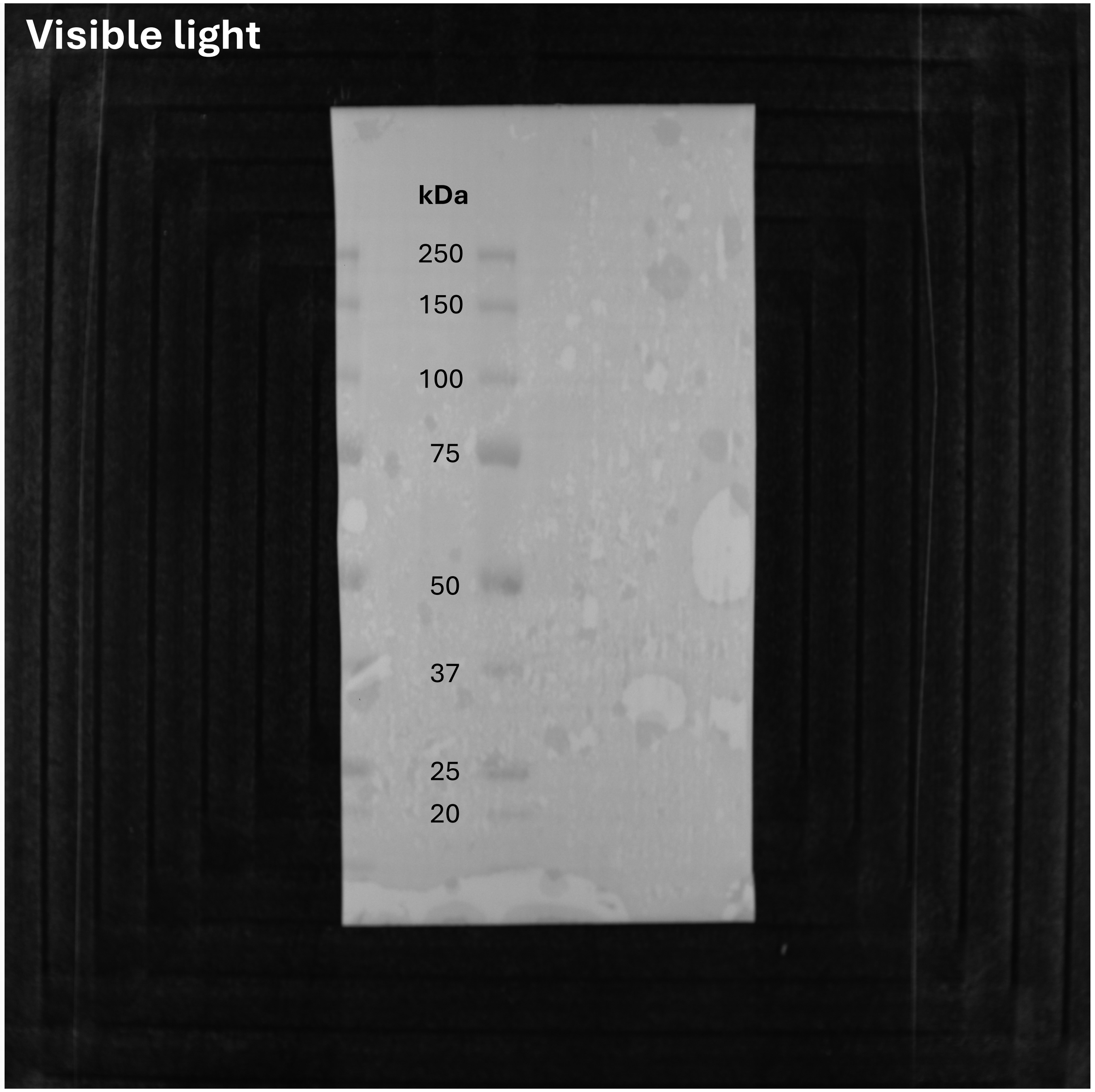

SourceDataF7A_BxPC3 cell_actin

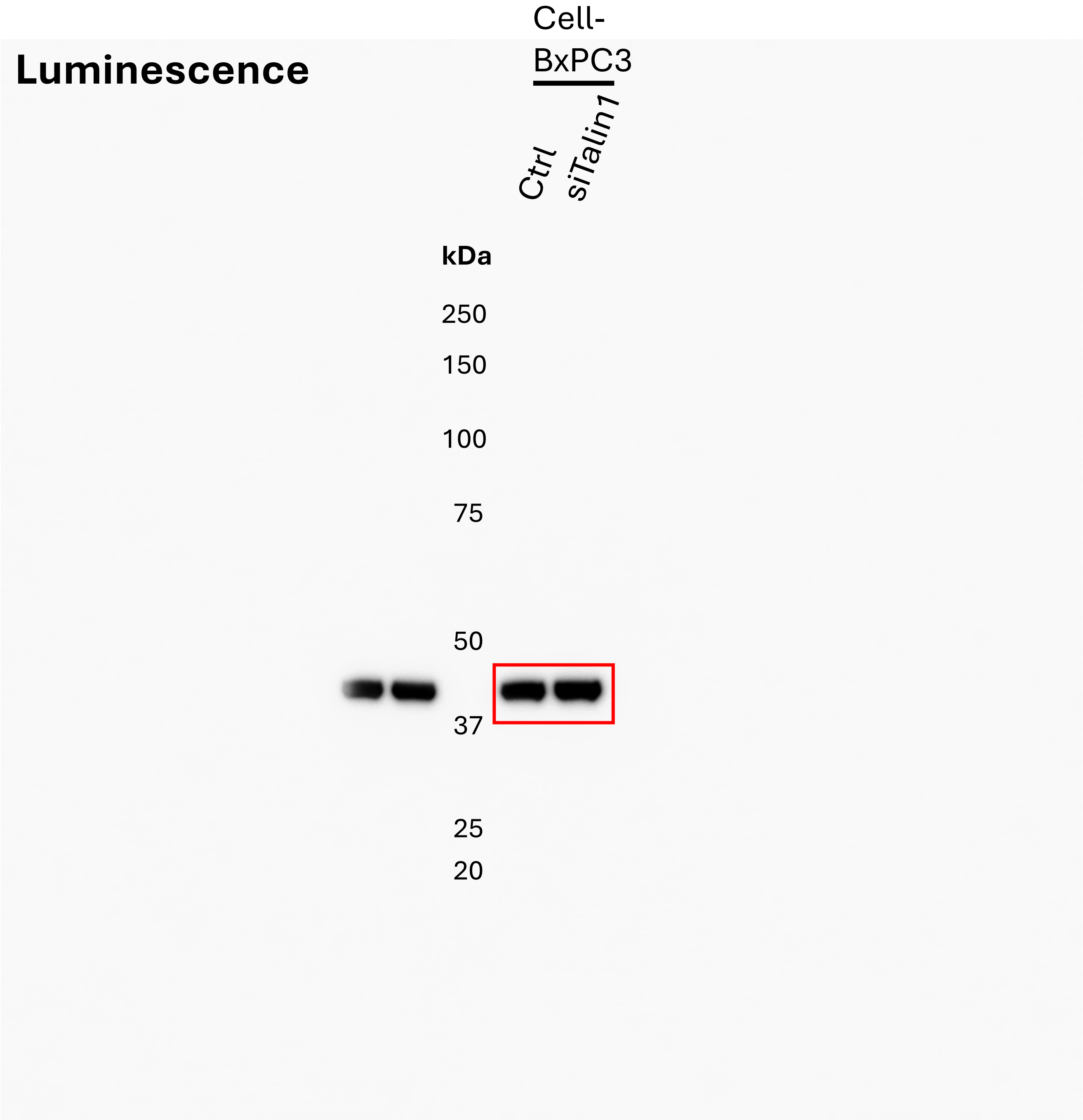

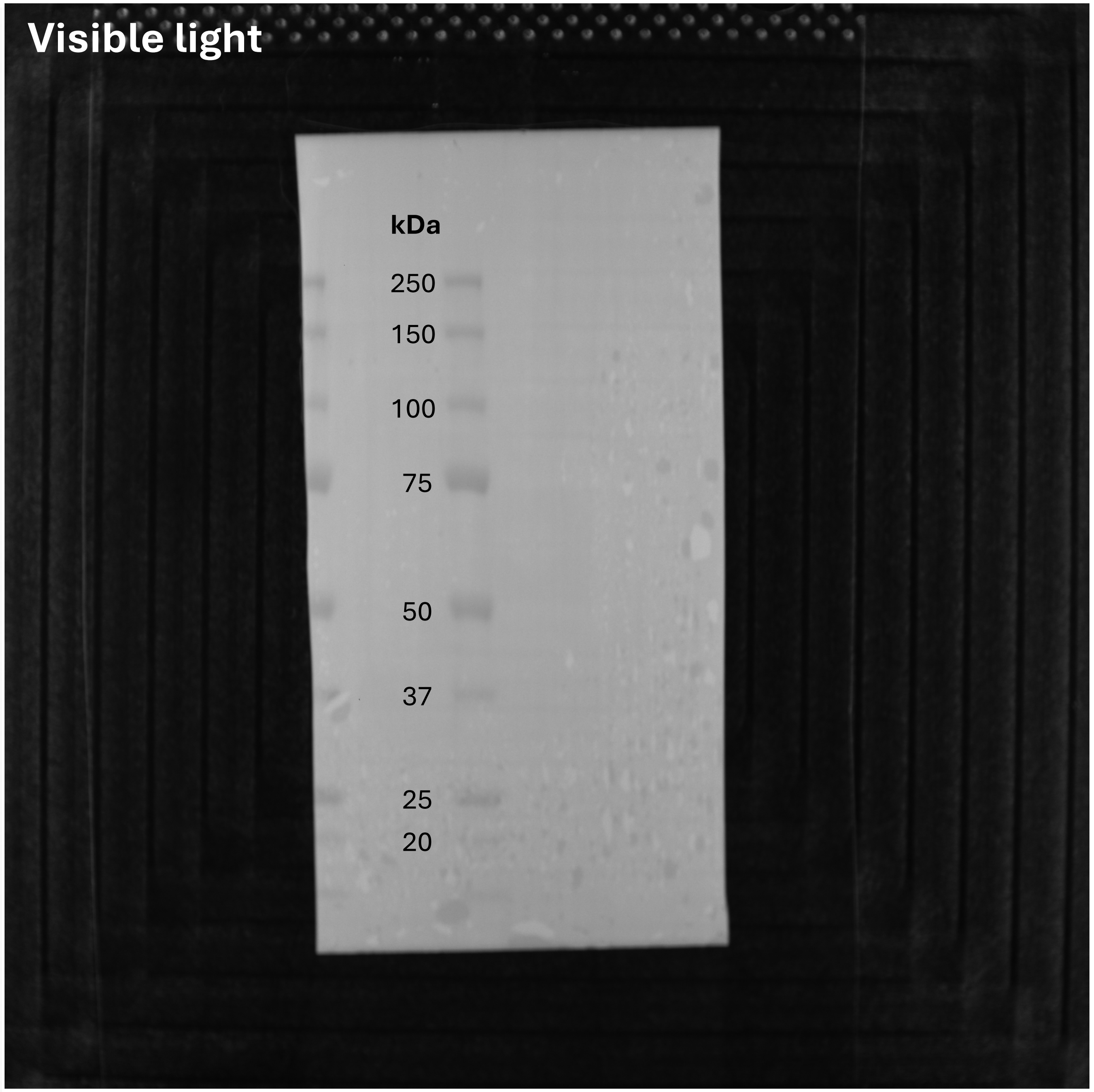

SourceDataF7D_PC3-siTalin1-sEV_CD81

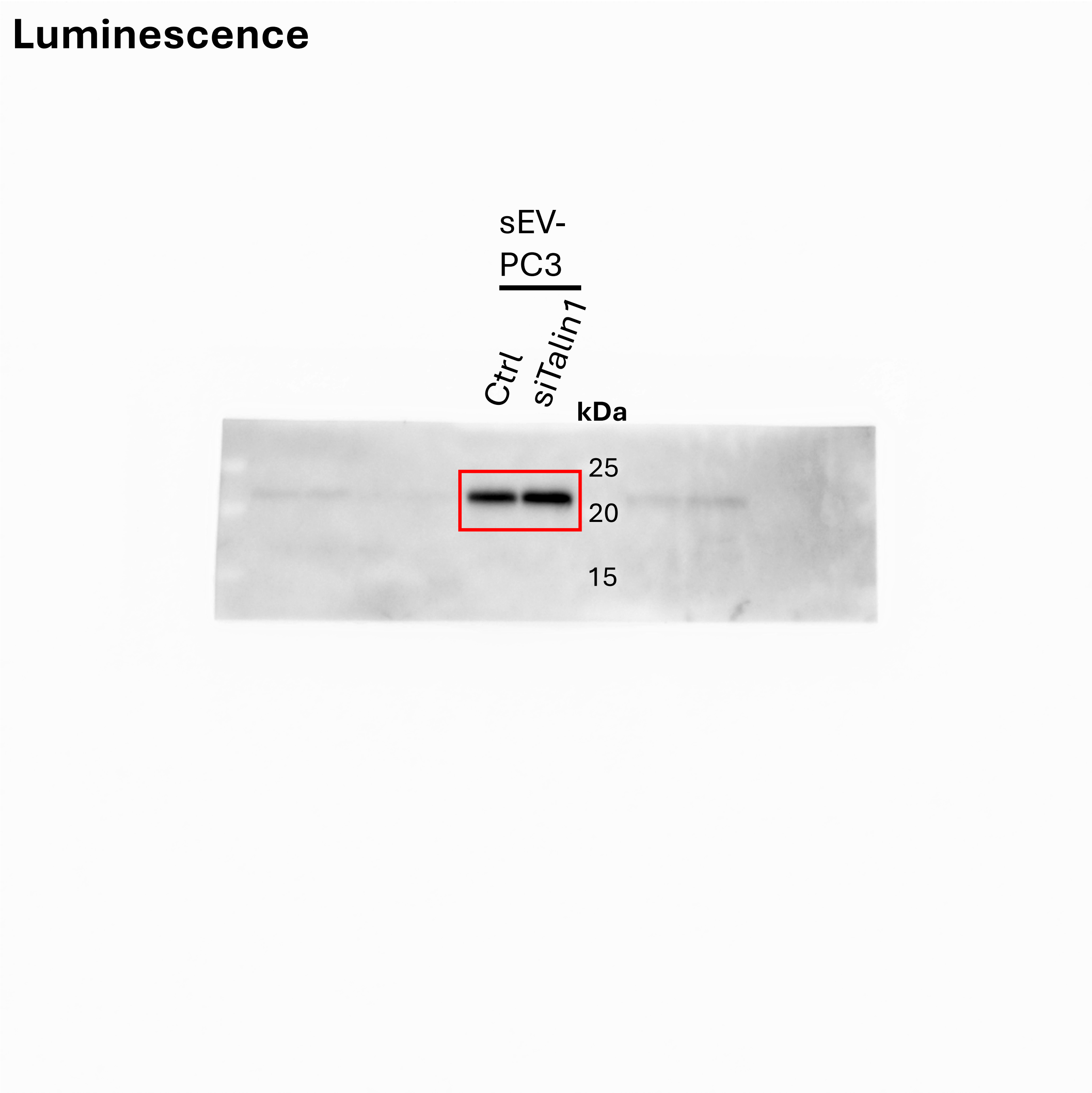

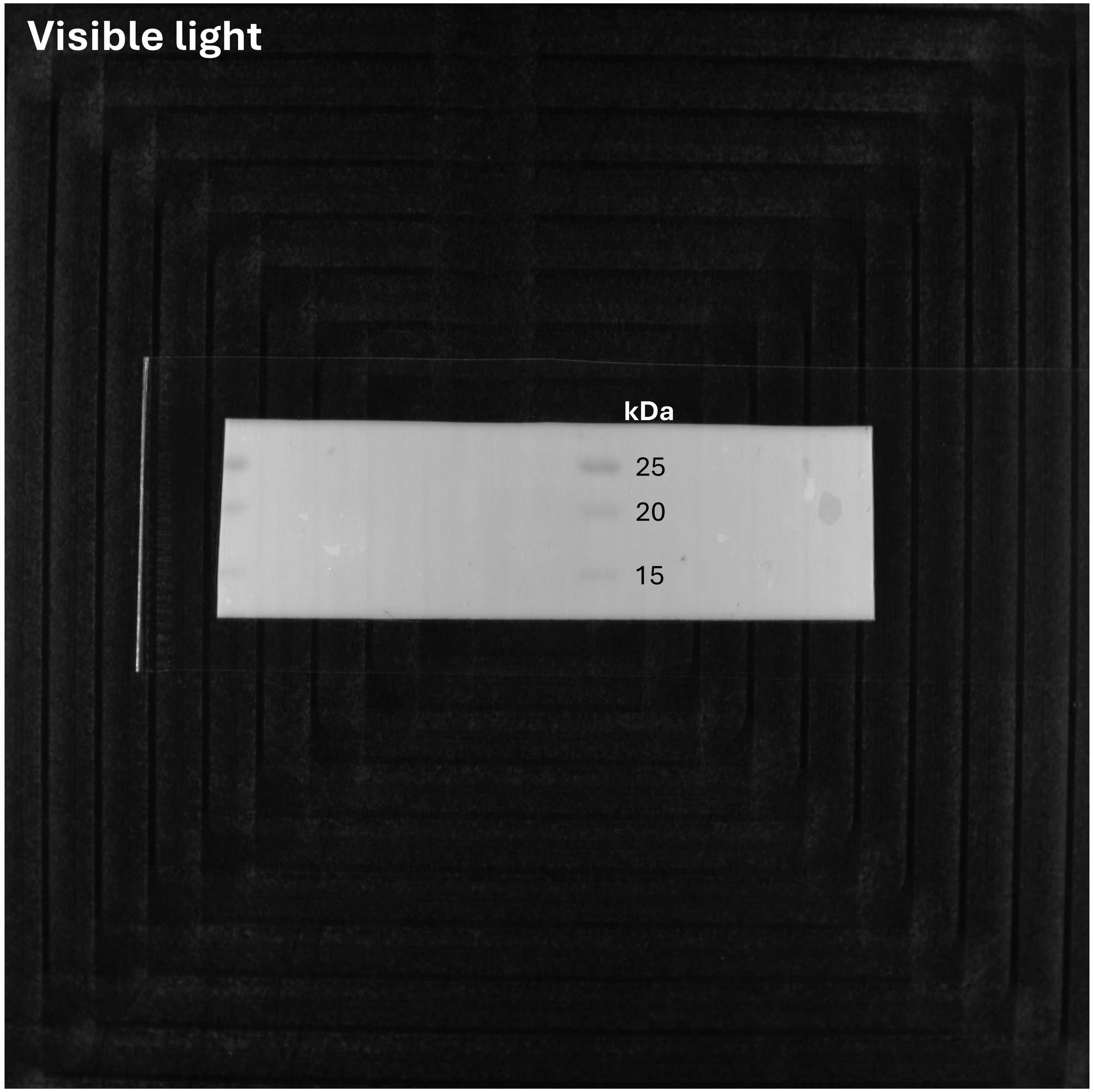

SourceDataF7D_PC3-Talin1OE-sEV_Talin1

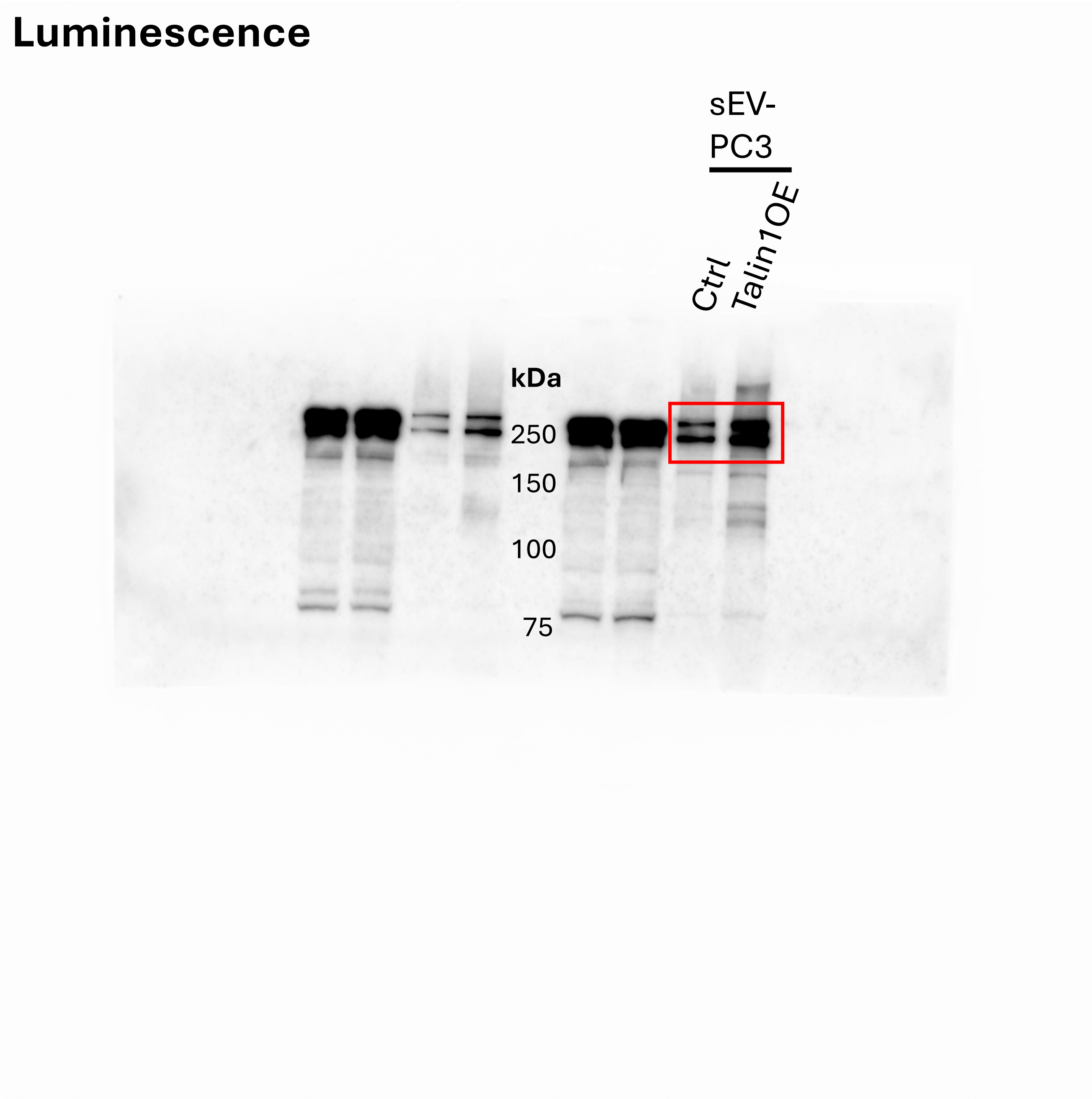

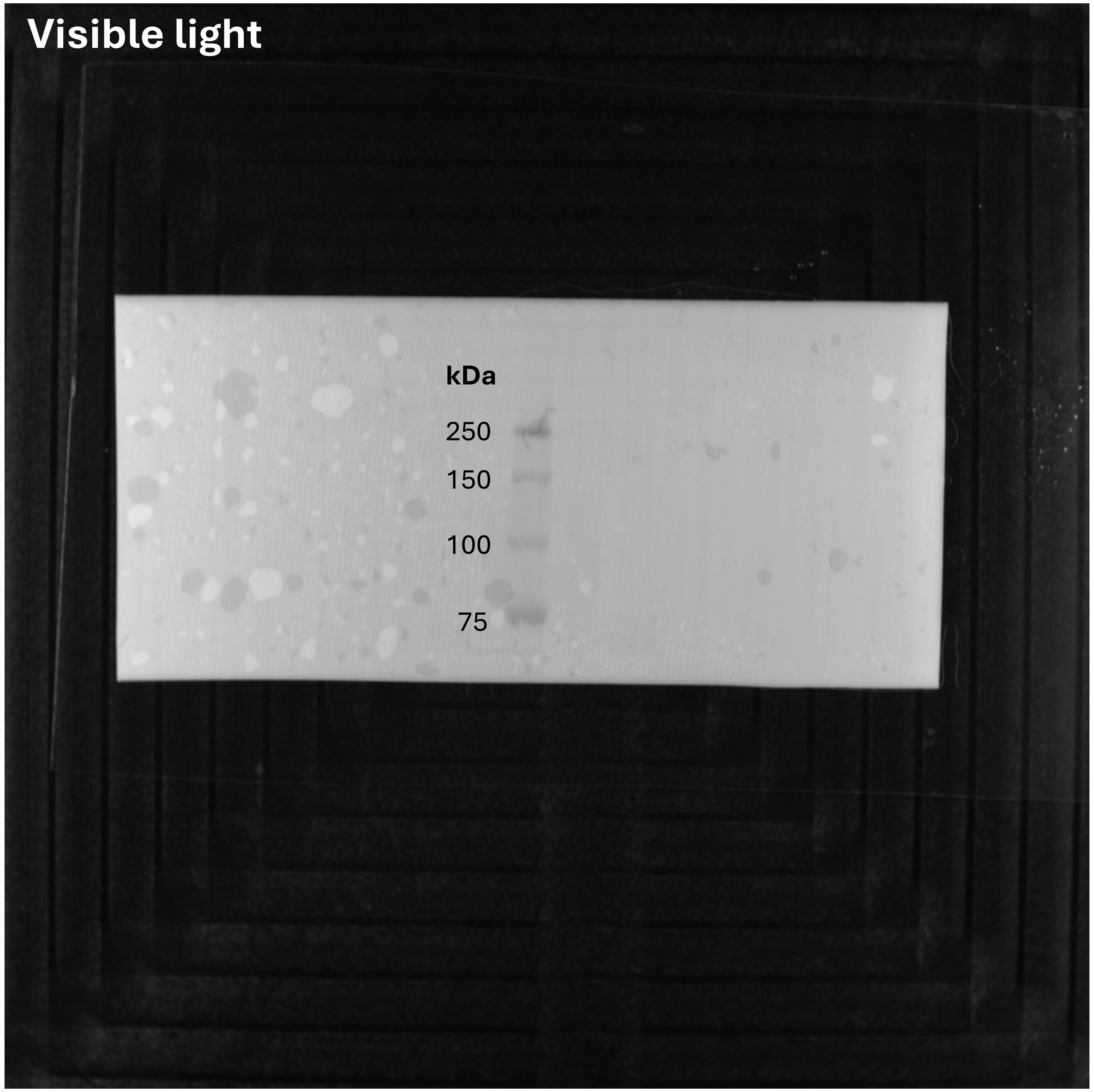

SourceDataF7D_PC3-Talin1OE-sEV_CD81

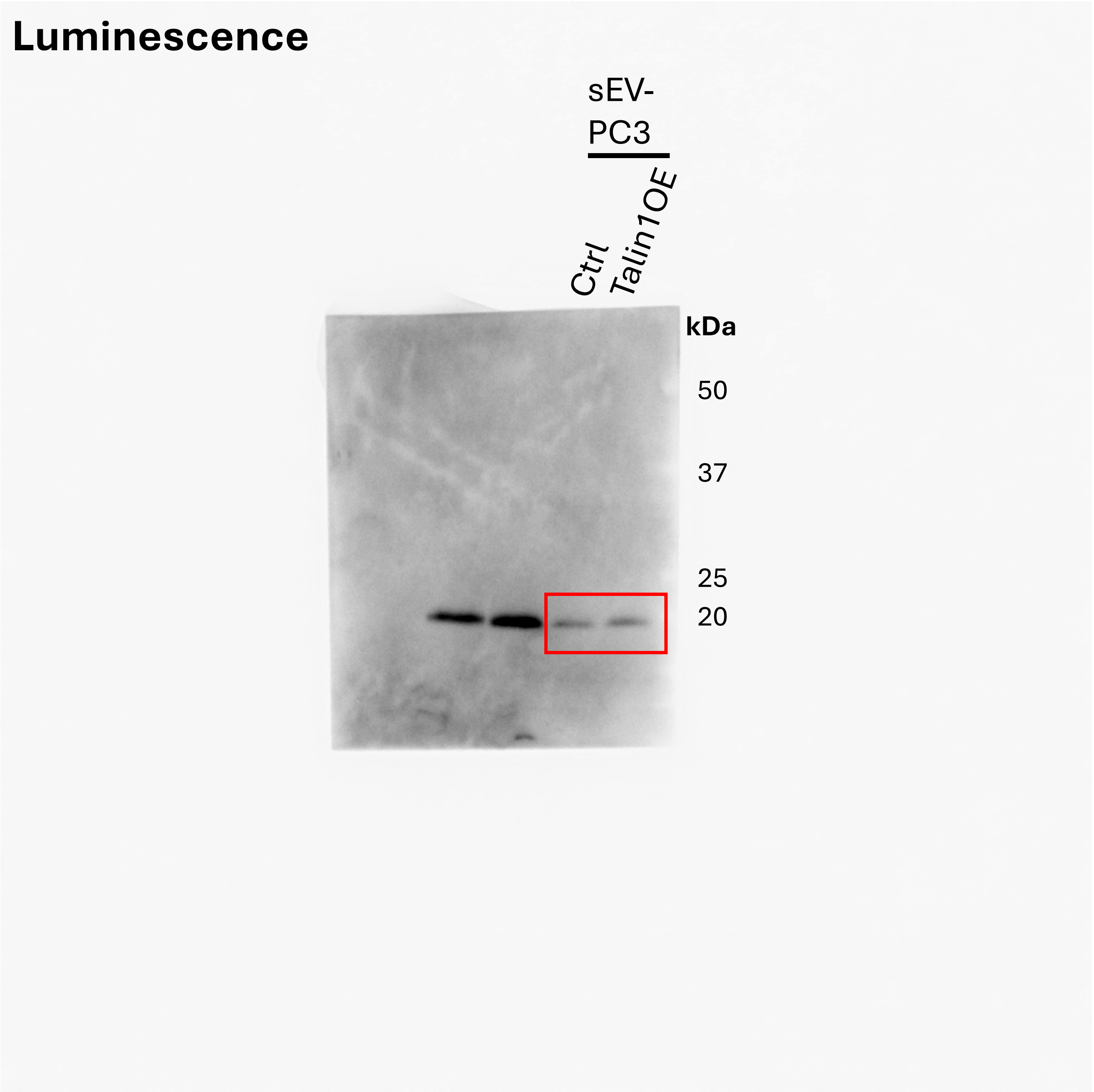

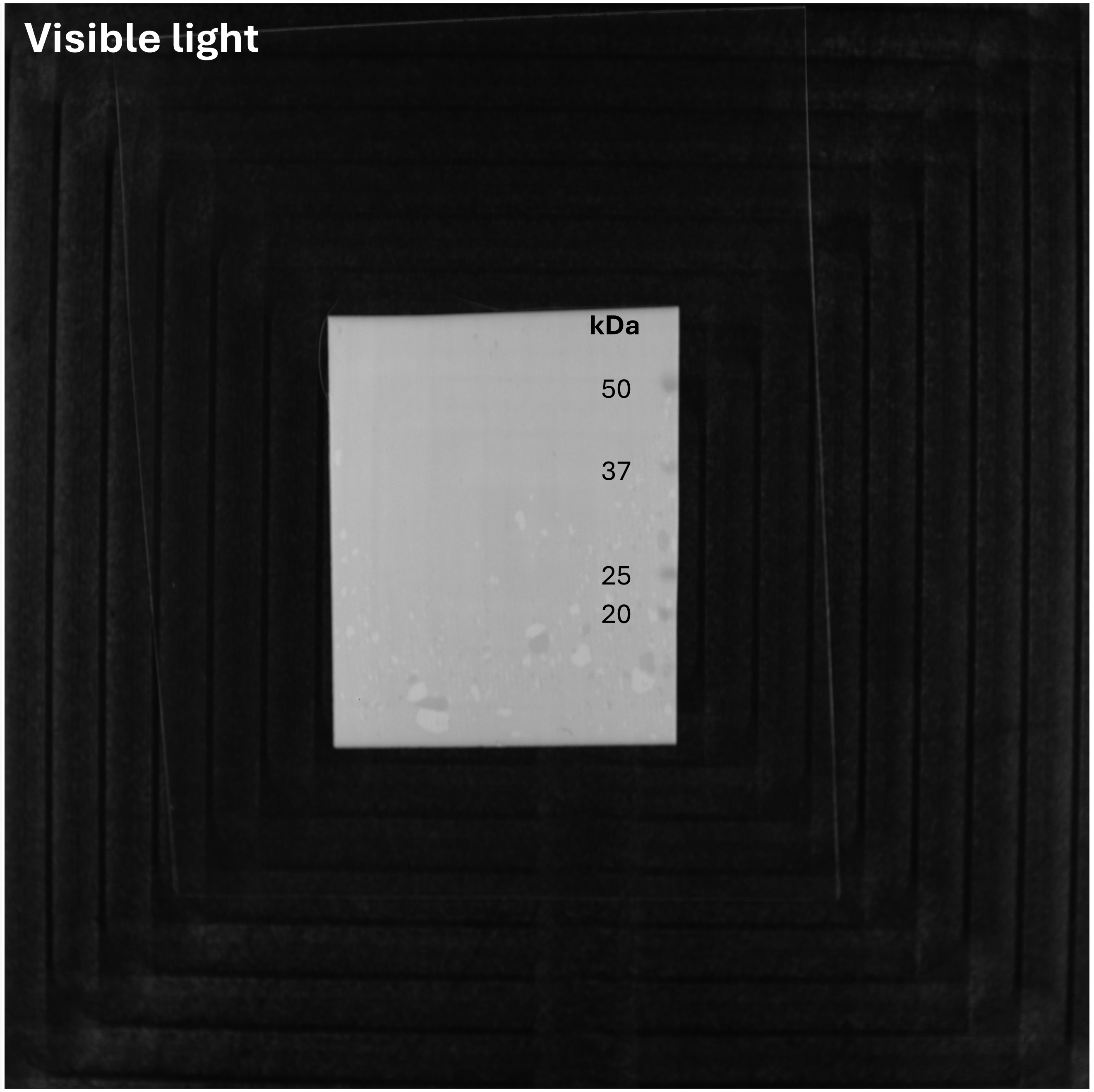

SourceDataF7M_sEV-BxPC3_Talin1

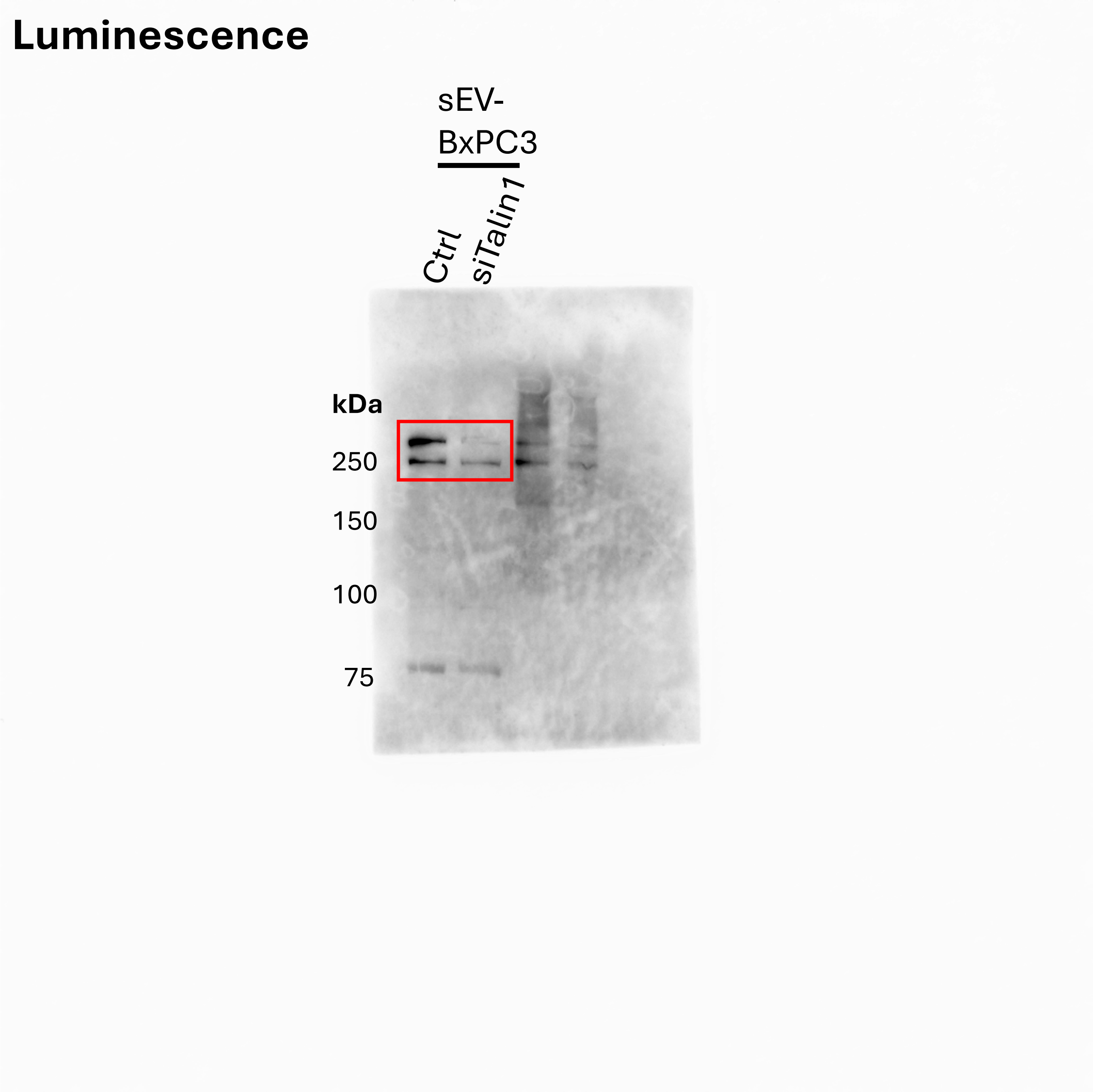

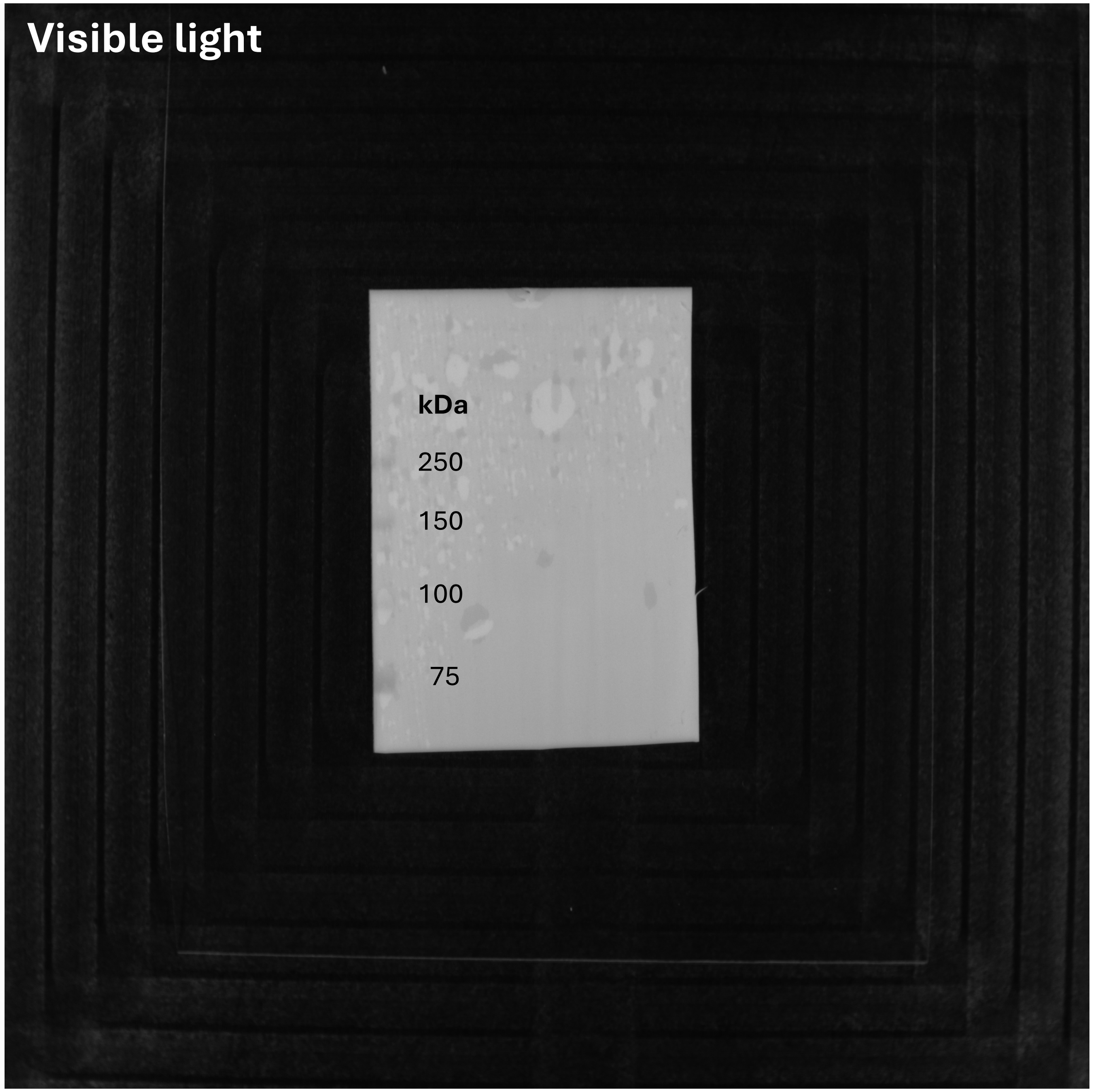

SourceDataF7M_sEV-BxPC3_CD9

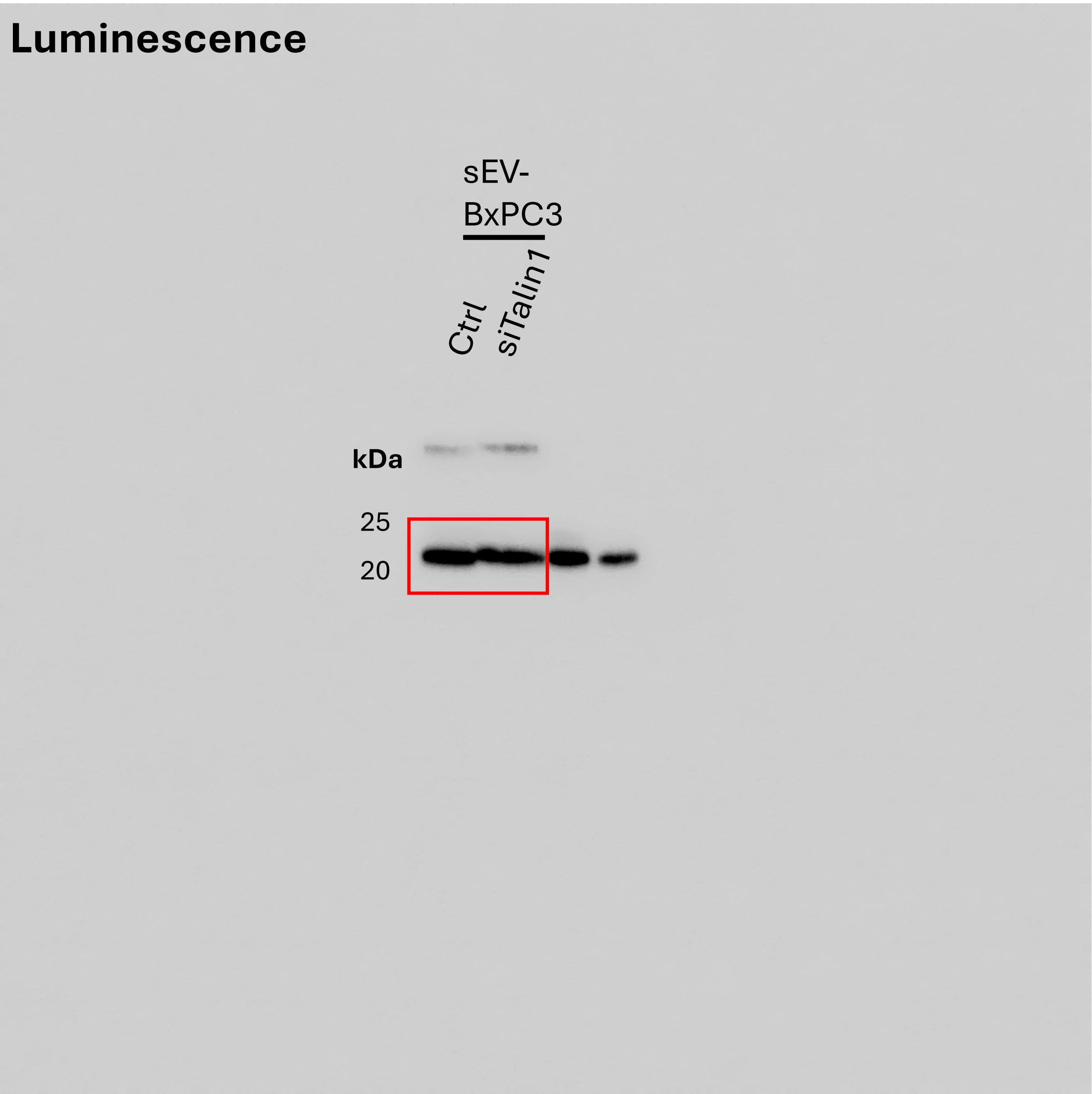

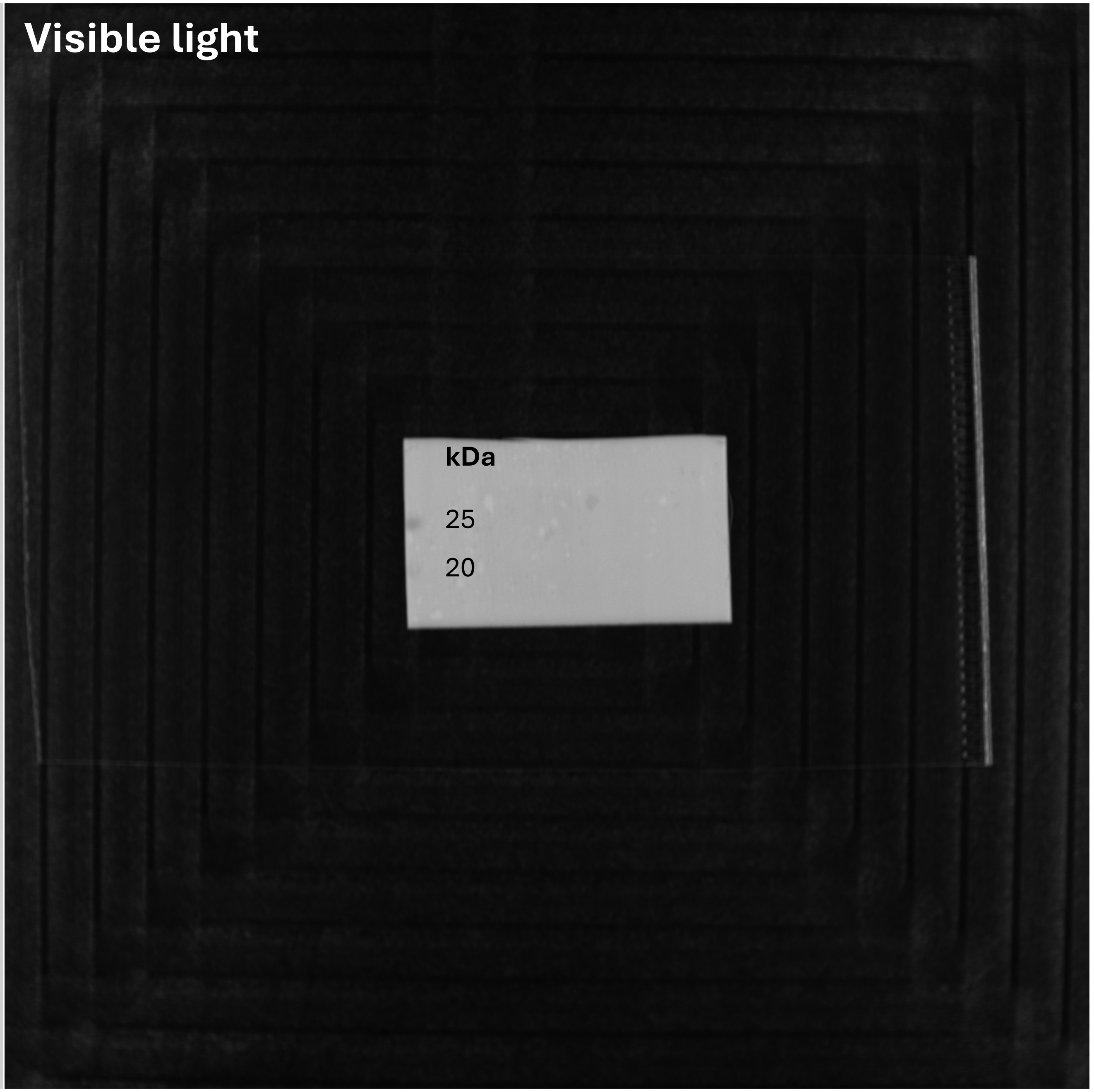

SourceDataF7Q_PC3 cell/sEV-PC3_Talin1

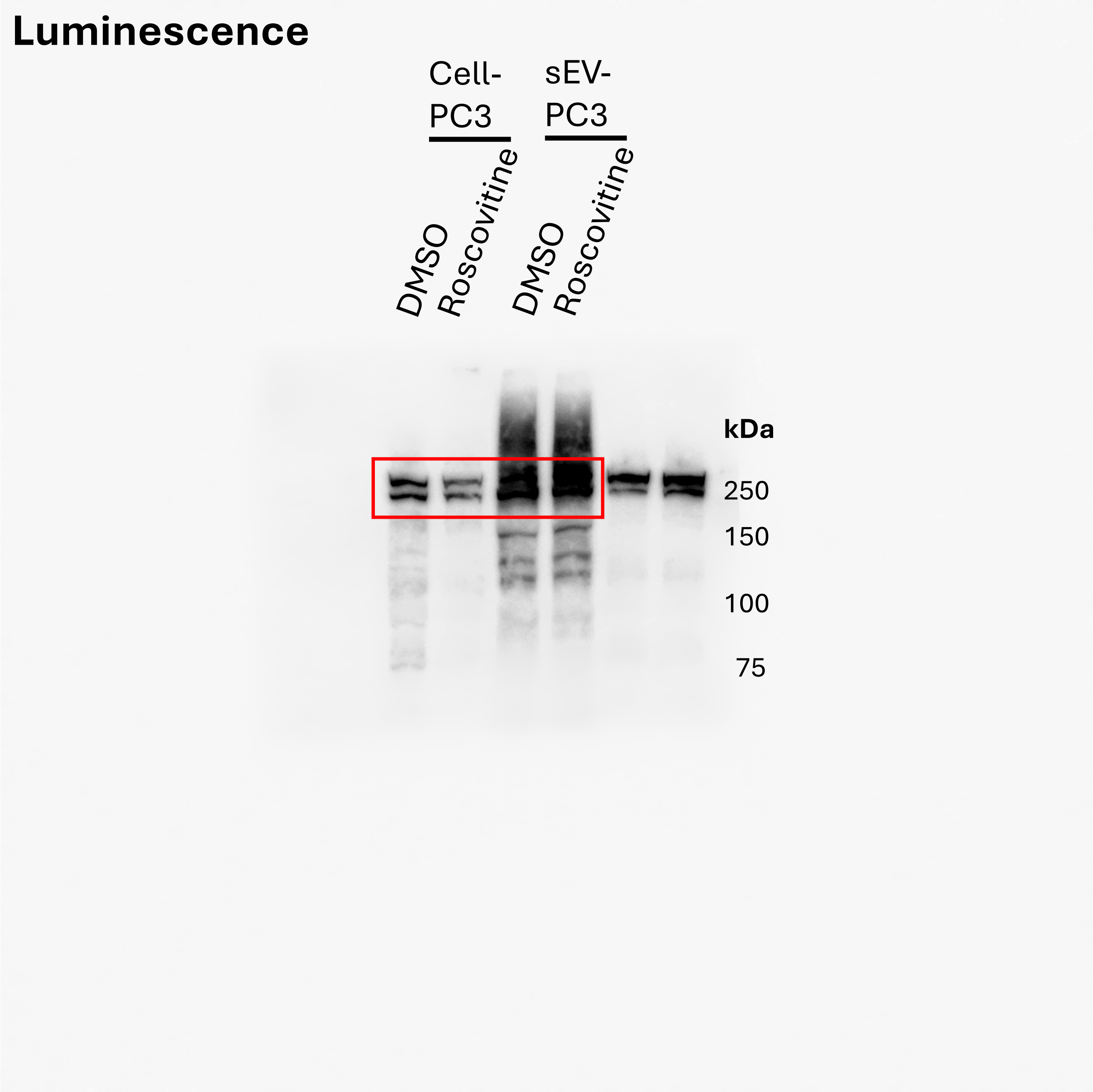

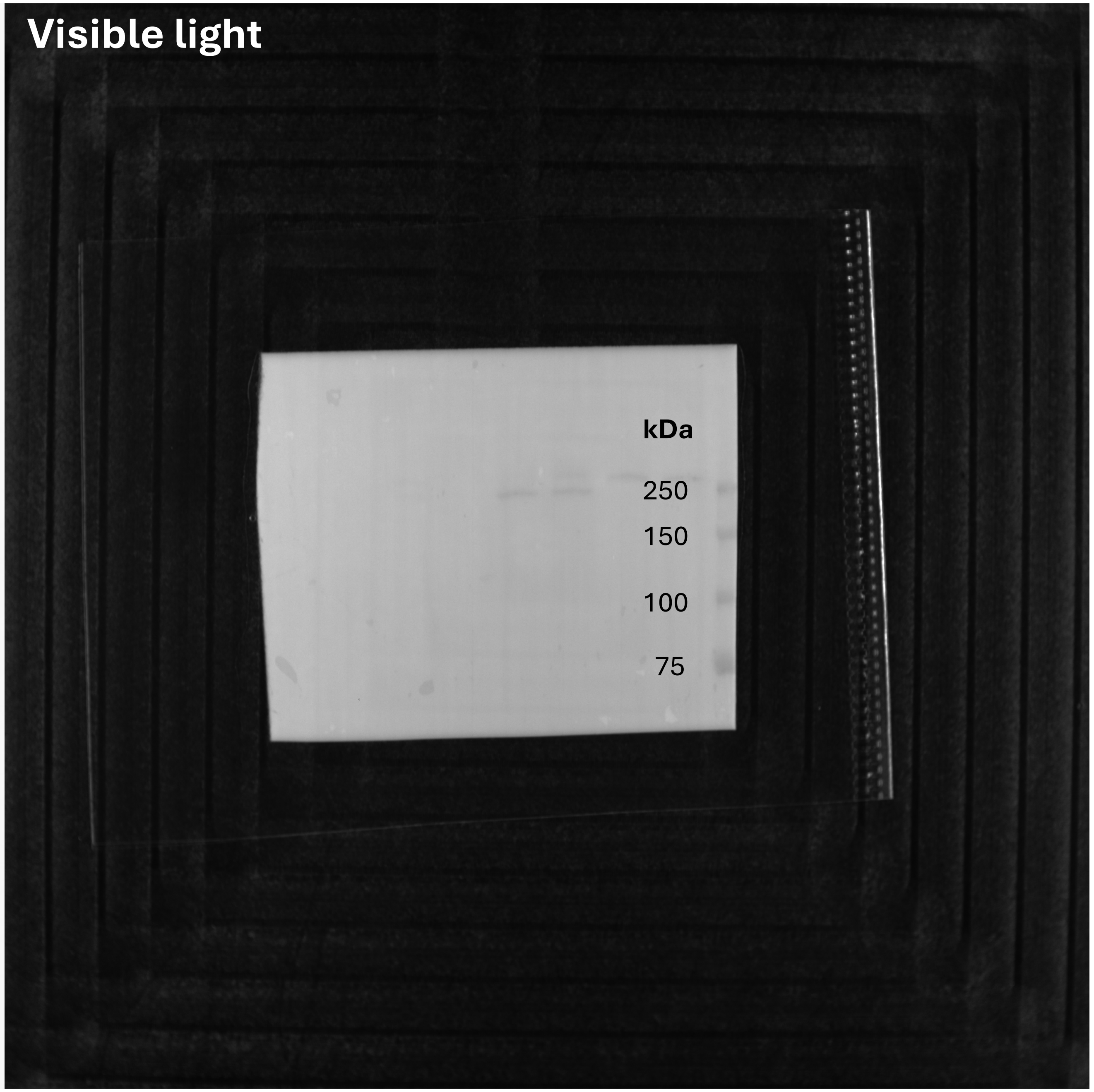

SourceDataF7Q_PC3 cell/sEV-PC3_Talin1 Ser425phospho

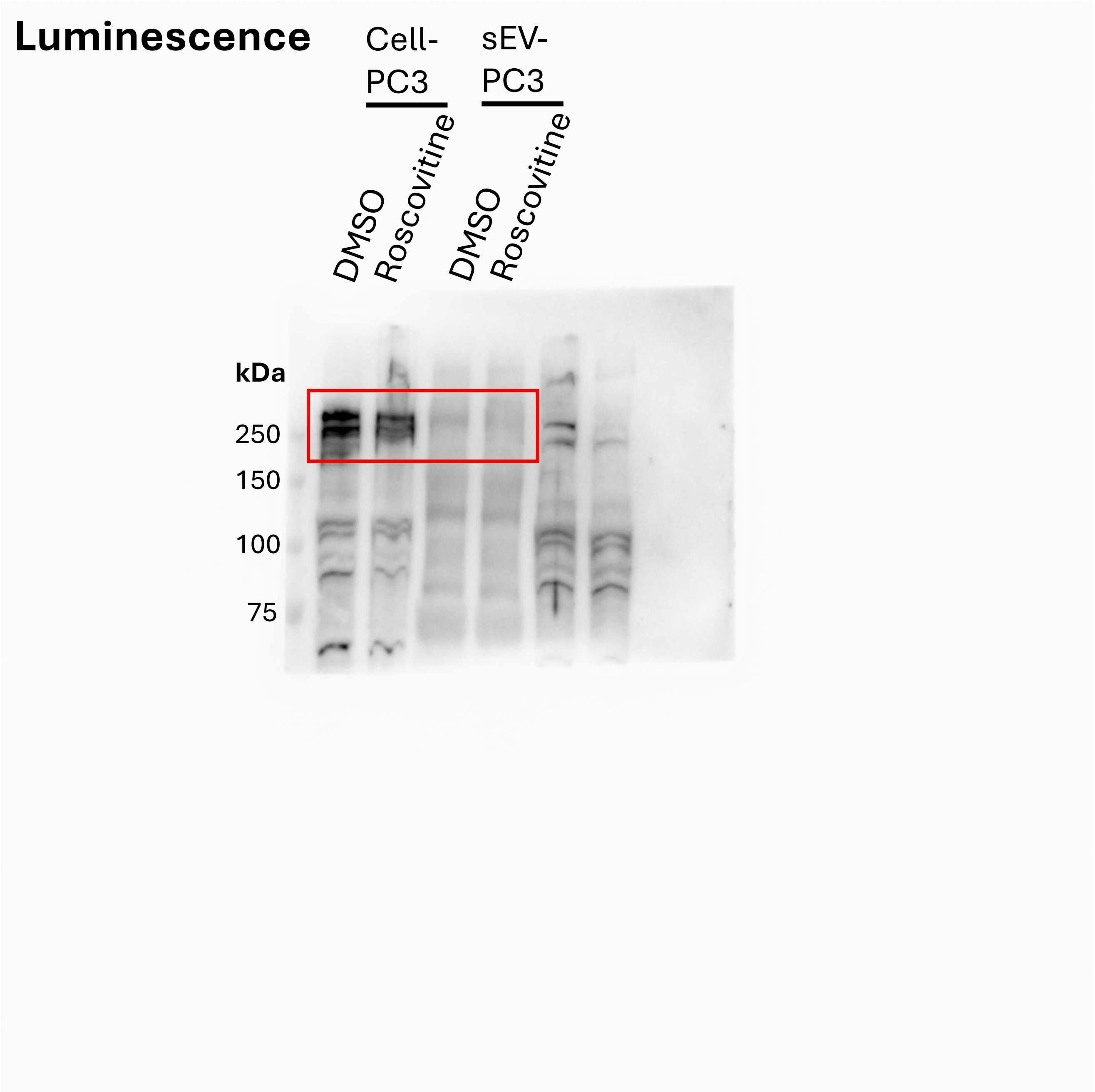

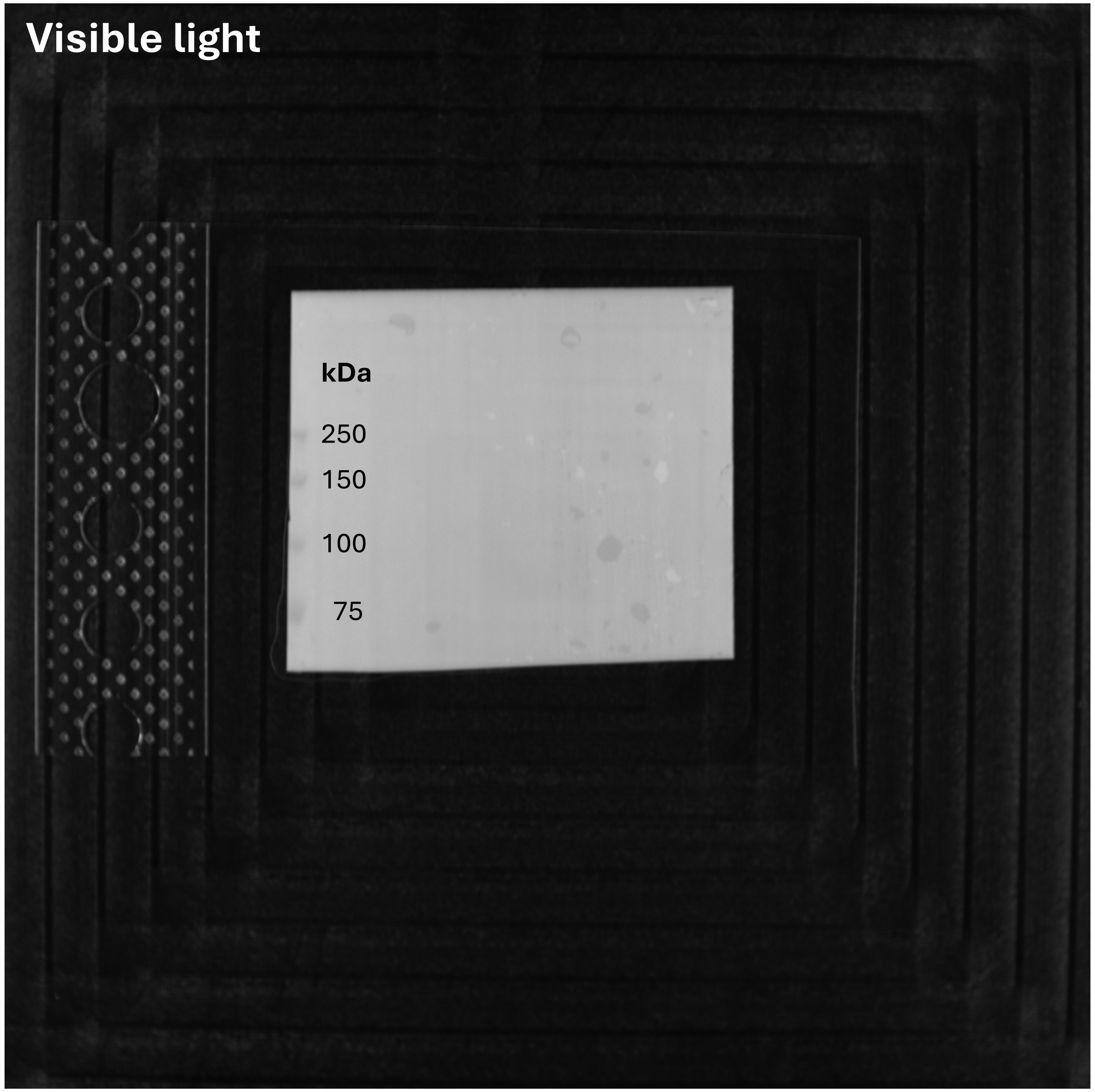

SourceDataF7Q_PC3 cell/sEV-PC3_actin

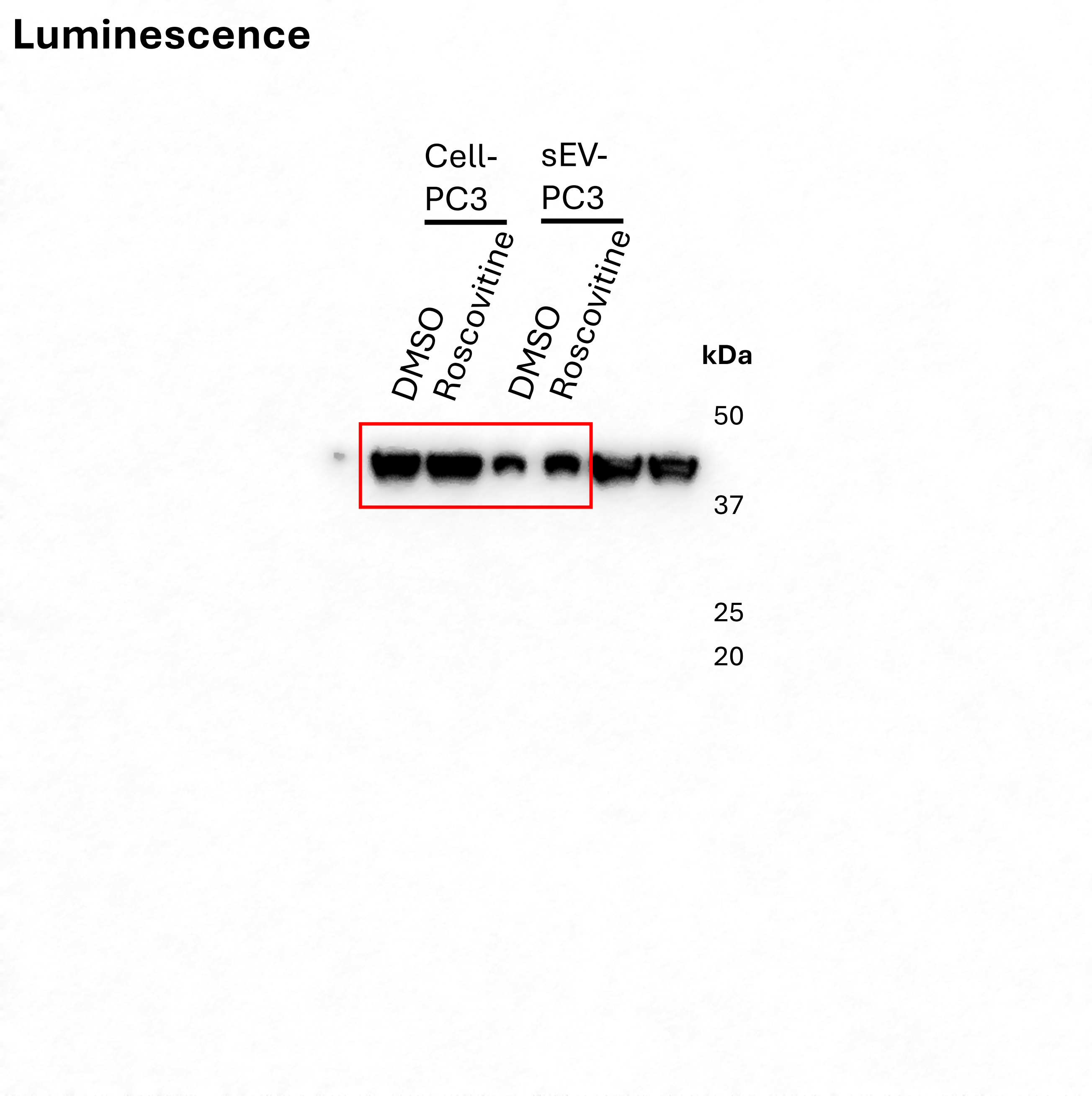

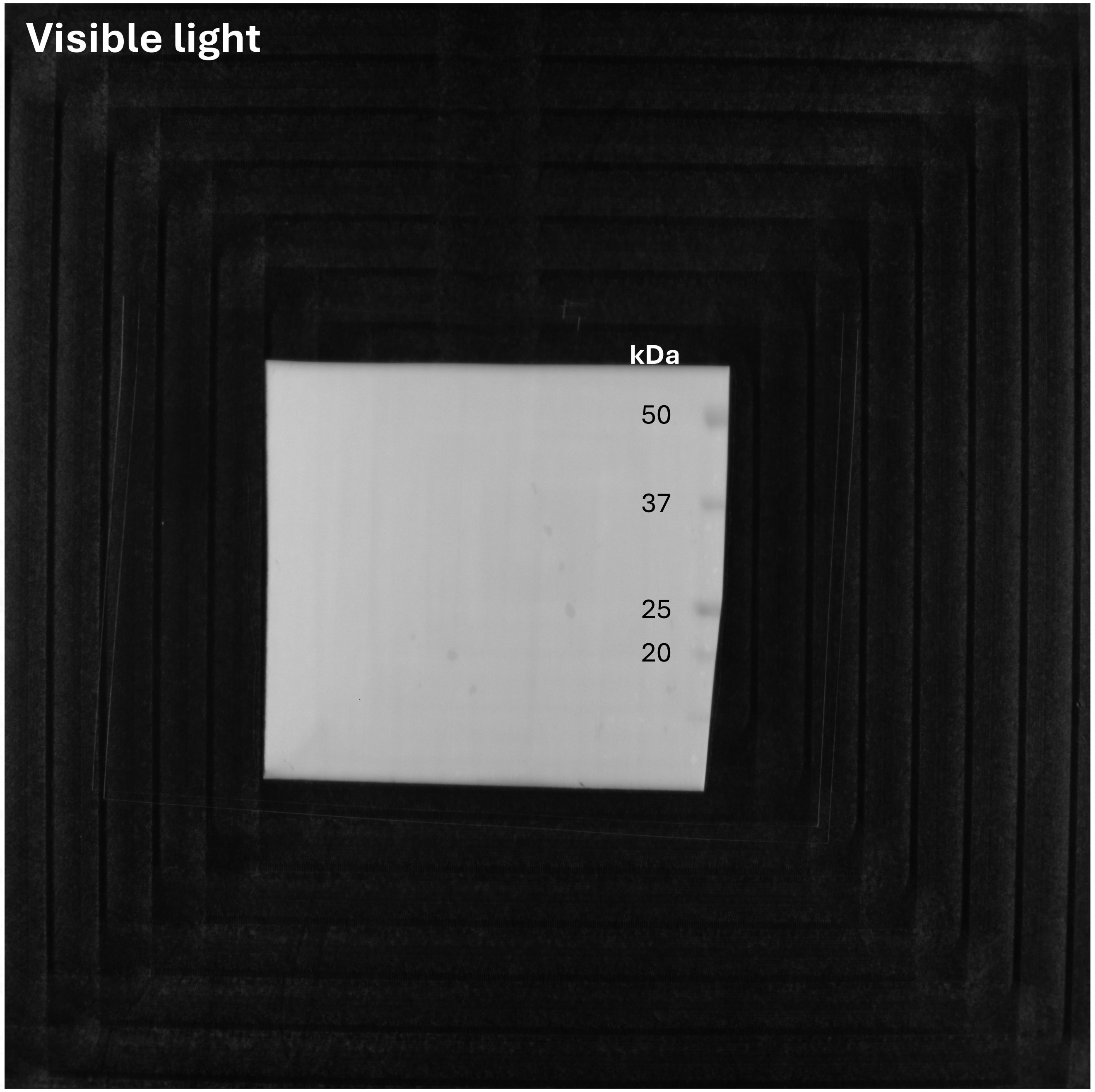

SourceDataF7R_PC3 cell/sEV-PC3_Kindlin-2

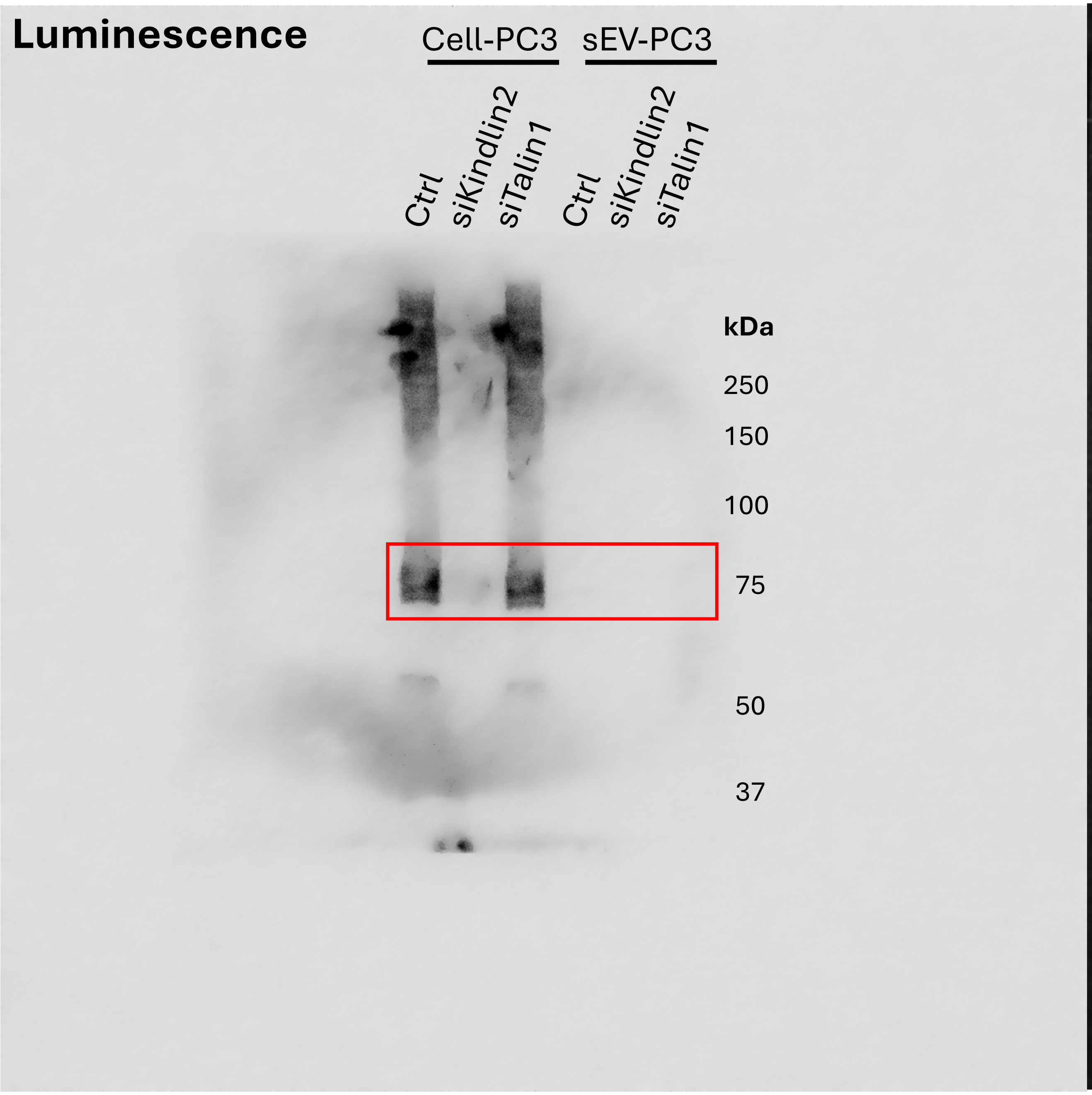

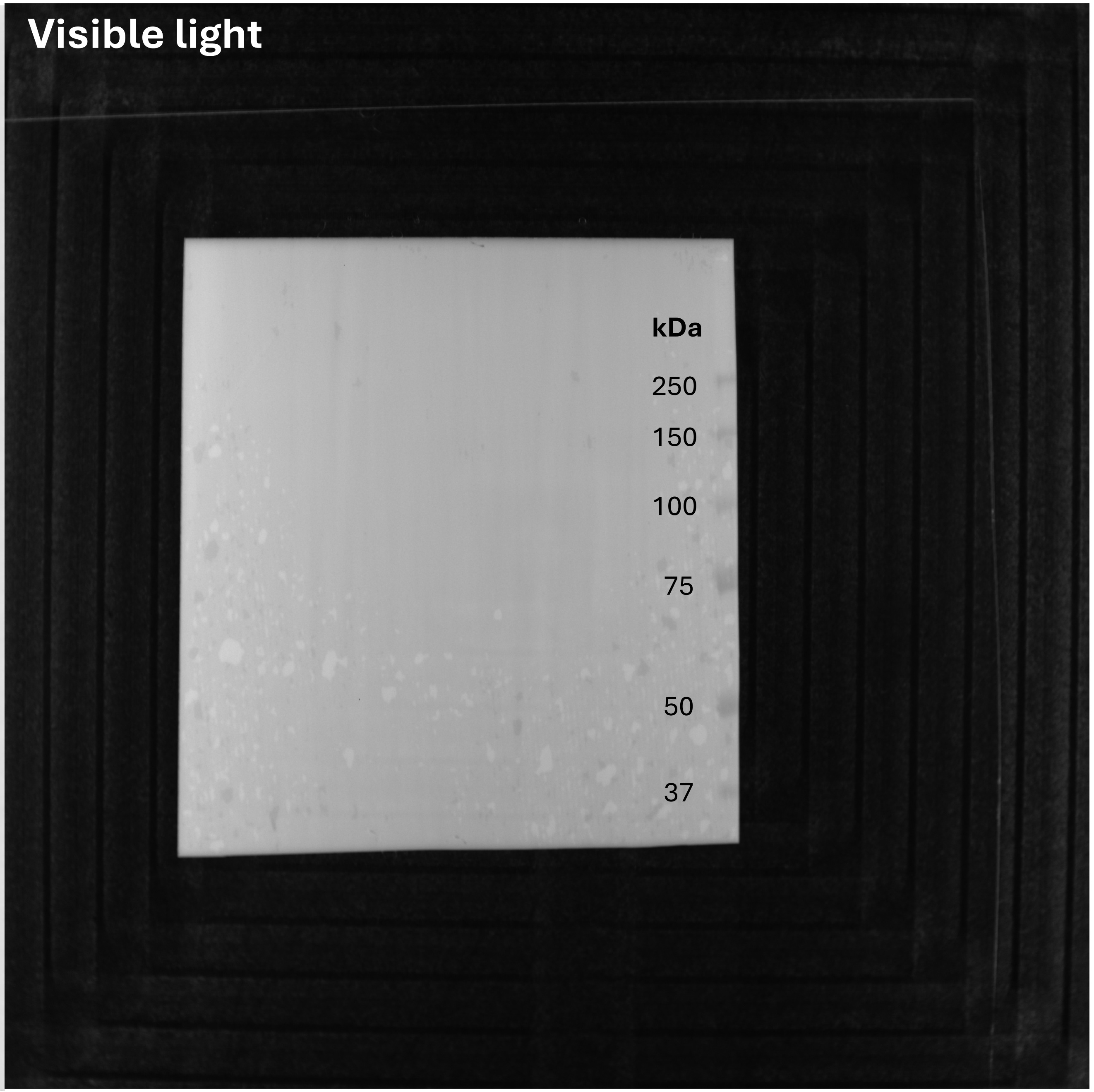

SourceDataF7R_PC3 cell/sEV-PC3_actin

Luminescence

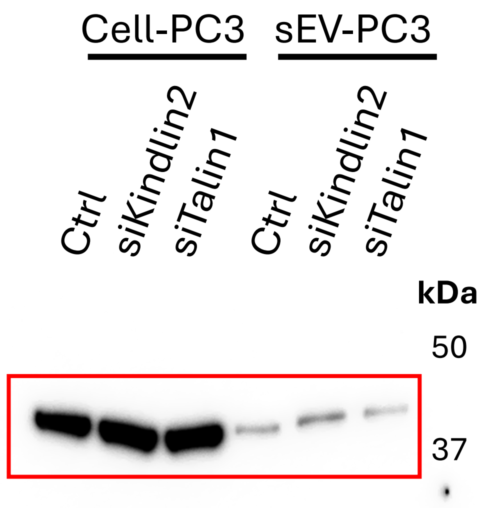

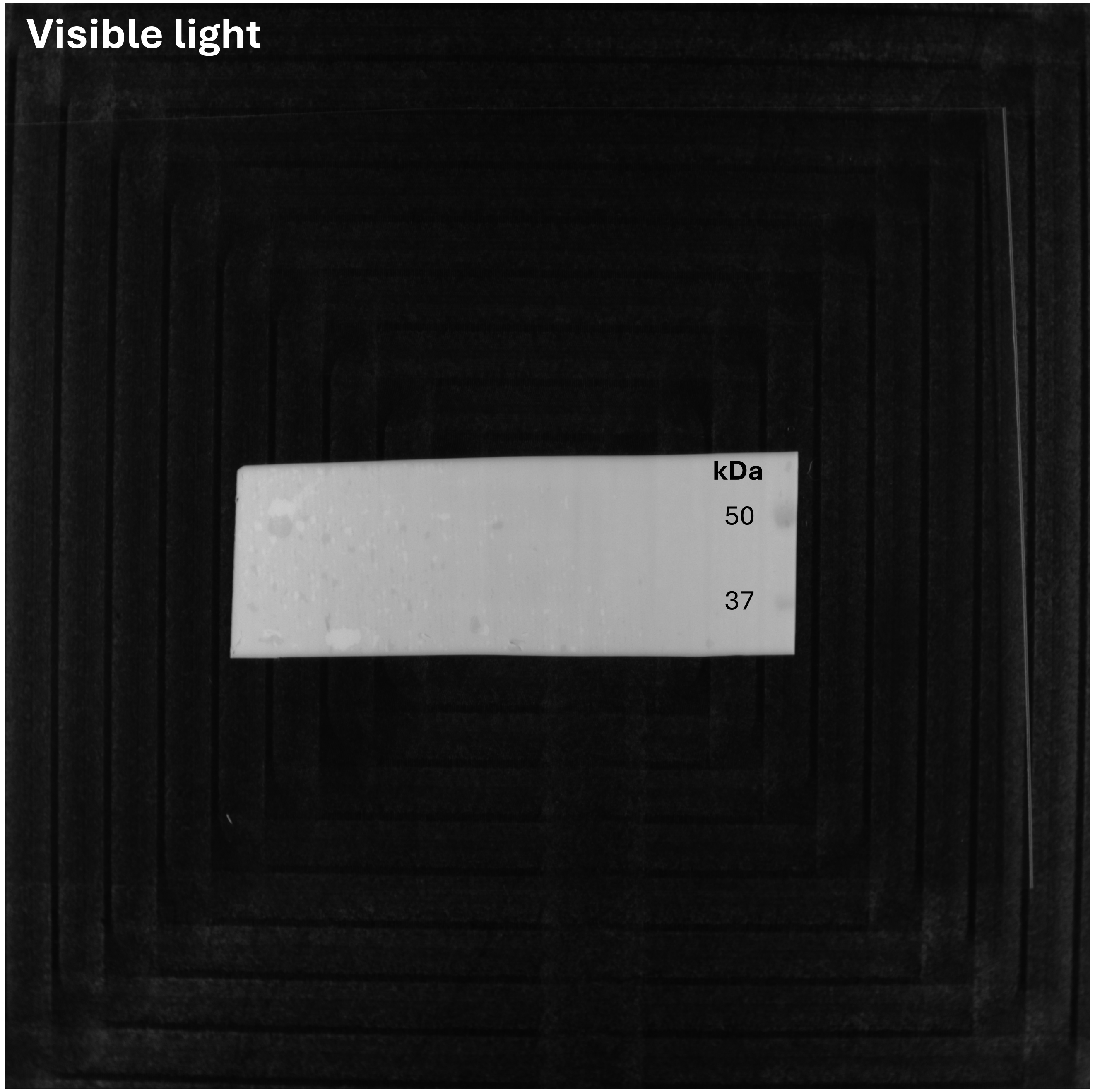

SourceDataF7R_PC3 cell/sEV-PC3_CD81

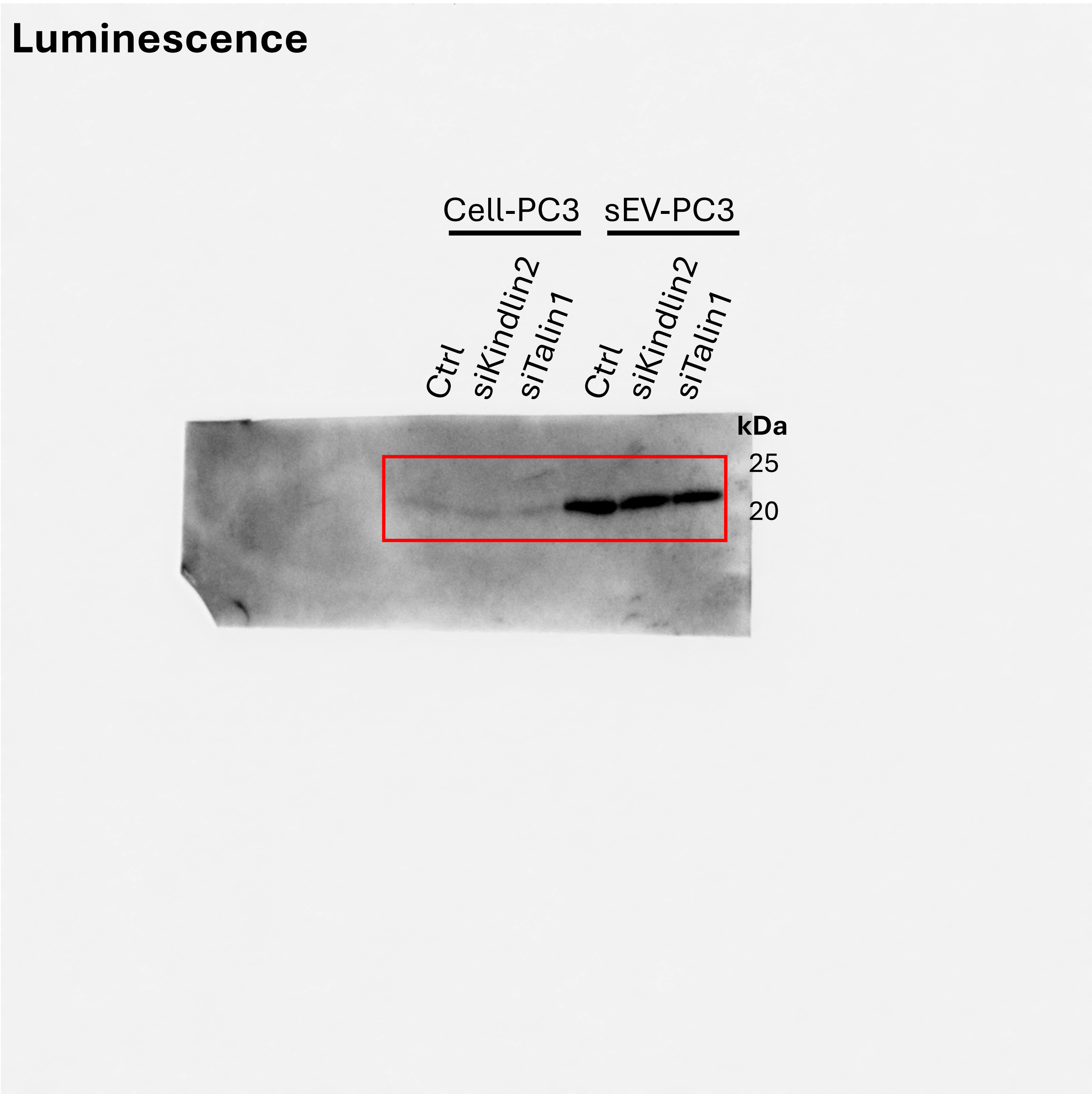

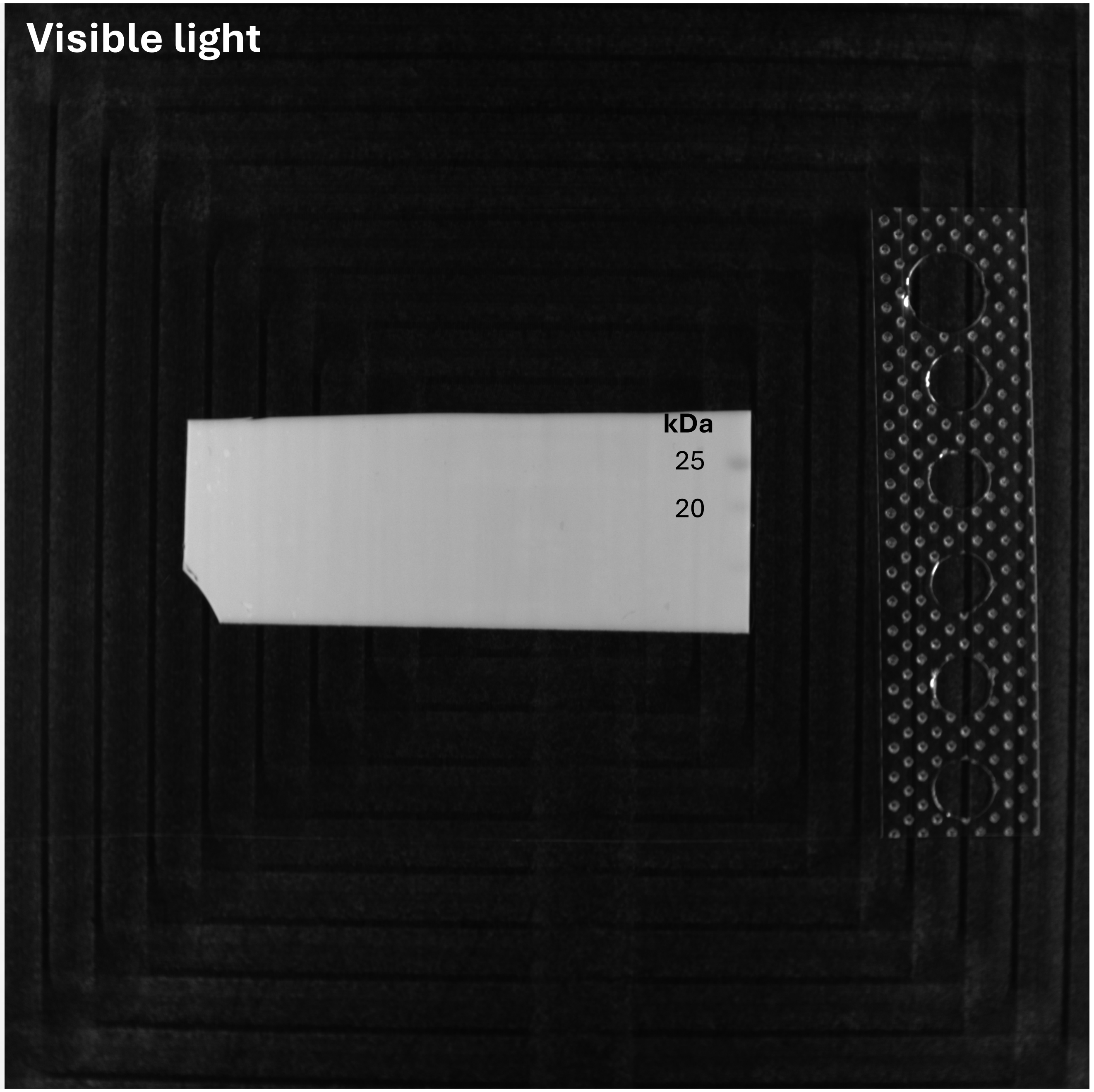

Supplement: SourceData F7 — is the source file for Fig. 7. [file jcb_202404064_sourcedataf7.pdf]
