# Supplementary material for: Extracellular vesicles adhere to cells primarily by interactions of integrins and GM1 with laminin
Source: J Cell Biol. 2025 Apr 30;224(6):e202404064. doi: 10.1083/jcb.202404064 (PMC12042775; doi:10.1083/jcb.202404064)

Fig. 8A, 8G

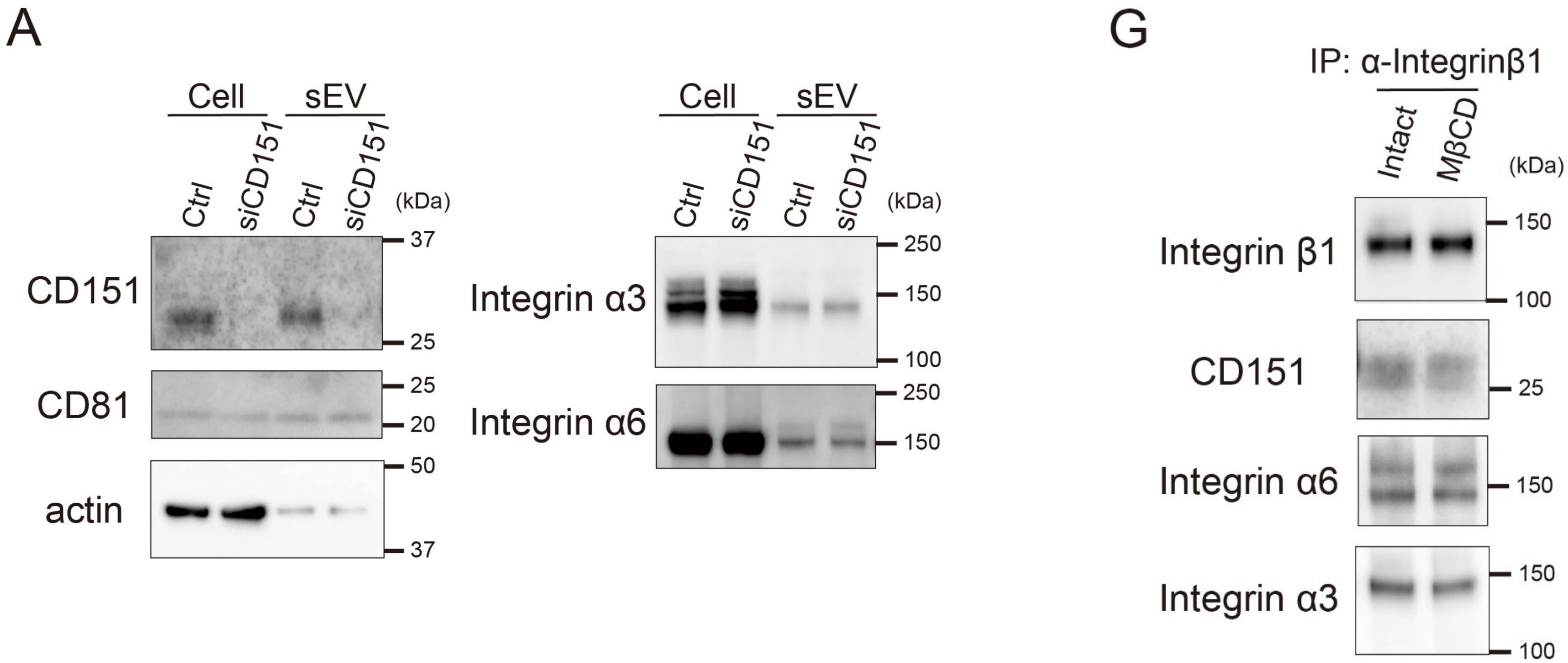

A
Cell
sEV
Ctrl
siCD151
Ctrl
siCD151
(kDa)
CD151
37
25
CD81
25
20
actin
50
37
Cell
sEV
Ctrl
siCD151
Ctrl
siCD151
(kDa)
Integrin α3
250
150
100
Integrin α6
250
150
G
IP: α-Integrinβ1
Intact
MβCD
(kDa)
Integrin β1
150
100
CD151
25
Integrin α6
150
Integrin α3
150
100

SourceDataF8A_CD151

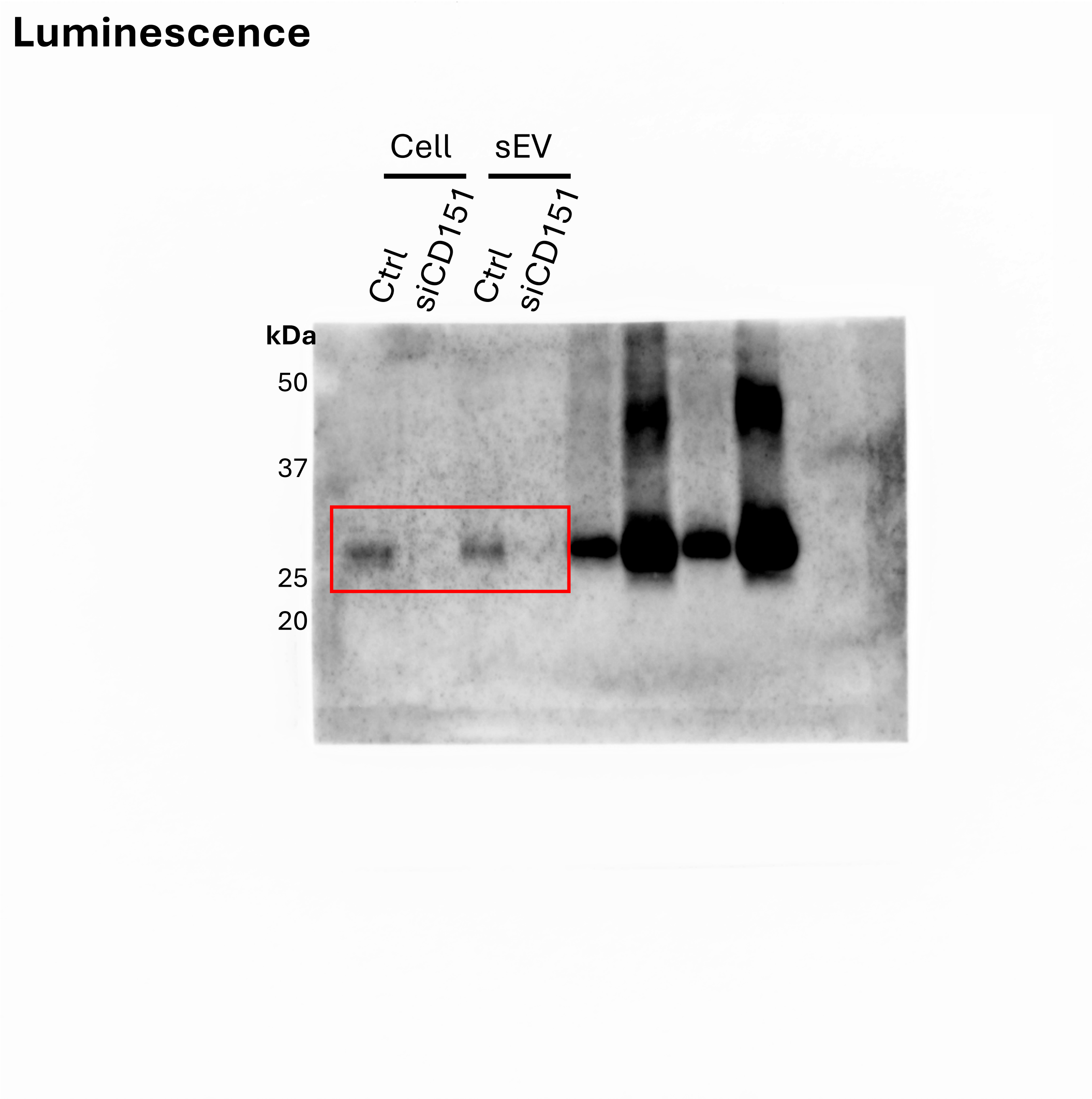

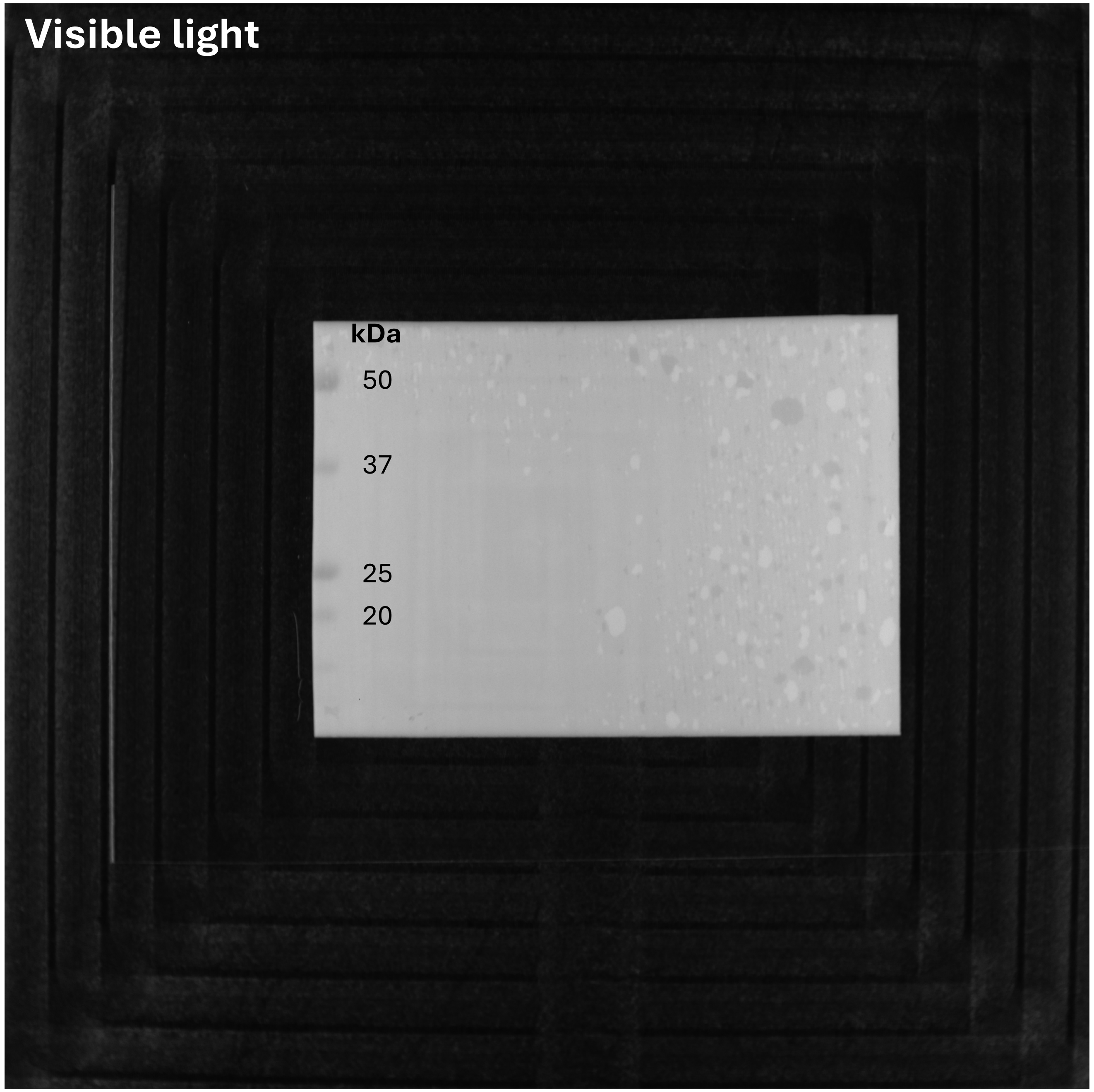

SourceDataF8A_CD81

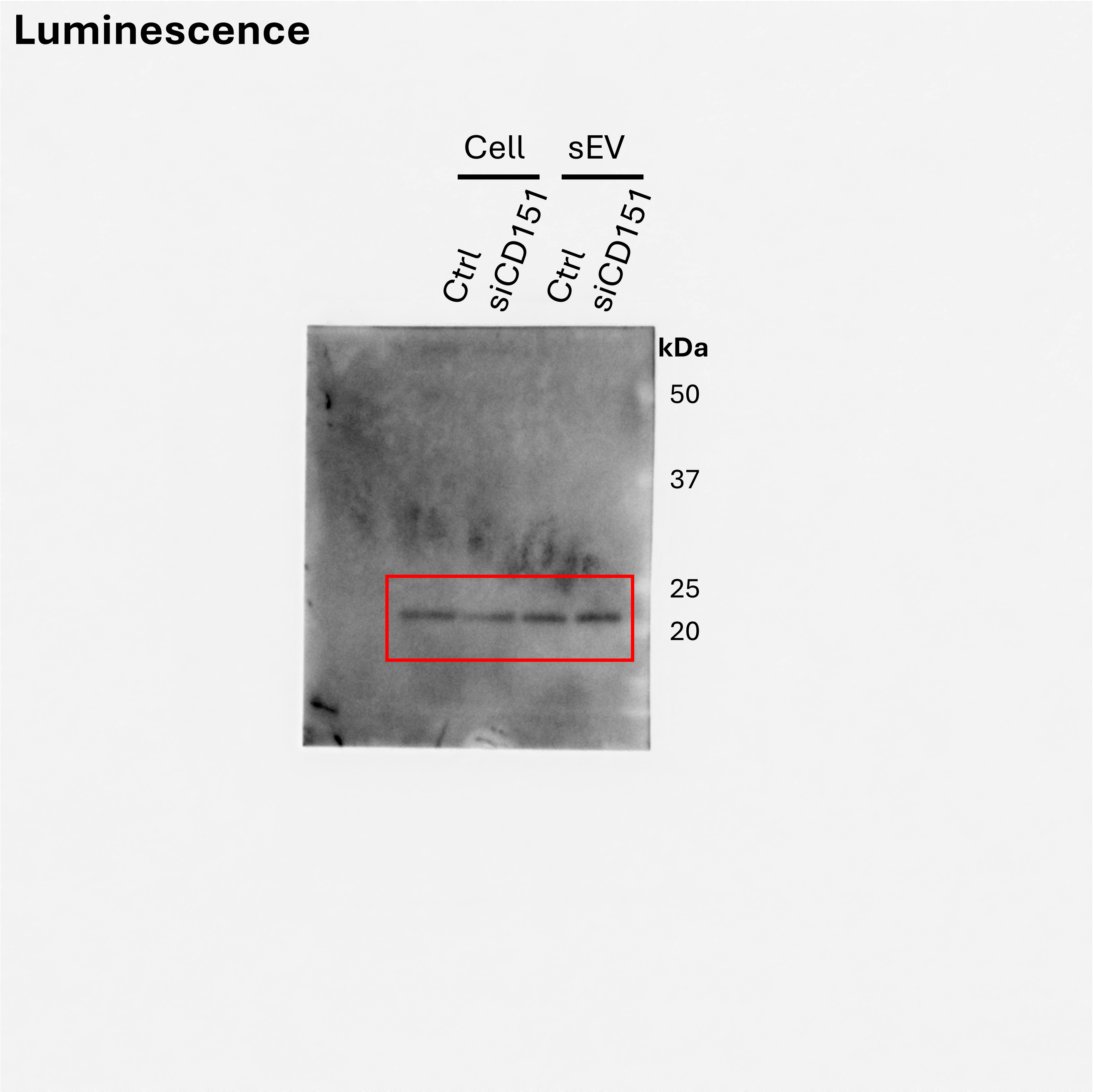

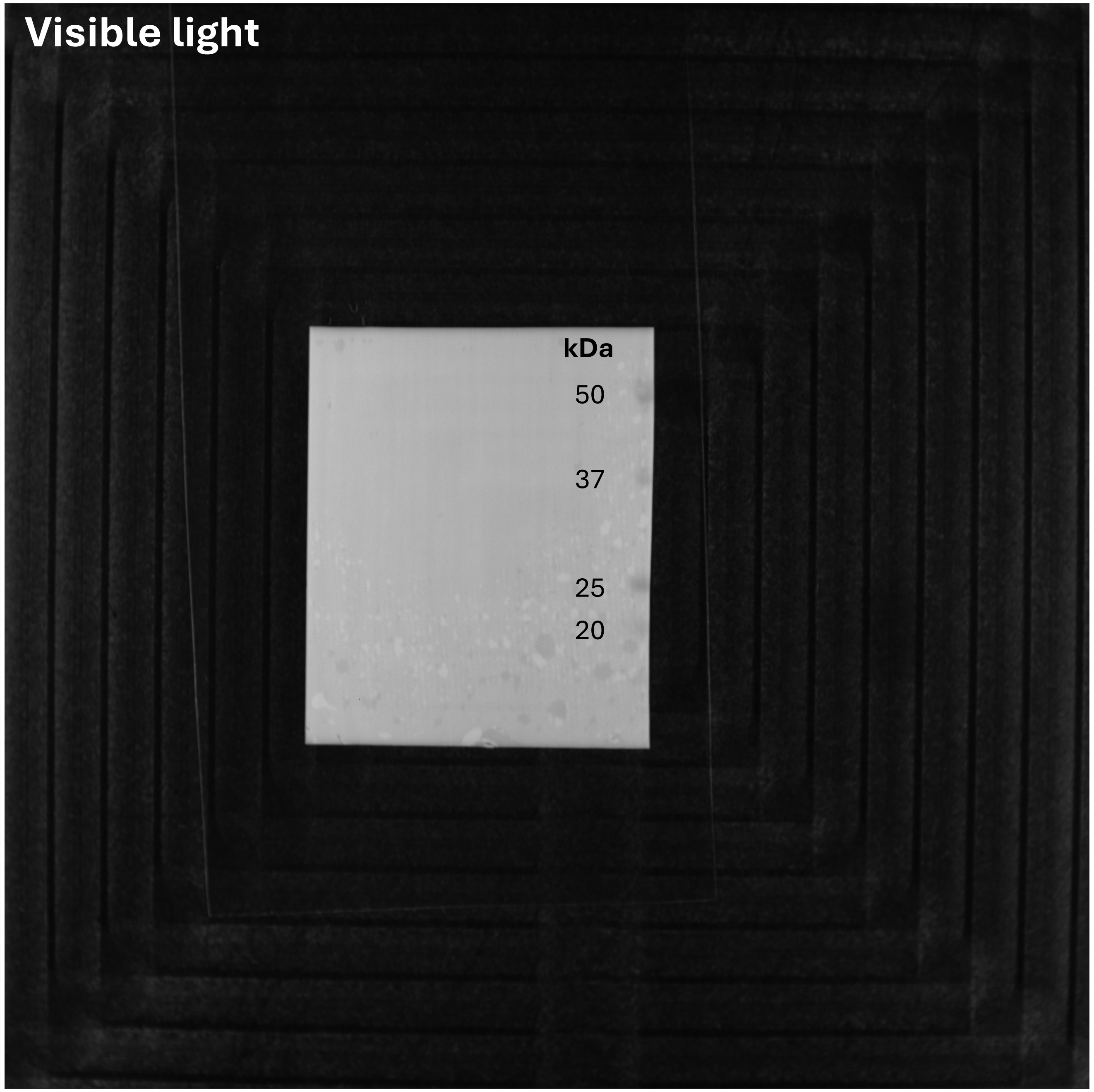

SourceDataF8A_actin

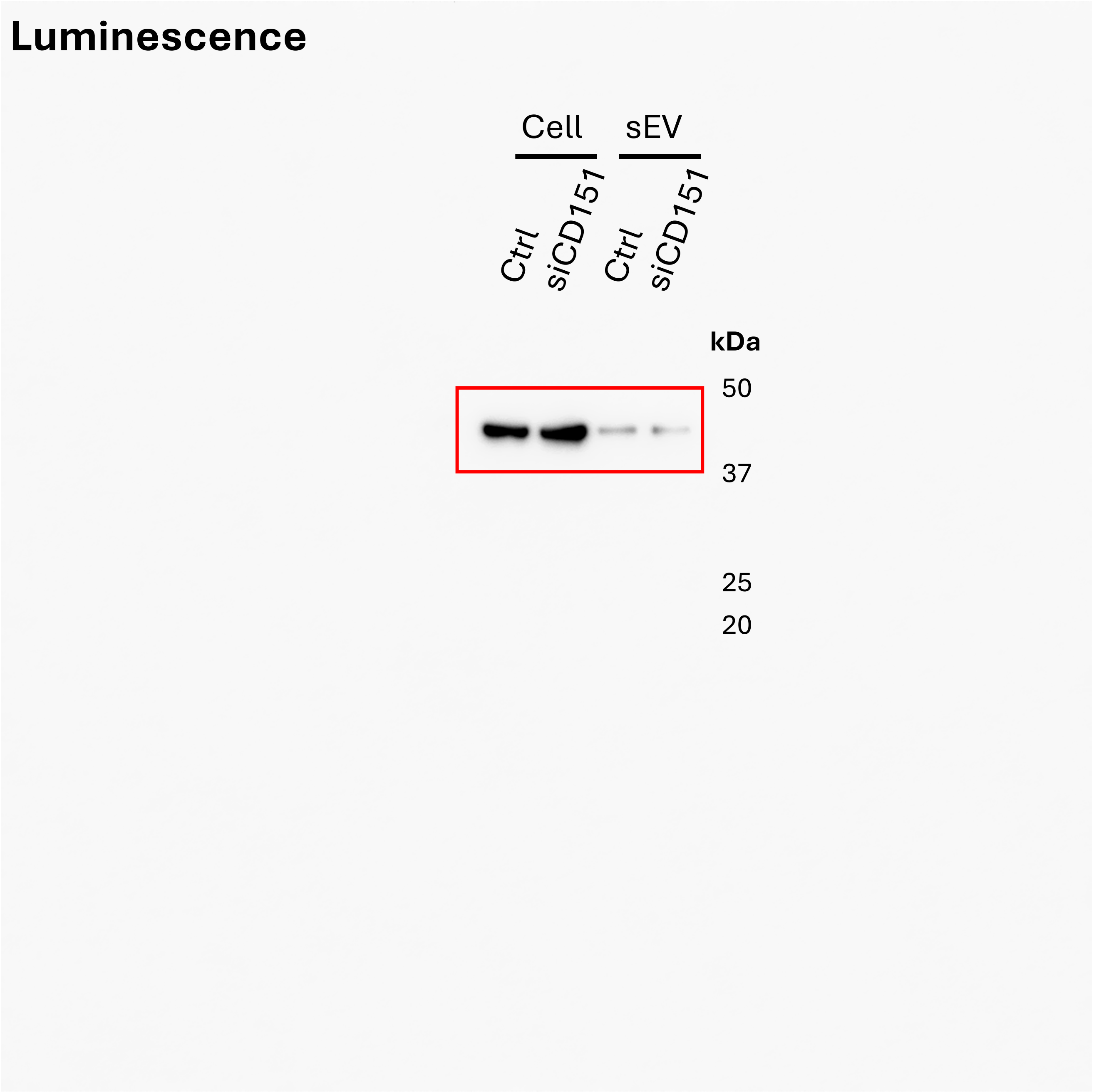

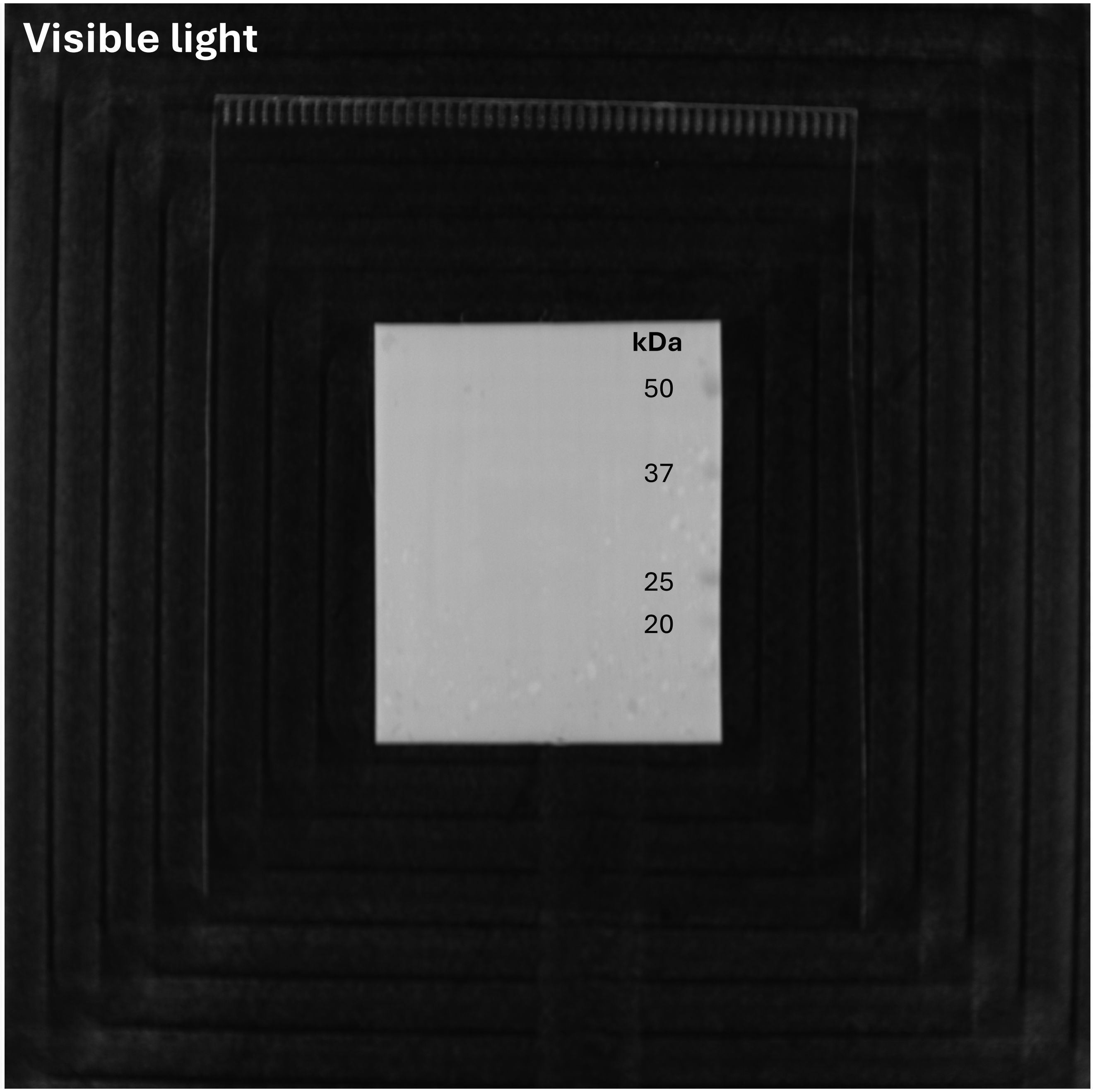

SourceDataF8A_Integrin α3

Luminescence

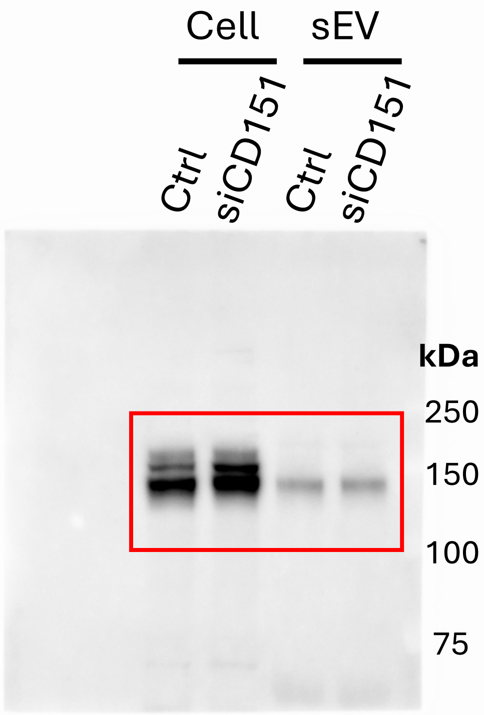

Visible light

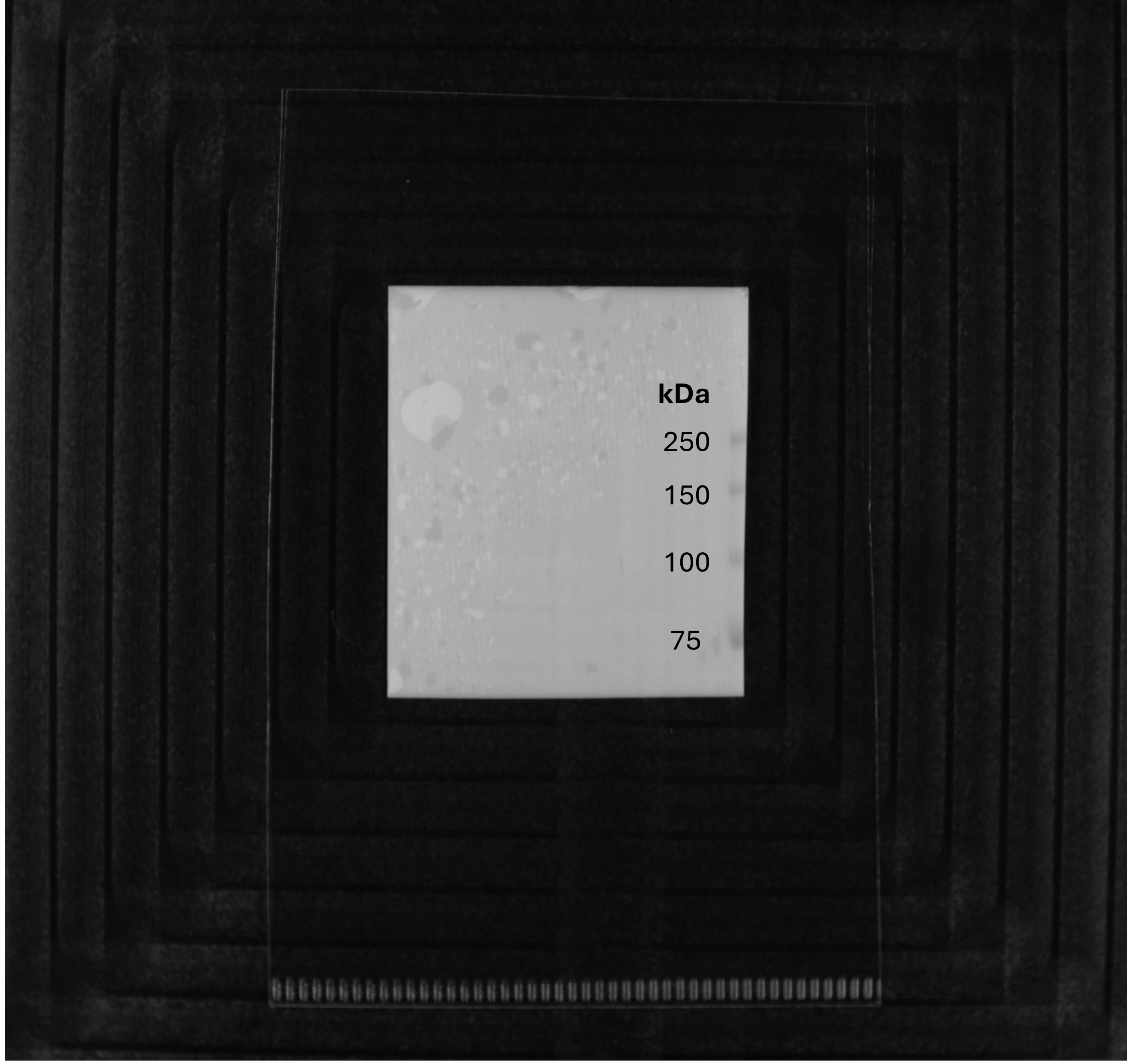

SourceDataF8A_Integrin α6

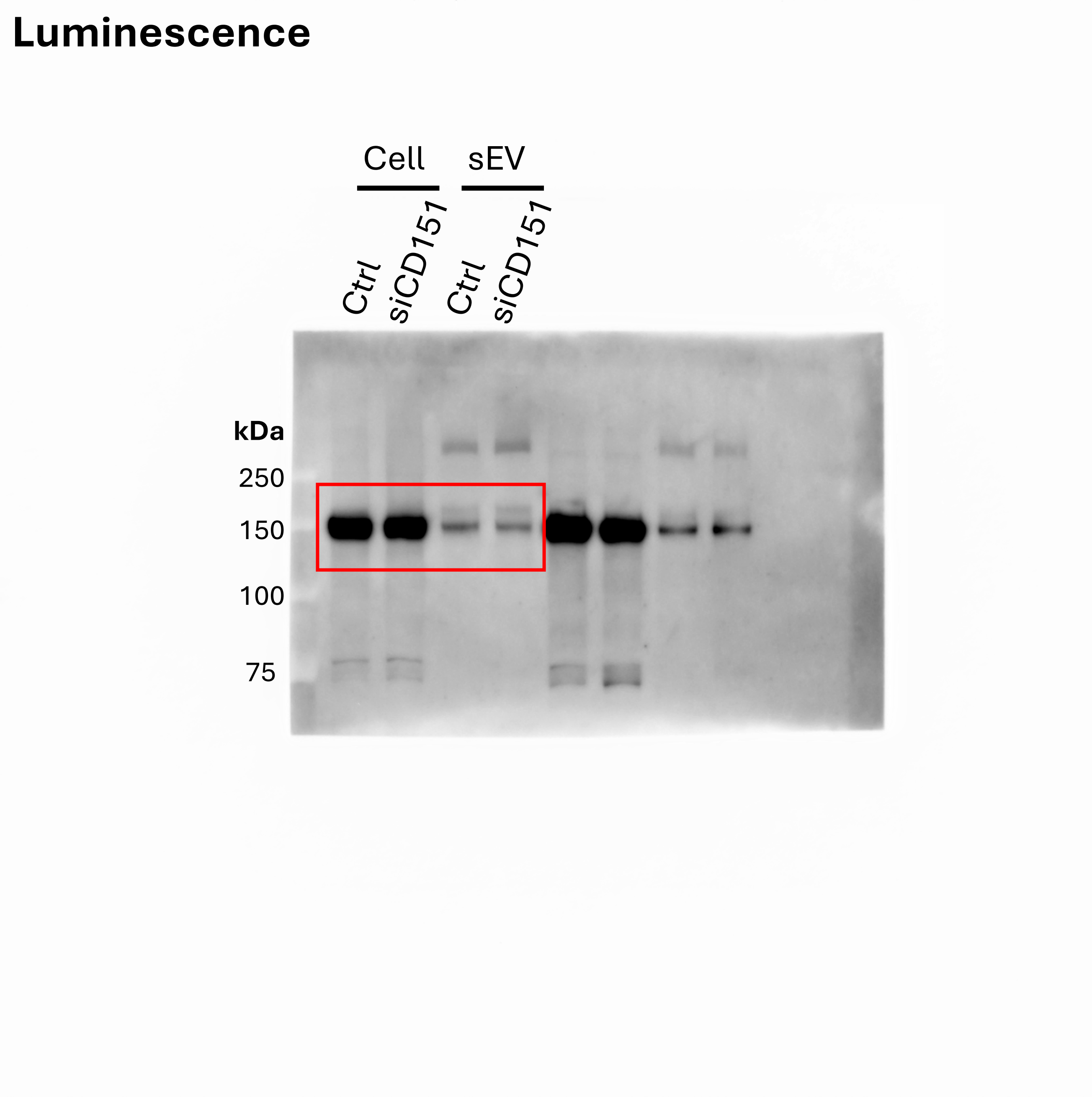

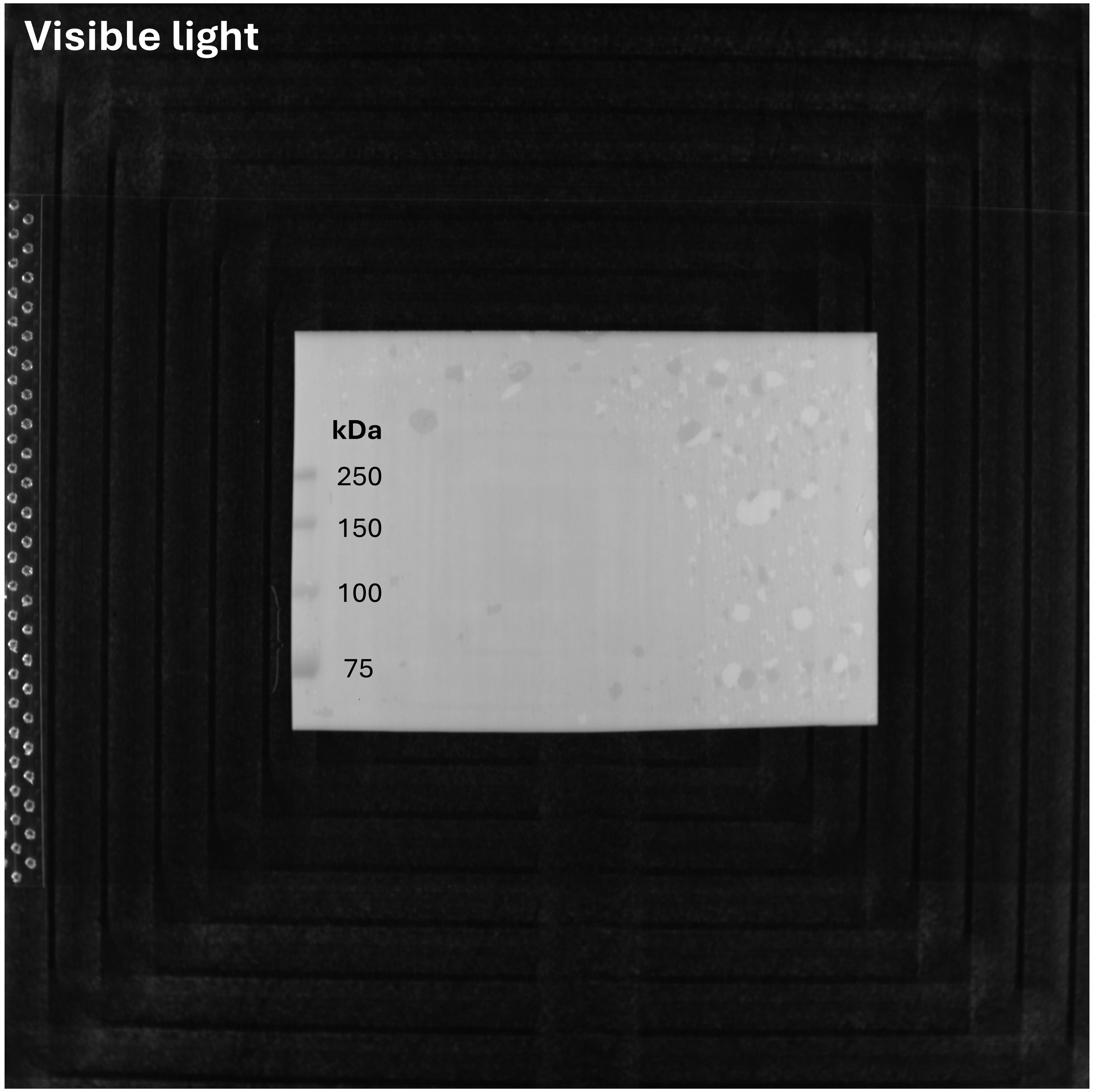

SourceDataF8G_Integrin β1

Luminescence

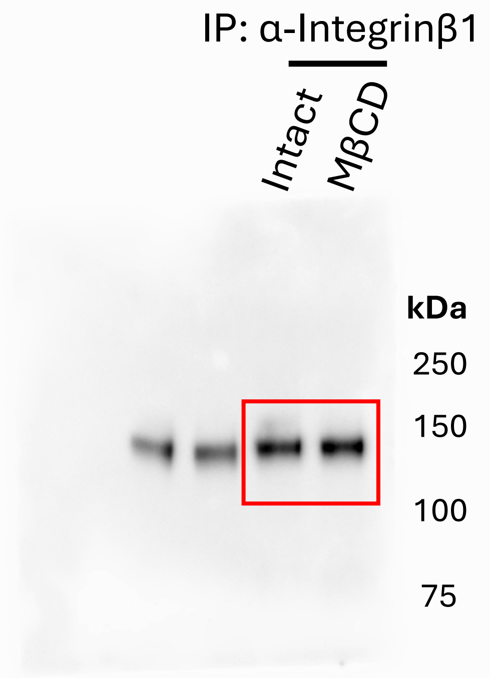

Visible light

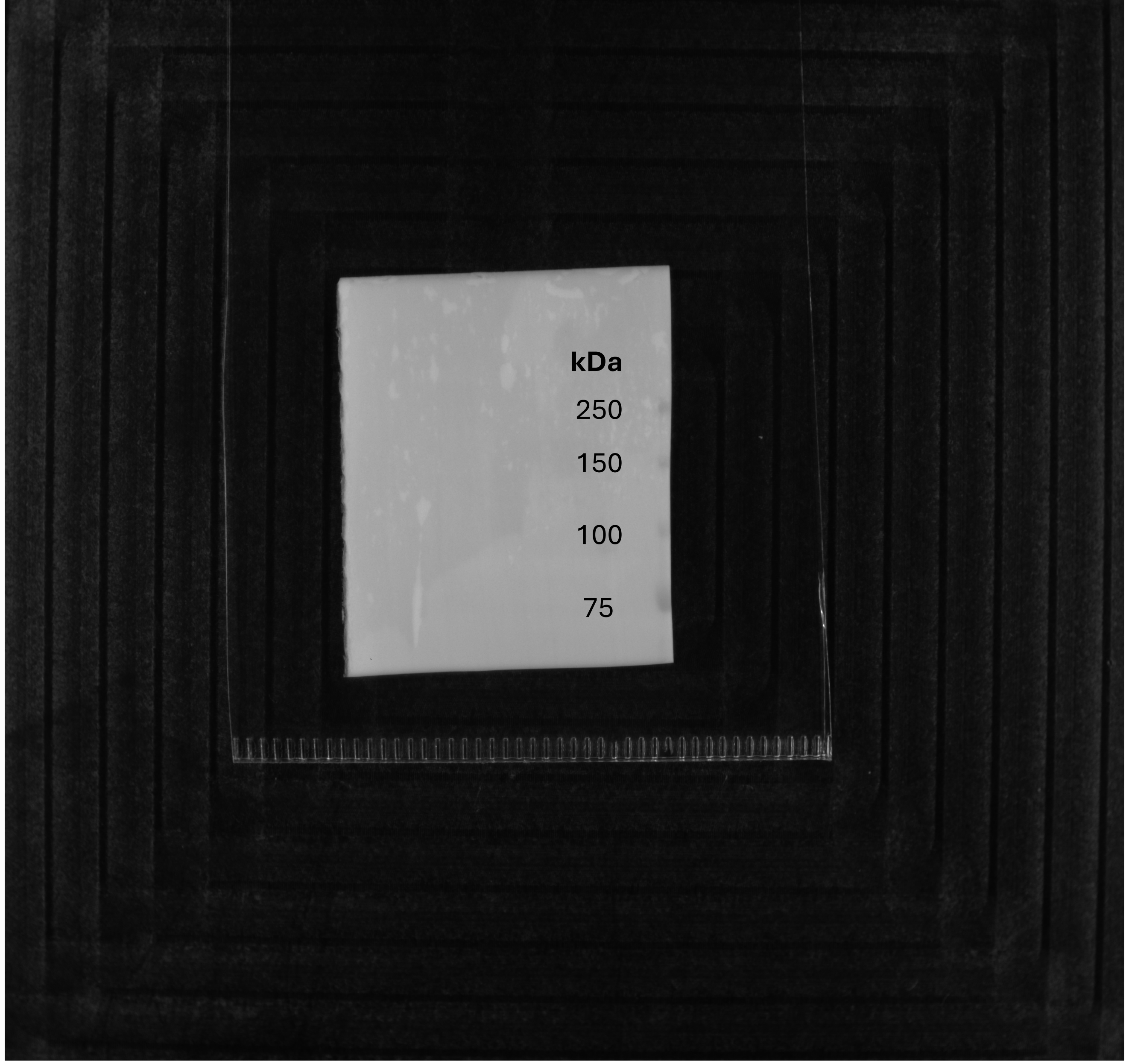

SourceDataF8G_CD151

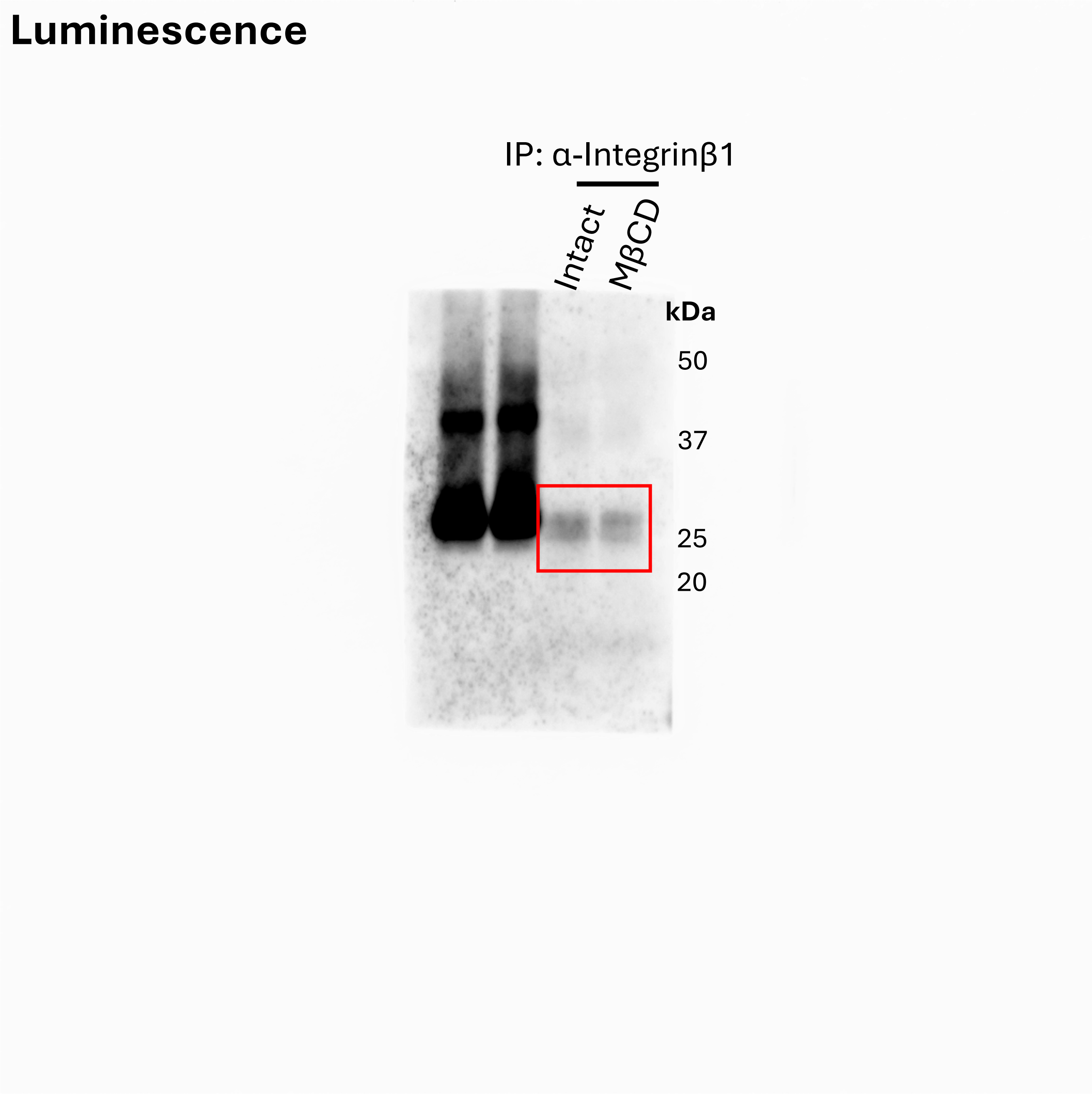

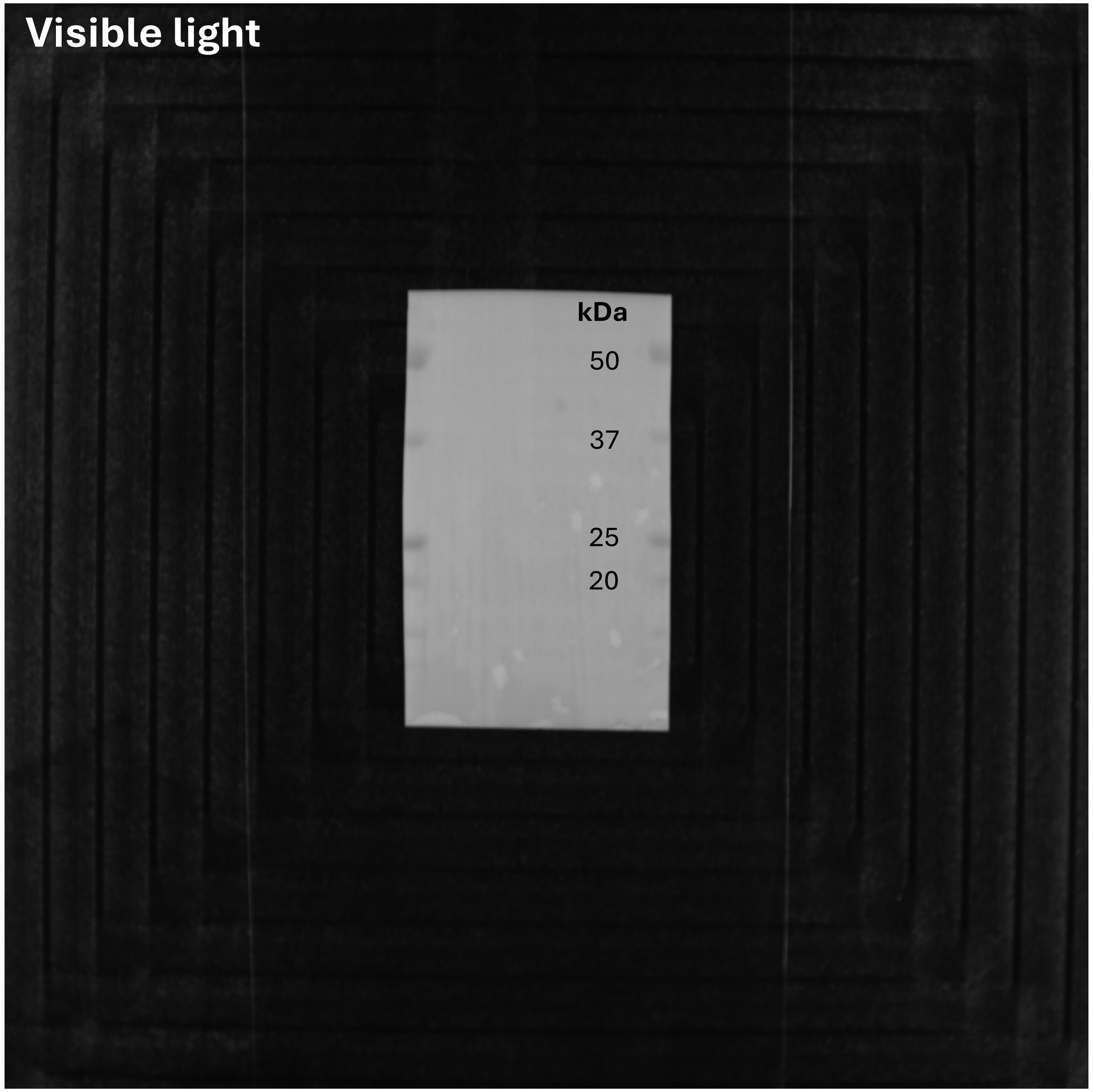

SourceDataF8G_Integrin α6

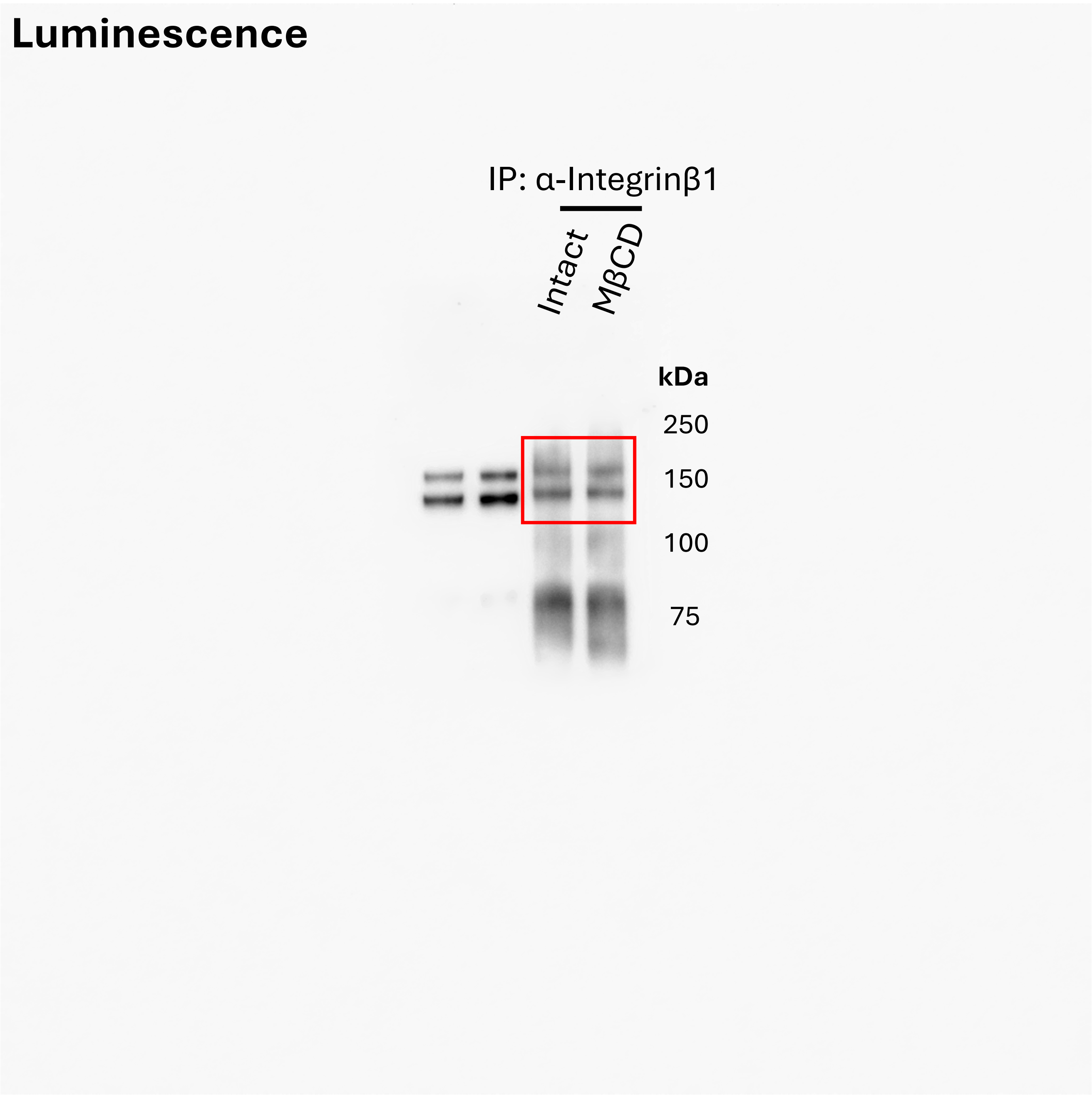

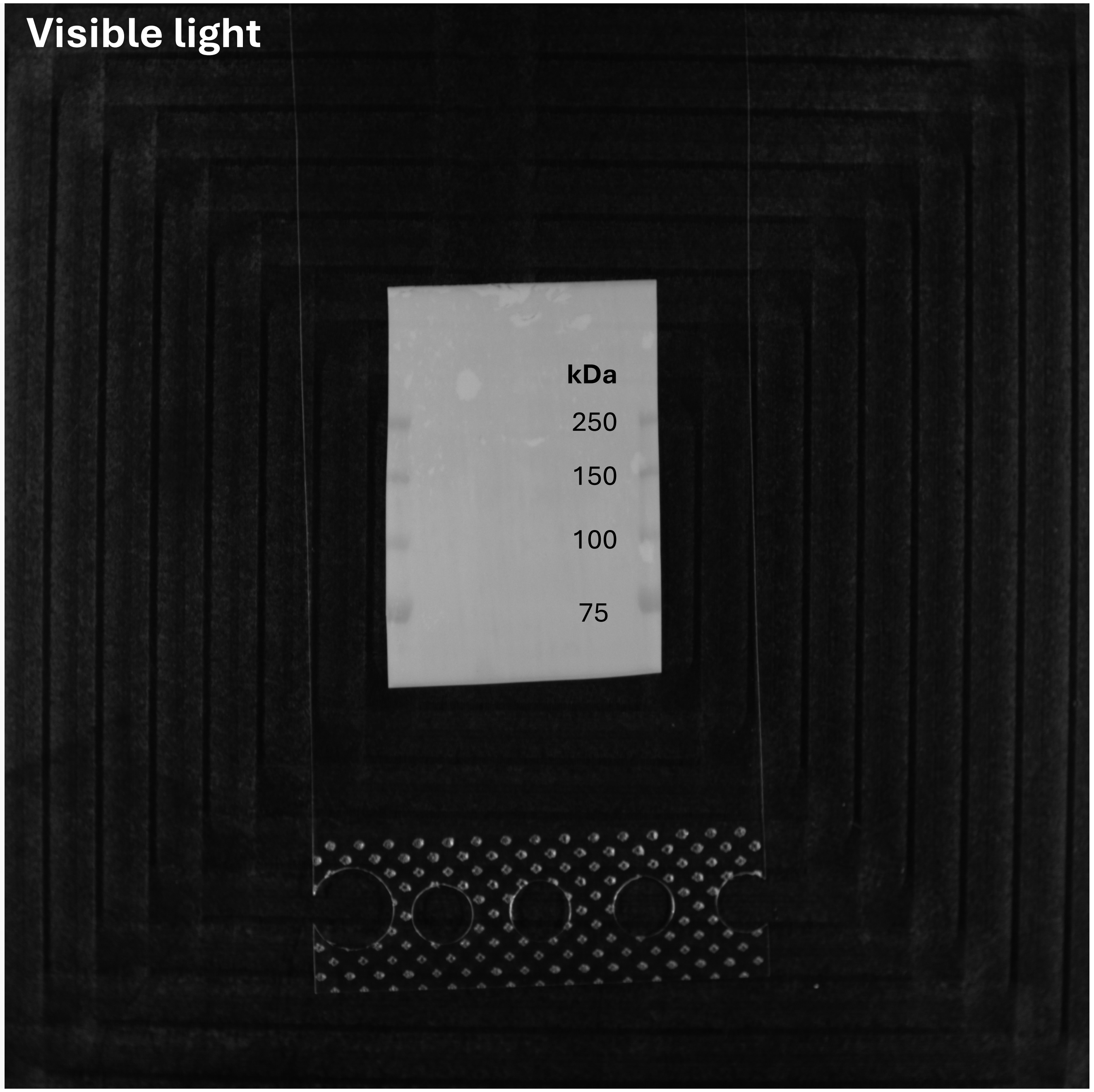

SourceDataF8G_Integrin α3

Luminescence

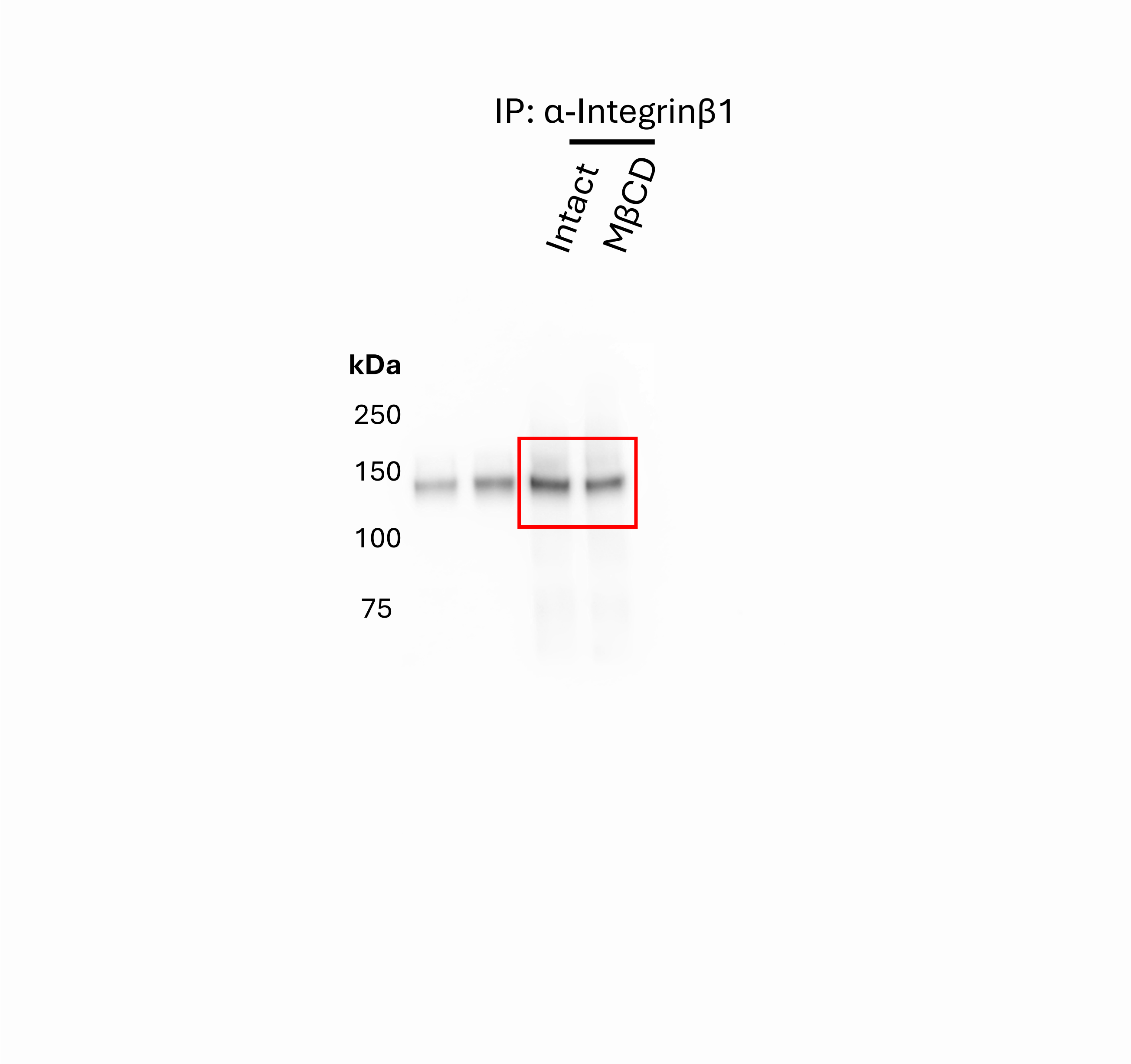

Visible light

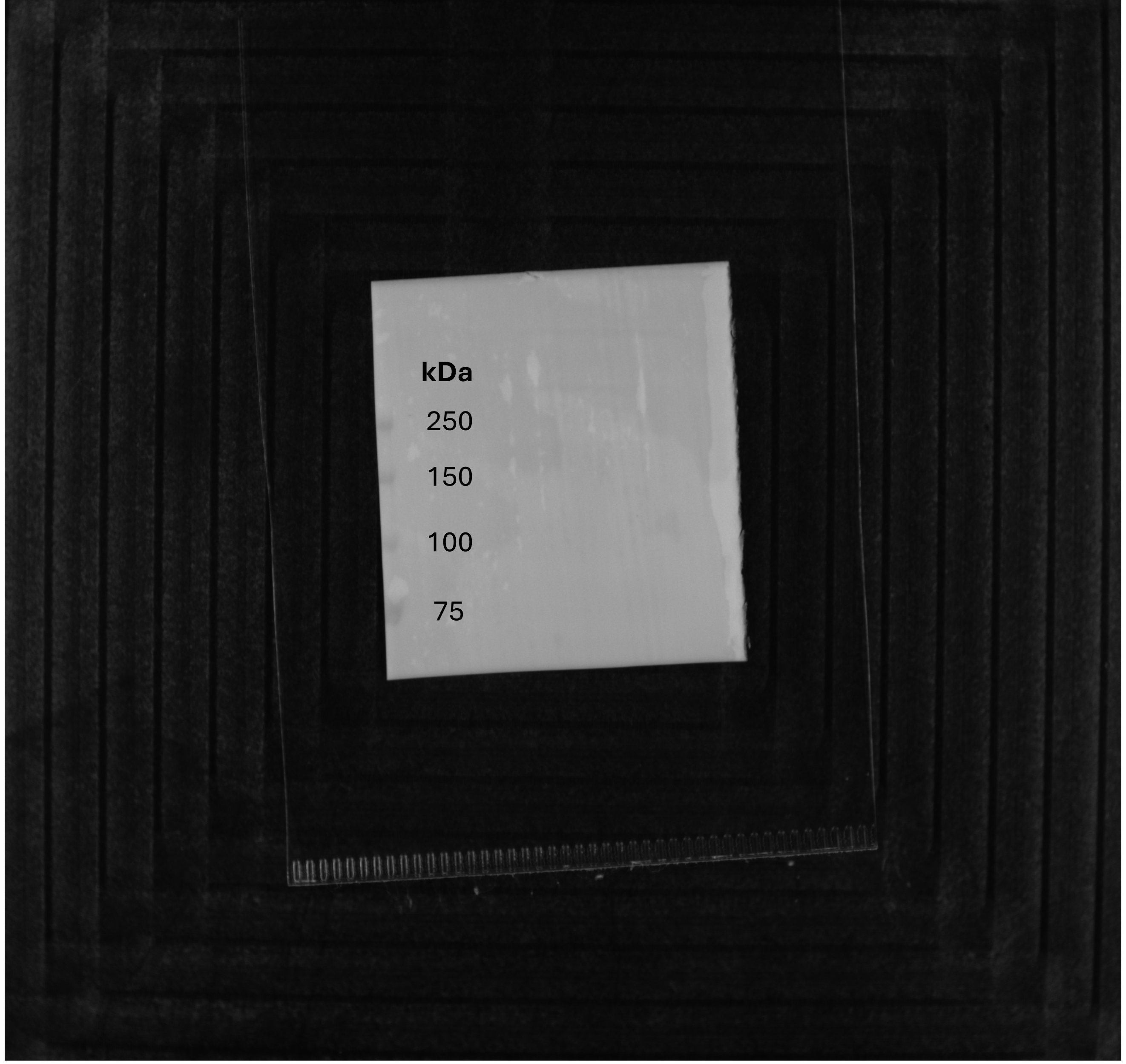

Supplement: SourceData F8 — is the source file for Fig. 8. [file jcb_202404064_sourcedataf8.pdf]
