# Supplementary material for: Extracellular vesicles adhere to cells primarily by interactions of integrins and GM1 with laminin
Source: J Cell Biol. 2025 Apr 30;224(6):e202404064. doi: 10.1083/jcb.202404064 (PMC12042775; doi:10.1083/jcb.202404064)

Fig. 9A

A

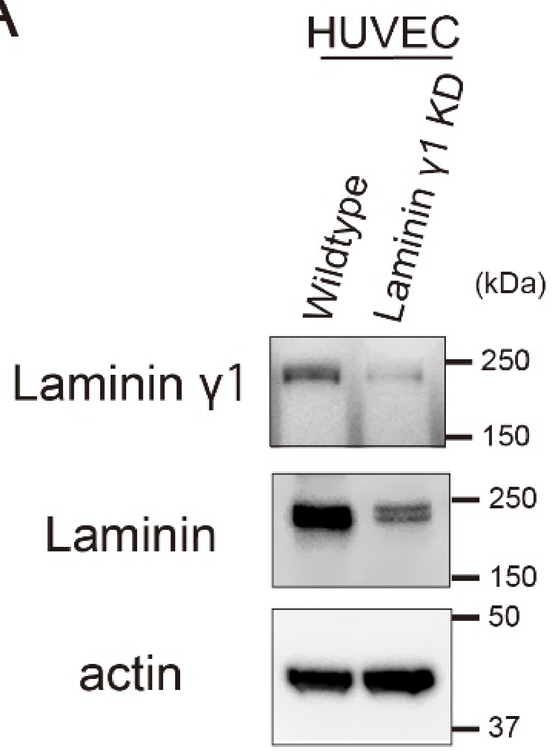
HUVEC
Wildtype
Laminin γ1 KD
(kDa)
Laminin γ1
Laminin
actin
250
150
250
150
50
37

SourceDataF9A_Lamininγ1

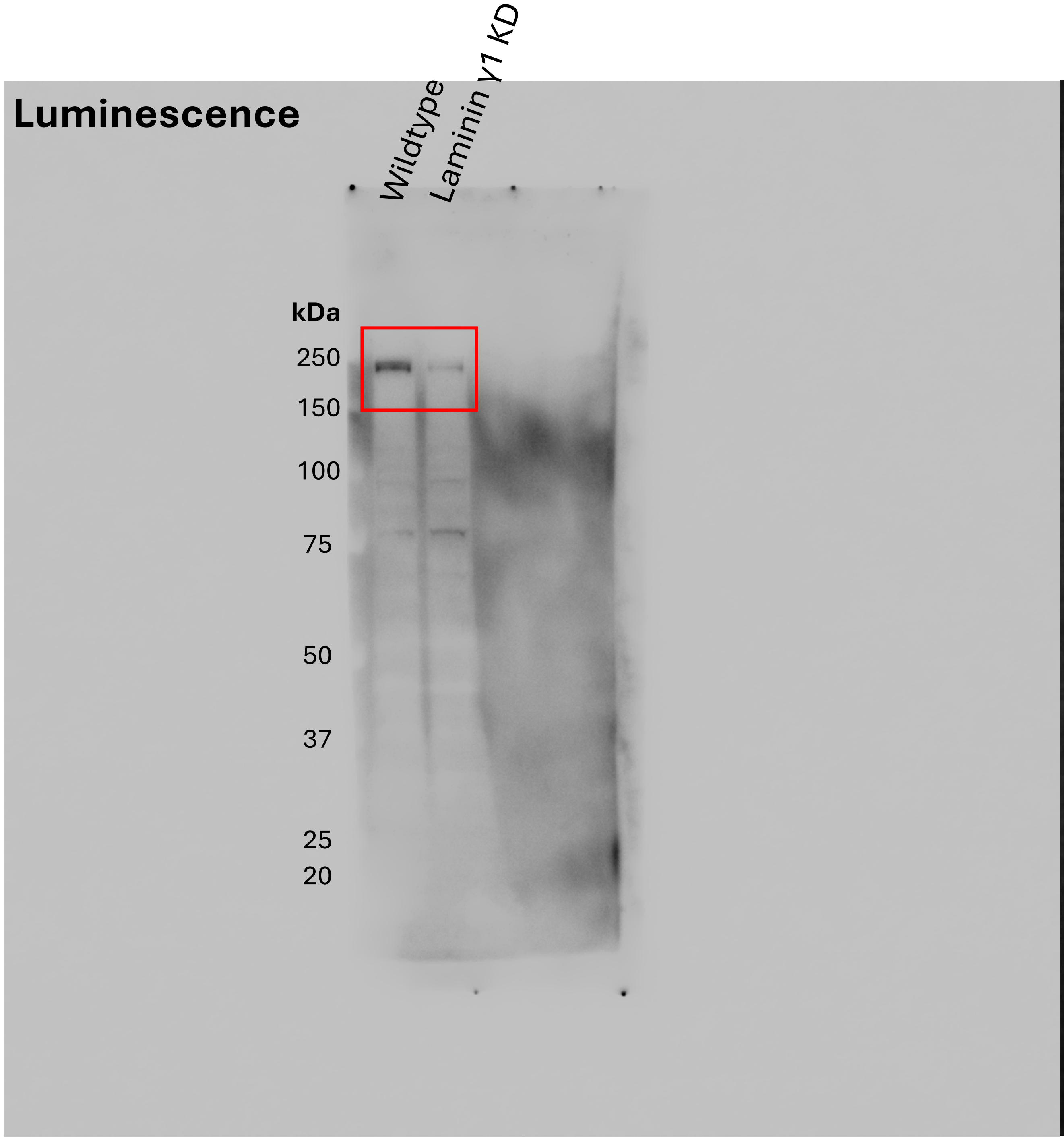

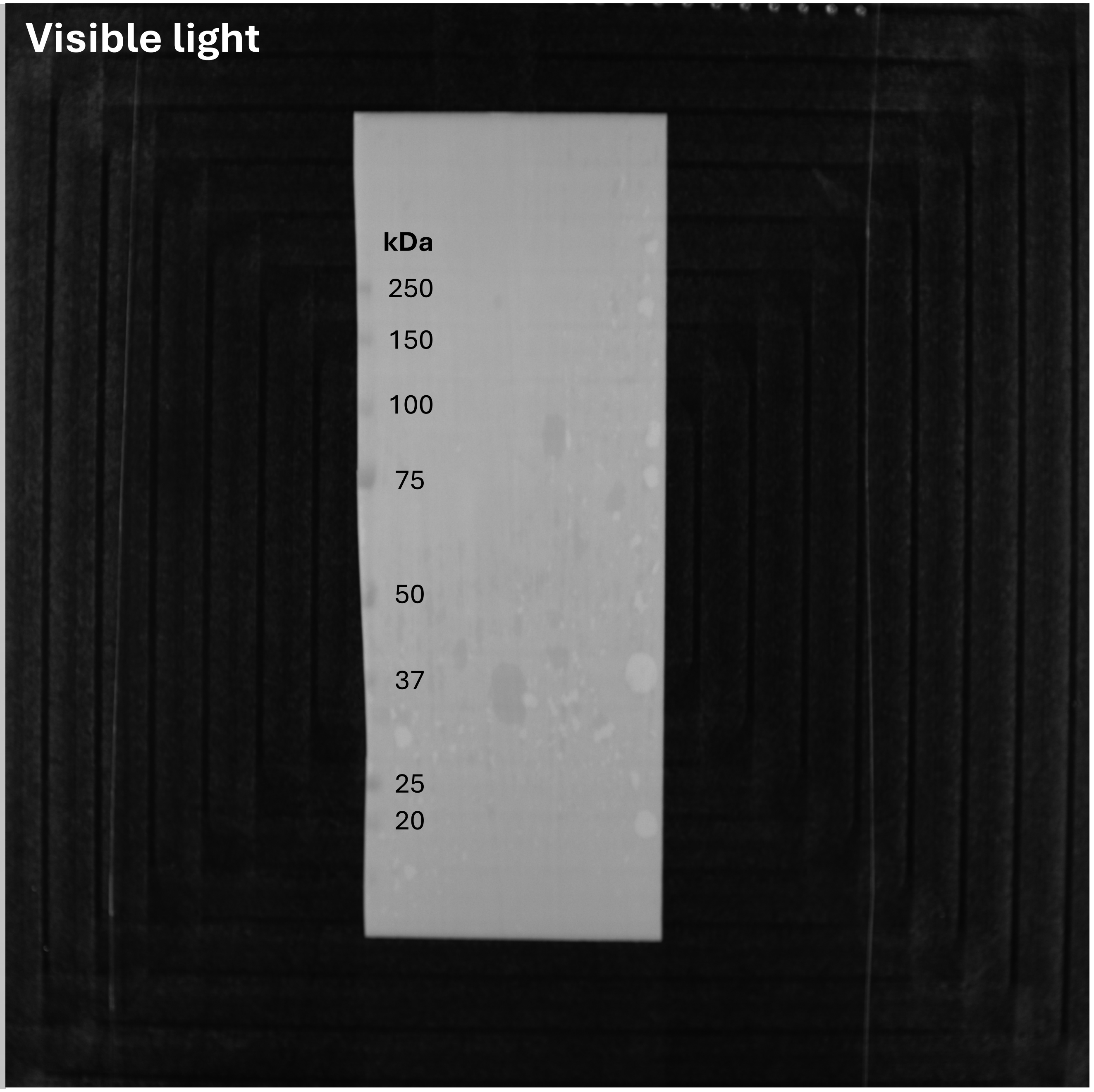

SourceDataF9A_Laminin(polyclonal)

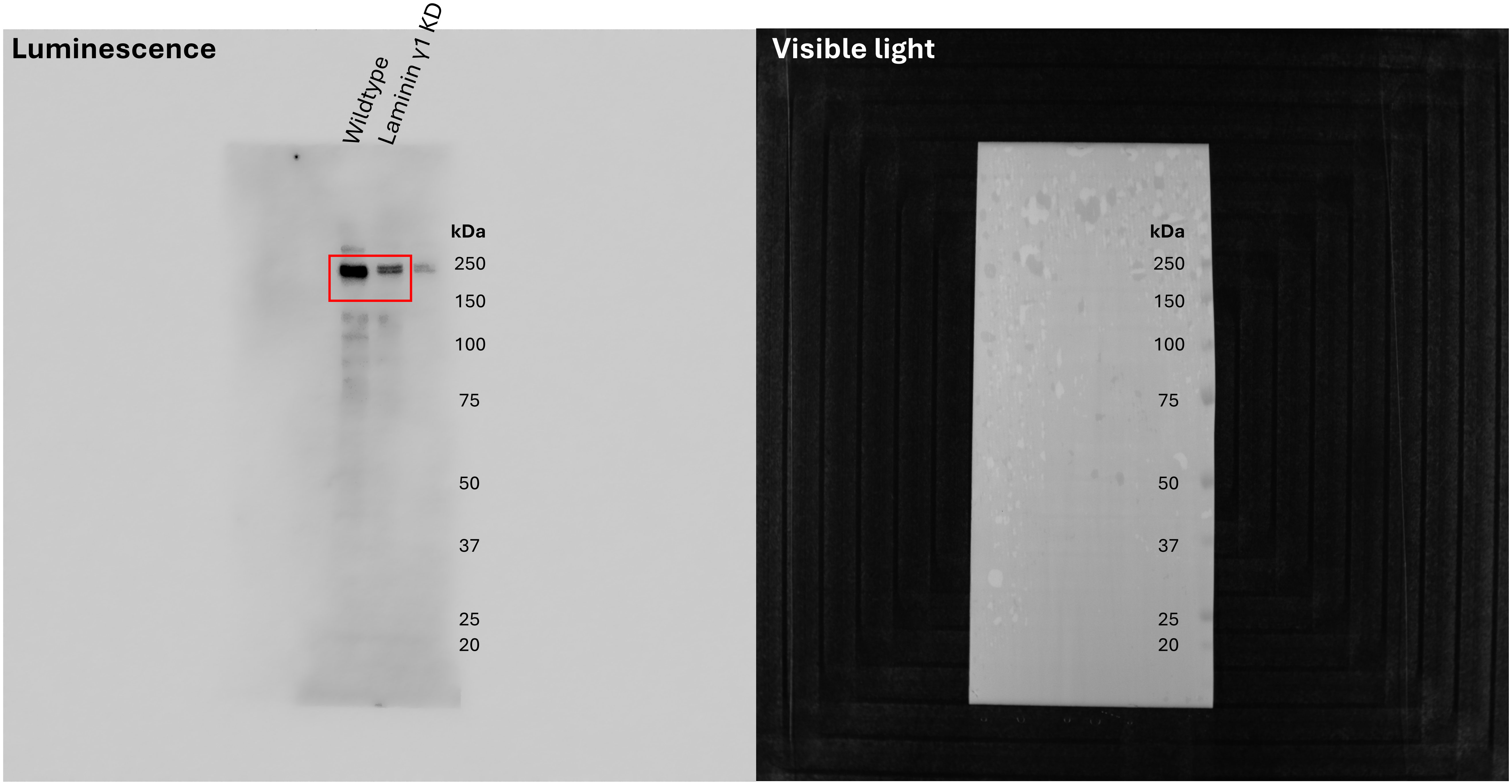

SourceDataF9A_actin

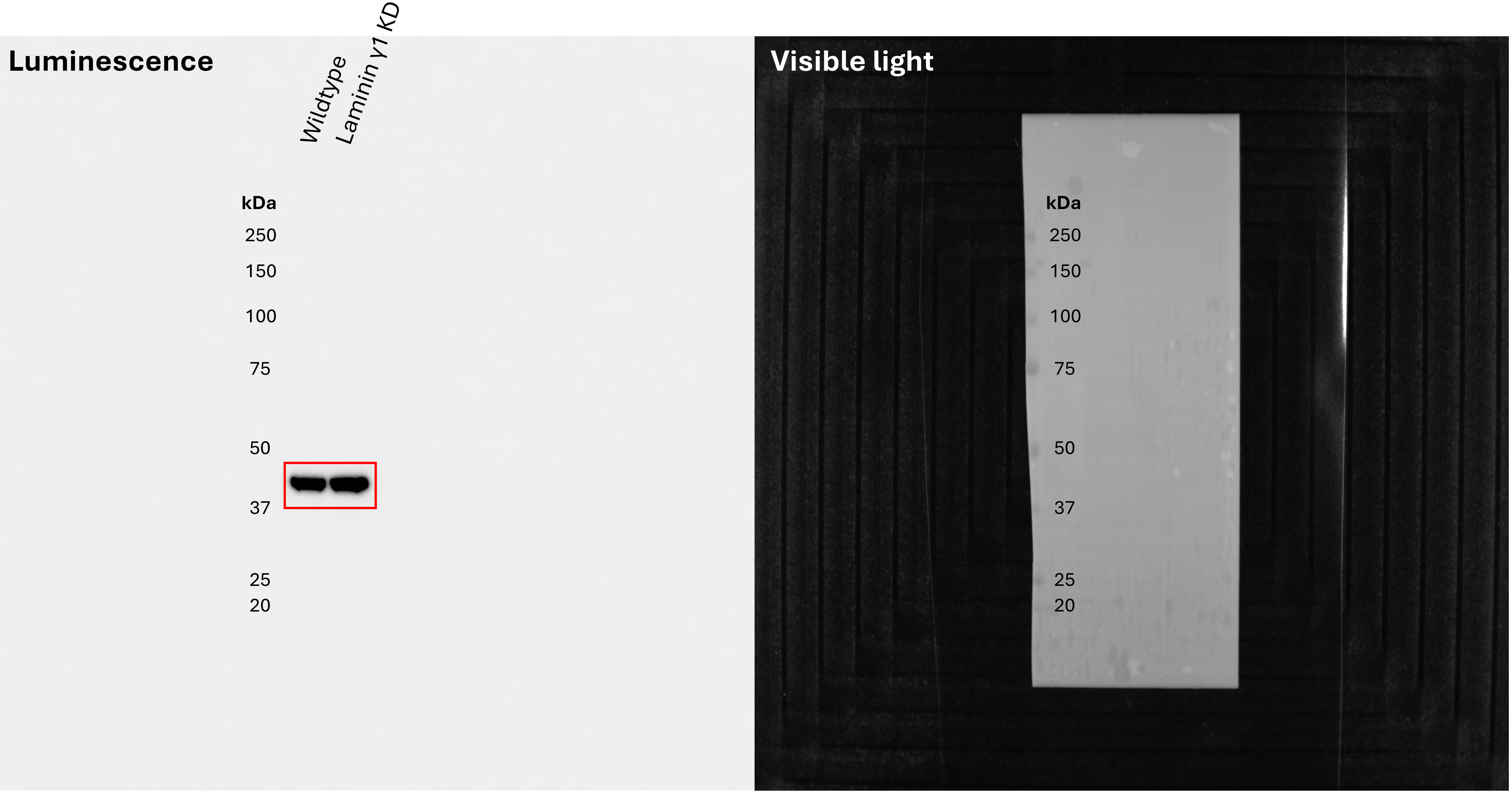

Supplement: SourceData F9 — is the source file for Fig. 9. [file jcb_202404064_sourcedataf9.pdf]
