# Supplementary material for: Extracellular vesicles adhere to cells primarily by interactions of integrins and GM1 with laminin
Source: J Cell Biol. 2025 Apr 30;224(6):e202404064. doi: 10.1083/jcb.202404064 (PMC12042775; doi:10.1083/jcb.202404064)

SupFig. 3B, 3C

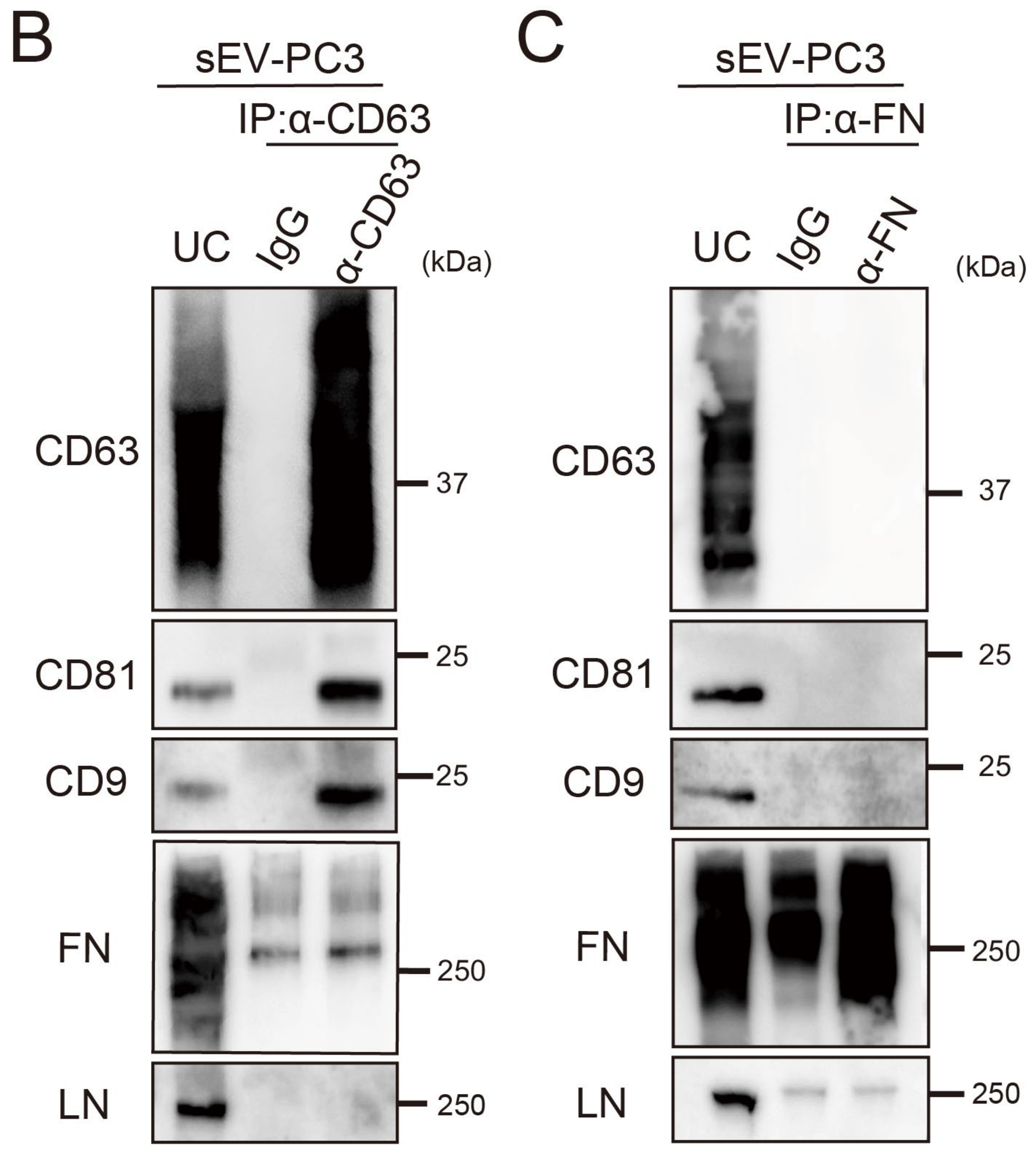
B
sEV-PC3
IP:α-CD63
UC
IgG
α-CD63
(kDa)
CD63
37
CD81
25
CD9
25
FN
250
LN
250
C
sEV-PC3
IP:α-FN
UC
IgG
α-FN
(kDa)
CD63
37
CD81
25
CD9
25
FN
250
LN
250

SourceDataSF3B_CD63

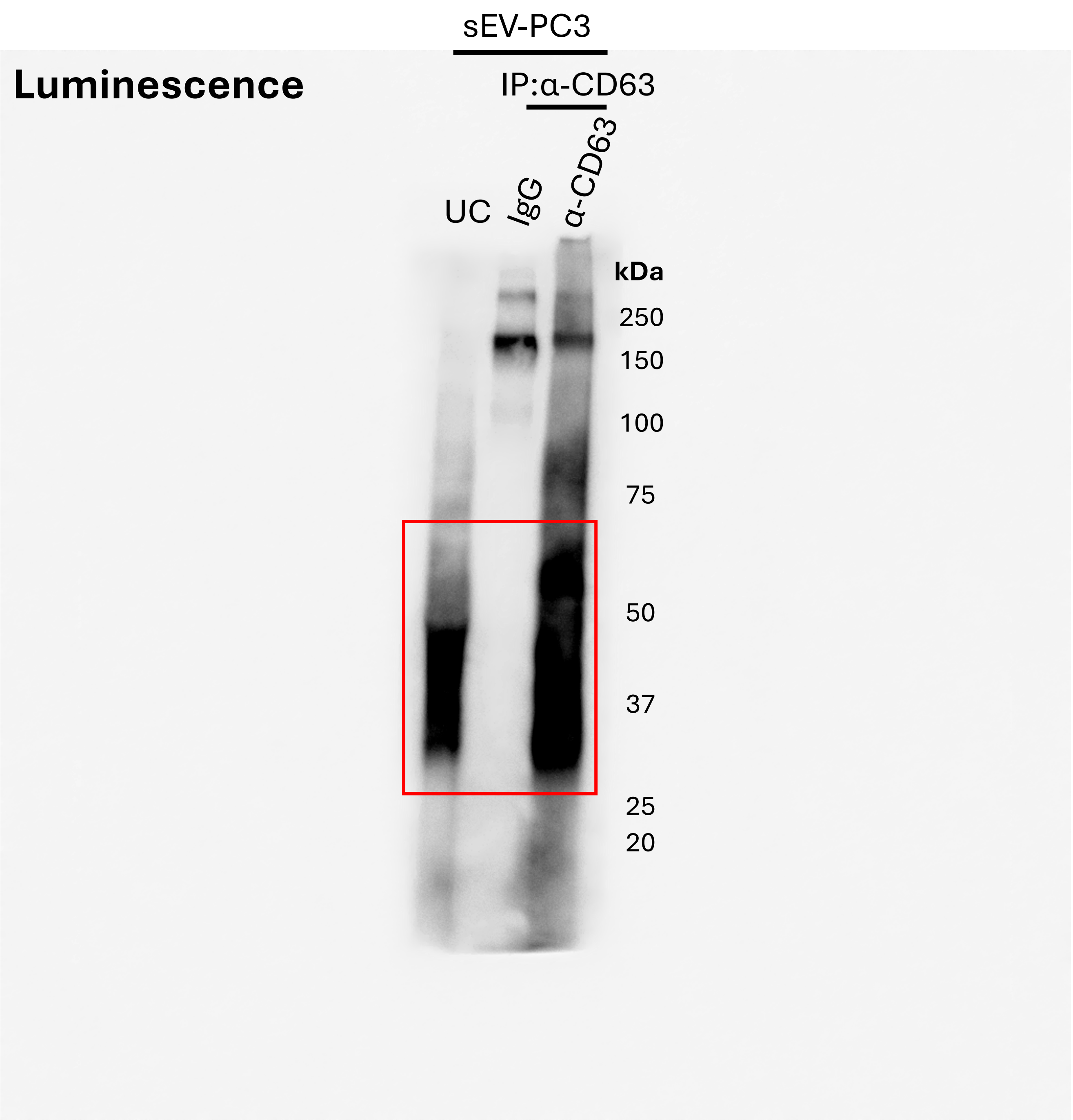

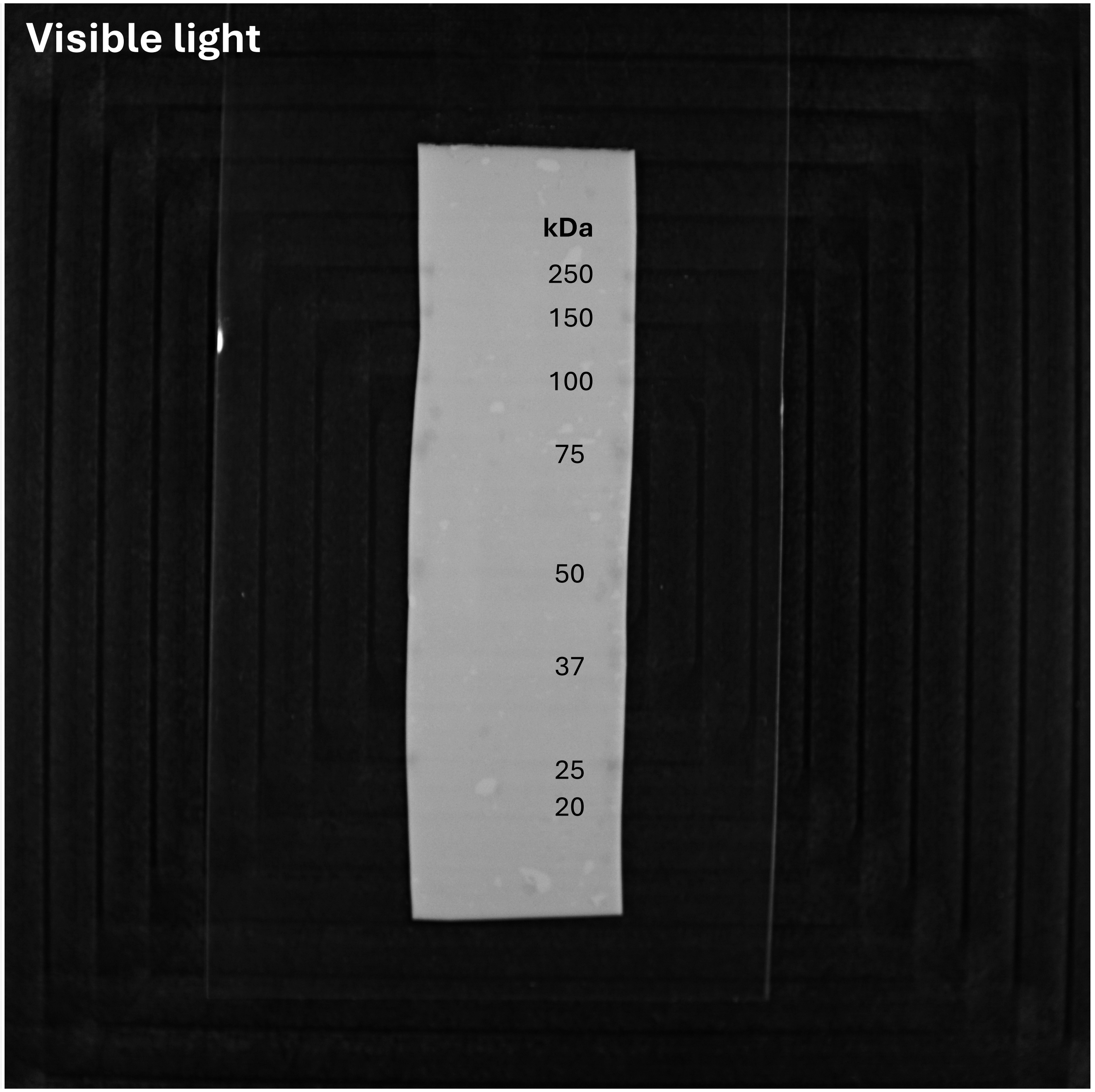

SourceDataSF3B_CD81

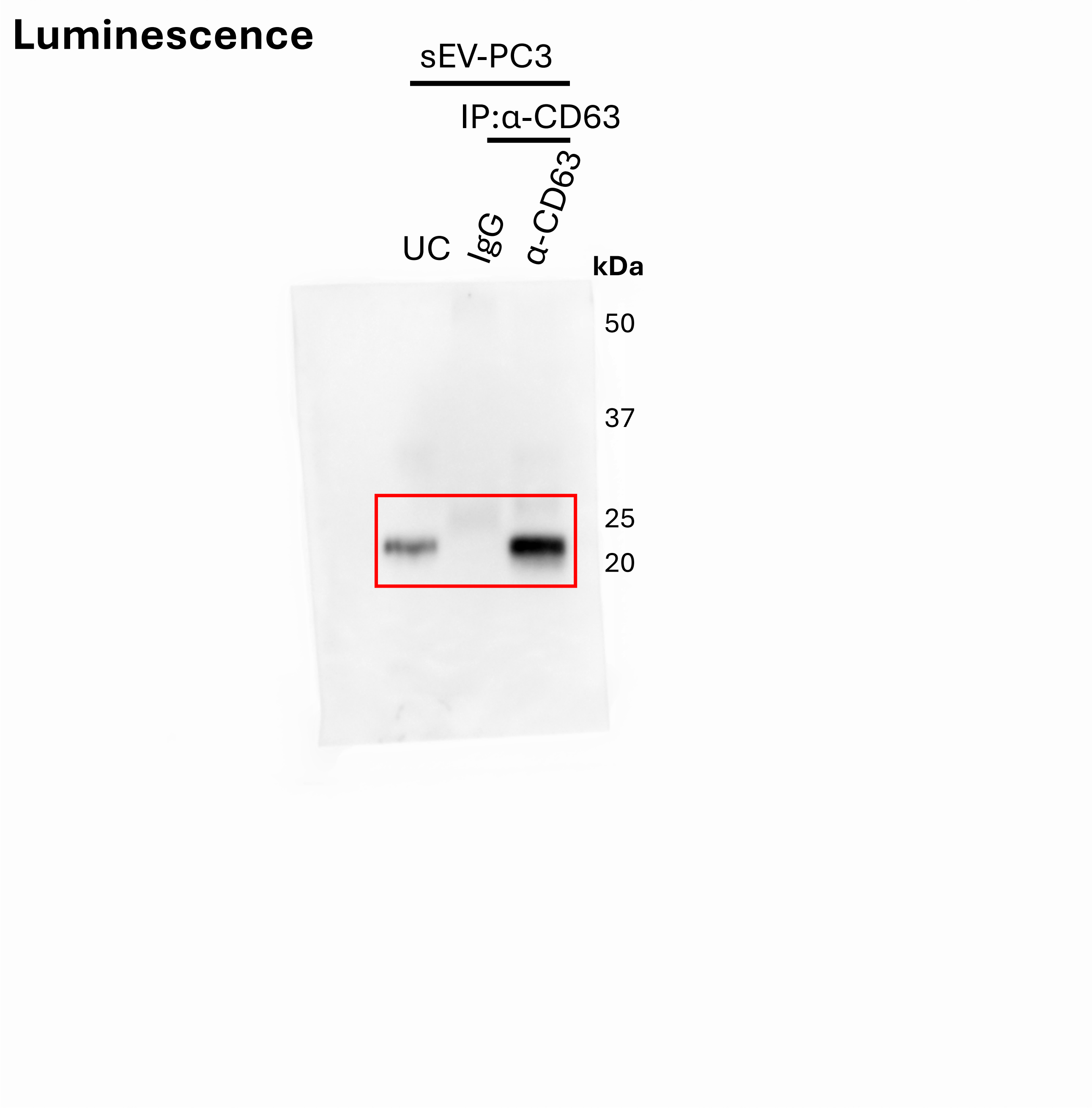

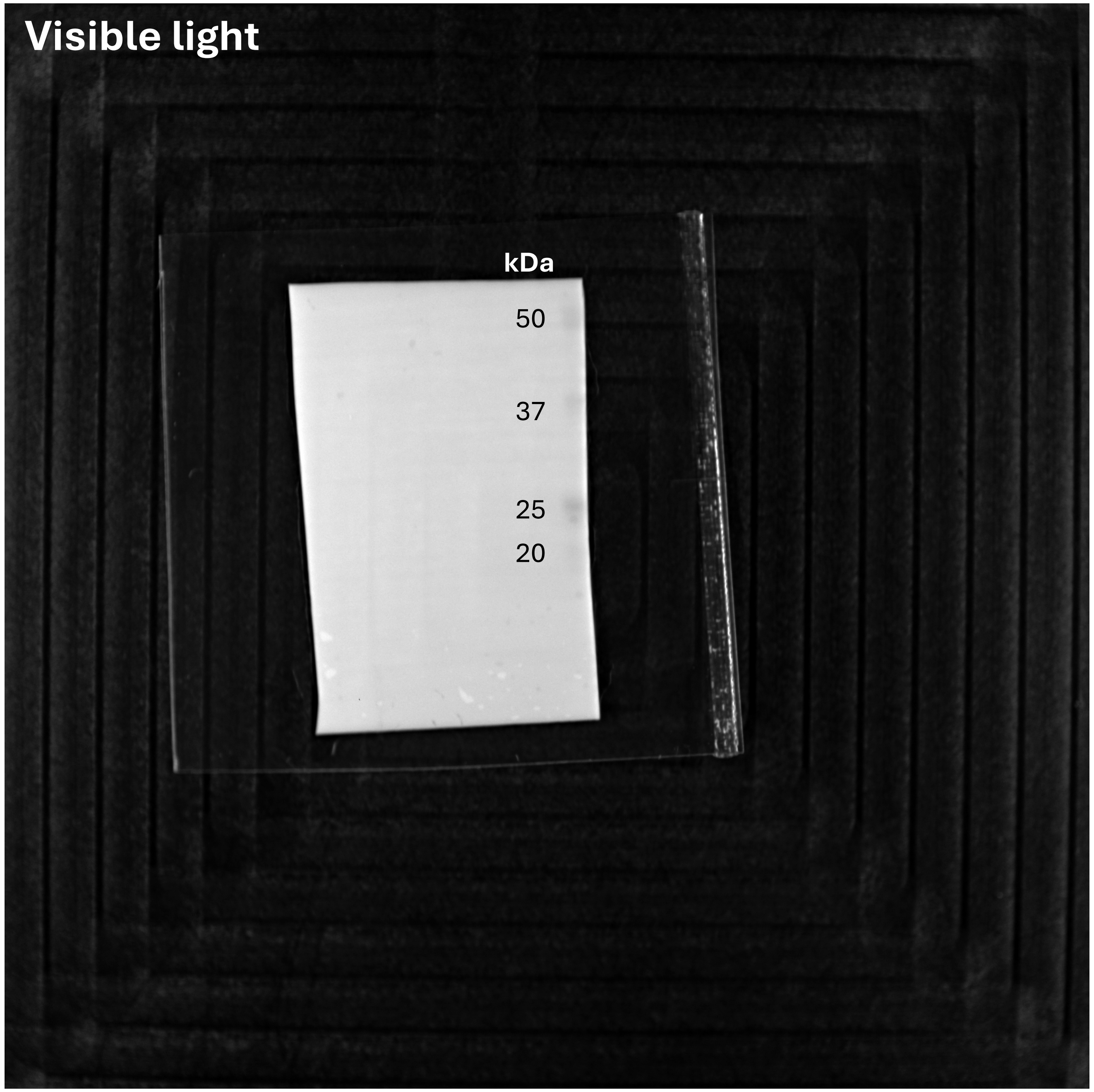

SourceDataSF3B_CD9

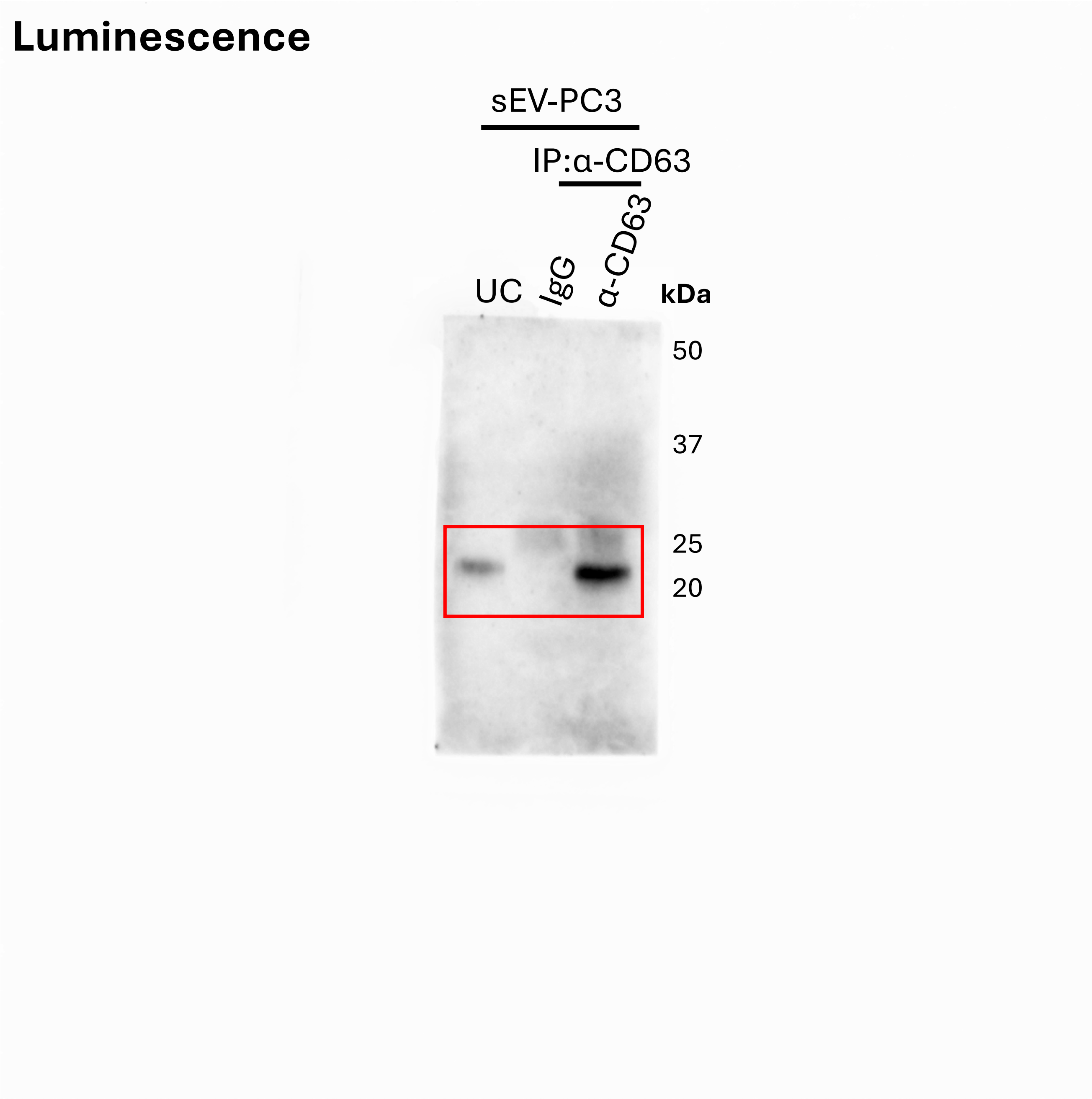

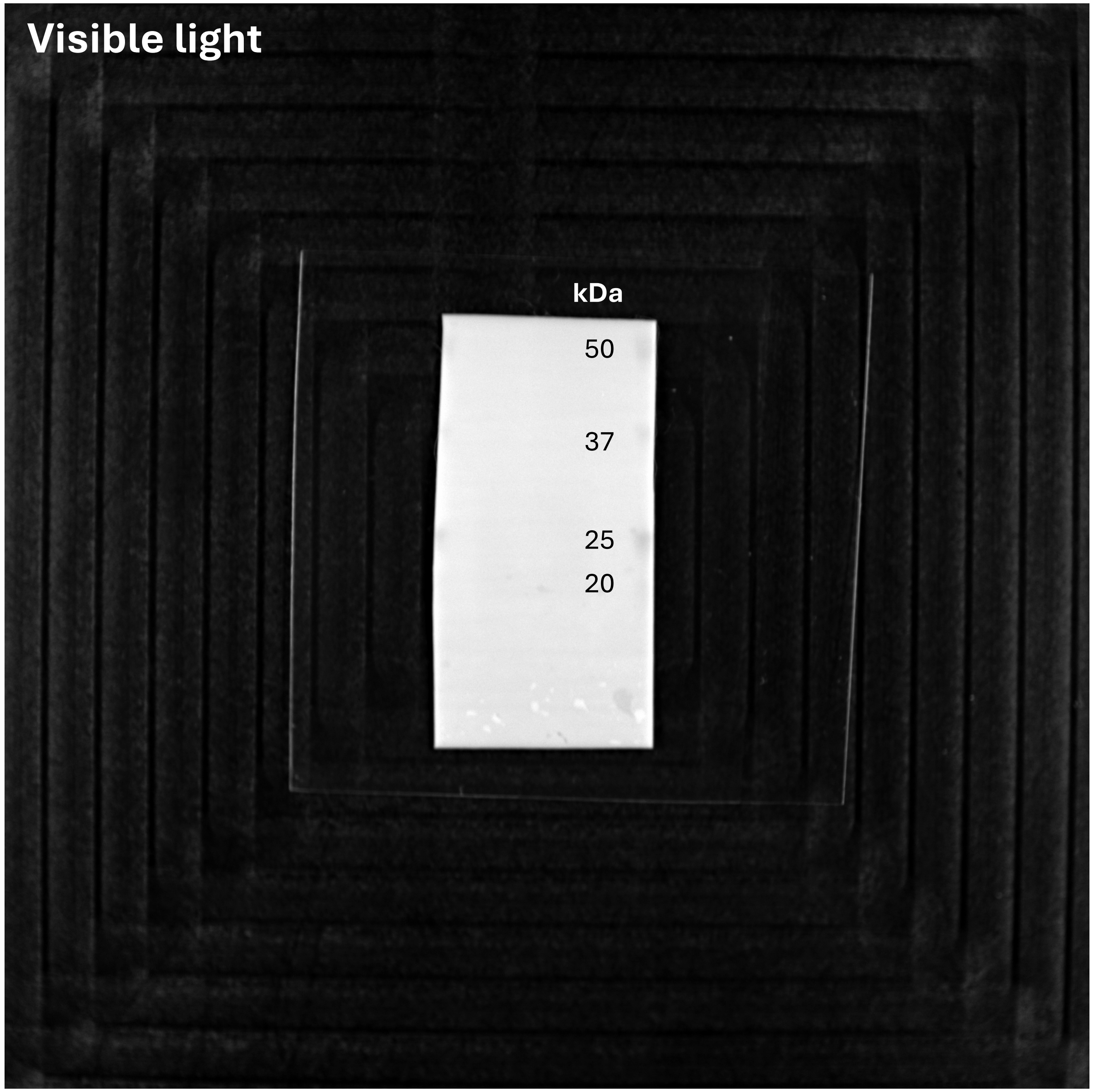

SourceDataSF3B_Fibronectin

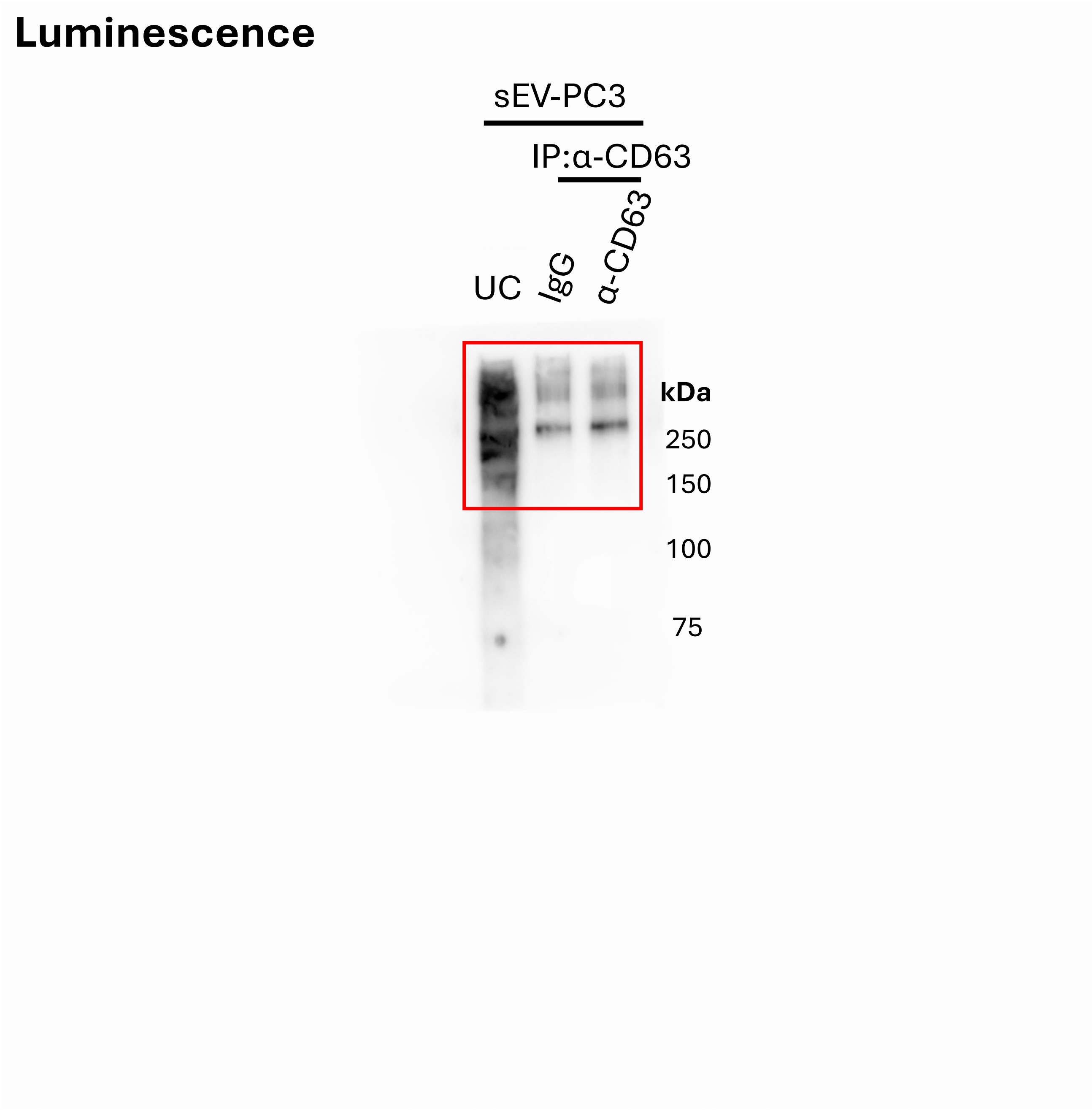

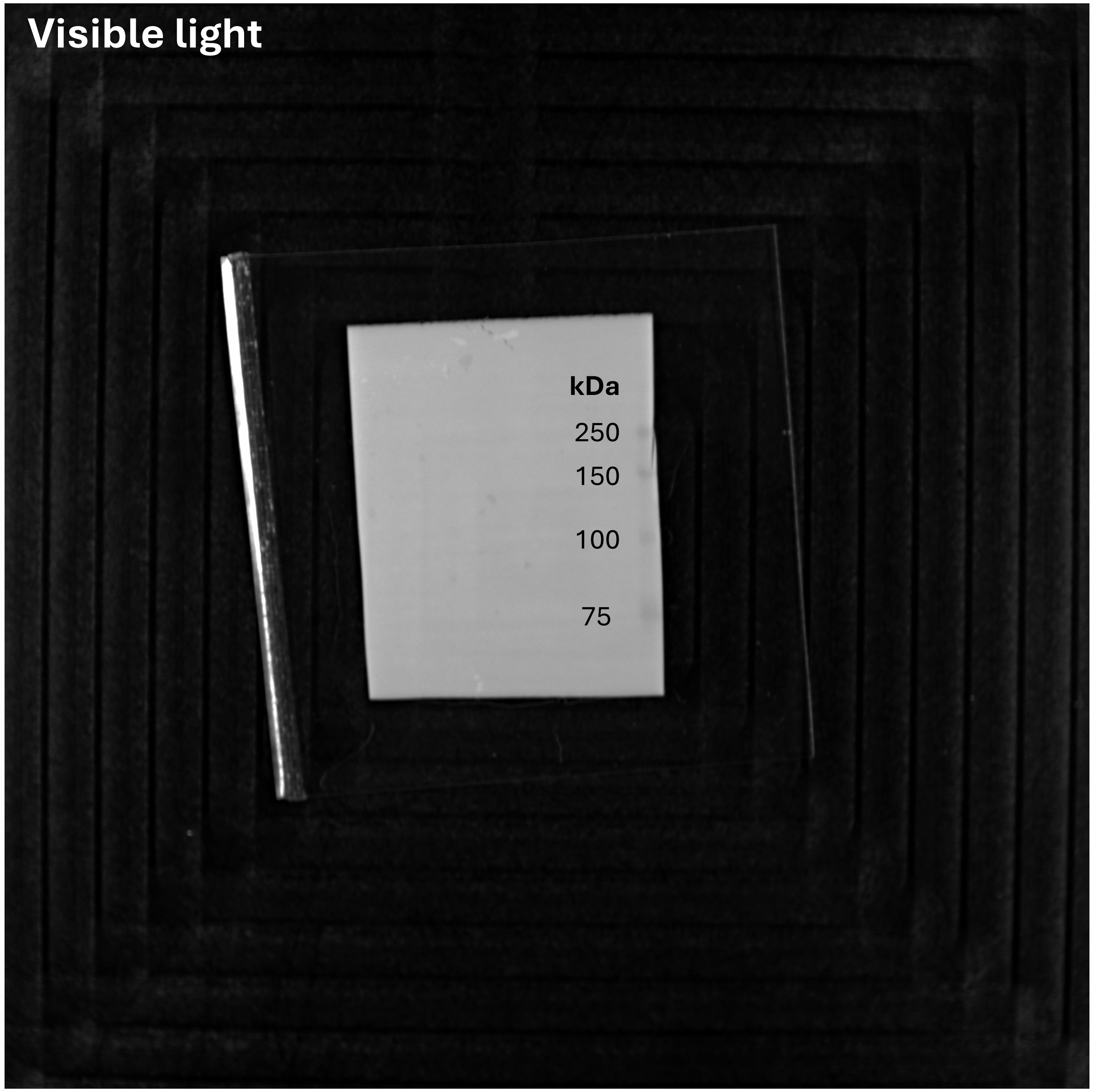

SourceDataSF3B_Laminin

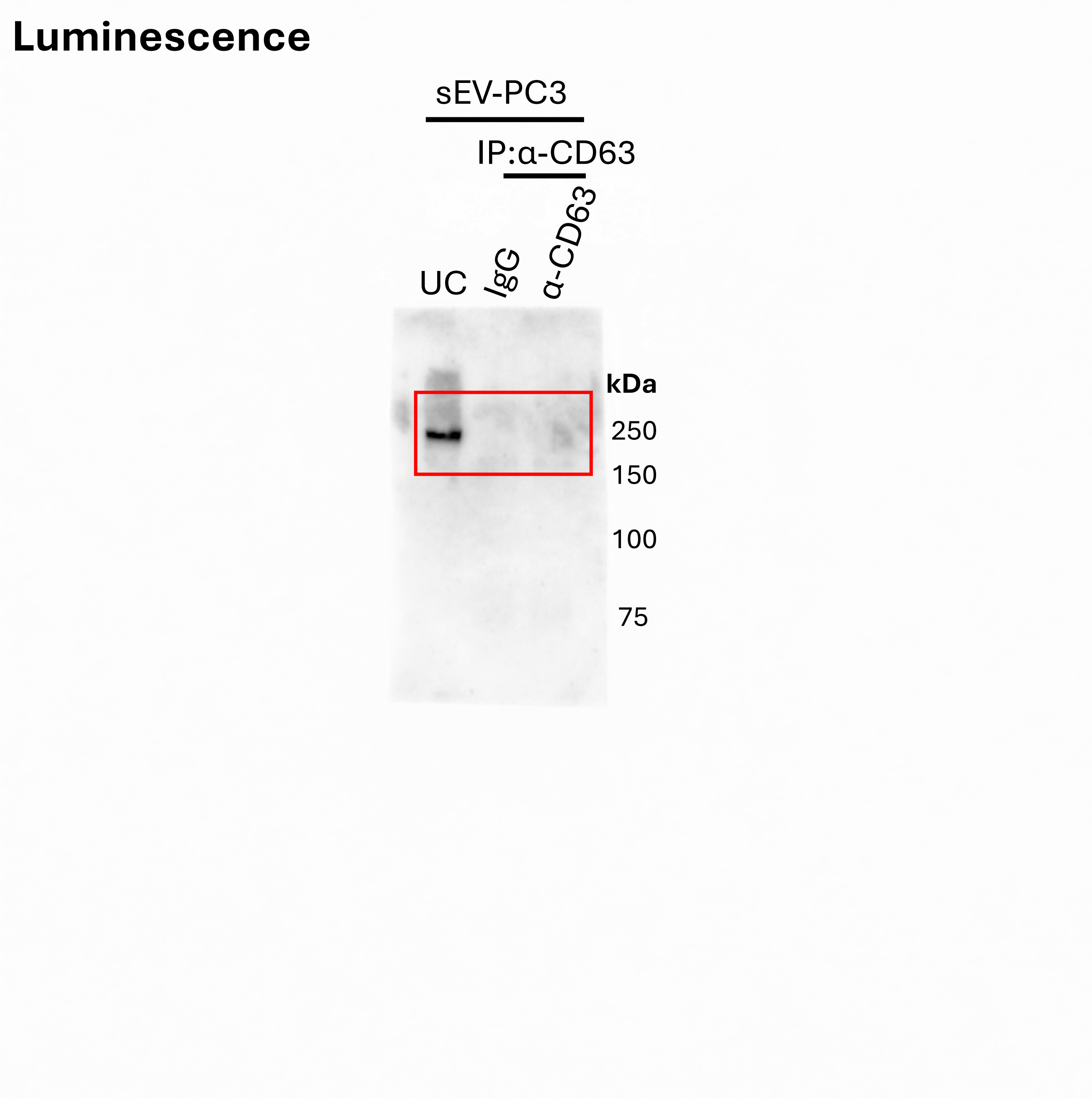

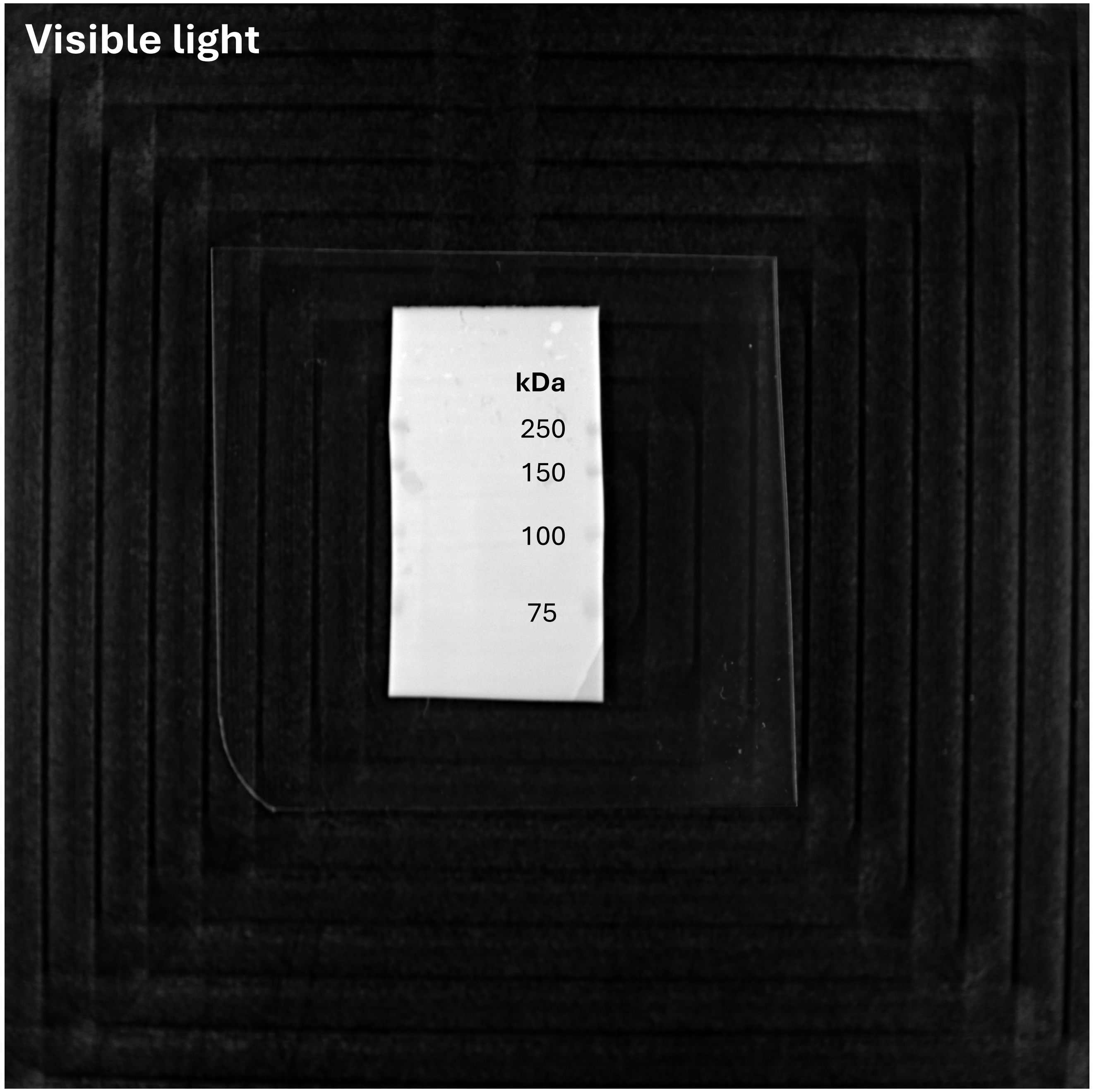

SourceDataSF3C_CD63

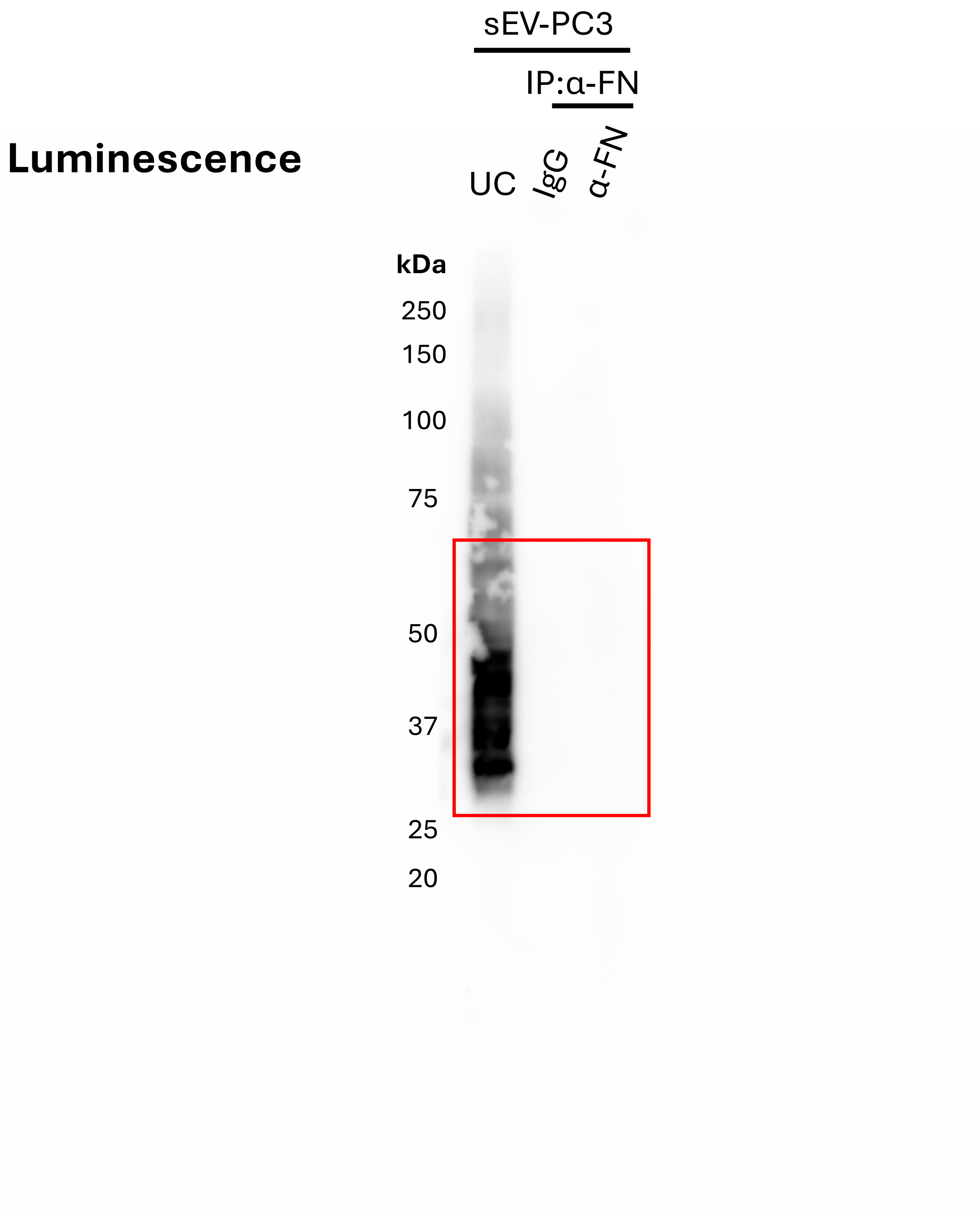

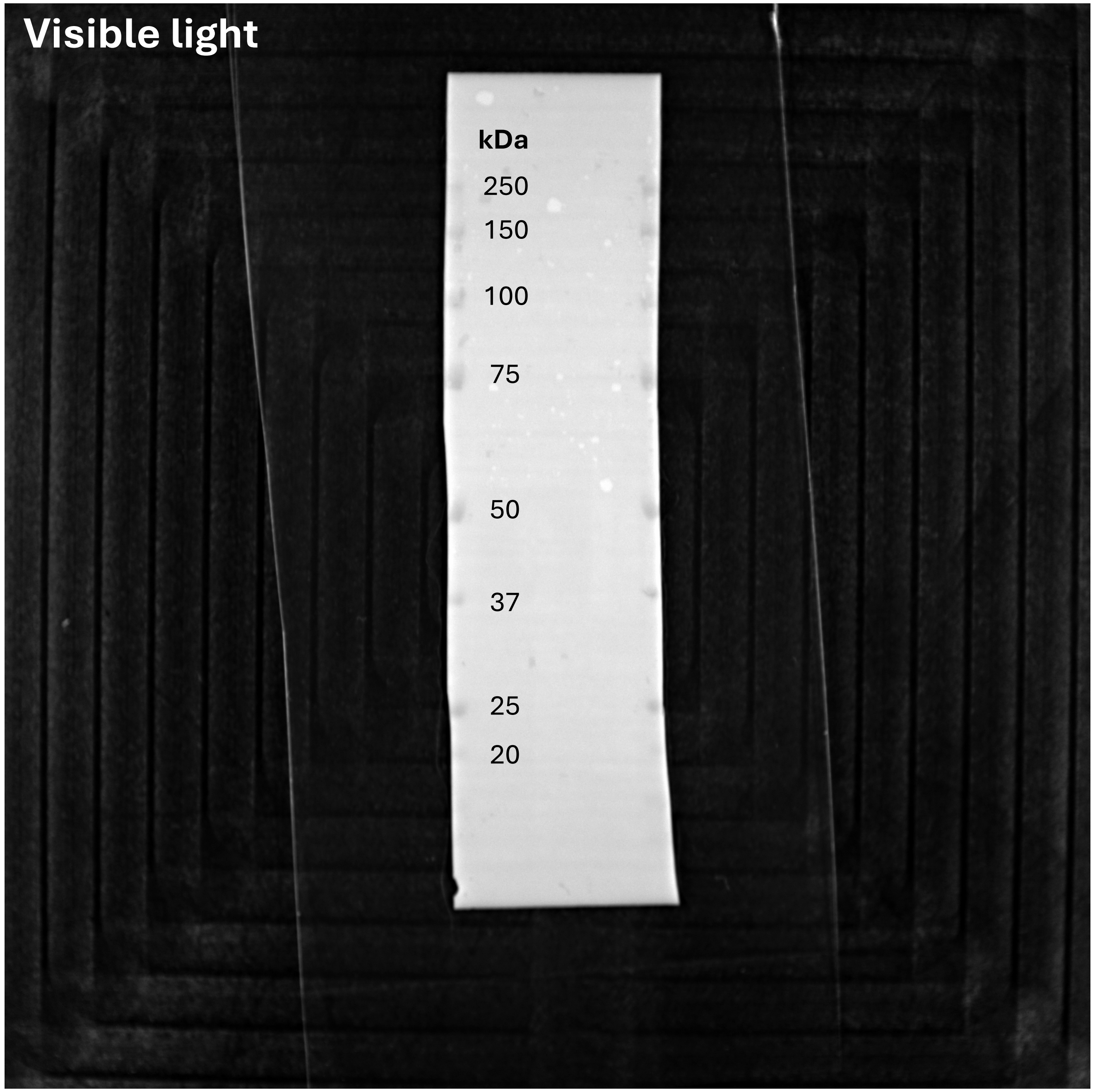

SourceDataSF3C_CD81

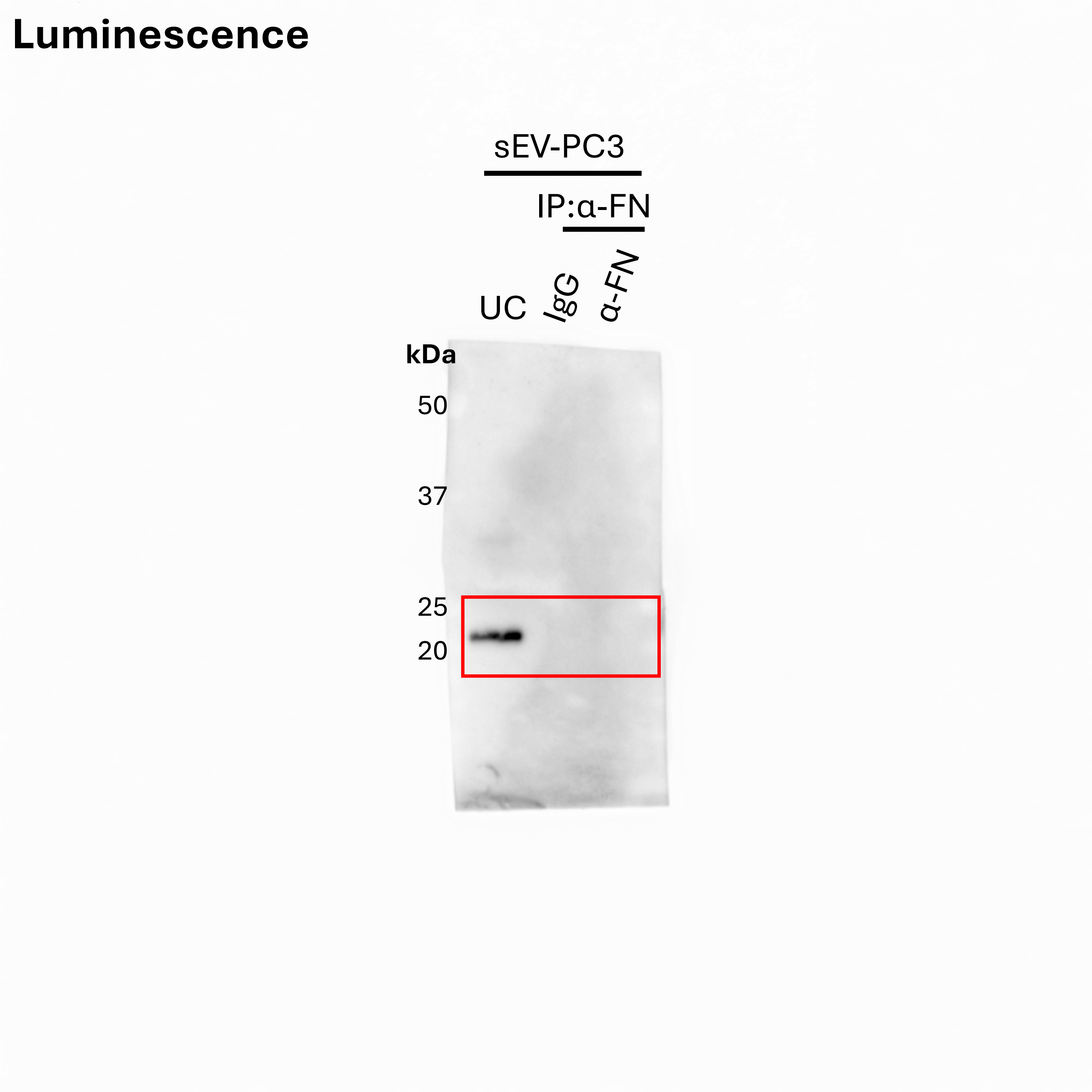

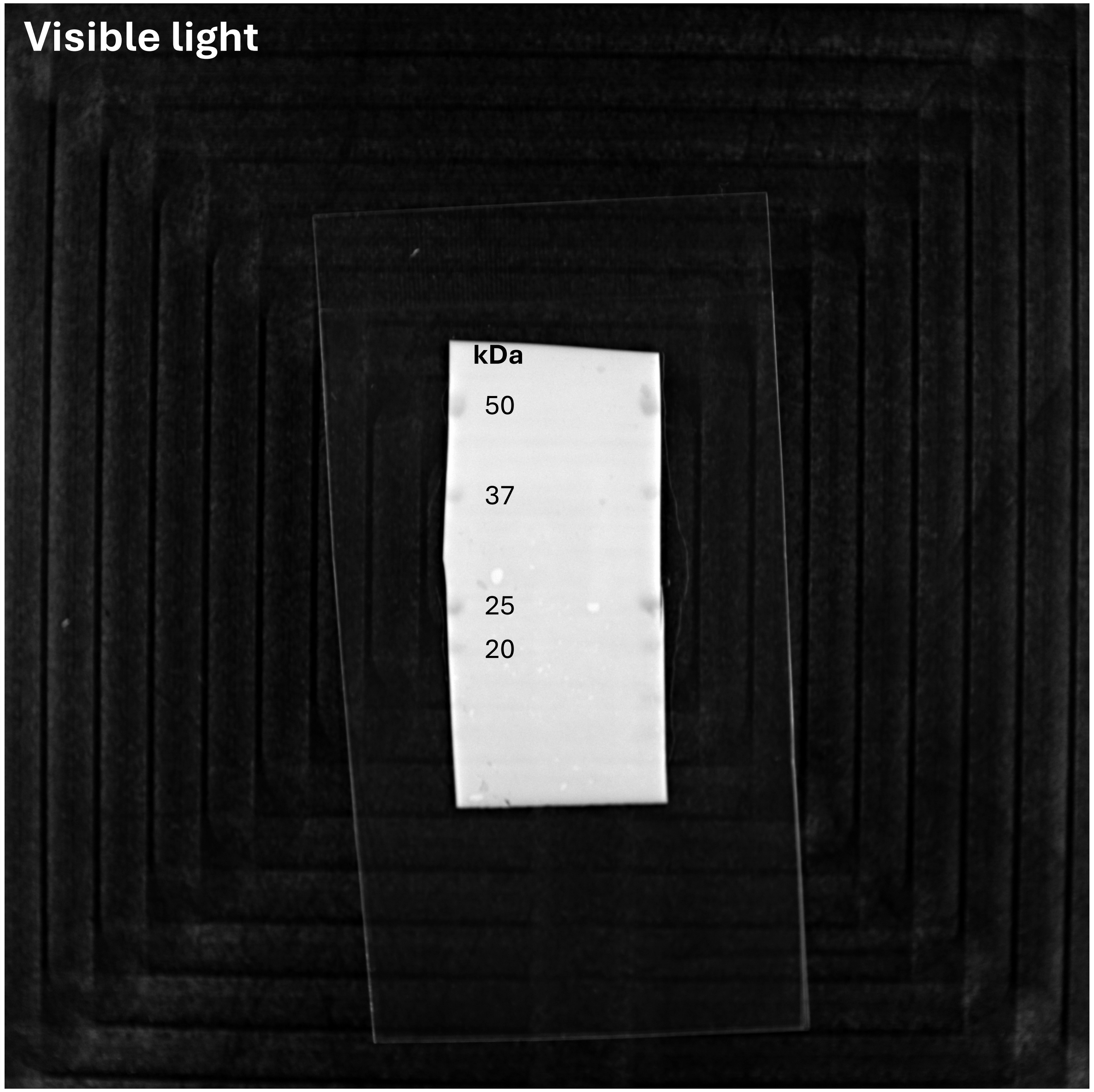

SourceDataSF3C_CD9

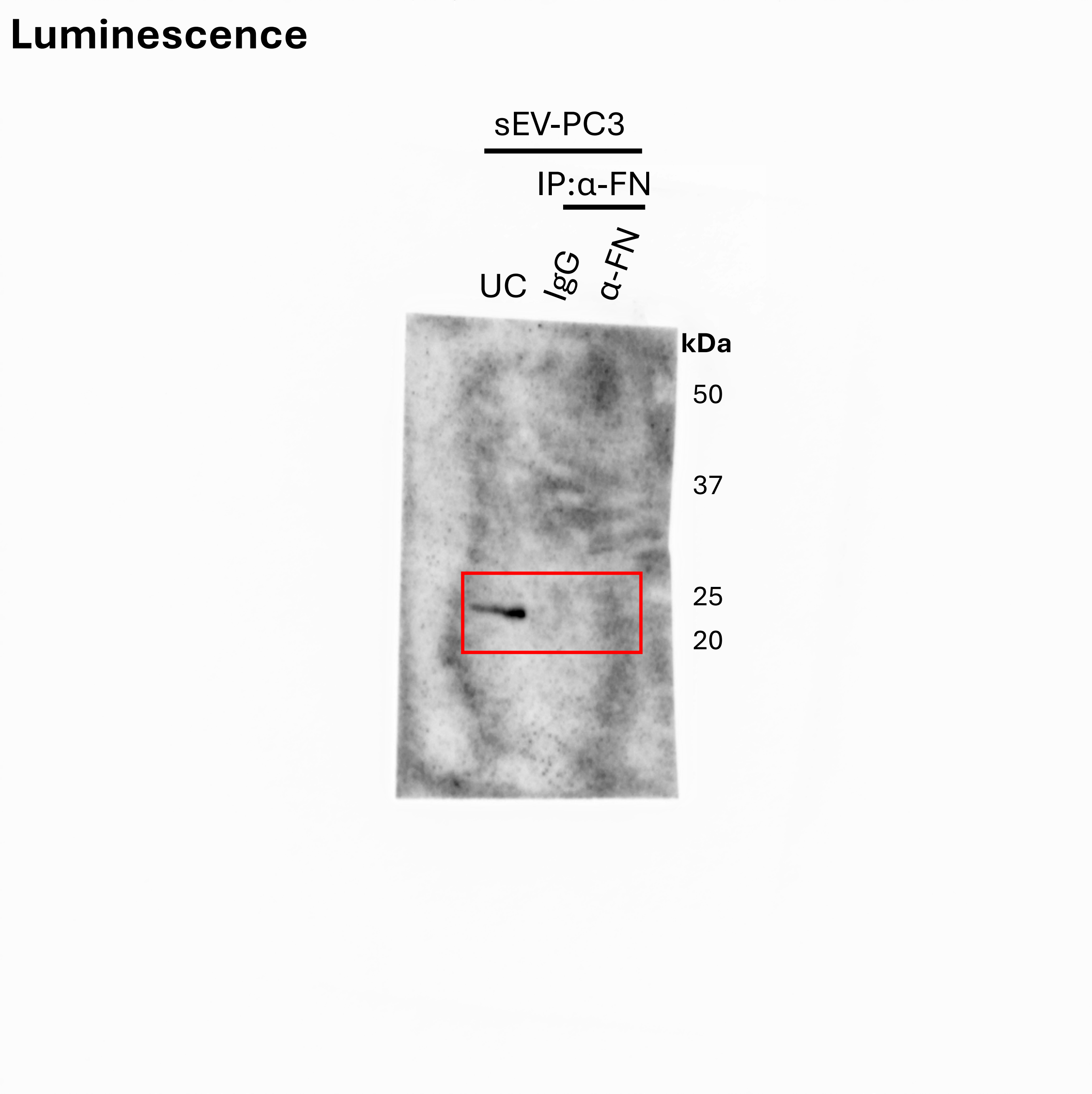

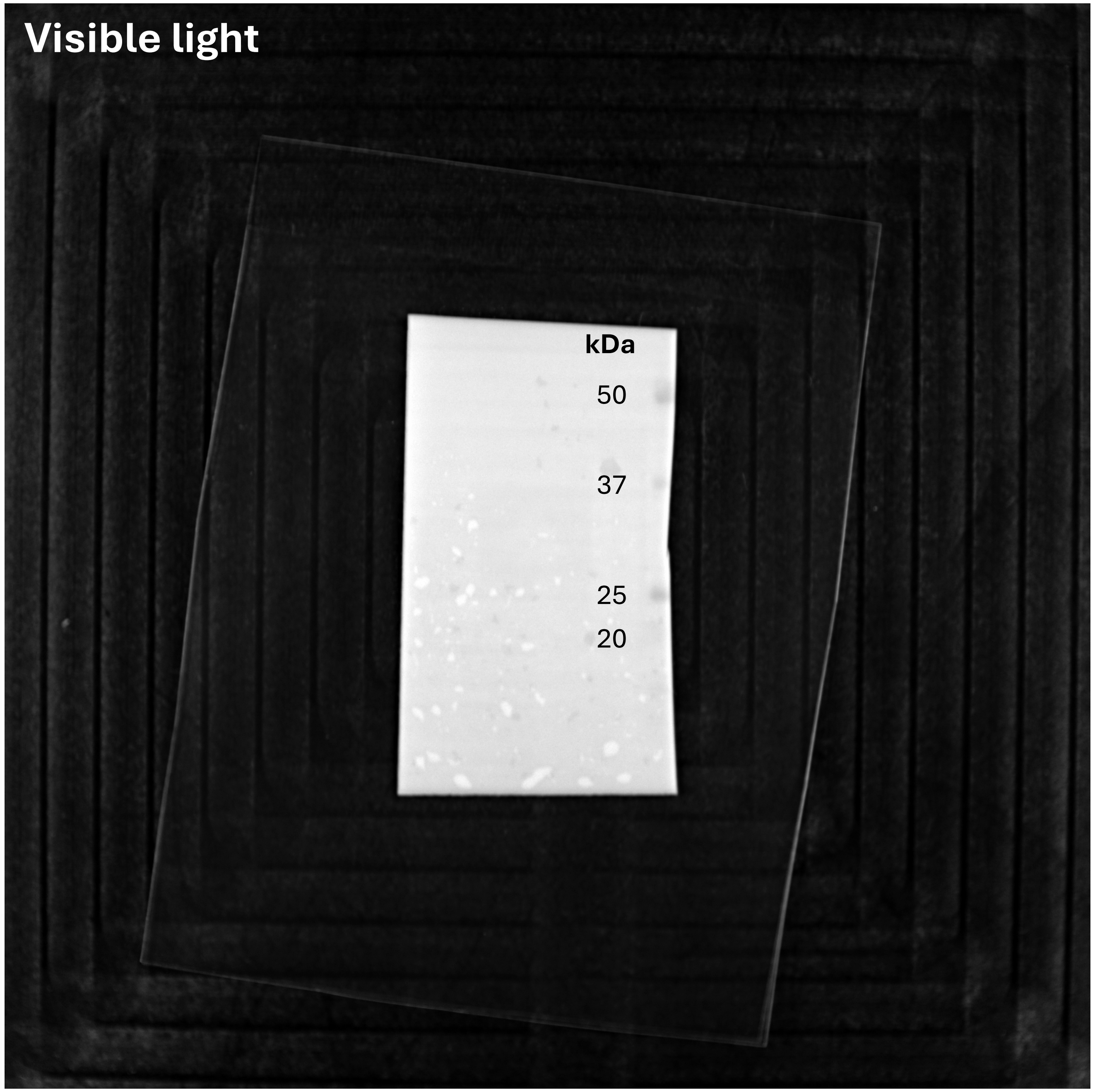

SourceDataSF3C_Fibronectin

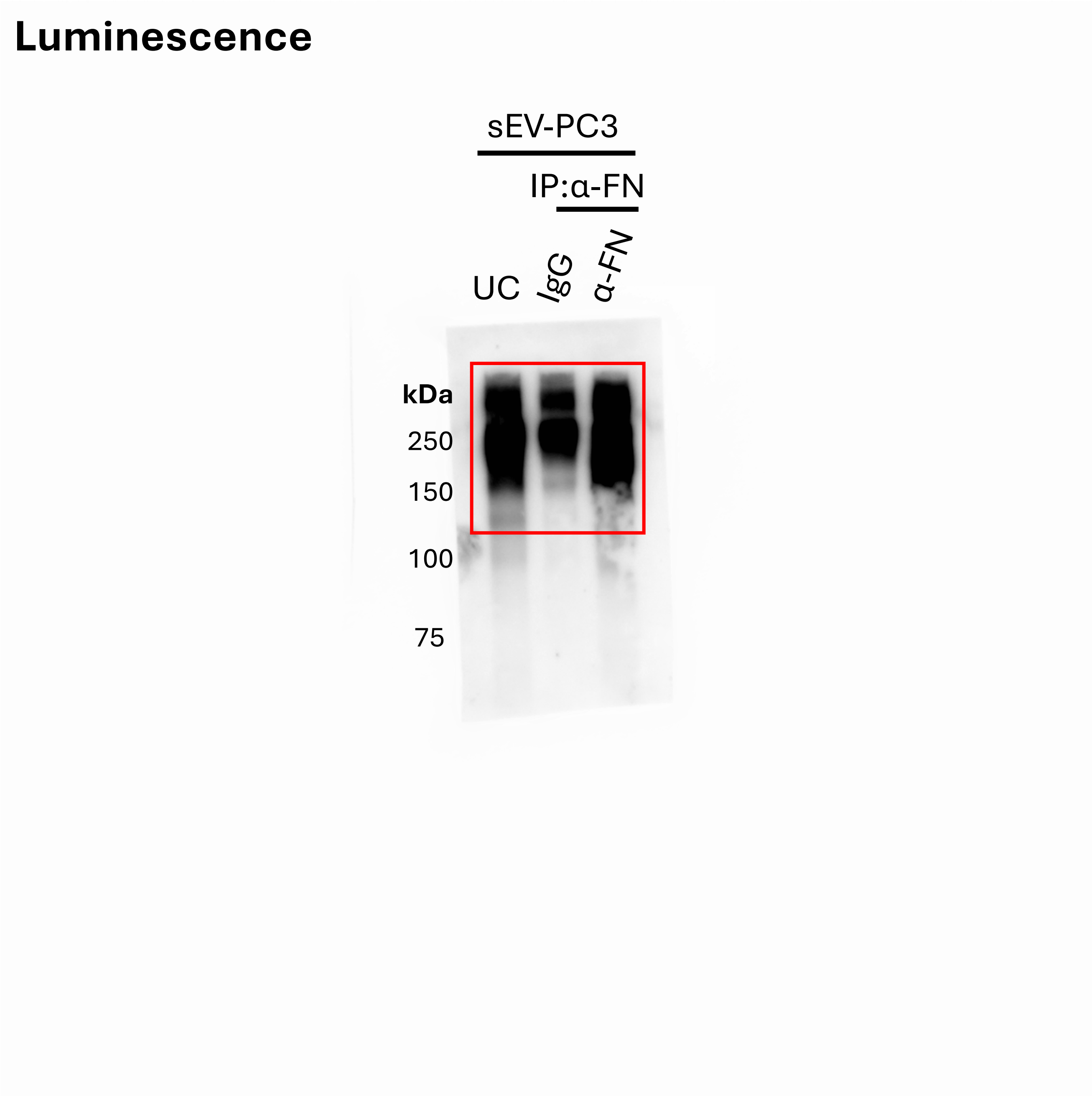

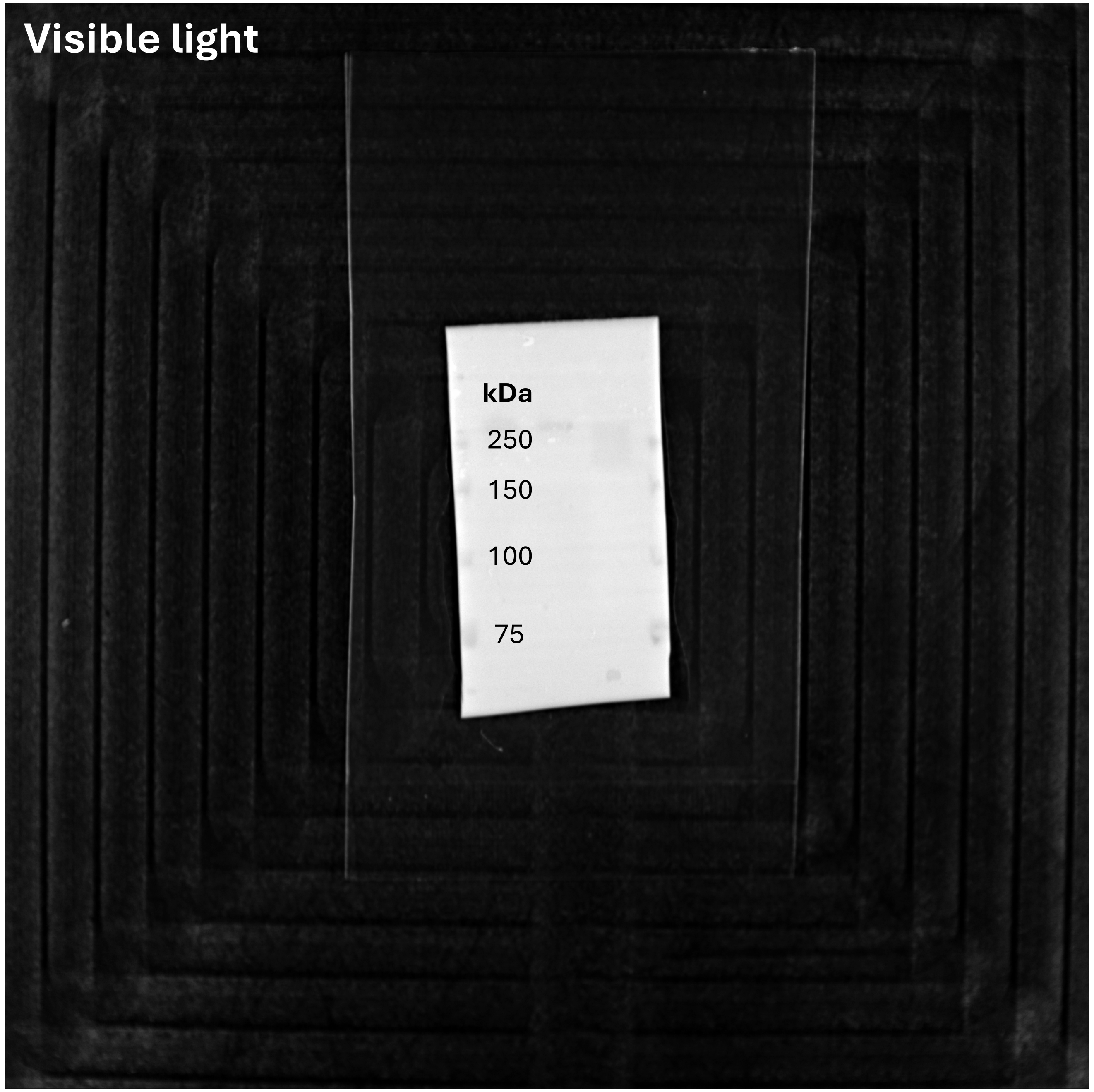

SourceDataSF3C_Laminin

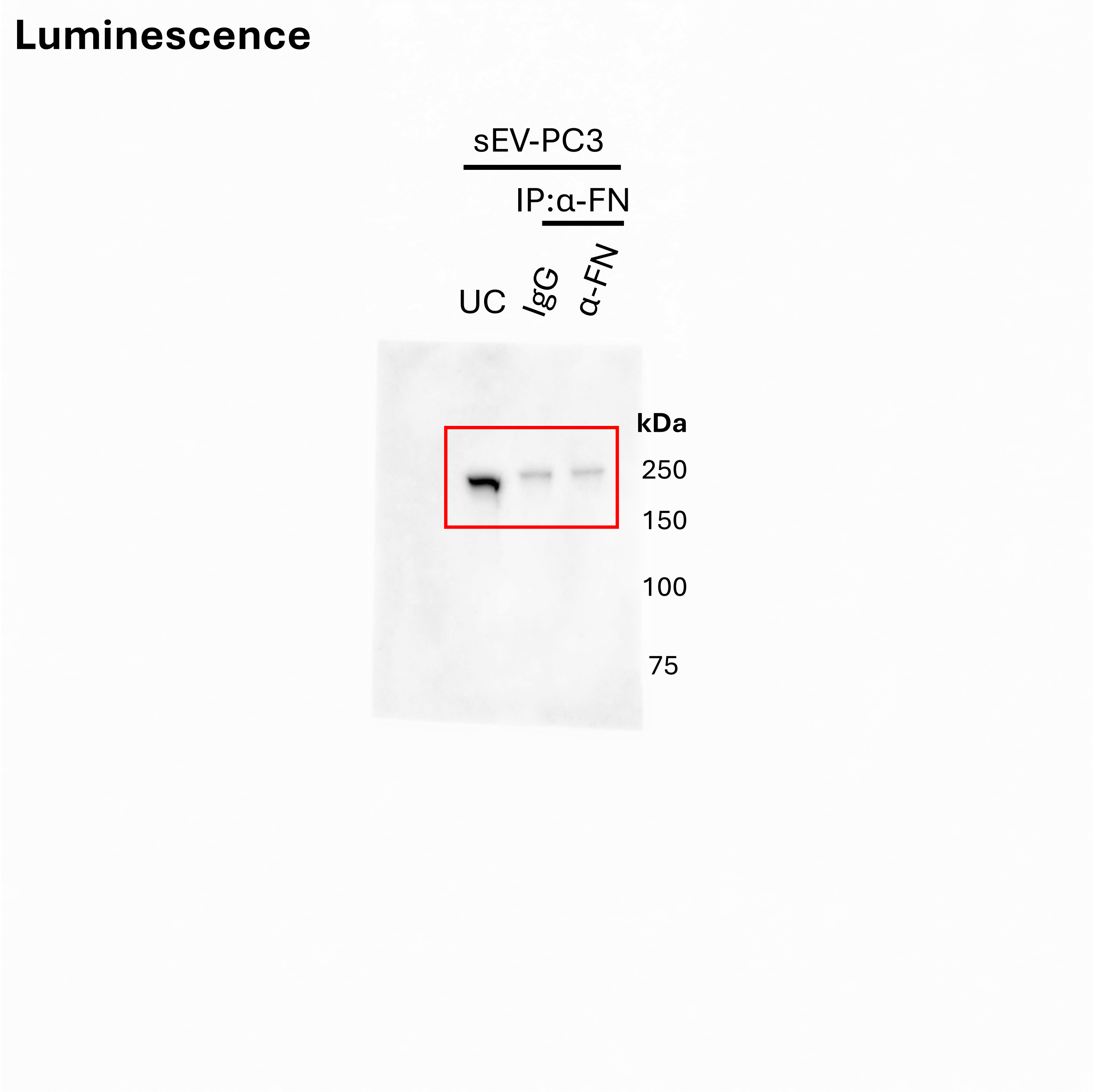

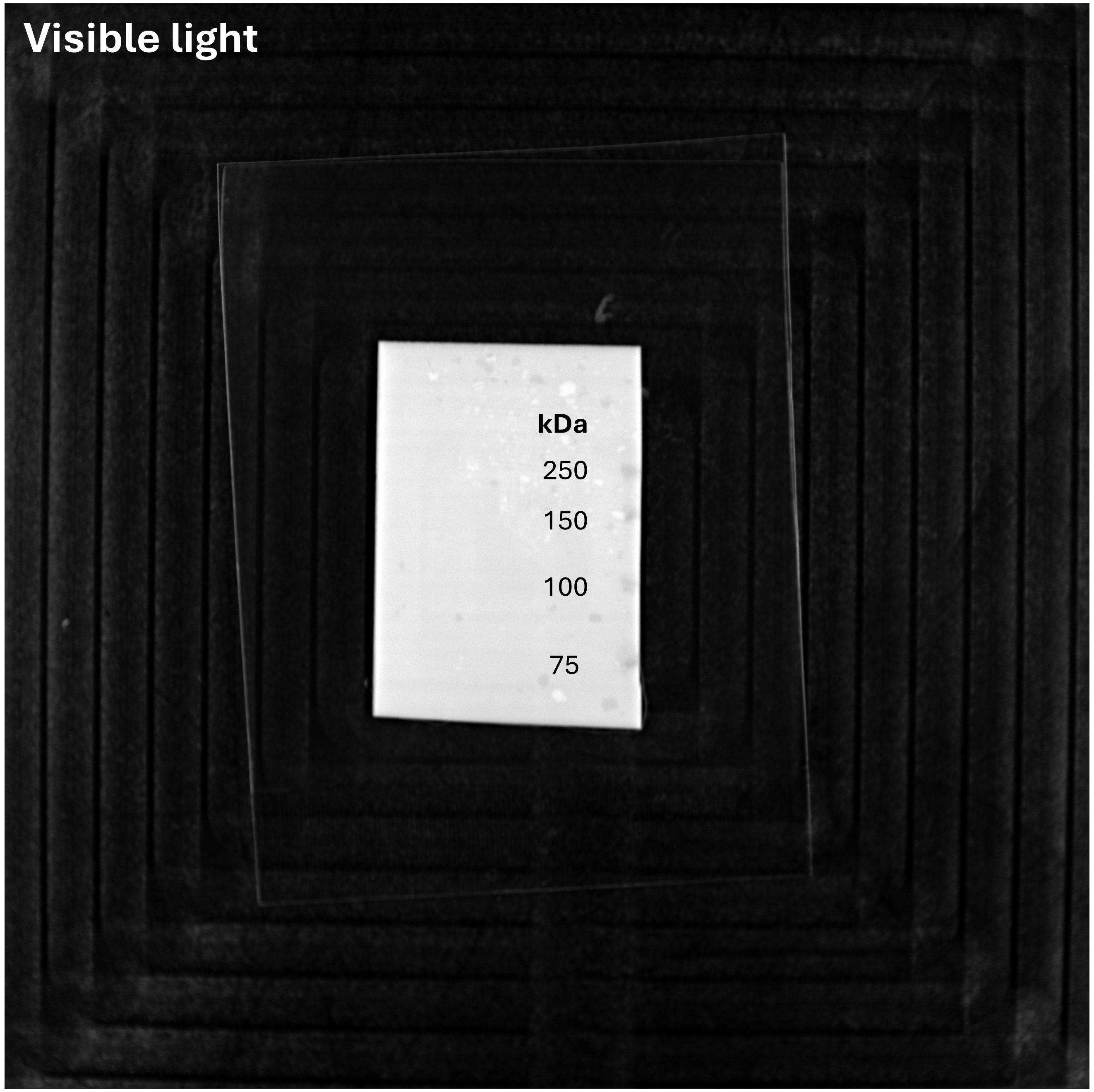

Supplement: SourceData FS3 — is the source file for Fig. S3. [file jcb_202404064_sourcedatafs3.pdf]
