# Supplementary material for: Extracellular vesicles adhere to cells primarily by interactions of integrins and GM1 with laminin
Source: J Cell Biol. 2025 Apr 30;224(6):e202404064. doi: 10.1083/jcb.202404064 (PMC12042775; doi:10.1083/jcb.202404064)

SupFig. 4B, 4C

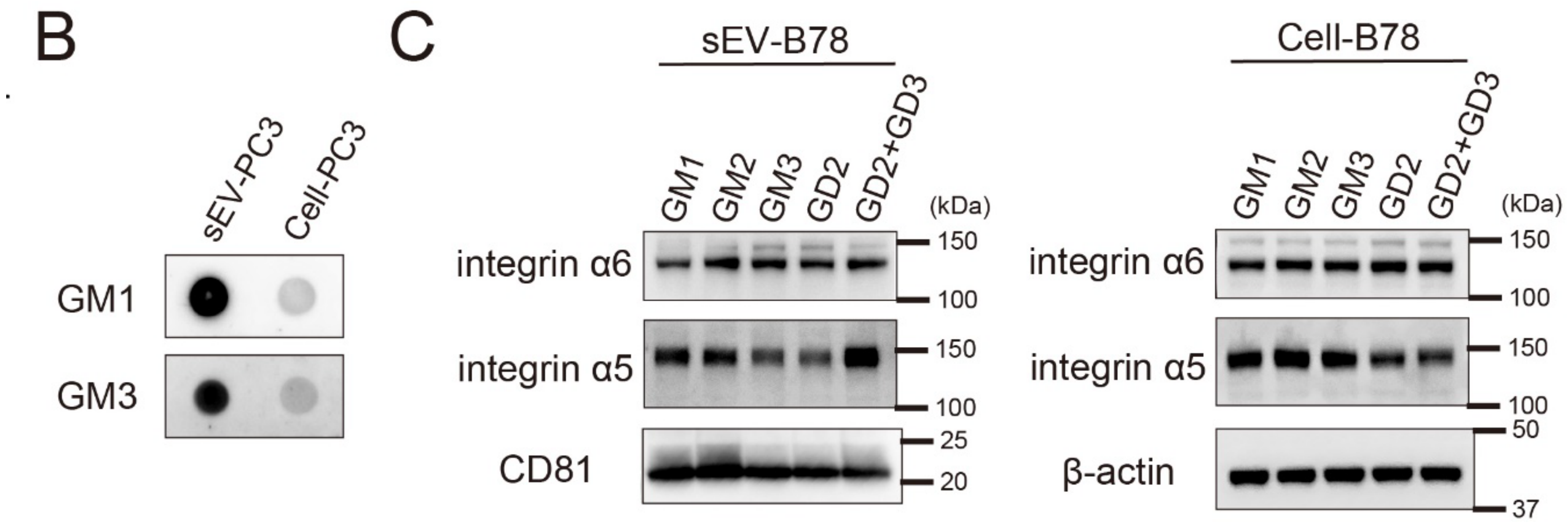

B
sEV-PC3
Cell-PC3
GM1
GM3
C
sEV-B78
Cell-B78
GM1
GM2
GM3
GD2
GD2+GD3
(kDa)
integrin α6
integrin α5
CD81
β-actin
150
100
25
20
50
37

SourceDataSF4B_Dot blot

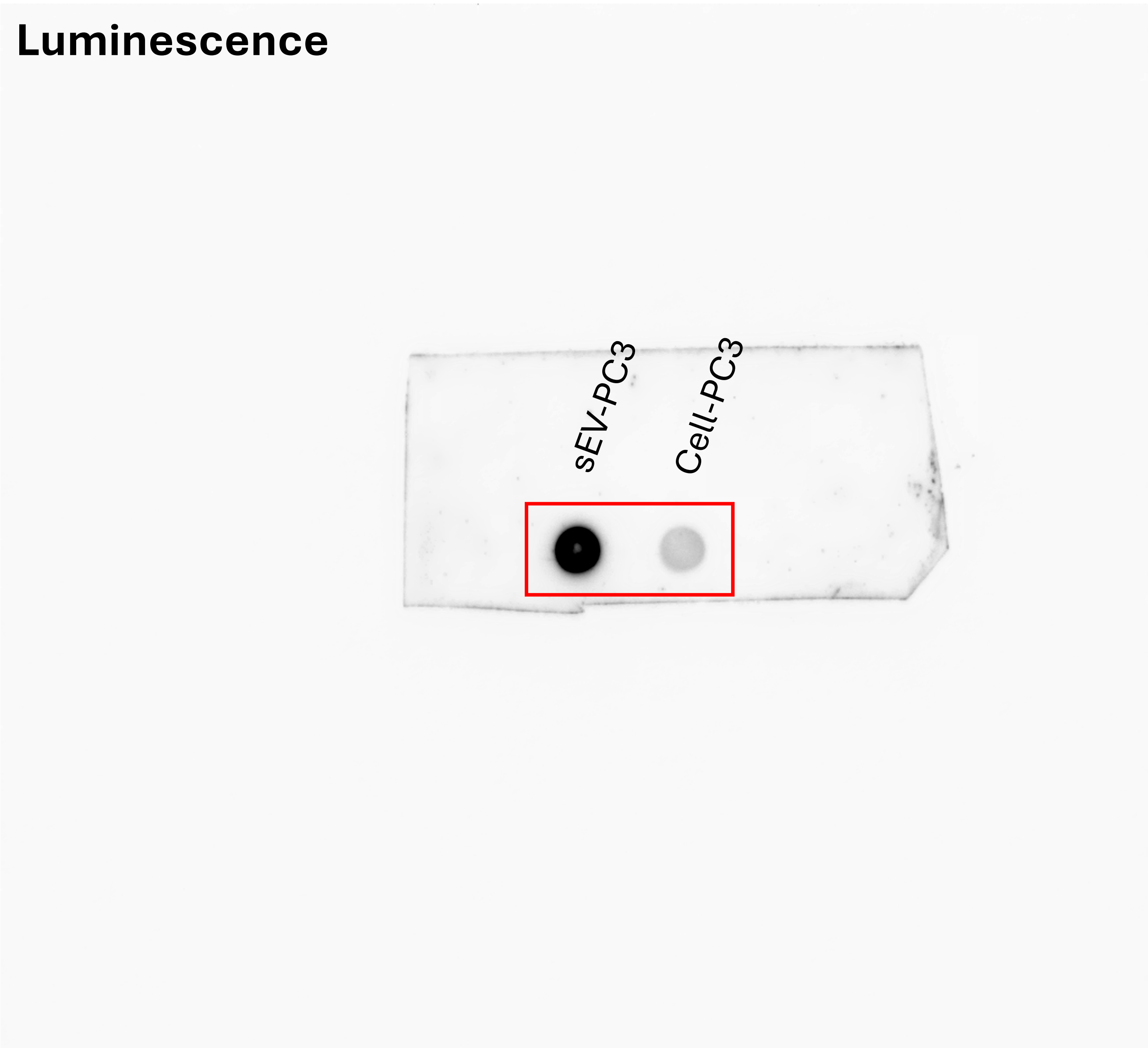

GM1(CTXB)

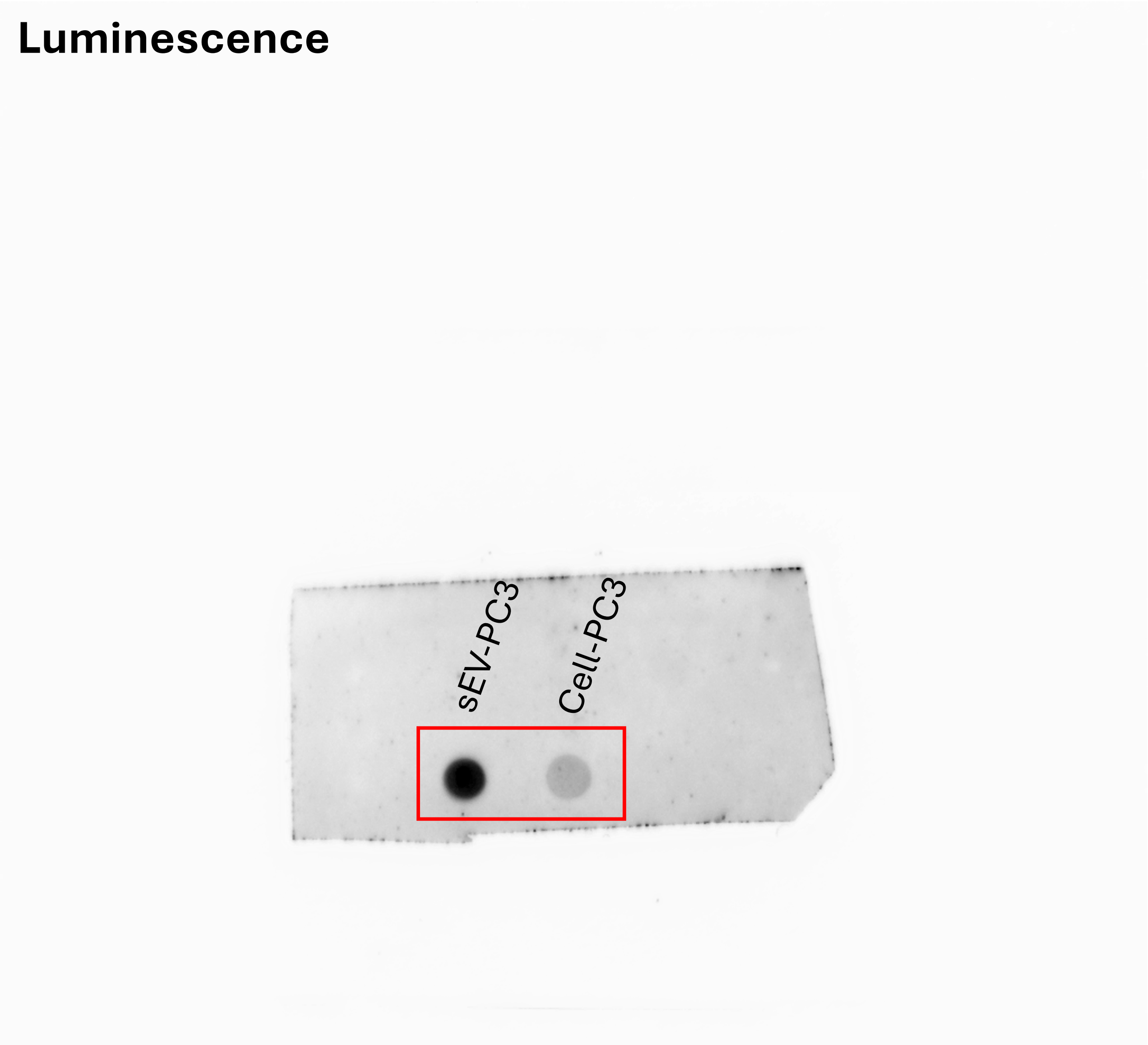

GM3(anti-GM3)

SourceDataSF4C_sEV-B78_integrin α6

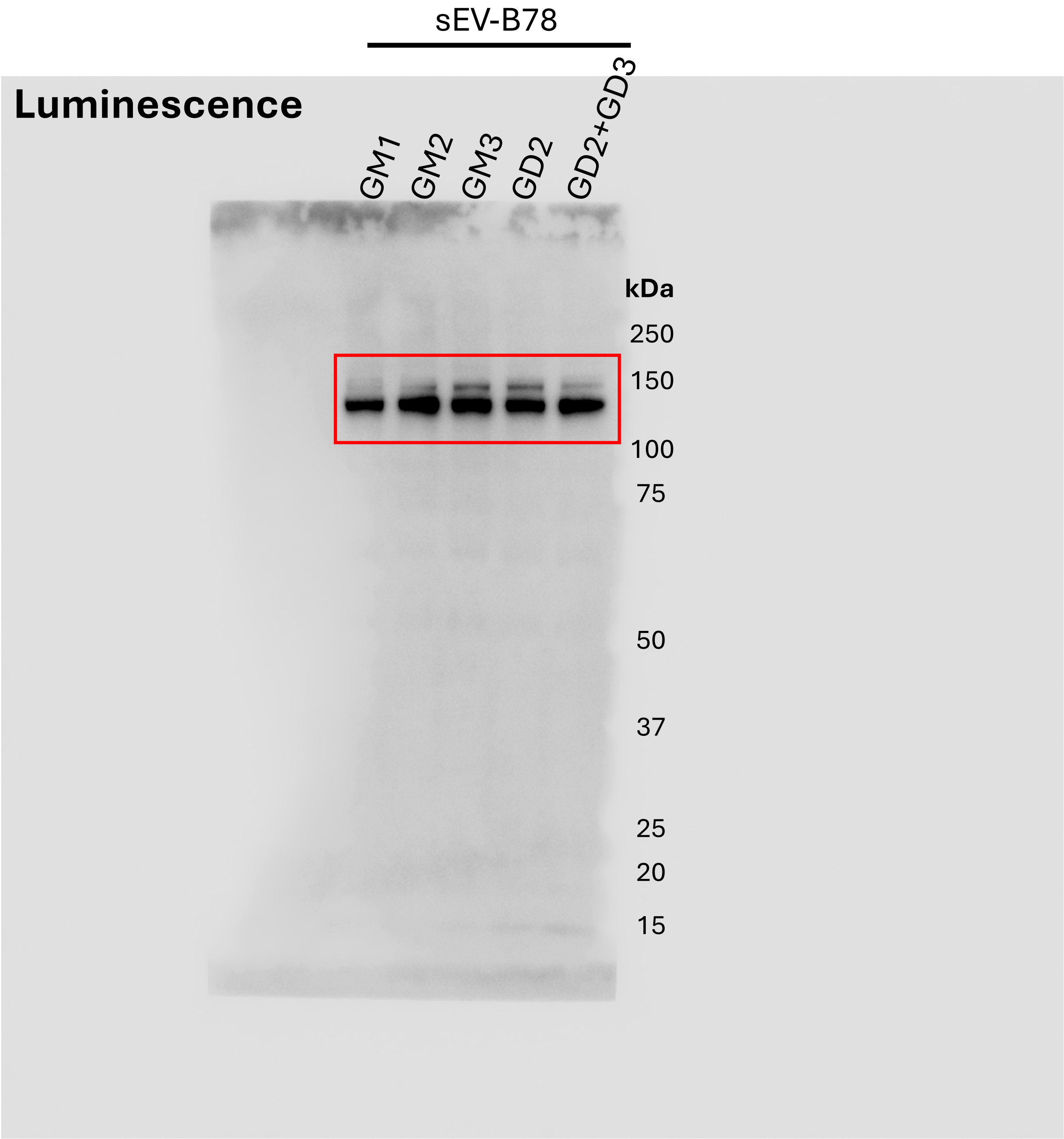

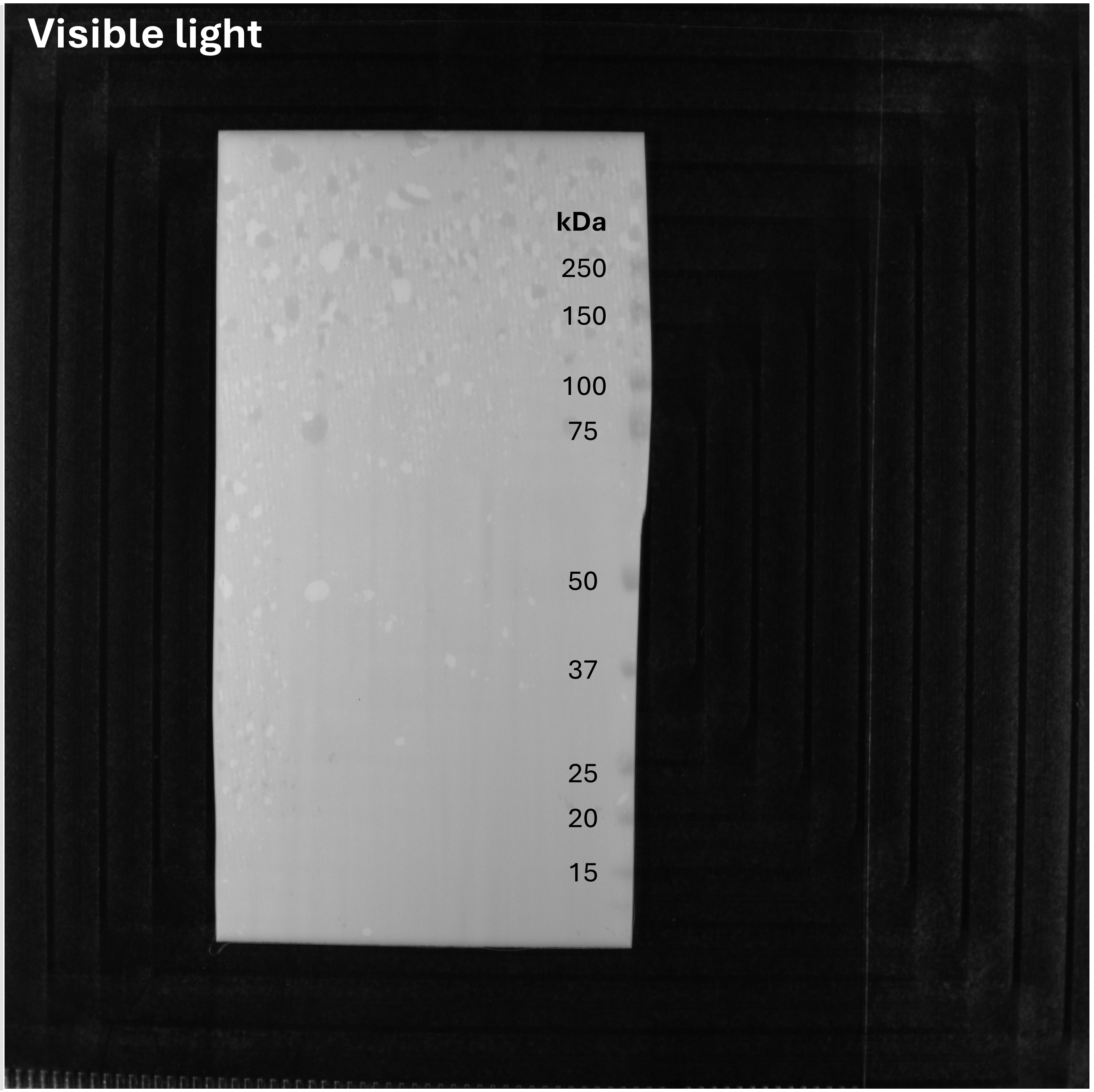

SourceDataSF4C_sEV-B78_integrin α5

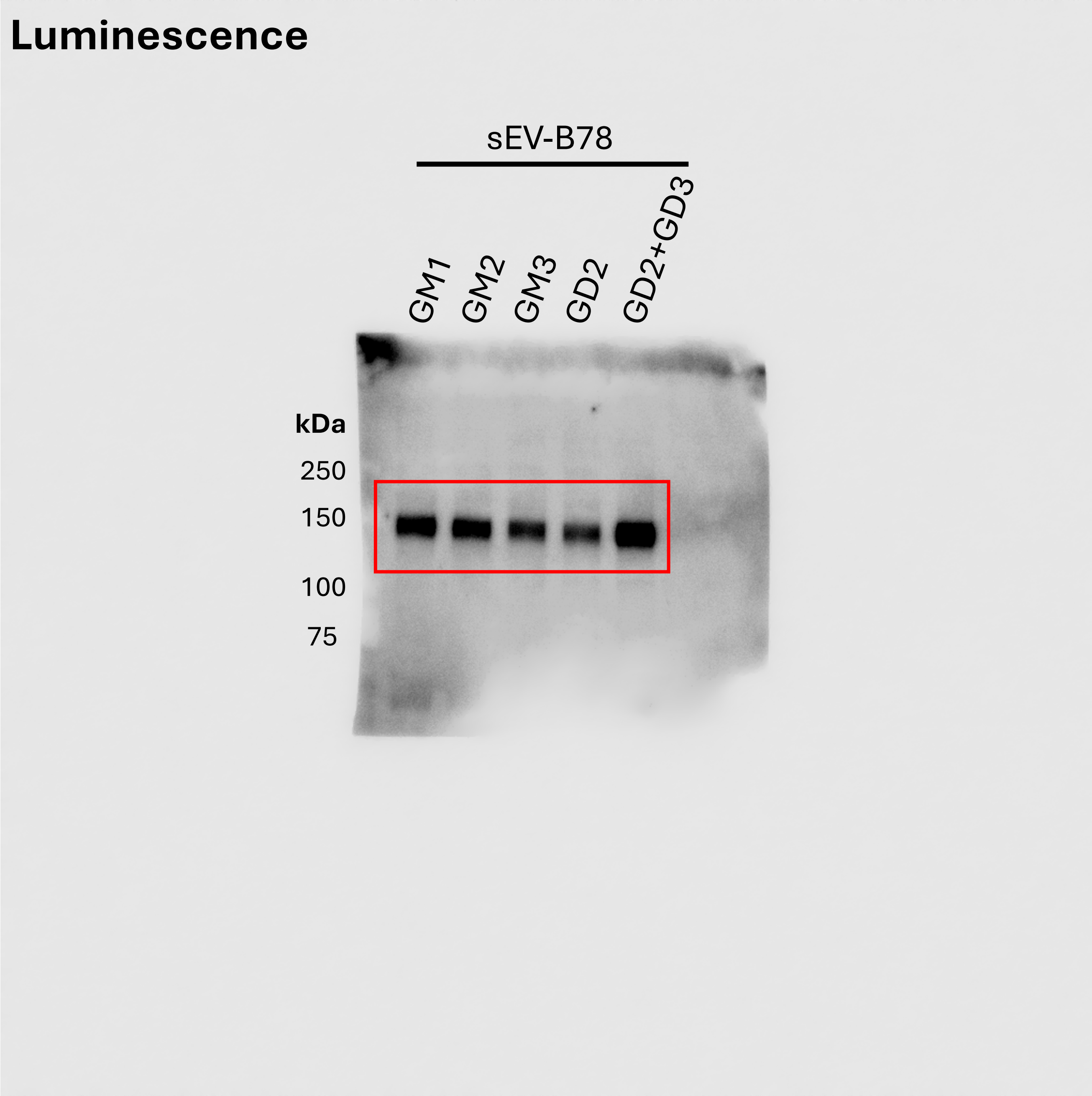

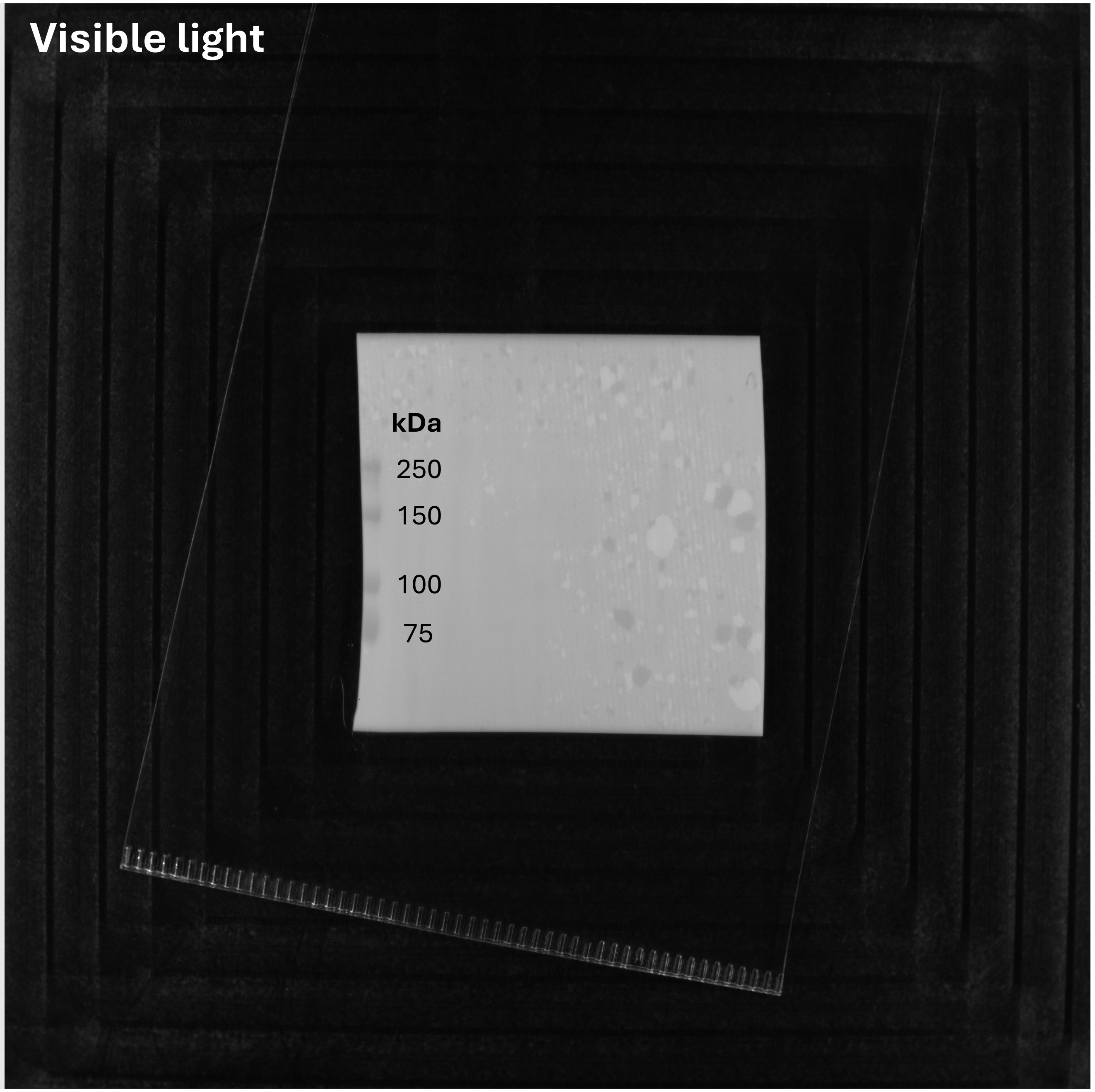

SourceDataSF4C_sEV-B78_CD81

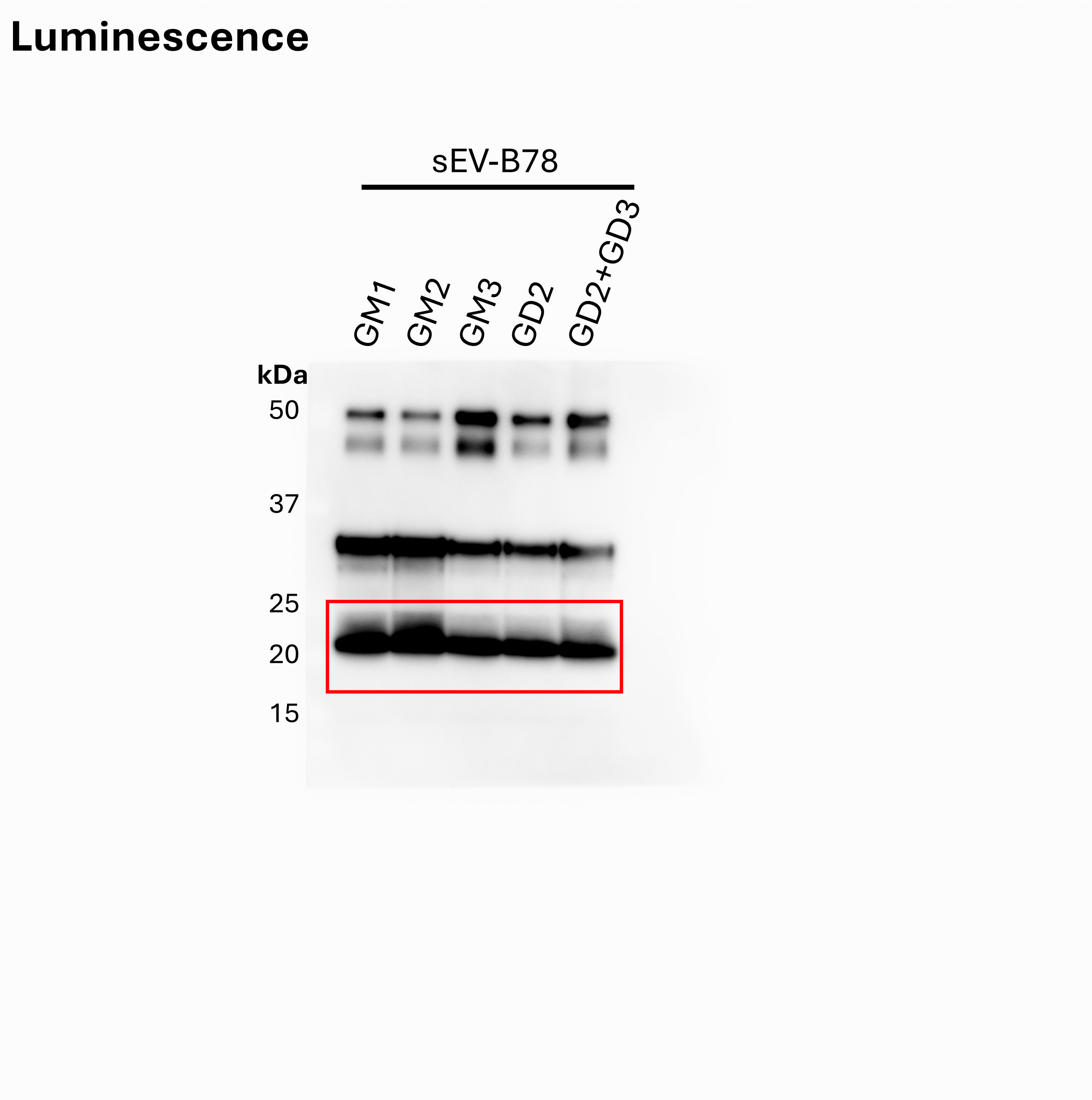

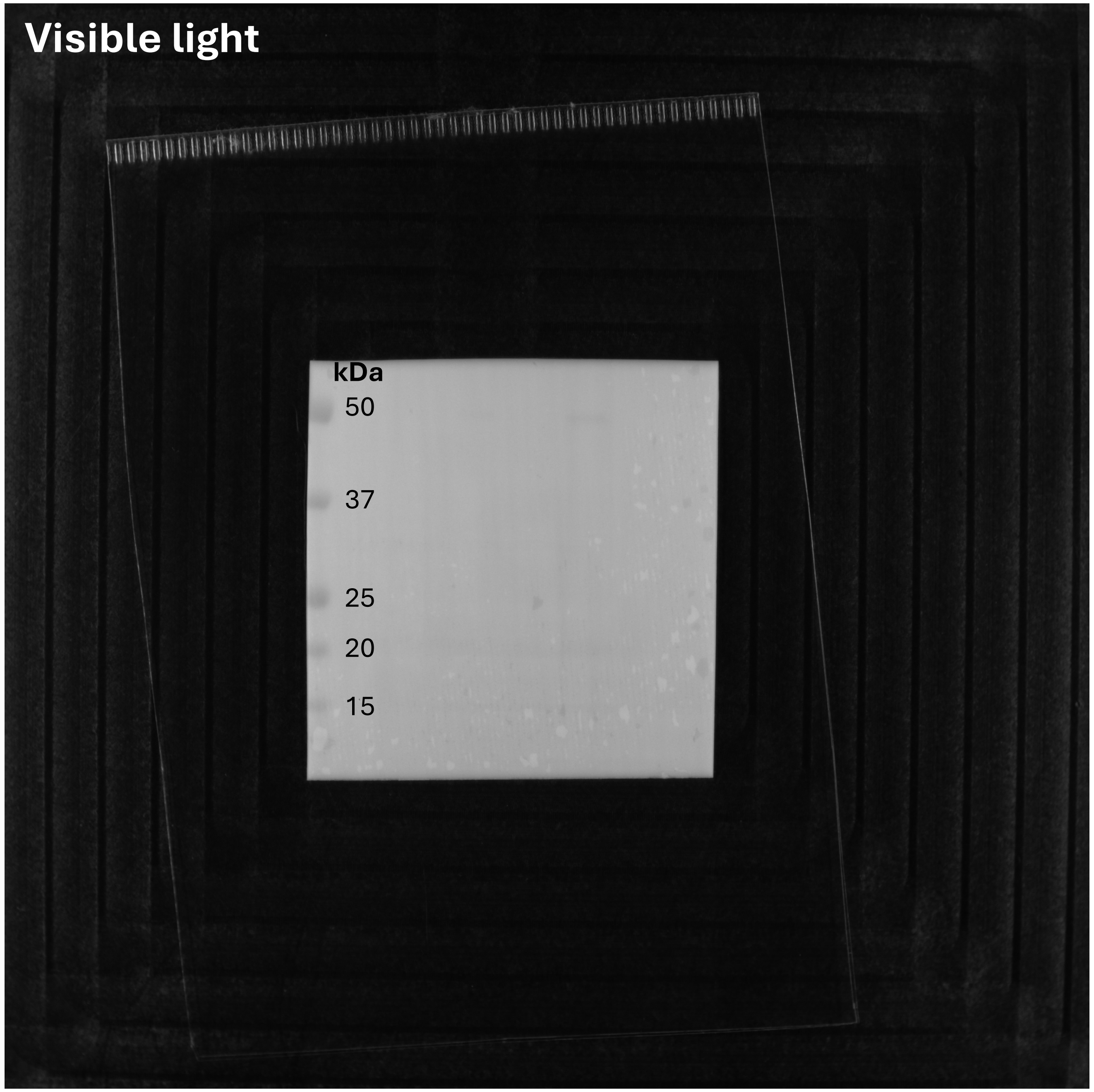

SourceDataSF4C_Cell-B78_integrin α6

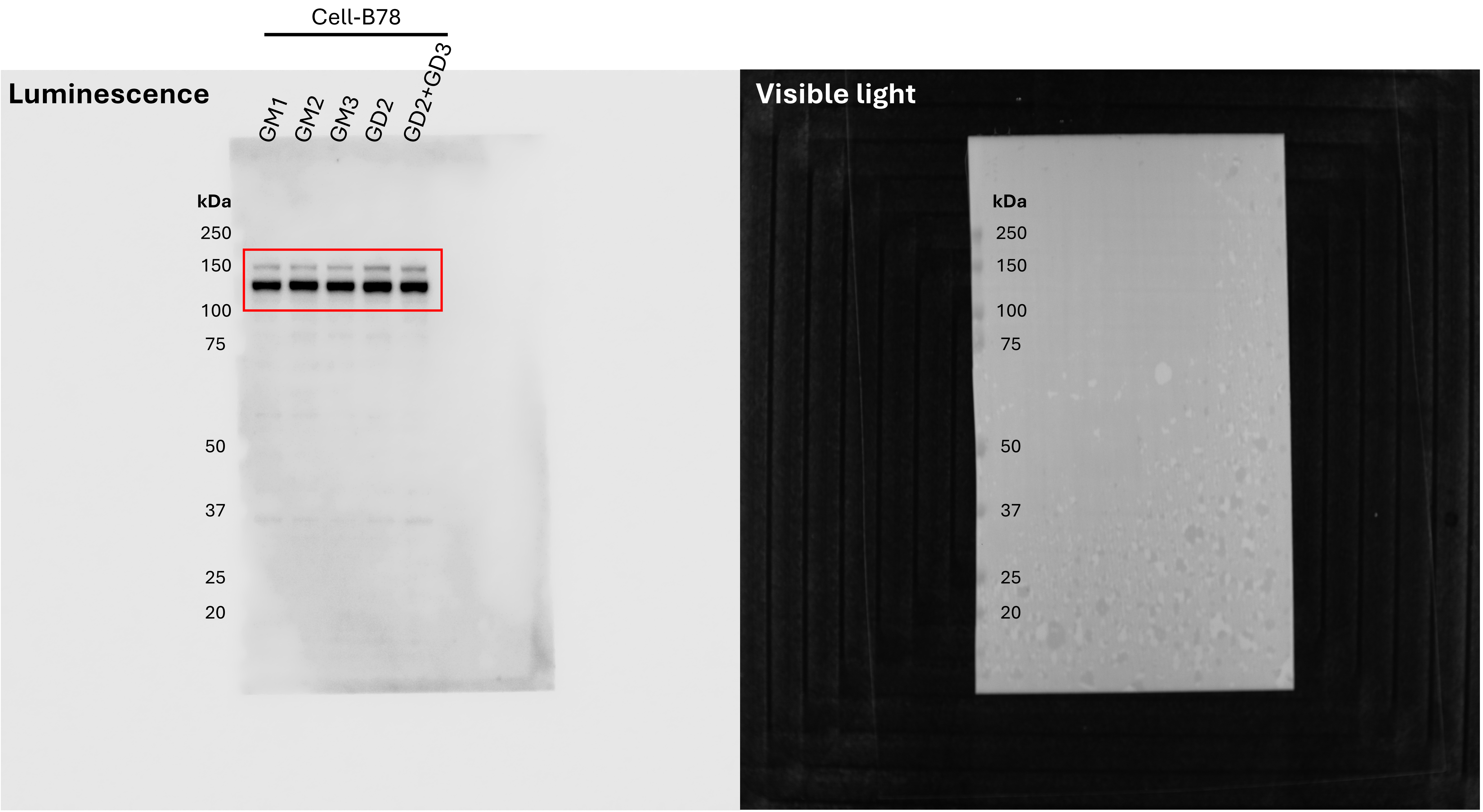

SourceDataSF4C_Cell-B78_integrin α5

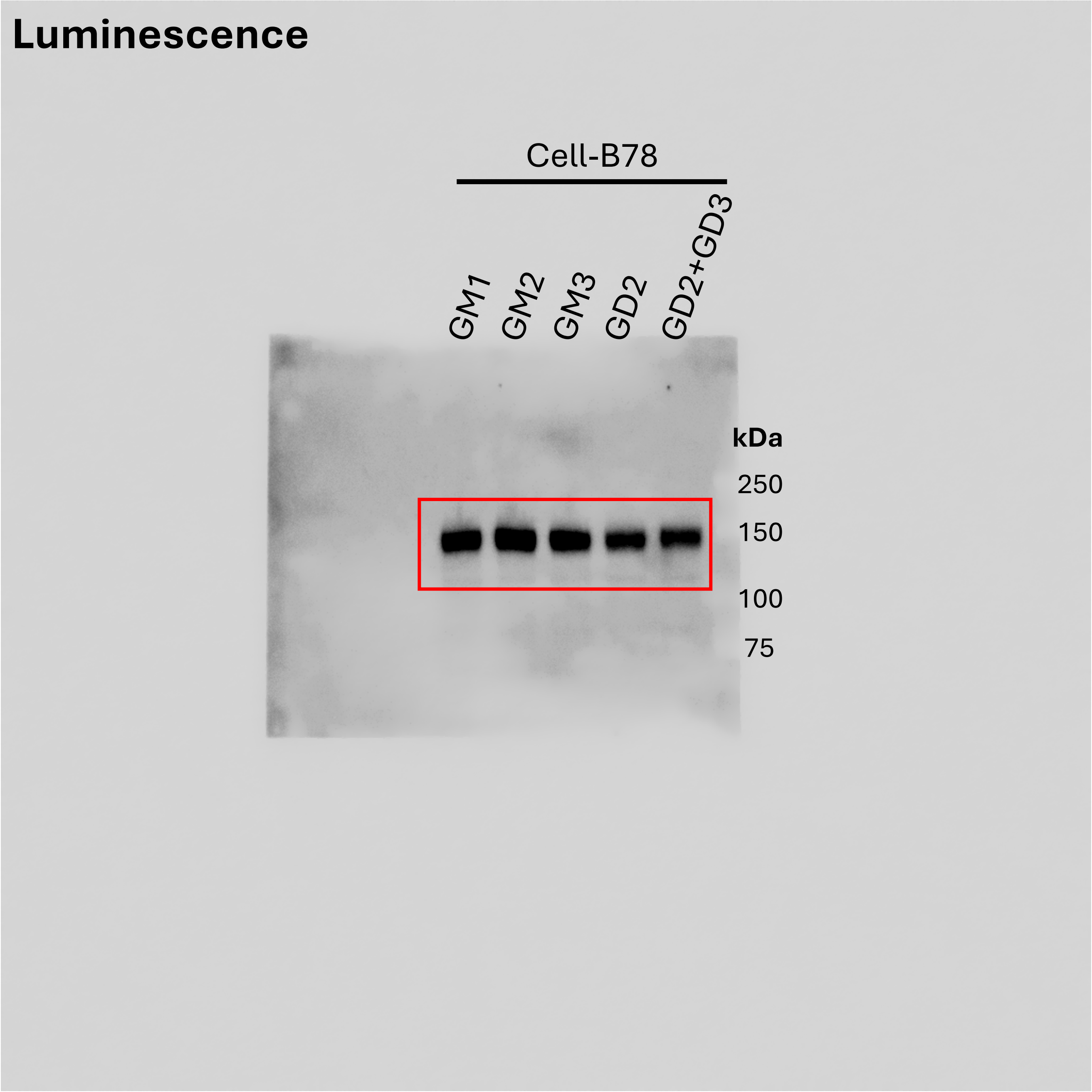

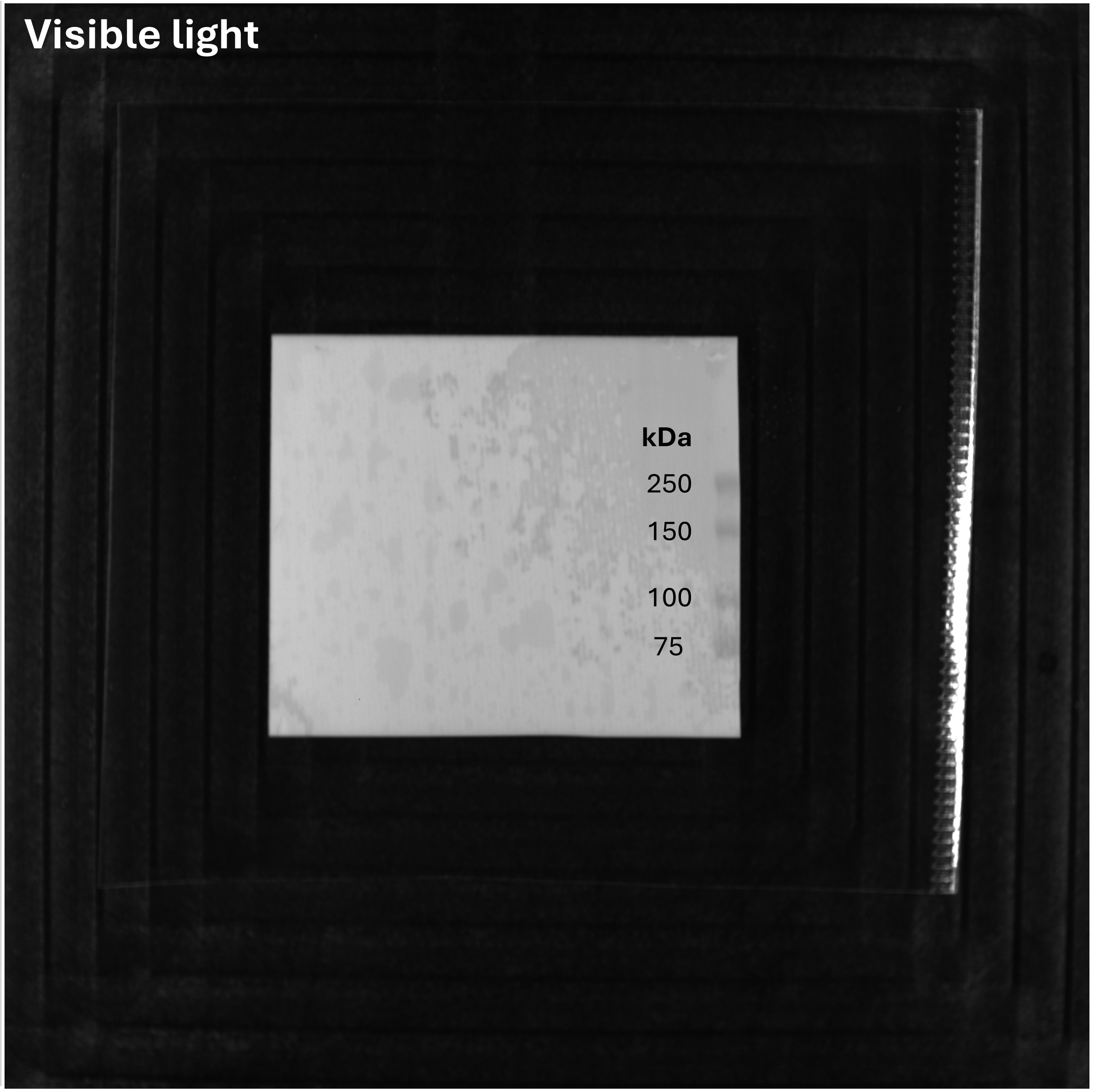

SourceDataSF4C_Cell-B78_actin

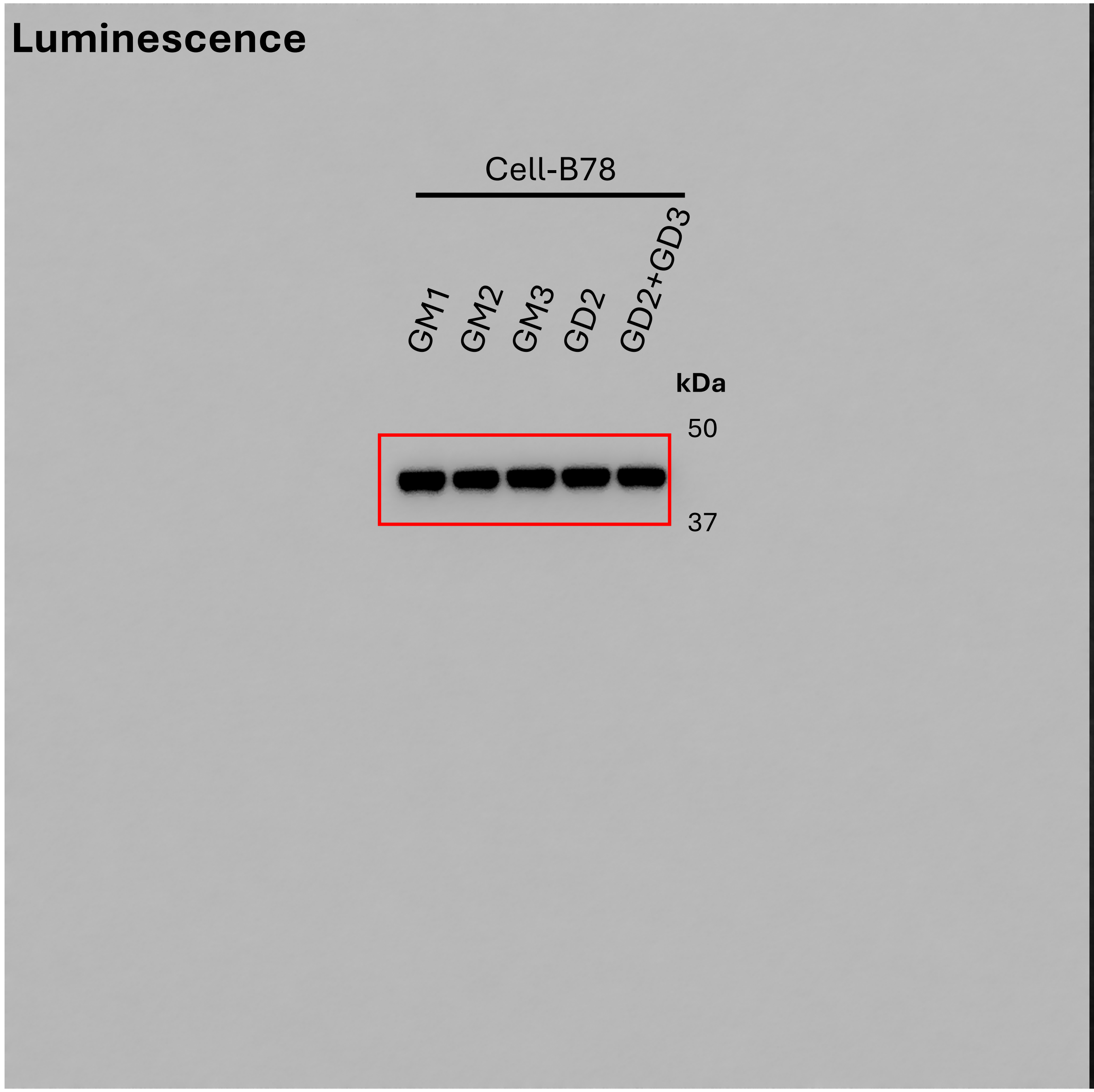

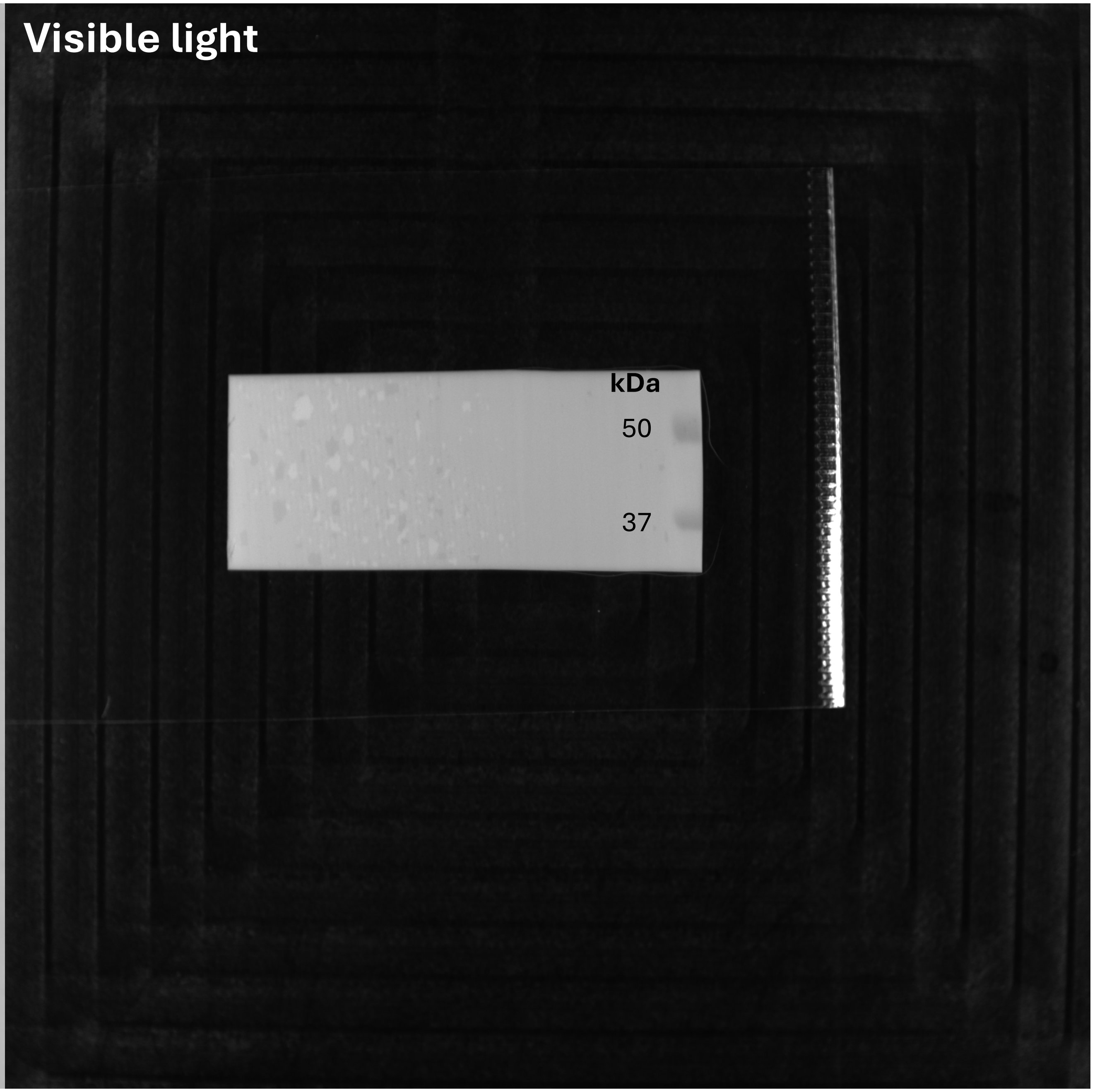

Supplement: SourceData FS4 — is the source file for Fig. S4. [file jcb_202404064_sourcedatafs4.pdf]
